# Supplementary material for: Plant genetic diversity affects multiple trophic levels and trophic interactions
Source: Nat Commun. 2022 Nov 27;13:7312. doi: 10.1038/s41467-022-35087-7 (PMC9701765; doi:10.1038/s41467-022-35087-7)
Supplement: Supplementary file 1 — Supplementary Info File #1 [file 41467_2022_35087_MOESM1_ESM.pdf]

Supplementary information

Supplementary Table 1 Overview of the meta-regression models used to test our research questions. For each model, we used mixed effect model meta-analysis, where “plant species” was included as a random effect with phylogenetic relatedness as part of the correlation structure. The table gives the predictor variables included, Akaike´s An Information Criterion (AIC), the reference model (model 1) for the log-likelihood ratio test, and the statistics of the log-likelihood ratio tests (log-likelihoods [L-L], X^2, degrees of freedom compared to the reference model (Ref.), degrees of freedom [df] and the corresponding P-value) based on model comparisons. Each test is two-sided and the original P value is reported with no multiple comparisons. Model 1 was nested within Model 1.1–1.5; and Model 2 was nested within Model 2.1–2.5. Details on the comparison between Model 1 and Model 2 are provided in Note S3. Ecosystem types included agroecosystems, grasslands, forests, old-field ecosystems, marine ecosystems, wetlands and shrublands. Type of experimental study was divided into plot and pot experiments. Plant life form comprised herbaceous and woody plants. Climatic zone type was divided into tropical and temperate climatic zones. There were 4702 effect sizes from 413 papers.

1.1) The response variable in all models was the effect size of the responses of invertebrate herbivores, natural enemies (predators and parasitoids) of herbivores, weeds, plant-feeding nematodes, plant diseases and plants to the different comparison groups. Trophic group included invertebrate herbivores, natural enemies (predators and parasitoids) of herbivores, weeds, plant-feeding nematodes, plant diseases and plants. Trophic group response category included invertebrate herbivore abundance, herbivore damage, herbivore diversity, predator abundance, predator diversity, parasitoid abundance, parasitoid diversity, parasitism, weed growth, weed diversity, plant-feeding nematode abundance, plant disease spread, plant disease damage, plant growth, plant reproduction, plant quality. Data from greenhouse and other indoor experiments were removed from the models with climatic predictors. The model with suffix “A” is compared with base model (Model 1 or 2). The model with suffix “B” is compared with the nearest model with suffix “A”.

Model	Question	Predictor variables	Ref.	AIC	L–L	X^2	df	P	N
**1**	1	Trophic group	-	15779.9572	-7881.9786	-	8	-	4702
**1.1A**	2	Trophic group + Ecosystem type	1	15766.3524	-7869.1762	25.6048	14	0.0003	4702
**1.1B**	2	Trophic group + Ecosystem type + Trophic group × Ecosystem type	1.1A	15689.2277	-7816.6138	105.1247	28	4.9053×10^{-16}	4702
**1.2A**	2	Trophic group + Type of experimental study	1	15781.8726	-7881.9363	0.0846	9	0.7711	4702
**1.2B**	2	Trophic group + Type of experimental study + Trophic group × Type of experimental study	1.2A	15761.9108	-7867.9554	27.9619	13	1.2697×10^{-5}	4702
**1.3A**	2	Trophic group + Plant life form	1	15780.3600	-7881.1800	1.5972	9	0.2063	4702
**1.3B**	2	Trophic group + Plant life form + Trophic group × Plant life form	1.3A	15756.2424	-7866.1212	30.1176	12	1.3036×10^{-6}	4702
**1.4A**	2	Trophic group + Climatic zone type	1	14604.7037	-7293.3518	3.2202	9	0.0727	4439
**1.4B**	2	Trophic group + Climatic zone type + Trophic group × Climatic zone type	1.4A	14560.9469	-7266.4734	53.7568	14	2.3514×10^{-10}	4439
**1.5A**	1	Trophic group + log_2 (added plant genotypes over control)	1	15773.2674	-7877.6337	8.6899	9	0.0032	4702
**1.5B**	1	Trophic group + log_2 (added plant genotypes over control) + Trophic group × log_2 (added plant genotypes over control)	1.5A	15724.0239	-7848.0119	59.2435	14	1.7419×10^{-11}	4702
**2**	1	Trophic group response category	1	15737.0827	-7850.5414	62.8745	18	1.0316×10^{-9}	4702
**2.1A**	2	Trophic group response category + Ecosystem type	2	15725.7715	-7838.8857	86.1858	24	1.2537×10^{-11}	4702
**2.1B**	2	Trophic group response category + Ecosystem type + Trophic group response category × Ecosystem type	2.1A	15673.9070	-7778.9535	119.8645	58	1.6796×10^{-11}	4702
**2.2A**	2	Trophic group response category + Type of experimental study	2	15738.8580	-7850.4290	63.0993	19	2.4500×10^{-9}	4702
**2.2B**	2	Trophic group response category + Type of experimental study + Trophic group response category × Type of experimental study	2.2A	15714.5857	-7829.2928	42.2723	28	2.9305×10^{-6}	4702
**2.3A**	2	Trophic group response category + Plant life form	2	15737.7351	-7849.8675	64.2222	19	1.5088×10^{-9}	4702
**2.3B**	2	Trophic group response category + Plant life form + Trophic group response category × Plant life form	2.3A	15701.7482	-7819.8741	59.9869	31	2.2698×10^{-8}	4702
**2.4A**	2	Trophic group response category + Climatic zone type	2	14559.3039	-7260.6519	6.0541	19	0.0139	4439
**2.4B**	2	Trophic group response category + Climatic zone type + Trophic group response category × Climatic zone type	2.4A	14522.5440	-7228.2720	64.7599	33	1.6884×10^{-8}	4439
**2.5A**	1	Trophic group response category + log_2 (added plant genotypes over control)	2	15731.9853	-7846.9927	69.9719	19	1.2368×10^{-10}	4702
**2.5B**	1	Trophic group response category + log_2 (added plant genotypes over control) + Trophic group response category × log_2	2.5A	15669.8442	-7801.9221	90.1412	33	3.5691×10^{-13}	4702

(added plant genotypes over control)

1.2) The response variable in all models was the effect size of the responses of plant antagonists, natural enemies of herbivores and plants to the different comparison groups. Trophic group included plant antagonists, natural enemies of herbivores and plants. Trophic group response category included plant antagonist intensity (abundance of herbivores and nematodes, damage of herbivores and plant disease, plant disease spread and weed growth), plant antagonist diversity (weed diversity and herbivore diversity), predator abundance, predator diversity, parasitoid abundance, parasitoid diversity, parasitism, plant growth, plant reproduction, plant quality. Data from greenhouse and other indoor experiments were removed from the models with climatic predictors. The model with suffix "A" is compared with base model (Model 1 or 2). The model with suffix "B" is compared with the nearest model with suffix "A".

Model	Question	Predictor variables	Ref.	AIC	L—L	X^2	df	P	N
**1**	1	Trophic group	-	15940.6807	-7965.3403	-	5	-	4702
**1.1A**	2	Trophic group + Ecosystem type	1	15904.1684	-7941.0842	48.5123	11	9.3342×10^{-9}	4702
**1.1B**	2	Trophic group + Ecosystem type + Trophic group × Ecosystem type	1.1A	15769.6606	-7863.8303	154.5078	21	4.3985×10^{-28}	4702
**1.2A**	2	Trophic group + Type of experimental study	1	15939.6066	-7963.8033	3.0741	6	0.0795	4702
**1.2B**	2	Trophic group + Type of experimental study + Trophic group × Type of experimental study	1.2A	15929.9041	-7956.9520	13.7025	8	0.0011	4702
**1.3A**	2	Trophic group + Plant life form	1	15942.2167	-7965.1084	0.4639	6	0.4958	4702
**1.3B**	2	Trophic group + Plant life form + Trophic group × Plant life form	1.3A	15884.4555	-7934.2277	61.7613	8	3.8789×10^{-14}	4702
**1.4A**	2	Trophic group + Climatic zone type	1	14761.4358	-7374.7179	1.4170	6	0.2339	4439
**1.4B**	2	Trophic group + Climatic zone type + Trophic group × Climatic zone type	1.4A	14727.2101	-7355.6050	38.2257	8	5.0050×10^{-9}	4439
**1.5A**	1	Trophic group + $\log_2$ (added plant genotypes over control)	1	15929.3271	-7958.6635	13.3536	6	0.0003	4702
**1.5B**	1	Trophic group + log2 (added plant genotypes over control) + Trophic group × $\log_2$ (added plant genotypes over control)	1.5A	15923.0228	-7953.5114	10.3043	8	0.0058	4702
**2**	1	Trophic group response category	1	15878.5824	-7927.2912	76.0983	12	8.5795×10^{-14}	4702
**2.1A**	2	Trophic group response category + Ecosystem type	2	15845.4767	-7904.7383	121.2040	18	1.1639×10^{-19}	4702
**2.1B**	2	Trophic group response category + Ecosystem type + Trophic group response category × Ecosystem type	2.1A	15755.4896	-7835.7448	137.9871	42	5.4486×10^{-18}	4702
**2.2A**	2	Trophic group response category + Type of experimental study	2	15875.9365	-7924.9682	80.7442	13	3.4623×10^{-14}	4702
**2.2B**	2	Trophic group response category + Type of experimental study + Trophic group response category × Type of experimental study	2.2A	15866.3719	-7915.1860	19.5645	18	0.0015	4702
**2.3A**	2	Trophic group response category + Plant life form	2	15879.5877	-7926.7939	77.0929	13	1.8772×10^{-13}	4702
**2.3B**	2	Trophic group response category + Plant life form + Trophic group response category × Plant life form	2.3A	15829.1484	-7892.5742	68.4393	22	3.0780×10^{-11}	4702
**2.4A**	2	Trophic group response category + Climatic zone type	2	14699.7239	-7336.8620	2.6810	13	0.1016	4439
**2.4B**	2	Trophic group response category + Climatic zone type + Trophic group response category × Climatic zone type	2.4A	14675.6355	-7316.8177	40.0885	21	3.0844×10^{-6}	4439
**2.5A**	1	Trophic group response category + $\log_2$ (added plant genotypes over control)	2	15869.1278	-7921.5639	87.5529	13	1.4581×10^{-15}	4702
**2.5B**	1	Trophic group response category + $\log_2$ (added plant genotypes over control) + Trophic group response category ×$\log_2$ (added plant genotypes over control)	2.5A	15849.0061	-7903.5030	36.1217	21	1.6682×10^{-5}	4702

Supplementary Table 2 Results of publication bias assessment based on the residuals from the various models. The results include the test-statistics (t value) and P-values of the regression tests. Each test is two-sided and the original P value is reported with no multiple comparisons. Again, we used mixed effects model meta-analysis, where "plant species" was included as random effect with phylogenetic relatedness as part of the correlation structure. The regression test value tests for an association between effect size and the inverse of the sample variance; a significant P value indicates publication bias. Ecosystem type included agroecosystems, grasslands, forests, old-field ecosystems, marine ecosystems, wetlands and shrublands. Type of experimental study was divided into plot and pot experiments. Plant life form comprised herbaceous and woody plants. Climatic zone type was divided into tropical and temperate zones. N, sample size.

2.1) The response variable in all models was the effect size of the responses of invertebrate herbivores, natural enemies (predators and parasitoids) of herbivores, weeds, plant-feeding nematodes, plant diseases and plants to the different comparison groups. Trophic group included invertebrate herbivores, natural enemies (predators and parasitoids) of herbivores, weeds, plant-feeding nematodes, plant diseases and plants. Trophic group response category included invertebrate herbivore abundance, herbivore damage, herbivore diversity, predator abundance, predator diversity, parasitoid abundance, parasitoid diversity, parasitism, weed growth, weed diversity, plant-feeding nematode abundance, plant disease spread, plant disease damage, plant growth, plant reproduction, and plant quality. Data from greenhouse and other indoor experiments were removed from the models with climatic predictors. The model with suffix "A" is compared with base model (Model 1 or 2). The model with suffix "B" is compared with the nearest model with suffix "A".

Model	Predictor variables	N	Regression test value	*P*-value
1	Trophic group	4702	-32.3545	1.2002×10^{-229}
**1.1A**	Trophic group + Ecosystem type	4702	-32.2451	4.1225×10^{-228}
**1.1B**	Trophic group + Ecosystem type + Trophic group × Ecosystem type	4702	-0.5602	0.5753
**1.2A**	Trophic group + Type of experimental study	4702	-32.3577	1.0807×10^{-229}
**1.2B**	Trophic group + Type of experimental study + Trophic group × Type of experimental study	4702	1.2560	0.2091
**1.3A**	Trophic group + Plant life form	4702	-32.3847	4.5109×10^{-230}
**1.3B**	Trophic group + Plant life form + Trophic group × Plant life form	4702	-3.2644	0.0011
**1.4A**	Trophic group + Climatic zone type	4439	-31.6212	1.8875×10^{-219}
**1.4B**	Trophic group + Climatic zone type + Trophic group × Climatic zone type	4439	1.4996	0.1337
**1.5A**	Trophic group + $\log_2$ (added plant genotypes over control)	4702	-32.3999	2.75621×10^{-230}
**1.5B**	Trophic group + $\log_2$ (added plant genotypes over control) + Trophic group × $\log_2$ (added plant genotypes over control)	4702	3.5127	0.0004
**2**	Trophic group response category	4702	-32.3873	4.14509×10^{-230}
**2.1A**	Trophic group response category + Ecosystem type	4702	-32.2700	1.84600×10^{-228}
**2.1B**	Trophic group response category + Ecosystem type + Trophic group response category × Ecosystem type	4702	0.1329	0.8943
**2.2A**	Trophic group response category + Type of experimental study	4702	-32.3866	4.23672×10^{-230}
**2.2B**	Trophic group response category + Type of experimental study + Trophic group response category × Type of experimental study	4702	0.9632	0.3354
**2.3A**	Trophic group response category + Plant life form	4702	-32.4240	1.2580×10^{-230}
**2.3B**	Trophic group response category + Plant life form + Trophic group response category × Plant life form	4702	-3.0293	0.0025
**2.4A**	Trophic group response category + Climatic zone type	4439	-31.6696	4.07691×10^{-220}
**2.4B**	Trophic group response category + Climatic zone type + Trophic group response category × Climatic zone type	4439	0.7799	0.4355
**2.5A**	Trophic group response category + $\log_2$ (added plant genotypes over control)	4702	-32.4371	8.23037×10^{-231}
**2.5B**	Trophic group response category + $\log_2$ (added plant genotypes over control) + Trophic group response category ×$\log_2$ (added plant genotypes over control)	4702	3.4010	0.0007

2.2) The response variable in all models was the effect size of the responses of plant antagonists, natural enemies of herbivores and plants to the different comparison groups. Trophic group included plant antagonists, natural enemies of herbivores and plants. Trophic group response categories included plant antagonist intensity (abundance of herbivores and nematodes, damage of herbivores and plant diseases, plant disease spread and weed growth), plant antagonist diversity (weed diversity and herbivore diversity), predator abundance, predator diversity, parasitoid abundance, parasitoid diversity, parasitism, plant growth, plant reproduction, plant quality. Data from greenhouse and other indoor experiments were removed from the models with climatic predictors. The model with suffix "A" is compared with base model (Model 1 or 2). The model with suffix "B" is compared with the nearest model with suffix "A".

Model	Predictor variables	N	Regression test value	*P*-value
1	Trophic group	4702	-33.4308	4.8925×10^{-245}
**1.1A**	Trophic group + Ecosystem type	4702	-33.2284	4.1817×10^{-242}
**1.1B**	Trophic group + Ecosystem type + Trophic group ×Ecosystem type	4702	0.5135	0.6076
**1.2A**	Trophic group + Type of experimental study	4702	-33.3904	1.8875×10^{-244}
**1.2B**	Trophic group + Type of experimental study + Trophic group × Type of experimental study	4702	0.1537	0.8778
**1.3A**	Trophic group + Plant life form	4702	-33.4640	1.6114×10^{-245}
**1.3B**	Trophic group + Plant life form + Trophic group × Plant life form	4702	4.7848	1.7115×10^{-6}
**1.4A**	Trophic group + Climatic zone type	4439	-32.7242	7.0765×10^{-235}
**1.4B**	Trophic group + Climatic zone type + Trophic group × Climatic zone type	4439	3.7652	0.0002
**1.5A**	Trophic group + $\log_2$ (added plant genotypes over control)	4702	-33.4751	1.1088×10^{-245}
**1.5B**	Trophic group + $\log_2$ (added plant genotypes over control) + Trophic group × $\log_2$ (added plant genotypes over control)	4702	1.0164	0.3095
**2**	Trophic group response category	4702	-33.3877	2.0705×10^{-244}
**2.1A**	Trophic group response category + Ecosystem type	4702	-33.1814	1.9951×10^{-241}
**2.1B**	Trophic group response category + Ecosystem type + Trophic group response category × Ecosystem type	4702	0.1479	0.8824
**2.2A**	Trophic group response category + Type of experimental study	4702	-33.3293	1.4555×10^{-243}
**2.2B**	Trophic group response category + Type of experimental study + Trophic group response category × Type of experimental study	4702	2.8986	0.0037
**2.3A**	Trophic group response category + Plant life form	4702	-33.4275	5.4691×10^{-245}
**2.3B**	Trophic group response category + Plant life form + Trophic group response category × Plant life form	4702	0.4618	0.6443
**2.4A**	Trophic group response category + Climatic zone type	4439	-32.7126	1.0332×10^{-234}
**2.4B**	Trophic group response category + Climatic zone type + Trophic group response category × Climatic zone type	4439	2.6349	0.0084
**2.5A**	Trophic group response category + $\log_2$ (added plant genotypes over control)	4702	-33.4386	3.7638×10^{-245}
**2.5B**	Trophic group response category + $\log_2$ (added plant genotypes over control) + Trophic group response category ×$\log_2$ (added plant genotypes over control)	4702	2.3505	0.0188

Supplementary Table 3 Results of the meta-regression analysis for the trophic groups and their response categories (as shown also in Fig. 1b). Each test is two-sided and the original P value is reported with no multiple comparisons. In addition to the estimated mean effect sizes, test-statistics (t-value), and the associated P-values, the number of studies and observations available for each predictor category are also presented.

Category	Number of observations	Number of Studies	Effect size	t-value	P-value
Total response of plant antagonist performance	1736	236	-0.5392	-2.0699	0.0385
Total response of invertebrate herbivore performance	468	93	-0.6061	-4.1267	3.6793×10^{-5}
Total response of performance of herbivore natural enemies	104	33	0.7777	4.2196	2.4478×10^{-5}
Total response of weed performance	200	14	-0.0709	-0.1673	0.8671
Total response of plant-feeding nematode performance	35	7	-2.1180	-1.3125	0.1894
Total response of plant disease performance	1033	136	-1.0866	-5.8256	5.6902×10^{-9}
Total response of plant performance	2862	320	0.3439	9.0976	9.2381×10^{-20}
Plant antagonist intensity response	1709	233	-0.5248	-1.8307	0.0671
Plant antagonist diversity response	27	8	0.0363	0.1195	0.9049
Herbivore abundance response	321	70	-0.6448	-3.9262	0.0001
Herbivore damage response	138	35	-0.5675	-2.6986	0.0070
Herbivore diversity response	9	6	0.2924	0.9913	0.3215
Predator abundance response	53	21	1.1968	5.4912	3.9926×10^{-8}
Predator diversity response	19	8	1.0714	2.8804	0.0040
Parasitoid abundance response	11	5	0.7530	1.7604	0.0783
Parasitoid diversity response	5	2	0.6500	2.0914	0.0365
Parasitism response	16	6	0.0893	0.1688	0.8659
Weed growth response	182	13	-0.0608	-0.1428	0.8865
Weed diversity response	18	2	-0.7615	-1.5701	0.1164
Plant-feeding nematode abundance response	35	7	-2.1180	-1.3125	0.1894
Plant disease spread response	591	75	-1.1835	-2.9021	0.0037
Plant disease damage response	442	87	-1.1960	-5.1023	3.3548×10^{-7}
Plant growth response	726	128	0.2994	5.6978	1.2138×10^{-8}
Plant quality response	318	44	0.1152	2.0019	0.0453
Plant reproduction response	1818	255	0.4811	8.0398	8.9997×10^{-16}

Supplementary Table 4 Results of the meta-regression analysis for the seven trophic groups and their response categories for different ecosystems (agroecosystems, grasslands, forests, old-field ecosystems, marine ecosystems, wetlands and shrublands) (as shown also in Fig. 4). In addition to the estimated mean effect sizes, test-statistics (t-value), and the associated P-values, the number of studies and observations available for each predictor category are also presented. Each test is two-sided and the original P value is reported with no multiple comparisons.

Ecosystem type	Category	Number of observations	Number of Studies	Effect size	t-value	P-value
Agroecosystem	Total plant antagonist performance	1536	195	-0.8785	-6.6770	2.4395×10^{-11}
	Total invertebrate herbivore performance	310	57	-1.0075	-5.4189	5.9975×10^{-8}
	Total performance of herbivore natural enemies	52	17	0.6758	1.1416	0.2536
	Total weed performance	178	12	-0.5821	-3.3504	0.0008
	Total plant-feeding nematode performance	35	7	-2.1180	-1.3125	0.1894
	Total plant disease performance	1013	131	-1.0850	-5.2113	1.8755×10^{-7}
	Total plant performance	2538	267	0.3619	8.2750	1.2842×10^{-16}
	Plant antagonist intensity response	1517	194	-0.9162	-7.1618	7.9647×10^{-13}
	Plant antagonist diversity response	19	3	-0.5368	-1.5274	0.1267
	Herbivore abundance response	222	45	-0.9331	-4.6176	3.8812×10^{-6}
	Herbivore damage response	87	18	-1.0734	-3.5704	0.0004
	Herbivore diversity response	N/A	N/A	N/A	N/A	N/A
	Predator abundance response	25	11	1.5606	4.1211	3.7700×10^{-5}
	Predator diversity response	5	1	0.8686	2.6869	0.0072
	Parasitoid abundance response	7	3	1.0076	1.2847	0.1989
	Parasitoid diversity response	N/A	N/A	N/A	N/A	N/A
	Parasitism response	15	5	0.2675	0.4783	0.6324
	Weed growth response	160	11	-0.5437	-2.5620	0.0104
	Weed diversity response	18	2	-0.7615	-1.5701	0.1164
	Plant-feeding nematode abundance response	35	7	-2.1180	-1.3125	0.1894
	Plant disease spread response	586	74	-1.1930	-2.3768	0.0175
	Plant disease damage response	427	83	-1.2382	-4.5785	4.6836×10^{-6}
	Plant growth response	510	85	0.3338	3.8529	0.0001
	Plant quality response	290	41	0.1310	1.7685	0.0770
	Plant reproduction response	1738	241	0.4676	6.6284	3.3938×10^{-11}
Grassland	Total plant antagonist performance	55	9	-0.6612	-1.8706	0.0614
	Total invertebrate herbivore performance	25	5	-0.9614	-2.2319	0.0256
	Total performance of herbivore natural enemies	20	3	1.0041	4.5133	6.3841×10^{-6}
	Total weed performance	22	2	0.2033	0.2362	0.8133
	Total plant-feeding nematode performance	N/A	N/A	N/A	N/A	N/A
	Total plant disease performance	8	3	-1.1609	-3.0311	0.0024
	Total plant performance	134	17	0.3533	4.6700	3.0118×10^{-6}
	Plant antagonist intensity response	55	9	-0.6612	-1.8706	0.0614
	Plant antagonist diversity response	N/A	N/A	N/A	N/A	N/A
	Herbivore abundance response	19	4	-1.1306	-2.7543	0.0059
	Herbivore damage response	6	3	-0.8567	-0.8328	0.4049
	Herbivore diversity response	N/A	N/A	N/A	N/A	N/A
	Predator abundance response	14	2	1.2439	4.0860	4.3894×10^{-5}
	Predator diversity response	N/A	N/A	N/A	N/A	N/A
	Parasitoid abundance response	2	1	0.5880	1.2639	0.2063
	Parasitoid diversity response	4	1	0.8655	1.8947	0.0581
	Parasitism response	N/A	N/A	N/A	N/A	N/A
	Weed growth response	22	2	0.2033	0.2362	0.8133
	Weed diversity response	N/A	N/A	N/A	N/A	N/A
	Plant-feeding nematode abundance response	N/A	N/A	N/A	N/A	N/A
	Plant disease spread response	5	1	-1.3108	-2.5167	0.0118
	Plant disease damage response	3	2	-0.9823	-1.6959	0.0899
	Plant growth response	52	12	0.2561	2.0384	0.0415
	Plant quality response	18	1	0.0100	0.0586	0.9532
	Plant reproduction response	64	7	0.5919	3.7431	0.0002
Forest	Total plant antagonist performance	89	14	-0.0923	-0.4616	0.6444
	Total invertebrate herbivore performance	79	14	-0.1508	-0.6788	0.4973
	Total performance of herbivore natural enemies	7	5	0.1136	0.6015	0.5475
	Total weed performance	N/A	N/A	N/A	N/A	N/A
	Total plant-feeding nematode performance	N/A	N/A	N/A	N/A	N/A
	Total plant disease performance	10	1	-0.3037	-1.0790	0.2806
	Total plant performance	61	4	0.1333	0.6028	0.5467
	Plant antagonist intensity response	85	13	-0.0654	-0.2368	0.8128
	Plant antagonist diversity response	4	3	-0.0704	-0.2199	0.8260
	Herbivore abundance response	52	8	-0.3471	-3.0402	0.0024
	Herbivore damage response	23	9	-0.2722	-0.7328	0.4637
	Herbivore diversity response	4	3	-0.0997	-0.2575	0.7968
	Predator abundance response	3	3	0.1733	0.4764	0.6338
	Predator diversity response	2	2	0.4727	0.9831	0.3256

	Parasitoid abundance response	N/A	N/A	N/A	N/A	N/A
	Parasitoid diversity response	N/A	N/A	N/A	N/A	N/A
	Parasitism response	N/A	N/A	N/A	N/A	N/A
	Weed growth response	N/A	N/A	N/A	N/A	N/A
	Weed diversity response	N/A	N/A	N/A	N/A	N/A
	Plant-feeding nematode abundance response	N/A	N/A	N/A	N/A	N/A
	Plant disease spread response	N/A	N/A	N/A	N/A	N/A
	Plant disease damage response	10	1	-0.3037	-1.0790	0.2806
	Plant growth response	57	4	0.1244	0.5272	0.5981
	Plant quality response	4	1	0.2055	1.0974	0.2725
	Plant reproduction response	N/A	N/A	N/A	N/A	N/A
Old-field ecosystem	Total plant antagonist performance	20	10	0.9726	2.3269	0.0200
	Total invertebrate herbivore performance	18	9	1.1018	1.2242	0.2209
	Total performance of herbivore natural enemies	14	4	1.8404	3.4054	0.0007
	Total weed performance	N/A	N/A	N/A	N/A	N/A
	Total plant-feeding nematode performance	N/A	N/A	N/A	N/A	N/A
	Total plant disease performance	2	1	-2.6589	-3.1762	0.0015
	Total plant performance	27	11	0.6805	5.5731	2.5022×10^{-8}
	Plant antagonist intensity response	16	9	0.9684	1.0458	0.2957
	Plant antagonist diversity response	4	2	1.0136	3.3837	0.0007
	Herbivore abundance response	11	7	1.2895	1.2197	0.2226
	Herbivore damage response	3	2	-0.0584	-0.1590	0.8737
	Herbivore diversity response	4	2	1.0136	3.3837	0.0007
	Predator abundance response	5	2	1.4984	1.7405	0.0818
	Predator diversity response	9	4	2.1044	6.5369	6.2807×10^{-11}
	Parasitoid abundance response	N/A	N/A	N/A	N/A	N/A
	Parasitoid diversity response	N/A	N/A	N/A	N/A	N/A
	Parasitism response	N/A	N/A	N/A	N/A	N/A
	Weed growth response	N/A	N/A	N/A	N/A	N/A
	Weed diversity response	N/A	N/A	N/A	N/A	N/A
	Plant-feeding nematode abundance response	N/A	N/A	N/A	N/A	N/A
	Plant disease spread response	N/A	N/A	N/A	N/A	N/A
	Plant disease damage response	2	1	-2.6589	-3.1762	0.0015
	Plant growth response	17	7	0.6752	4.2833	1.8417×10^{-5}
	Plant quality response	N/A	N/A	N/A	N/A	N/A
	Plant reproduction response	10	6	0.6884	3.5660	0.0004
Marine ecosystem	Total plant antagonist performance	13	5	-0.2886	-1.7652	0.0775
	Total invertebrate herbivore performance	13	5	-0.2886	-1.7652	0.0775
	Total performance of herbivore natural enemies	N/A	N/A	N/A	N/A	N/A
	Total weed performance	N/A	N/A	N/A	N/A	N/A
	Total plant-feeding nematode performance	N/A	N/A	N/A	N/A	N/A
	Total plant disease performance	N/A	N/A	N/A	N/A	N/A
	Total plant performance	37	9	0.7779	3.7529	0.0002
	Plant antagonist intensity response	13	5	-0.2886	-1.7652	0.0775
	Plant antagonist diversity response	N/A	N/A	N/A	N/A	N/A
	Herbivore abundance response	8	3	-0.1536	-0.7081	0.4789
	Herbivore damage response	5	2	-0.4658	-1.8733	0.0610
	Herbivore diversity response	N/A	N/A	N/A	N/A	N/A
	Predator abundance response	N/A	N/A	N/A	N/A	N/A
	Predator diversity response	N/A	N/A	N/A	N/A	N/A
	Parasitoid abundance response	N/A	N/A	N/A	N/A	N/A
	Parasitoid diversity response	N/A	N/A	N/A	N/A	N/A
	Parasitism response	N/A	N/A	N/A	N/A	N/A
	Weed growth response	N/A	N/A	N/A	N/A	N/A
	Weed diversity response	N/A	N/A	N/A	N/A	N/A
	Plant-feeding nematode abundance response	N/A	N/A	N/A	N/A	N/A
	Plant disease spread response	N/A	N/A	N/A	N/A	N/A
	Plant disease damage response	N/A	N/A	N/A	N/A	N/A
	Plant growth response	37	9	0.7779	3.7529	0.0002
	Plant quality response	N/A	N/A	N/A	N/A	N/A
	Plant reproduction response	N/A	N/A	N/A	N/A	N/A
Wetland	Total plant antagonist performance	N/A	N/A	N/A	N/A	N/A
	Total invertebrate herbivore performance	N/A	N/A	N/A	N/A	N/A
	Total performance of herbivore natural enemies	3	1	0.2273	0.4134	0.6793
	Total weed performance	N/A	N/A	N/A	N/A	N/A
	Total plant-feeding nematode performance	N/A	N/A	N/A	N/A	N/A
	Total plant disease performance	N/A	N/A	N/A	N/A	N/A
	Total plant performance	43	9	0.2064	0.9669	0.3336
	Plant antagonist intensity response	N/A	N/A	N/A	N/A	N/A
	Plant antagonist diversity response	N/A	N/A	N/A	N/A	N/A
	Herbivore abundance response	N/A	N/A	N/A	N/A	N/A
	Herbivore damage response	N/A	N/A	N/A	N/A	N/A
	Herbivore diversity response	N/A	N/A	N/A	N/A	N/A
	Predator abundance response	N/A	N/A	N/A	N/A	N/A
	Predator diversity response	3	1	0.2273	0.4134	0.6793

	Parasitoid abundance response	N/A	N/A	N/A	N/A	N/A
	Parasitoid diversity response	N/A	N/A	N/A	N/A	N/A
	Parasitism response	N/A	N/A	N/A	N/A	N/A
	Weed growth response	N/A	N/A	N/A	N/A	N/A
	Weed diversity response	N/A	N/A	N/A	N/A	N/A
	Plant-feeding nematode abundance response	N/A	N/A	N/A	N/A	N/A
	Plant disease spread response	N/A	N/A	N/A	N/A	N/A
	Plant disease damage response	N/A	N/A	N/A	N/A	N/A
	Plant growth response	37	8	0.2455	1.0115	0.3118
	Plant quality response	N/A	N/A	N/A	N/A	N/A
	Plant reproduction response	6	1	-0.0902	-0.2521	0.8010
Shrubland	Total plant antagonist performance	23	3	0.1779	0.9116	0.3620
	Total invertebrate herbivore performance	23	3	0.1779	0.9116	0.3620
	Total performance of herbivore natural enemies	8	3	0.6626	2.1728	0.0298
	Total weed performance	N/A	N/A	N/A	N/A	N/A
	Total plant-feeding nematode performance	N/A	N/A	N/A	N/A	N/A
	Total plant disease performance	N/A	N/A	N/A	N/A	N/A
	Total plant performance	22	3	0.1580	0.5941	0.5524
	Plant antagonist intensity response	23	3	0.1779	0.9116	0.3620
	Plant antagonist diversity response	N/A	N/A	N/A	N/A	N/A
	Herbivore abundance response	9	3	0.0223	0.1516	0.8795
	Herbivore damage response	14	1	0.4394	2.9282	0.0034
	Herbivore diversity response	N/A	N/A	N/A	N/A	N/A
	Predator abundance response	6	3	0.7057	1.9710	0.0487
	Predator diversity response	N/A	N/A	N/A	N/A	N/A
	Parasitoid abundance response	2	1	0.5486	0.9429	0.3457
	Parasitoid diversity response	N/A	N/A	N/A	N/A	N/A
	Parasitism response	N/A	N/A	N/A	N/A	N/A
	Weed growth response	N/A	N/A	N/A	N/A	N/A
	Weed diversity response	N/A	N/A	N/A	N/A	N/A
	Plant-feeding nematode abundance response	N/A	N/A	N/A	N/A	N/A
	Plant disease spread response	N/A	N/A	N/A	N/A	N/A
	Plant disease damage response	N/A	N/A	N/A	N/A	N/A
	Plant growth response	16	3	0.1807	0.6821	0.4952
	Plant quality response	6	1	-0.0650	-0.2315	0.8169
	Plant reproduction response	N/A	N/A	N/A	N/A	N/A

Supplementary Table 5 Results of the meta-regression analysis for the seven trophic groups and their response categories for two types of experimental study (plot and pot experiments) (as shown also in Fig. 5). In addition to the estimated mean effect sizes, test-statistics (t-value), and the associated P-values, the number of studies and observations available for each predictor category are also presented. Each test is two-sided and the original P value is reported with no multiple comparisons.

Type of experimental study	Category	Number of observations	Number of Studies	Effect size	t-value	P-value
Plot experiment	Total plant antagonist performance	1582	216	-0.3954	-0.7231	0.4696
	Total invertebrate herbivore performance	406	78	-0.6277	-3.9446	7.9918×10^{-5}
	Total performance of herbivore natural enemies	100	32	0.7735	3.9787	6.9283×10^{-5}
	Total weed performance	131	13	-0.0621	-0.1478	0.8825
	Total plant-feeding nematode performance	35	7	-2.1180	-1.3125	0.1894
	Total plant disease performance	1010	132	-1.1231	-4.3422	1.4103×10^{-5}
	Total plant performance	2667	286	0.3625	8.5367	1.3814×10^{-17}
	Plant antagonist intensity response	1557	214	-0.3948	-0.6906	0.4898
	Plant antagonist diversity response	25	7	-0.0114	-0.0352	0.9719
	Herbivore abundance response	294	60	-0.6313	-3.6348	0.0003
	Herbivore damage response	105	29	-0.5900	-2.3228	0.0202
	Herbivore diversity response	7	5	0.2979	0.8487	0.3961
	Predator abundance response	53	21	1.1968	5.4912	3.9926×10^{-8}
	Predator diversity response	19	8	1.0714	2.8804	0.0040
	Parasitoid abundance response	11	5	0.7530	1.7604	0.0783
	Parasitoid diversity response	N/A	N/A	N/A	N/A	N/A
	Parasitism response	16	6	0.0893	0.1688	0.8659
	Weed growth response	113	12	-0.0594	-0.1389	0.8895
	Weed diversity response	18	2	-0.7615	-1.5701	0.1164
	Plant-feeding nematode abundance response	35	7	-2.1180	-1.3125	0.1894
	Plant disease spread response	574	73	-1.1810	-2.8892	0.0039
	Plant disease damage response	436	85	-1.2551	-5.1330	2.8510×10^{-7}
	Plant growth response	623	101	0.3244	5.4459	5.1535×10^{-8}
	Plant quality response	297	42	0.0809	2.0524	0.0401
	Plant reproduction response	1747	242	0.4876	7.4397	1.0090×10^{-13}
Pot experiment	Total plant antagonist performance	154	24	-0.6230	-1.2485	0.2119
	Total invertebrate herbivore performance	62	16	-0.3077	-0.8224	0.4109
	Total performance of herbivore natural enemies	4	1	0.8655	1.8947	0.0581
	Total weed performance	69	3	-0.8187	-6.4918	8.4808×10^{-11}
	Total plant-feeding nematode performance	N/A	N/A	N/A	N/A	N/A
	Total plant disease performance	23	5	-1.8871	-1.2167	0.2237
	Total plant performance	195	34	0.3967	3.6388	0.0003
	Plant antagonist intensity response	152	23	-0.6432	-1.2699	0.2041
	Plant antagonist diversity response	2	1	0.2431	0.4823	0.6296
	Herbivore abundance response	27	10	-0.4785	-1.1834	0.2367
	Herbivore damage response	33	7	-0.1256	-0.2964	0.7669
	Herbivore diversity response	2	1	0.2431	0.4823	0.6296
	Predator abundance response	N/A	N/A	N/A	N/A	N/A
	Predator diversity response	N/A	N/A	N/A	N/A	N/A
	Parasitoid abundance response	N/A	N/A	N/A	N/A	N/A
	Parasitoid diversity response	4	1	0.8655	1.8947	0.0581
	Parasitism response	N/A	N/A	N/A	N/A	N/A
	Weed growth response	69	3	-0.8187	-6.4918	8.4808×10^{-11}
	Weed diversity response	N/A	N/A	N/A	N/A	N/A
	Plant-feeding nematode abundance response	N/A	N/A	N/A	N/A	N/A
	Plant disease spread response	17	3	-2.1647	-6.5567	5.5012×10^{-11}
	Plant disease damage response	6	2	-2.2650	-0.9865	0.3239
	Plant growth response	103	27	0.3196	2.6242	0.0087
	Plant quality response	21	2	0.5962	2.8035	0.0051
	Plant reproduction response	71	13	0.6495	2.5947	0.0095

Supplementary Table 6 Results of the meta-regression analysis for the seven trophic groups and their response categories for two plant life forms (herbaceous plants and woody plants) (as shown also in Fig. 6). In addition to the estimated mean effect sizes, test-statistics (t-value), and the associated P-values, the number of studies and observations available for each predictor category are also presented. Each test is two-sided and the original P value is reported with no multiple comparisons.

Plant life form	Category	Number of observations	Number of Studies	Effect size	t-value	P-value
Herbaceous plant	Total plant antagonist performance	1581	207	-0.5722	-1.3647	0.1723
	Total invertebrate herbivore performance	336	65	-0.7032	-4.1377	3.5088×10^{-5}
	Total performance of herbivore natural enemies	84	23	0.8167	3.7356	0.0002
	Total weed performance	200	14	-0.0709	-0.1673	0.8671
	Total plant-feeding nematode performance	35	7	-2.1180	-1.3125	0.1894
	Total plant disease performance	1010	131	-1.1744	-6.8910	5.5397×10^{-12}
	Total plant performance	2741	306	0.3870	8.9148	4.8890×10^{-19}
	Plant antagonist intensity response	1558	205	-0.6095	-1.5086	0.1314
	Plant antagonist diversity response	23	5	0.0311	0.0729	0.9419
	Herbivore abundance response	232	49	-0.6061	-3.2475	0.0012
	Herbivore damage response	99	24	-0.9485	-3.6320	0.0003
	Herbivore diversity response	5	3	0.6457	1.4910	0.1360
	Predator abundance response	39	13	1.3364	5.8626	4.5566×10^{-9}
	Predator diversity response	17	6	1.3586	2.4058	0.0161
	Parasitoid abundance response	9	4	0.7767	1.3768	0.1686
	Parasitoid diversity response	4	1	0.8655	1.8947	0.0581
	Parasitism response	15	5	0.2675	0.4783	0.6324
	Weed growth response	182	13	-0.0608	-0.1428	0.8865
	Weed diversity response	18	2	-0.7615	-1.5701	0.1164
	Plant-feeding nematode abundance response	35	7	-2.1180	-1.3125	0.1894
	Plant disease spread response	584	71	-1.2195	-2.6179	0.0088
	Plant disease damage response	426	84	-1.3153	-4.9702	6.6880×10^{-7}
	Plant growth response	646	119	0.3658	5.6978	1.2134×10^{-8}
	Plant quality response	307	41	0.1146	1.7175	0.0859
	Plant reproduction response	1788	248	0.4873	7.4198	1.1731×10^{-13}
Woody plant	Total plant antagonist performance	155	29	-0.3931	-1.6345	0.1021
	Total invertebrate herbivore performance	132	28	-0.5146	-1.7636	0.0778
	Total performance of herbivore natural enemies	20	10	0.6561	1.8212	0.0686
	Total weed performance	N/A	N/A	N/A	N/A	N/A
	Total plant-feeding nematode performance	N/A	N/A	N/A	N/A	N/A
	Total plant disease performance	23	5	-0.7930	-0.6864	0.4925
	Total plant performance	121	14	0.1935	1.4127	0.1577
	Plant antagonist intensity response	151	28	-0.4597	-1.6511	0.0987
	Plant antagonist diversity response	4	3	-0.0704	-0.2199	0.8260
	Herbivore abundance response	89	21	-0.9308	-2.2941	0.0218
	Herbivore damage response	39	11	-0.0137	-0.0467	0.9627
	Herbivore diversity response	4	3	-0.0997	-0.2575	0.7968
	Predator abundance response	14	8	0.9073	1.9840	0.0473
	Predator diversity response	2	2	0.4727	0.9831	0.3256
	Parasitoid abundance response	2	1	0.5486	0.9429	0.3457
	Parasitoid diversity response	N/A	N/A	N/A	N/A	N/A
	Parasitism response	N/A	N/A	N/A	N/A	N/A
	Weed growth response	N/A	N/A	N/A	N/A	N/A
	Weed diversity response	N/A	N/A	N/A	N/A	N/A
	Plant-feeding nematode abundance response	N/A	N/A	N/A	N/A	N/A
	Plant disease spread response	7	4	-0.9104	-0.6418	0.5210
	Plant disease damage response	16	3	-0.4965	-1.9282	0.0538
	Plant growth response	80	9	0.1451	0.8491	0.3958
	Plant quality response	11	3	0.1380	0.9059	0.3650
	Plant reproduction response	30	7	0.4362	1.6906	0.0909

Supplementary Table 7 Results of the meta-regression analysis for the seven trophic groups and their response categories for two climatic zones (temperate and tropical climatic zones) (as shown also in Fig. 7). In addition to the estimated mean effect sizes, test-statistics (t-value), and the associated P-values, the number of studies and observations available for each predictor category are also presented. Data from greenhouse and other indoor experiments have been removed from the models with climatic predictors. Each test is two-sided and the original P value is reported with no multiple comparisons.

Climatic zone type	Category	Number of observations	Number of Studies	Effect size	t-value	P-value
Temperate zone	Total plant antagonist performance	1490	192	-0.5875	-2.0265	0.0427
	Total invertebrate herbivore performance	384	66	-0.5837	-3.2895	0.0010
	Total performance of herbivore natural enemies	85	23	0.9887	5.7467	9.1006×10^{-9}
	Total weed performance	138	11	-0.0612	-0.1462	0.8838
	Total plant-feeding nematode performance	29	6	-3.2725	-1.2976	0.1944
	Total plant disease performance	939	119	-1.1934	-5.4873	4.0805×10^{-8}
	Total plant performance	2516	266	0.3449	7.8416	4.4474×10^{-15}
	Plant antagonist intensity response	1468	189	-0.5799	-1.7605	0.0783
	Plant antagonist diversity response	22	6	0.0424	0.0818	0.9348
	Herbivore abundance response	261	49	-0.6510	-3.2959	0.0010
	Herbivore damage response	115	24	-0.4123	-1.7039	0.0884
	Herbivore diversity response	8	5	0.2700	0.7698	0.4414
	Predator abundance response	44	15	1.1635	6.1498	7.7569×10^{-10}
	Predator diversity response	19	8	1.0714	2.8804	0.0040
	Parasitoid abundance response	9	3	1.0535	2.0944	0.0362
	Parasitoid diversity response	4	1	0.8655	1.8947	0.0581
	Parasitism response	9	2	0.7852	1.5603	0.1187
	Weed growth response	124	11	-0.0450	-0.1082	0.9139
	Weed diversity response	14	1	-1.2098	-3.5399	0.0004
	Plant-feeding nematode abundance response	29	6	-3.2725	-1.2976	0.1944
	Plant disease spread response	539	65	-1.3258	-3.5128	0.0004
	Plant disease damage response	400	78	-1.2228	-5.0365	4.7422×10^{-7}
	Plant growth response	624	100	0.3188	5.1484	2.6277×10^{-7}
	Plant quality response	273	36	0.0539	1.2935	0.1958
	Plant reproduction response	1619	217	0.4678	6.8927	5.4760×10^{-12}
Tropical zone	Total plant antagonist performance	140	32	-0.9666	-4.4638	8.0519×10^{-6}
	Total invertebrate herbivore performance	58	20	-0.8655	-3.4824	0.0005
	Total performance of herbivore natural enemies	19	11	0.2060	0.3883	0.6978
	Total weed performance	5	2	-0.2895	-0.7449	0.4563
	Total plant-feeding nematode performance	6	1	-0.3404	-2.0777	0.0377
	Total plant disease performance	71	14	-1.0636	-2.2319	0.0256
	Total plant performance	189	31	0.4464	4.8670	1.1330×10^{-6}
	Plant antagonist intensity response	135	32	-1.0564	-4.7490	2.0443×10^{-6}
	Plant antagonist diversity response	5	2	0.0231	0.0654	0.9478
	Herbivore abundance response	41	16	-0.6667	-2.7078	0.0068
	Herbivore damage response	16	8	-2.1676	-1.6825	0.0925
	Herbivore diversity response	N/A	N/A	N/A	N/A	N/A
	Predator abundance response	9	6	1.5005	1.4850	0.1376
	Predator diversity response	N/A	N/A	N/A	N/A	N/A
	Parasitoid abundance response	2	2	0.1341	0.2518	0.8012
	Parasitoid diversity response	N/A	N/A	N/A	N/A	N/A
	Parasitism response	7	4	-0.6658	-2.5542	0.0106
	Weed growth response	N/A	N/A	N/A	N/A	N/A
	Weed diversity response	4	1	-0.2367	-0.5414	0.5883
	Plant-feeding nematode abundance response	6	1	-0.3404	-2.0777	0.0377
	Plant disease spread response	35	8	-1.0297	-1.2901	0.1970
	Plant disease damage response	36	8	-1.5739	-2.6338	0.0084
	Plant growth response	32	9	0.3537	2.5915	0.0096
	Plant quality response	25	7	0.3990	2.0559	0.0398
	Plant reproduction response	132	27	0.5200	4.2433	2.2025×10^{-5}

Supplementary Table 8 Results of the meta-regression analysis for natural enemy performance (predator abundance, predator diversity, parasitoid abundance, parasitoid diversity and parasitism), herbivore performance (abundance, damage and diversity of herbivores), weed performance (growth and diversity of weeds), plant-feeding nematode performance (nematode abundance), plant disease performance (disease spread and damage) and plant performance (growth, quality and reproduction of plants) and their response categories in path analysis (as presented also in Fig. 3 and Supplementary Fig. 18). In addition to the estimated mean effect sizes, test-statistics (t-value), and the associated P-values, the number of studies and observations available for each predictor category are also presented. Each test is two-sided and the original P value is reported with no multiple comparisons.

Trophic interaction	Category	Number of observations	Number of Studies	Effect size	t-value	P-value
Natural enemy→ invertebrate herbivore→ plant	Natural enemy performance (Natural enemy performance vs. invertebrate herbivore performance vs. Plant performance)	91	13	0.7088	1.2052	0.2281
	Invertebrate herbivore performance (Natural enemy performance vs. invertebrate herbivore performance vs. Plant performance)	91	13	-0.8646	-2.4039	0.0162
	Plant performance (Natural enemy performance vs. invertebrate herbivore performance vs. Plant performance)	91	13	0.8835	2.5846	0.0097
Herbivore→ plant	Invertebrate performance (Invertebrate herbivore performance vs. Plant performance)	295	44	-0.6091	-3.6221	0.0003
	Plant performance (Invertebrate herbivore performance vs. Plant performance)	295	44	0.4090	5.9164	3.2906×10^{-9}
Weed→ plant	Weed performance (Weed performance vs. Plant performance)	218	10	-0.6496	-3.4897	0.0005
	Plant performance (Weed performance vs. Plant performance)	218	10	0.3016	1.7848	0.0743
Plant-feeding nematode→ plant	Plant-feeding nematode performance (Plant-feeding nematode performance vs. Plant performance)	28	5	-0.9865	-5.3417	9.2103×10^{-8}
	Plant performance (Plant-feeding nematode performance vs. Plant performance)	28	5	0.2852	2.1792	0.0293
Plant disease→ plant	Plant disease performance (Plant disease performance vs. Plant performance)	969	91	-0.9959	-4.4165	1.0033×10^{-5}
	Plant performance (Plant disease performance vs. Plant performance)	969	91	0.3550	2.4493	0.0143

Supplementary Table 9 Results of the path analyses for the effects of plant genetic diversity or number of added genotypes on the tri-trophic interactions of natural enemy performance of invertebrate herbivores, invertebrate herbivore performance and plant performance, and on the bi-trophic interactions of invertebrate herbivore and plant performances, weed and plant performances, plant-feeding nematode and plant performances, and plant disease and plant performances in all analyzed ecosystems (as presented also in Fig. 3 and Supplementary Figs. 15, 18). The predictor and response columns specify the trophic group pairs and the moderator category. The estimate represents the strength of the relationship. The std. err. of estimate denotes the standardized error of the estimate coefficients for the fitted path-analytic models. The number of studies and observations for the predictor-response pair are also presented. Each test is two-sided and the original P value is reported with no multiple comparisons.

Trophic interaction	Predictor	Response	Number of observations	Number of Studies	Estimate	Std. Err. of Estimate	P-value
Plant genetic diversity on tri-trophic interactions of natural enemies, herbivores and plants	Plant genetic diversity	Enemy performance	91	13	0.7088	0.5881	0.2281
	Enemy performance	Herbivore performance	91	13	-0.0114	0.0958	0.9055
	Plant genetic diversity	Herbivore performance	91	13	-0.8646	0.3597	0.0162
	Herbivore performance	Plant performance	91	13	-0.0247	0.0536	0.6465
	Plant genetic diversity	Plant performance	91	13	0.8835	0.3418	0.0097
Number of added genotypes on tri-trophic interactions of natural enemies, herbivores and plants	Number of added genotypes	Enemy performance	91	13	0.0690	0.1550	0.6573
	Enemy performance	Herbivore performance	91	13	-0.0115	0.0960	0.9050
	Number of added genotypes	Herbivore performance	91	13	0.0347	0.3341	0.9175
	Herbivore performance	Plant performance	91	13	-0.0369	0.0559	0.5107
	Number of added genotypes	Plant performance	91	13	0.1056	0.1362	0.4404
Plant genetic diversity on bi-trophic interactions of herbivores and plants	Plant genetic diversity	Herbivore performance	295	44	-0.6091	0.1682	0.0003
	Herbivore performance	Plant performance	295	44	-0.0267	0.0261	0.3085
	Plant genetic diversity	Plant performance	295	44	0.4090	0.0691	3.2906×10^{-9}
Number of added genotypes on bi-trophic interactions of herbivores and plants	Number of added genotypes	Herbivore performance	295	44	0.3305	0.0953	0.0006
	Herbivore performance	Plant performance	295	44	-0.0414	0.0265	0.1198
	Number of added genotypes	Plant performance	295	44	0.2291	0.0840	0.0069
Plant genetic diversity on bi-trophic interactions of weeds and plants	Plant genetic diversity	Weed performance	218	10	-0.6496	0.1862	0.0005
	Weed performance	Plant performance	218	10	-0.0074	0.0125	0.5518
	Plant genetic diversity	Plant performance	218	10	0.3016	0.1690	0.0743
Number of added genotypes on bi-trophic interactions of weeds and plants	Number of added genotypes	Weed performance	218	10	0.0188	0.1126	0.8676
	Weed performance	Plant performance	218	10	-0.0074	0.0125	0.5569
	Number of added genotypes	Plant performance	218	10	-0.0018	0.0958	0.9851
Plant genetic diversity on bi-trophic interactions of nematodes and plants	Plant genetic diversity	Nematode performance	28	5	-0.9865	0.1847	9.2103×10^{-8}
	Nematode performance	Plant performance	28	5	-0.0393	0.0692	0.5759
	Plant genetic diversity	Plant performance	28	5	0.2852	0.1309	0.0293
Number of added genotypes on bi-trophic interactions of nematodes and plants	Number of added genotypes	Nematode performance	NA	NA	NA	NA	NA
	Nematode performance	Plant performance	NA	NA	NA	NA	NA
	Number of added genotypes	Plant performance	NA	NA	NA	NA	NA
Plant genetic diversity on bi-trophic interactions of diseases and plants	Plant genetic diversity	Disease performance	969	91	-0.9959	0.2255	1.0033×10^{-5}
	Disease performance	Plant performance	969	91	-0.0321	0.0085	0.0002
	Plant genetic diversity	Plant performance	969	91	0.3550	0.1449	0.0143
Number of added genotypes on bi-trophic interactions of diseases and plants	Number of added genotypes	Disease performance	969	91	-0.2943	0.0767	0.0001
	Disease performance	Plant performance	969	91	-0.0323	0.0085	0.0001
	Number of added genotypes	Plant performance	969	91	-0.0245	0.0527	0.6424

Supplementary Table 10 Results of the meta-regression analysis for plant antagonist performance and plant performance (growth, quality and reproduction of plants) and their response categories in path analysis in global ecosystems, agroecosystems, grasslands, forests, old-field ecosystems, marine ecosystems, wetlands and shrublands (as presented also in Supplementary Figs. 16, 19). The plant antagonist performance includes herbivore performance (abundance, damage and diversity of herbivores), weed performance (growth and diversity of weeds), plant-feeding nematode performance (nematode abundance), and plant disease performance (disease spread and damage). In addition to the estimated mean effect sizes, test-statistics (t-value), and the associated P-values, the number of studies and observations available for each predictor category are also presented. Each test is two-sided and the original P value is reported with no multiple comparisons.

Ecosystem type	Category	Number of observations	Number of Studies	Effect size	t-value	P-value
Global ecosystem	Plant antagonist performance (Plant antagonist performance vs. Plant performance)	1484	139	-0.6407	-4.7446	2.0896×10^{-6}
	Plant performance (plant antagonist performance vs. Plant performance)	1484	139	0.3952	6.6793	2.4006×10^{-11}
Agroecosystem	Plant antagonist performance (plant antagonist performance vs. Plant performance)	1376	123	-0.8515	-4.6563	3.2193×10^{-6}
	Plant performance (plant antagonist performance vs. Plant performance)	1376	123	0.3942	4.6830	2.8266×10^{-6}
Grassland	Plant antagonist performance (plant antagonist performance vs. Plant performance)	32	3	-0.7854	-3.7081	0.0002
	Plant performance (plant antagonist performance vs. Plant performance)	32	3	0.6824	4.6889	2.7467×10^{-6}
Forest	Plant antagonist performance (plant antagonist performance vs. Plant performance)	48	3	-0.0642	-0.2280	0.8196
	Plant performance (plant antagonist performance vs. Plant performance)	48	3	0.1136	0.4935	0.6216
Old-field ecosystem	Plant antagonist performance (plant antagonist performance vs. Plant performance)	10	6	0.1953	0.1129	0.9101
	Plant performance (plant antagonist performance vs. Plant performance)	10	6	-0.0763	-0.1184	0.9058
Marine ecosystem	Plant antagonist performance (plant antagonist performance vs. Plant performance)	12	3	-0.3358	-1.3292	0.1838
	Plant performance (plant antagonist performance vs. Plant performance)	12	3	0.7067	2.0257	0.0428
Wetland	Plant antagonist performance (plant antagonist performance vs. Plant performance)	NA	NA	N/A	N/A	N/A
	Plant performance (plant antagonist performance vs. Plant performance)	NA	NA	N/A	N/A	N/A
Shrubland	Plant antagonist performance (plant antagonist performance vs. Plant performance)	6	1	-0.0768	-0.2238	0.8229
	Plant performance (plant antagonist performance vs. Plant performance)	6	1	0.5073	1.4176	0.1563

Supplementary Table 11 Results of the path analyses for the effects of plant genetic diversity or number of added genotypes on the bi-trophic interactions of plant antagonist performance and plant performance in global ecosystems, agroecosystems, grasslands, forests, old-field ecosystems, marine ecosystems, wetlands and in shrublands (as presented also in Supplementary Figs. 16, 19). The predictor and response columns specify the trophic group pairs and the moderator category. The estimate represents the strength of the relationship. The std. err. of estimate denotes the standardized error of the estimate coefficients for the fitted path-analytic models. The number of studies and observations for the predictor-response pair are also presented. Each test is two-sided and the original P value is reported with no multiple comparisons.

Ecosystem type	Trophic interaction	Predictor	Response	Number of observations	Number of Studies	Estimate	Std. Err. of Estimate	P -value
Global ecosystems	Plant genetic diversity on bi-trophic interactions of plant antagonists and plants	Plant genetic diversity	Plant antagonist performance	1483	139	-0.6407	0.1350	2.0896×10^{-6}
		Plant antagonist performance	Plant performance	1483	139	-0.0225	0.0068	0.0009
		Plant genetic diversity	Plant performance	1483	139	0.3951	0.0592	2.3963×10^{-11}
	Number of added genotypes on bi-trophic interactions of plant antagonists and plants	Number of added genotypes	Plant antagonist performance	1483	139	-0.0912	0.0580	0.1163
		Plant antagonist performance	Plant performance	1483	139	-0.0229	0.0068	0.0007
		Number of added genotypes	Plant performance	1483	139	0.0481	0.0405	0.2353
Agroecosystem	Plant genetic diversity on bi-trophic interactions of plant antagonists and plants	Plant genetic diversity	Plant antagonist performance	1375	123	-0.8515	0.1829	3.2193×10^{-6}
		Plant antagonist performance	Plant performance	1375	123	-0.0223	0.0068	0.0012
		Plant genetic diversity	Plant performance	1375	123	0.3942	0.0842	2.8266×10^{-6}
	Number of added genotypes on bi-trophic interactions of plant antagonists and plants	Number of added genotypes	Plant antagonist performance	1375	123	-0.1072	0.0610	0.0791
		Plant antagonist performance	Plant performance	1375	123	-0.0224	0.0069	0.0011
		Number of added genotypes	Plant performance	1375	123	0.0506	0.0434	0.2440
Grassland	Plant genetic diversity on bi-trophic interactions of plant antagonists and plants	Plant genetic diversity	Plant antagonist performance	32	3	-0.7854	0.2118	0.0002
		Plant antagonist performance	Plant performance	32	3	-0.1126	0.0694	0.1158
		Plant genetic diversity	Plant performance	32	3	0.6824	0.1455	2.7467×10^{-6}
	Number of added genotypes on bi-trophic interactions of plant antagonists and plants	Number of added genotypes	Plant antagonist performance	32	3	-0.4913	0.3560	0.1785
		Plant antagonist performance	Plant performance	32	3	-0.1287	0.0720	0.0852
		Number of added genotypes	Plant performance	32	3	0.2157	0.2595	0.4132
Forest	Plant genetic diversity on bi-trophic interactions of plant antagonists and plants	Plant genetic diversity	Plant antagonist performance	48	3	-0.0642	0.2816	0.8196
		Plant antagonist performance	Plant performance	48	3	0.1474	0.1600	0.3623
		Plant genetic diversity	Plant performance	48	3	0.1136	0.2302	0.6216
	Number of added genotypes on bi-trophic interactions of plant antagonists and plants	Number of added genotypes	Plant antagonist performance	48	3	0.0042	0.2514	0.9868
		Plant antagonist performance	Plant performance	48	3	0.1590	0.1721	0.3611
		Number of added genotypes	Plant performance	48	3	-0.6412	0.3065	0.0428
Old-field ecosystem	Plant genetic diversity on bi-trophic interactions of plant antagonists and plants	Plant genetic diversity	plant antagonist performance	10	6	0.1953	1.7297	0.9101
		Plant antagonist performance	Plant performance	10	6	0.1927	0.1849	0.3738
		Plant genetic diversity	Plant performance	10	6	-0.0763	0.6450	0.9058
	Number of added genotypes on bi-trophic interactions of plant antagonists and plants	Number of added genotypes	Plant antagonist performance	10	6	0.0078	1.4295	0.9959
		Plant antagonist performance	Plant performance	10	6	0.2149	0.1593	0.2700
		Number of added genotypes	Plant performance	10	6	1.1513	0.6113	0.1328
Marine ecosystem	Plant genetic diversity on bi-trophic interactions of plant antagonists and plants	Plant genetic diversity	Plant antagonist performance	12	3	-0.3358	0.2526	0.1838
		Plant antagonist performance	Plant performance	12	3	0.4138	0.3205	0.2327
		Plant genetic diversity	Plant performance	12	3	0.7067	0.3489	0.0428
	Number of added genotypes on bi-trophic interactions of plant antagonists and plants	Number of added genotypes	Plant antagonist performance	12	3	0.1627	0.4280	0.7138
		Plant antagonist performance	Plant performance	12	3	0.4519	0.3110	0.1895
		Number of added genotypes	Plant performance	12	3	0.3677	0.4635	0.4537
Wetland	Plant genetic diversity on bi-trophic interactions of plant antagonists and plants	Plant genetic diversity	Plant antagonist performance	NA	NA	NA	NA	NA
		Plant antagonist performance	Plant performance	NA	NA	NA	NA	NA
		Plant genetic diversity	Plant performance	NA	NA	NA	NA	NA
	Number of added genotypes on bi-trophic interactions of plant antagonists and plants	Number of added genotypes	Plant antagonist performance	NA	NA	NA	NA	NA
		Plant antagonist performance	Plant performance	NA	NA	NA	NA	NA
		Number of added genotypes	Plant performance	NA	NA	NA	NA	NA
Shrubland	Plant genetic diversity on bi-trophic interactions of plant antagonists and plants	Plant genetic diversity	Plant antagonist performance	6	1	-0.0768	0.3433	0.8229
		Plant antagonist performance	Plant performance	6	1	-1.0149	0.8168	0.2819
		Plant genetic diversity	Plant performance	6	1	0.5073	0.3578	0.1563
	Number of added genotypes on bi-trophic interactions of plant antagonists and plants	Number of added genotypes	Plant antagonist performance	NA	NA	NA	NA	NA
		Plant antagonist performance	Plant performance	NA	NA	NA	NA	NA
		Number of added genotypes	Plant performance	NA	NA	NA	NA	NA

Supplementary Table 12 Results of the meta-regression analysis for plant antagonist performance and plant performance (growth, quality and reproduction of plants) and their response categories in path analysis in plot experiments, pot experiments, herbaceous plants, woody plants, temperate zones, and tropical zones (as presented also in Supplementary Figs. 17, 20). The plant antagonist performance including herbivore performance (abundance, damage and diversity of herbivores), weed performance (growth and diversity of weeds), plant-feeding nematode performance (nematode abundance), and plant disease performance (disease spread and damage). In addition to the estimated mean effect sizes, test-statistics (t-value), and the associated P-values, the number of studies and observations available for each predictor category are also presented. Data from greenhouse and other indoor experiments were removed from the models with climatic predictors. Each test is two-sided and the original P value is reported with no multiple comparisons.

Item	Category	Number of observations	Number of Studies	Effect size	t-value	P-value
Plot experiment	Plant antagonist performance (Plant antagonist performance vs. Plant performance)	1386	129	-0.7180	-4.8345	1.3346×10^{-6}
	Plant performance (Plant antagonist performance vs. Plant performance)	1386	129	0.4138	5.8291	5.5719×10^{-9}
Pot experiment	Plant antagonist performance (Plant antagonist performance vs. Plant performance)	98	11	-0.7128	-1.0299	0.3030
	Plant performance (Plant antagonist performance vs. Plant performance)	98	11	0.3232	1.5609	0.1185
Herbaceous plant	Plant antagonist performance (Plant antagonist performance vs. Plant performance)	1411	131	-0.7966	-5.0875	3.6285×10^{-7}
	Plant performance (Plant antagonist performance vs. Plant performance)	1411	131	0.4313	6.2918	3.1377×10^{-10}
Woody plant	Plant antagonist performance (plant antagonist performance vs. Plant performance)	73	8	-0.0559	-0.1456	0.8842
	Plant performance (Plant antagonist performance vs. Plant performance)	73	8	0.2631	2.0980	0.0359
Temperate zone	Plant antagonist performance (Plant antagonist performance vs. Plant performance)	1349	116	-0.5548	-3.9983	6.3789×10^{-5}
	Plant performance (Plant antagonist performance vs. Plant performance)	1349	116	0.3480	5.0770	3.8342×10^{-7}
Tropical zone	Plant antagonist performance (Plant antagonist performance vs. Plant performance)	111	17	-1.4460	-2.5612	0.0104
	Plant performance (Plant antagonist performance vs. Plant performance)	111	17	0.5385	4.1108	3.9429×10^{-5}

Supplementary Table 13 Results of the path analyses for the effects of plant genetic diversity or number of added genotypes on the bi-trophic interactions of plant antagonist performance and plant performance in plot experiments, pot experiments, herbaceous plants, woody plants, temperate zones, and tropical zones (as presented also in Supplementary Figs. 17 and 20). The predictor and response columns specify the trophic group pairs and the moderator category. The estimate represents the strength of the relationship. The std. err. of estimate denotes the standard error of the estimate coefficients for the fitted path-analytic models. The number of studies and observations for the predictor-response pair are also presented. Data from greenhouse and other indoor experiments were removed from the models with climatic predictors. Each test is two-sided and the original P value is reported with no multiple comparisons.

Category	Trophic interaction	Predictor	Response	Number of observations	Number of Studies	Estimate	Std. Err. of Estimate	P-value
Plot experiment	Plant genetic diversity on bi-trophic interactions of plant antagonists and plants	Plant genetic diversity	Plant antagonist performance	1385	129	-0.7180	0.1485	1.3346×10^{-6}
		Plant antagonist performance	Plant performance	1385	129	-0.0221	0.0072	0.0021
		Plant genetic diversity	Plant performance	1385	129	0.4139	0.0710	5.6113×10^{-9}
	Number of added genotypes on bi-trophic interactions of plant antagonists and plants	Number of added genotypes	Plant antagonist performance	1385	129	-0.1049	0.0590	0.0757
		Plant antagonist performance	Plant performance	1385	129	-0.0224	0.0072	0.0018
		Number of added genotypes	Plant performance	1385	129	0.0433	0.0418	0.3007
Pot experiment	Plant genetic diversity on bi-trophic interactions of plant antagonists and plants	Plant genetic diversity	Plant antagonist performance	98	11	-0.7128	0.6921	0.3030
		Plant antagonist performance	Plant performance	98	11	-0.0190	0.0209	0.3667
		Plant genetic diversity	Plant performance	98	11	0.3232	0.2070	0.1185
	Number of added genotypes on bi-trophic interactions of plant antagonists and plants	Number of added genotypes	Plant antagonist performance	98	11	0.3369	0.2673	0.2109
		Plant antagonist performance	Plant performance	98	11	-0.0213	0.0211	0.3154
		Number of added genotypes	Plant performance	98	11	0.1412	0.1588	0.3765
Herbaceous plant	Plant genetic diversity on bi-trophic interactions of plant antagonists and plants	Plant genetic diversity	Plant antagonist performance	1410	131	-0.7966	0.1566	3.6285×10^{-7}
		Plant antagonist performance	Plant performance	1410	131	-0.0221	0.0068	0.0013
		Plant genetic diversity	Plant performance	1410	131	0.4312	0.0685	3.1355×10^{-10}
	Number of added genotypes on bi-trophic interactions of plant antagonists and plants	Number of added genotypes	Plant antagonist performance	1410	131	-0.0942	0.0591	0.1109
		Plant antagonist performance	Plant performance	1410	131	-0.0226	0.0069	0.0010
		Number of added genotypes	Plant performance	1410	131	0.0655	0.0415	0.1147
Woody plant	Plant genetic diversity on bi-trophic interactions of plant antagonists and plants	Plant genetic diversity	Plant antagonist performance	73	8	-0.0559	0.3842	0.8842
		Plant antagonist performance	Plant performance	73	8	-0.0231	0.0527	0.6633
		Plant genetic diversity	Plant performance	73	8	0.2631	0.1254	0.0359
	Number of added genotypes on bi-trophic interactions of plant antagonists and plants	Number of added genotypes	Plant antagonist performance	73	8	0.2035	0.2389	0.3975
		Plant antagonist performance	Plant performance	73	8	0.0182	0.0594	0.7604
		Number of added genotypes	Plant performance	73	8	-0.3995	0.2026	0.0533
Temperate climatic zone	Plant genetic diversity on bi-trophic interactions of plant antagonists and plants	Plant genetic diversity	Plant antagonist performance	1348	116	-0.5548	0.1388	6.3789×10^{-5}
		Plant antagonist performance	Plant performance	1348	116	-0.0197	0.0069	0.0042
		Plant genetic diversity	Plant performance	1348	116	0.3479	0.0685	3.8330×10^{-7}
	Number of added genotypes on bi-trophic interactions of plant antagonists and plants	Number of added genotypes	Plant antagonist performance	1348	116	-0.0941	0.0600	0.1167
		Plant antagonist performance	Plant performance	1348	116	-0.0202	0.0069	0.0034
		Number of added genotypes	Plant performance	1348	116	0.0540	0.0416	0.1949
Tropical climatic zone	Plant genetic diversity on bi-trophic interactions of plant antagonists and plants	Plant genetic diversity	Plant antagonist performance	111	17	-1.4460	0.5646	0.0104
		Plant antagonist performance	Plant performance	111	17	-0.1025	0.0334	0.0028
		Plant genetic diversity	Plant performance	111	17	0.5385	0.1310	3.9429×10^{-5}
	Number of added genotypes on bi-trophic interactions of plant antagonists and plants	Number of added genotypes	Plant antagonist performance	111	17	-0.0260	0.2492	0.9172
		Plant antagonist performance	Plant performance	111	17	-0.1027	0.0337	0.0030
		Number of added genotypes	Plant performance	111	17	0.0292	0.1566	0.8523

Supplementary Table 14 Sensitivity analysis for trophic groups of plant antagonist performance, herbivore performance, natural enemy performance, weed performance, nematode performance, disease performance and plant performance. Each test is two-sided and the original P value is reported with no multiple comparisons.

Trophic group	Number of missing studies	Effect size	t-value	P-value
Plant antagonist performance	470	-0.7665	-11.6815	1.59×10^{-31}
Invertebrate herbivore performance	114	-0.2919	-3.7344	0.0002
Performance of natural enemies of herbivores	32	0.5225	3.6509	0.0003
Weed performance	42	-0.4436	-2.7444	0.0061
Plant-feeding nematode performance	10	-1.3420	-3.0737	0.0021
Plant disease performance	303	-1.1122	-11.7033	1.23×10^{-31}
Plant performance	124	0.2401	17.7729	1.15×10^{-70}

Supplementary Table 15 Extra description for analyses of plant genetic diversity and number of added genotypes.

Supplementary Table 15a: Plant genetic diversity.

1) an experimental activity of conducting plant genetic diversity planting; 2) does not consider the number of added plant genotypes (i.e., all the diversity treatments with ≥ 2 genotypes were put into only one group in data analysis); 3) compared with plant genetic diversity, the pure / mono- genotypic planting was considered as the control. Namely, plant genetic diversity denotes the experimental behavior or activity in the treatment with more genotypes (i.e., ≥2 genotypes), and we confirmed that both the control and treatment were compared to a single plant species; and 4) the treatments with different genetic diversities (i.e., ≥2 genotypes) were classified into the same one group in path analysis for Figs. 2 and 3 and for Supplementary Figs. 18-20. The term "plant genetic diversity" is analyzed using the "rma.mv()" function in R package "metafor", employing a mixed effect model in the meta-analysis, where "plant species" was included as random effect with phylogenetic relatedness as part of the correlation structure. The descriptions of relevant parameters can be found in the documentation of the R packages "metafor" and "nlme". Each test is two-sided and the original P value is reported with no multiple comparisons.

Item	Plant genetic diversity							
	AIC	AICc	BIC	logLik	sigma2	tau2	QE	QEp
Tri-trophic interactions of invertebrate herbivore, natural enemy and plant performances (Fig. 3)	273.4121	273.6880	280.9115	-133.7061	0.0069	0.0000	161.5724	5.5249×10^{-6}
Bi-trophic interactions of invertebrate herbivore and plant performances (Supplementary Fig. 18a)	1047.7949	1047.8774	1058.8457	-520.8975	0.5283	0.0000	535.8797	2.4912×10^{-16}
Bi-trophic interactions of weed and plant performances (Supplementary Fig. 18b)	1224.6259	1224.7380	1234.7656	-609.3129	0.0000	0.0000	742.7485	1.0276×10^{-58}
Bi-trophic interactions of plant-feeding nematode and plant performances (Supplementary Fig. 18c)	189.0798	189.2337	190.3756	-93.5399	0.0000	0.0000	121.4871	5.9962×10^{-14}
Bi-trophic interactions of plant disease and plant performances (Supplementary Fig. 18d)	4748.1026	4748.1275	4762.7283	-2371.0513	0.6661	0.0000	2794.2248	1.7748×10^{-176}
Bi-trophic interactions of plant antagonist and plant performances across global ecosystems (Fig. 2)	7313.5845	7313.6007	7329.4880	-3653.7923	0.4632	0.0000	4313.4446	7.0824×10^{-274}
Bi-trophic interactions of plant antagonist and plant performances on agroecosystems (Supplementary Fig. 19a)	7019.3554	7019.3729	7035.0318	-3506.6777	0.5186	0.0000	4128.3458	1.0533×10^{-272}
Bi-trophic interactions of plant antagonist and plant performances on grasslands (Supplementary Fig. 19b)	112.7128	113.5699	117.0148	-53.3564	0.0000	0.0000	44.1570	0.0591
Bi-trophic interactions of plant antagonist and plant performances on forests (Supplementary Fig. 19c)	102.5386	103.0840	108.0890	-48.2693	0.0000	0.0000	30.3185	0.9719
Bi-trophic interactions of plant antagonist and plant performances on old-field ecosystems (Supplementary Fig. 19d)	34.6890	38.6890	35.2806	-14.3445	8.1080	0.0000	25.3746	0.0026
Bi-trophic interactions of plant antagonist and plant performances on marine ecosystems (Supplementary Fig. 19e)	27.5564	30.5564	28.7501	-10.7782	0.0309	0.0000	12.2812	0.3429
Bi-trophic interactions of plant antagonist and plant performances on shrublands (Supplementary Fig. 19f)	11.0170	12.0170	10.6264	-4.5085	0.0000	0.0000	1.5519	0.9070
Bi-trophic interactions of plant antagonist and plant performances in plot experiments (Supplementary Fig. 20a)	6723.2261	6723.2435	6738.9243	-3358.6130	0.5273	0.0000	3949.1853	1.0149×10^{-244}
Bi-trophic interactions of plant antagonist and plant performances in pot experiments (Supplementary Fig. 20b)	536.2075	536.4628	543.9316	-265.1038	2.1564	0.0000	357.0774	1.9949×10^{-31}
Bi-trophic interactions of plant antagonist and plant performances for herbaceous plants (Supplementary Fig. 20c)	7098.2275	7098.2445	7113.9794	-3546.1137	0.4816	0.0000	4178.4489	1.2651×10^{-271}
Bi-trophic interactions of plant antagonist and plant performances for woody plants (Supplementary Fig. 20d)	211.7418	212.0896	218.5718	-102.8709	0.0000	0.0000	95.7484	0.0322
Bi-trophic interactions of plant antagonist and plant performances in temperate zones (Supplementary Fig. 20e)	6587.3236	6587.3414	6602.9405	-3290.6618	0.3745	0.0000	3794.9732	2.1090×10^{-231}
Bi-trophic interactions of plant antagonist and plant performances in tropical zones (Supplementary Fig. 20f)	565.6424	565.8667	573.7438	-279.8212	0.0999	0.0000	434.4654	4.0826×10^{-40}

Supplementary Table 15b: Number of added genotypes.

When analyzing the effects of the number of number of added genotypes on bi-or-tri-trophic interactions, we put the treatments with different genetic diversities (i.e., ≥ 2 genotypes) into different subgroups (Supplementary Figs. 15-17, respectively). Each test is two-sided and the original P value is reported with no multiple comparisons.

Item	Number of added genotypes			
	AIC	AICc	BIC	logLik
Tri-trophic interactions of invertebrate herbivore, natural enemy and plant performances (Supplementary Fig. 15a)	282.6064	283.0715	292.5610	-137.3032
bi-trophic interactions of invertebrate herbivore and plant performances (Supplementary Fig. 15b)	1021.0363	1021.1742	1035.7570	-506.5182
Bi-trophic interactions of weed and plant performances (Supplementary Fig. 15c)	1208.8094	1208.9971	1222.3105	-600.4047
Bi-trophic interactions of plant disease and plant performances (Supplementary Fig. 15d)	4092.4554	4092.4969	4111.9522	-2042.2277
Bi-trophic interactions of plant antagonist and plant performances across global ecosystems (Supplementary Fig. 16a)	6390.7200	6390.7470	6411.9219	-3191.3600
Bi-trophic interactions of plant antagonist and plant performances on agroecosystems (Supplementary Fig. 16b)	6074.9417	6074.9709	6095.8407	-3033.4709
Bi-trophic interactions of plant antagonist and plant performances on grasslands (Supplementary Fig. 16c)	114.3942	115.8757	119.9990	-53.1971
Bi-trophic interactions of plant antagonist and plant performances on forests (Supplementary Fig. 16d)	106.5048	107.4351	113.8194	-49.2524
Bi-trophic interactions of plant antagonist and plant performances on old-field ecosystems (Supplementary Fig. 16e)	37.6819	45.6819	37.9996	-14.8409
Bi-trophic interactions of plant antagonist and plant performances on marine ecosystems (Supplementary Fig. 16f)	27.6448	33.3591	28.8551	-9.8224
Bi-trophic interactions of plant antagonist and plant performances in plot experiments (Supplementary Fig. 17a)	5842.7913	5842.8203	5863.7193	-2917.3957
Bi-trophic interactions of plant antagonist and plant performances in pot experiments (Supplementary Fig. 17b)	553.4513	553.8814	563.7087	-272.7256
Bi-trophic interactions of plant antagonist and plant performances for herbaceous plants (Supplementary Fig. 17c)	6174.3911	6174.4196	6195.3908	-3083.1956
Bi-trophic interactions of plant antagonist and plant performances for woody plants (Supplementary Fig. 17d)	210.7795	211.3677	219.8302	-101.3897
Bi-trophic interactions of plant antagonist and plant performances in temperate zones (Supplementary Fig. 17e)	5884.8955	5884.9252	5905.7150	-2938.4477
Bi-trophic interactions of plant antagonist and plant performances in tropical zones (Supplementary Fig. 17f)	422.3105	422.6879	433.0759	-207.1552

Supplementary Table 16 Statistic values for the relationship between number of added genotypes in the plant genetic diversity treatment over the control and the different effect sizes along with fitted meta-regression lines. Note that a value of zero on the x axis indicates that only one genotype was added (log scale). Each test is two-sided and the original P value is reported with no multiple comparisons.

Figure source	Effect size of trophic response category (Y)	Number of added genotypes over the control (X)	Number of observations	Number of Studies	d.f.	Regression equation	95%CL	95%CU	Std. Error	t-value	P-value
Supplementary Fig. 1a	Plant antagonist performance	Number of added genotypes over the control across all studies	1736	236	1734	Y = 0.581X - 2.796	0.4096	0.7514	0.0872	6.6570	3.74×10^{-11}
Supplementary Fig. 1b	Herbivore performance	Number of added genotypes over the control across all studies	468	93	466	Y = 0.536X - 1.828	0.3128	0.7596	0.1140	4.7040	3.36×10^{-6}
Supplementary Fig. 1c	Natural enemy performance	Number of added genotypes over the control across all studies	104	33	102	Y = -0.144X + 1.997	-0.4914	0.2035	0.1773	-0.8118	0.4188
Supplementary Fig. 1d	Weed performance	Number of added genotypes over the control across all studies	200	14	198	Y = 0.632X - 1.728	0.3990	0.8651	0.1189	5.3160	2.85×10^{-7}
Supplementary Fig. 1e	Nematode performance	Number of added genotypes over the control across all studies	35	7	33	Y = -2.05X - 2.806	-5.5210	1.4220	1.7710	-1.1570	0.2555
Supplementary Fig. 1f	Plant disease performance	Number of added genotypes over the control across all studies	1033	136	1031	Y = 0.073X - 3.101	-0.2225	0.3676	0.1505	0.4819	0.6300
Supplementary Fig. 1g	Plant performance	Number of added genotypes over the control across all studies	2862	320	2860	Y = 0.075X + 0.407	0.0185	0.1318	0.0289	2.5990	0.0094
Supplementary Fig. 2a	Plant antagonist performance	Number of added genotypes over the control in agroecosystems	1536	195	1534	Y = 0.346X - 2.901	0.1507	0.5415	0.0997	3.4720	0.0005
Supplementary Fig. 2b	Herbivore performance	Number of added genotypes over the control in agroecosystems	310	57	308	Y = 0.306X - 2.042	-0.0485	0.6611	0.1810	1.6920	0.0916
Supplementary Fig. 2c	Natural enemy performance	Number of added genotypes over the control in agroecosystems	52	17	50	Y = -0.25X + 2.763	-1.0590	0.5586	0.4128	-0.6067	0.5468
Supplementary Fig. 2d	Weed performance	Number of added genotypes over the control in agroecosystems	178	12	176	Y = 0.585X - 1.813	0.3866	0.7833	0.1012	5.7810	3.32×10^{-8}
Supplementary Fig. 2e	Nematode performance	Number of added genotypes over the control in agroecosystems	35	7	33	Y = -2.05X - 2.806	-5.5210	1.4220	1.7710	-1.1570	0.2555
Supplementary Fig. 2f	Plant disease performance	Number of added genotypes over the control in agroecosystems	1013	131	1011	Y = 0.09X - 3.142	-0.2095	0.3893	0.1528	0.5885	0.5563
Supplementary Fig. 2g	Plant performance	Number of added genotypes over the control in agroecosystems	2538	267	2536	Y = 0.026X + 0.427	-0.0383	0.0897	0.0327	0.7872	0.4313
Supplementary Fig. 3a	Plant antagonist performance	Number of added genotypes over the control in grasslands	55	9	53	Y = 0.962X - 2.323	0.0924	1.8320	0.4437	2.1680	0.0346
Supplementary Fig. 3b	Herbivore performance	Number of added genotypes over the control in grasslands	25	5	23	Y = 0.662X - 2.7	-0.9601	2.2840	0.8276	0.7999	0.4320
Supplementary Fig. 3c	Natural enemy performance	Number of added genotypes over the control in grasslands	20	3	18	Y = -1.152X + 1.91	-2.7970	0.4938	0.8395	-1.3720	0.1870
Supplementary Fig. 3d	Weed performance	Number of added genotypes over the control in grasslands	22	2	20	Y = 1.344X - 2.115	0.2136	2.4750	0.5770	2.3300	0.0304
Supplementary Fig. 3e	Plant disease performance	Number of added genotypes over the control in grasslands	8	3	6	Y = -0.655X - 1.458	-3.1440	1.8350	1.2700	-0.5155	0.6246
Supplementary Fig. 3f	Plant performance	Number of added genotypes over the control in grasslands	134	17	132	Y = 0.382X + 0.257	0.0891	0.6743	0.1493	2.5570	0.0117

Supplementary Fig. 4a	Plant antagonist performance	Number of added genotypes over the control in forests	89	14	87	Y = -0.399X - 0.443	-0.7831	-0.0156	0.1958	-2.0400	0.0444
Supplementary Fig. 4b	Herbivore performance	Number of added genotypes over the control in forests	79	14	77	Y = -0.536X - 0.314	-0.9252	-0.1466	0.1986	-2.6980	0.0086
Supplementary Fig. 4c	Natural enemy performance	Number of added genotypes over the control in forests	7	5	N/A	N/A	N/A	N/A	N/A	N/A	N/A
Supplementary Fig. 4d	Plant disease performance	Number of added genotypes over the control in forests	10	1	8	Y = 0.383X - 0.746	-0.5801	1.3460	0.4914	0.7794	0.4582
Supplementary Fig. 4e	Plant performance	Number of added genotypes over the control in forests	61	4	59	Y = -0.081X + 0.335	-0.6285	0.4667	0.2794	-0.2896	0.7731
Supplementary Fig. 5a	Plant antagonist performance	Number of added genotypes over the control in old-field ecosystems	20	10	18	Y = 0.426X - 0.339	-0.6530	1.5060	0.5506	0.7742	0.4489
Supplementary Fig. 5b	Herbivore performance	Number of added genotypes over the control in old-field ecosystems	18	9	16	Y = -0.525X + 2.29	-1.3000	0.2495	0.3954	-1.3290	0.2025
Supplementary Fig. 5c	Natural enemy performance	Number of added genotypes over the control in old-field ecosystems	14	4	12	Y = 0.436X + 0.978	-0.0402	0.9123	0.2430	1.7950	0.0979
Supplementary Fig. 5d	Plant disease performance	Number of added genotypes over the control in old-field ecosystems	2	1	N/A	N/A	N/A	N/A	N/A	N/A	N/A
Supplementary Fig. 5e	Plant performance	Number of added genotypes over the control in old-field ecosystems	27	11	25	Y = 0.398X - 0.157	-0.0273	0.8238	0.2171	1.8340	0.0785
Supplementary Fig. 6a	Plant antagonist performance	Number of added genotypes over the control in marine ecosystems	13	5	11	Y = 0.736X - 1.888	-0.5574	2.0290	0.6598	1.1150	0.2885
Supplementary Fig. 6b	Herbivore performance	Number of added genotypes over the control in marine ecosystems	13	5	11	Y = 0.736X - 1.888	-0.5574	2.0290	0.6598	1.1150	0.2885
Supplementary Fig. 6c	Plant performance	Number of added genotypes over the control in marine ecosystems	37	9	35	Y = 0.287X + 0.329	-0.1910	0.7658	0.2441	1.1770	0.2470
Supplementary Fig. 7a	Natural enemy performance	Number of added genotypes over the control in wetlands	3	1	1	Y = 0.012X + 0.235	-0.2896	0.3132	0.1538	0.0768	0.9512
Supplementary Fig. 7b	Plant performance	Number of added genotypes over the control in wetlands	43	9	41	Y = 0.531X - 0.314	0.1944	0.8673	0.1717	3.0920	0.0036
Supplementary Fig. 8a	Plant antagonist performance	Number of added genotypes over the control in shrublands	23	3	21	Y = 0.21X - 0.009	-0.2082	0.6291	0.2136	0.9852	0.3357
Supplementary Fig. 8b	Herbivore performance	Number of added genotypes over the control in shrublands	23	3	21	Y = 0.21X - 0.009	-0.2082	0.6291	0.2136	0.9852	0.3357
Supplementary Fig. 8c	Natural enemy performance	Number of added genotypes over the control in shrublands	8	3	6	Y = 0.716X + 0.197	-0.0413	1.4730	0.3863	1.8530	0.1133
Supplementary Fig. 8d	Plant performance	Number of added genotypes over the control in shrublands	22	3	20	Y = -0.151X + 0.231	-0.3976	0.0948	0.1256	-1.2050	0.2422
Supplementary Fig. 9a	Plant antagonist performance	Number of added genotypes over the control in plot experiments	1582	216	1580	Y = 0.514X - 2.789	0.3229	0.7060	0.0977	5.2650	1.60×10^{-7}
Supplementary Fig. 9b	Herbivore performance	Number of added genotypes over the control in plot experiments	406	78	404	Y = 0.581X - 1.976	0.3079	0.8532	0.1391	4.1730	3.69×10^{-5}
Supplementary Fig. 9c	Natural enemy performance	Number of added genotypes over the control in plot experiments	100	32	98	Y = -0.152X + 2.05	-0.5156	0.2120	0.1856	-0.8177	0.4155
Supplementary Fig. 9d	Weed performance	Number of added genotypes over the control in plot experiments	131	13	129	Y = 0.565X - 1.542	0.2948	0.8346	0.1377	4.1010	7.25×10^{-5}
Supplementary Fig. 9e	Nematode performance	Number of added genotypes over the control in plot experiments	35	7	33	Y = -2.05X - 2.806	-5.5210	1.4220	1.7710	-1.1570	0.2555

Supplementary Fig. 9f	Plant disease performance	Number of added genotypes over the control in plot experiments	1010	132	1008	Y = 0.02X - 2.999	-0.2716	0.3116	0.1488	0.1345	0.8931
Supplementary Fig. 9g	Plant performance	Number of added genotypes over the control in plot experiments	2667	286	2665	Y = 0.063X + 0.409	0.0029	0.1239	0.0309	2.0550	0.03998
Supplementary Fig. 10a	Plant antagonist performance	Number of added genotypes over the control in pot experiments	154	24	152	Y = 0.365X - 1.854	0.0277	0.7030	0.1723	2.1210	0.0356
Supplementary Fig. 10b	Herbivore performance	Number of added genotypes over the control in pot experiments	62	16	60	Y = 0.122X - 0.64	-0.1849	0.4293	0.1567	0.7799	0.4385
Supplementary Fig. 10c	Natural enemy performance	Number of added genotypes over the control in pot experiments	4	1	N/A	N/A	N/A	N/A	N/A	N/A	N/A
Supplementary Fig. 10d	Weed performance	Number of added genotypes over the control in pot experiments	69	3	67	Y = 1.512X - 2.451	0.3337	2.6910	0.6013	2.5150	0.0143
Supplementary Fig. 10e	Plant disease performance	Number of added genotypes over the control in pot experiments	23	5	N/A	N/A	N/A	N/A	N/A	N/A	N/A
Supplementary Fig. 10f	Plant performance	Number of added genotypes over the control in pot experiments	195	34	193	Y = 0.081X + 0.406	-0.0659	0.2269	0.0747	1.0780	0.2826
Supplementary Fig. 11a	Plant antagonist performance	Number of added genotypes over the control for herbaceous plants	1581	207	1579	Y = 0.565X - 2.92	0.3847	0.7461	0.0922	6.1330	1.09×10^{-9}
Supplementary Fig. 11b	Herbivore performance	Number of added genotypes over the control for herbaceous plants	336	65	334	Y = 0.552X - 1.98	0.2855	0.8190	0.1361	4.0570	6.19×10^{-5}
Supplementary Fig. 11c	Natural enemy performance	Number of added genotypes over the control for herbaceous plants	84	23	82	Y = -0.157X + 2.127	-0.5505	0.2370	0.2009	-0.7801	0.4376
Supplementary Fig. 11d	Weed performance	Number of added genotypes over the control for herbaceous plants	200	14	198	Y = 0.632X - 1.728	0.3990	0.8651	0.1189	5.3160	2.85×10^{-7}
Supplementary Fig. 11e	Nematode performance	Number of added genotypes over the control for herbaceous plants	35	7	33	Y = -2.05X - 2.806	-5.5210	1.4220	1.7710	-1.1570	0.2555
Supplementary Fig. 11f	Plant disease performance	Number of added genotypes over the control for herbaceous plants	1010	131	1008	Y = 0.075X - 3.134	-0.2227	0.3719	0.1517	0.4919	0.6229
Supplementary Fig. 11g	Plant performance	Number of added genotypes over the control for herbaceous plants	2741	306	2739	Y = 0.082X + 0.41	0.0242	0.1399	0.0295	2.7790	0.0055
Supplementary Fig. 12a	Plant antagonist performance	Number of added genotypes over the control for woody plants	155	29	153	Y = 0.525X - 1.489	0.1228	0.9276	0.2053	2.5580	0.0115
Supplementary Fig. 12b	Herbivore performance	Number of added genotypes over the control for woody plants	132	28	130	Y = 0.429X - 1.389	-0.0006	0.8577	0.2190	1.9570	0.0525
Supplementary Fig. 12c	Natural enemy performance	Number of added genotypes over the control for woody plants	20	10	18	Y = -1.726X + 3.26	-3.5830	0.1304	0.9473	-1.8220	0.0851
Supplementary Fig. 12d	Plant disease performance	Number of added genotypes over the control for woody plants	23	5	21	Y = 2.367X - 2.13	1.5950	3.1380	0.3937	6.0110	5.77×10^{-6}
Supplementary Fig. 12e	Plant performance	Number of added genotypes over the control for woody plants	121	14	119	Y = -0.126X + 0.368	-0.4007	0.1495	0.1404	-0.8950	0.3726
Supplementary Fig. 13a	Plant antagonist performance	Number of added genotypes over the control for temperate zones	1490	192	1488	Y = 0.53X - 2.789	0.3417	0.7187	0.0962	5.5120	4.17×10^{-8}
Supplementary Fig. 13b	Herbivore performance	Number of added genotypes over the control for temperate zones	384	66	382	Y = 0.517X - 1.747	0.2689	0.7641	0.1263	4.0890	5.29×10^{-5}
Supplementary Fig. 13c	Natural enemy performance	Number of added genotypes over the control for temperate zones	85	23	83	Y = -0.185X + 2.233	-0.5664	0.1961	0.1945	-0.9520	0.3439
Supplementary Fig. 13d	Weed performance	Number of added genotypes over the control for temperate zones	138	11	136	Y = 0.58X - 1.57	0.3113	0.8489	0.1371	4.2300	$4.27\times10^{-}$

Supplementary Fig. 13e	Nematode performance	Number of added genotypes over the control for temperate zones	29	6	27	Y = -1.766X - 3.256	-5.2210	1.6890	1.7630	-1.0020	0.3253
Supplementary Fig. 13f	Plant disease performance	Number of added genotypes over the control for temperate zones	939	119	937	Y = 0.01X - 3.063	-0.2961	0.3157	0.1561	0.0630	0.9498
Supplementary Fig. 13g	Plant performance	Number of added genotypes over the control for temperate zones	2516	266	2514	Y = 0.069X + 0.387	0.0089	0.1292	0.0307	2.2510	0.0245
Supplementary Fig. 14a	Plant antagonist performance	Number of added genotypes over the control for tropical zones	140	32	138	Y = 0.469X - 2.135	-0.1184	1.0570	0.2999	1.5650	0.1198
Supplementary Fig. 14b	Herbivore performance	Number of added genotypes over the control for tropical zones	58	20	56	Y = 0.865X - 2.687	-0.0570	1.7870	0.4704	1.8390	0.0713
Supplementary Fig. 14c	Natural enemy performance	Number of added genotypes over the control for tropical zones	19	11	17	Y = -0.637X + 1.19	-1.8790	0.6046	0.6335	-1.0050	0.3288
Supplementary Fig. 14d	Weed performance	Number of added genotypes over the control for tropical zones	5	2	3	Y = -0.257X - 0.135	-1.2040	0.6901	0.4831	-0.5315	0.6319
Supplementary Fig. 14e	Nematode performance	Number of added genotypes over the control for tropical zones	6	1	N/A	N/A	N/A	N/A	N/A	N/A	N/A
Supplementary Fig. 14f	Plant disease performance	Number of added genotypes over the control for tropical zones	71	14	69	Y = 0.264X - 2.001	-0.4174	0.9449	0.3475	0.7590	0.4504
Supplementary Fig. 14g	Plant performance	Number of added genotypes over the control for tropical zones	189	31	187	Y = -0.052X + 0.669	-0.2628	0.1583	0.1074	-0.4862	0.6274

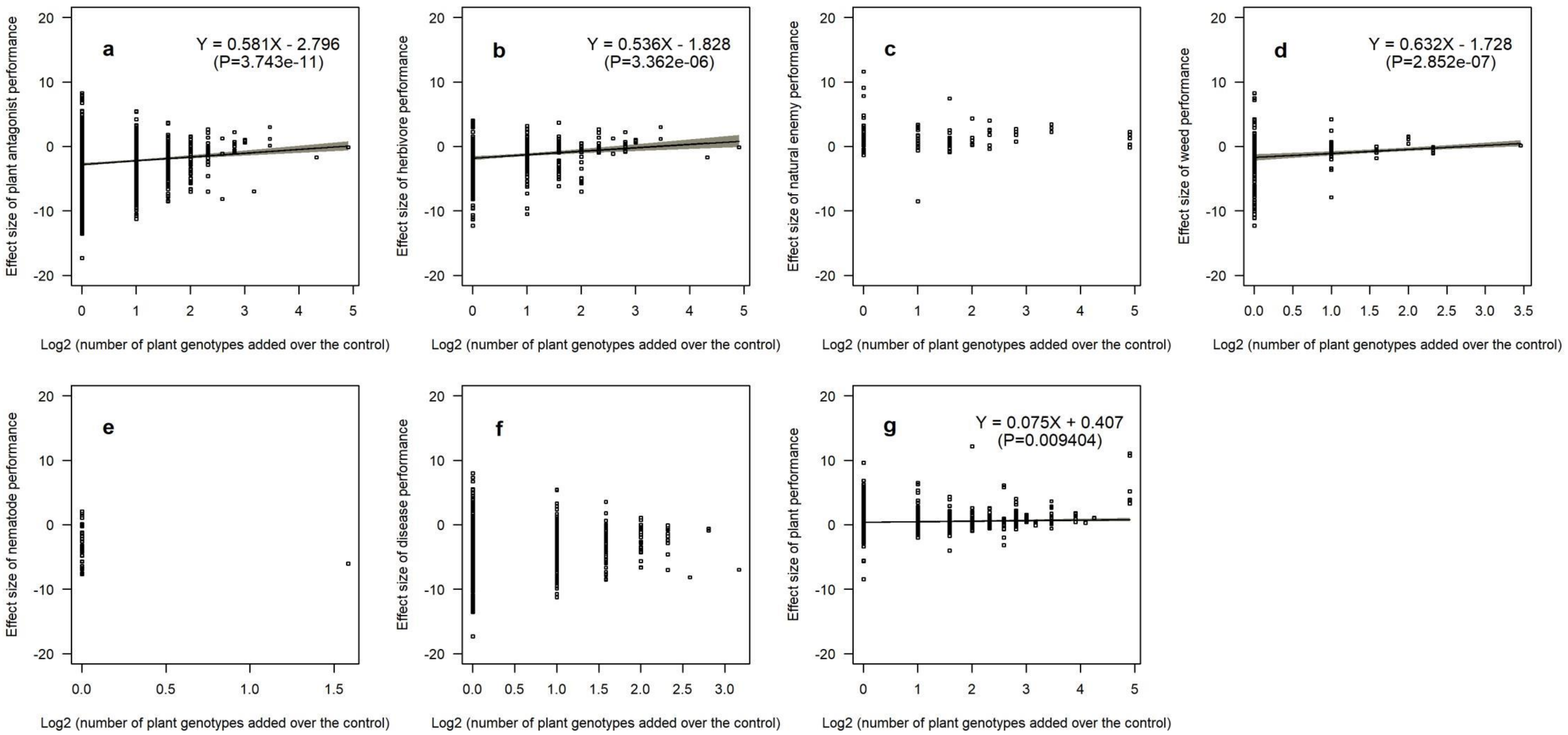

Supplementary Fig. 1 | Relationship between number of added genotypes in the plant genetic diversity treatment over the control and the different effect sizes along with fitted meta-regression lines across all studies. Note that a value of zero on the x axis indicates that only one genotype was added (log scale). **a**, Scatter plot for plant antagonist performance (1736 observations / 236 studies). **b**, Scatter plot for invertebrate herbivore performance (468 observations / 93 studies). **c**, Scatter plot for natural enemy performance (104 observations / 33 studies). **d**, Scatter plot for weed performance (200 observations / 14 studies). **e**, Scatter plot for plant-feeding nematode performance (35 observations / 7 studies). **f**, Scatter plot for plant disease performance (1033 observations / 136 studies). **g**, Scatter plot for plant performance (2862 observations / 320 studies). Plant antagonist performance include herbivore, weed, nematode and disease performances. Herbivore performance includes herbivore abundance, herbivore damage and herbivore diversity. Natural enemy performance includes predator abundance and diversity of predators, abundance and diversity of parasitoids and parasitism. Weed performance includes weed growth and weed diversity. Nematode performance is nematode abundance. Disease performance includes spread and damage of disease. Plant performance includes growth, reproduction and quality of plants. The dark shaded region indicates the 95% confidence interval for the predicted average SMD. The regression model intercepts, slopes and the P-values for the slopes are presented when the slope was significant.

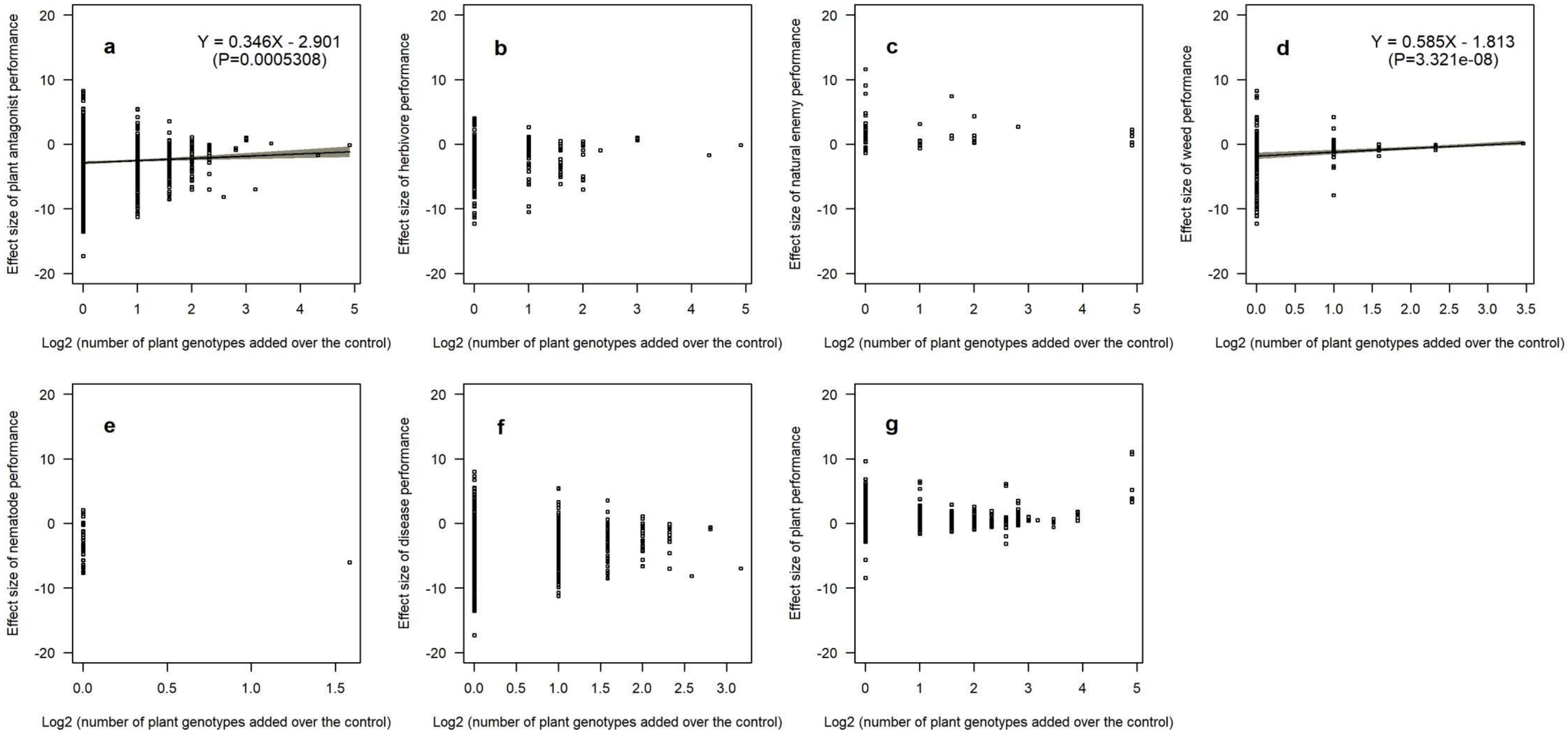

Supplementary Fig. 2 | Relationship between log-transformed number of added genotypes in the plant genetic diversity treatment over the control and the different effect sizes along with fitted meta-regression lines in agroecosystems. **a**, Scatter plot for plant antagonist performance (1536 observations / 195 studies). **b**, Scatter plot for invertebrate herbivore performance (311 observations / 57 studies). **c**, Scatter plot for natural enemy performance (52 observations / 17 studies). **d**, Scatter plot for weed performance (178 observations / 12 studies). **e**, Scatter plot for plant-feeding nematode performance (35 observations / 7 studies). **f**, Scatter plot for plant disease performance (1013 observations / 131 studies). **g**, Scatter plot for plant performance (2538 observations / 267 studies). Plant antagonist performance include herbivore, weed, nematode and disease performances. Herbivore performance includes herbivore abundance, herbivore damage and herbivore diversity. Natural enemy performance includes predator abundance and diversity of predators, abundance and diversity of parasitoids and parasitism. Weed performance includes weed growth and weed diversity. Nematode performance is nematode abundance. Disease performance includes spread and damage of disease. Plant performance includes growth, reproduction and quality of plants. The dark shaded region indicates the 95% confidence interval for the predicted average SMD. The regression model intercepts, slopes and the P-values for the slopes are presented when the slope was significant.

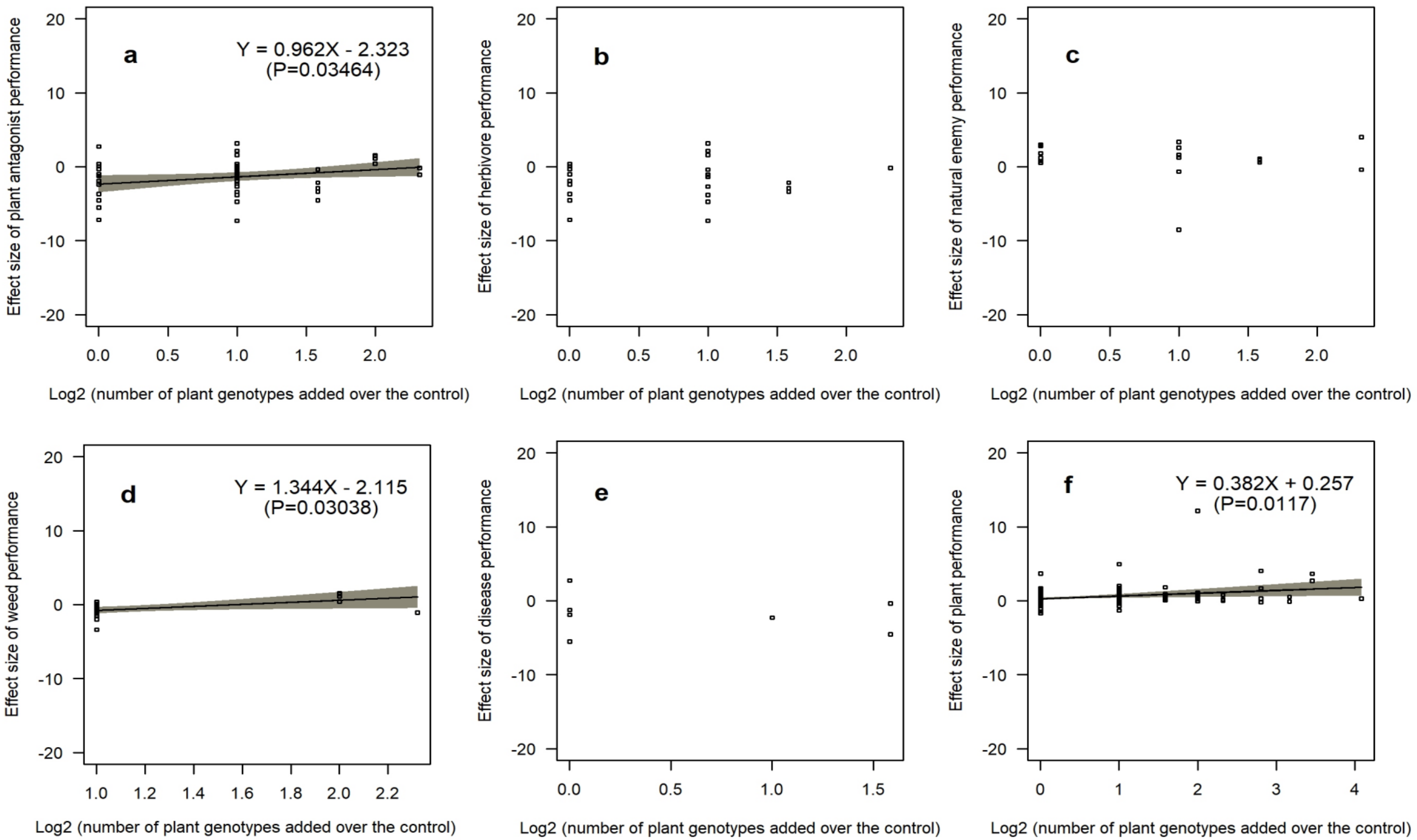

Supplementary Fig. 3 | Relationship between log-transformed number of added genotypes in the plant genetic diversity treatment over the control and the different effect sizes along with fitted meta-regression lines in grasslands. **a**, Scatter plot for plant antagonist performance (55 observations / 9 studies). **b**, Scatter plot for invertebrate herbivore performance (25 observations / 5 studies). **c**, Scatter plot for natural enemy performance (20 observations / 3 studies). **d**, Scatter plot for weed performance (22 observations / 2 studies). **e**, Scatter plot for plant disease performance (8 observations / 3 studies). **f**, Scatter plot for plant performance (134 observations / 17 studies). Plant antagonist performance include herbivore, weed, nematode and disease performances. No scatter plots were found for plant-feeding nematode (i.e., 0 observations / 0 studies). Herbivore performance includes herbivore abundance, herbivore damage and herbivore diversity. Natural enemy performance includes predator abundance and diversity of predators, abundance and diversity of parasitoids and parasitism. Weed performance includes weed growth and weed diversity. Nematode performance is nematode abundance. Disease performance includes spread and damage of disease. Plant performance includes growth, reproduction and quality of plants. The dark shaded region indicates the 95% confidence interval for the predicted average SMD. The regression model intercepts, slopes and the P-values for the slopes are presented when the slope was significant.

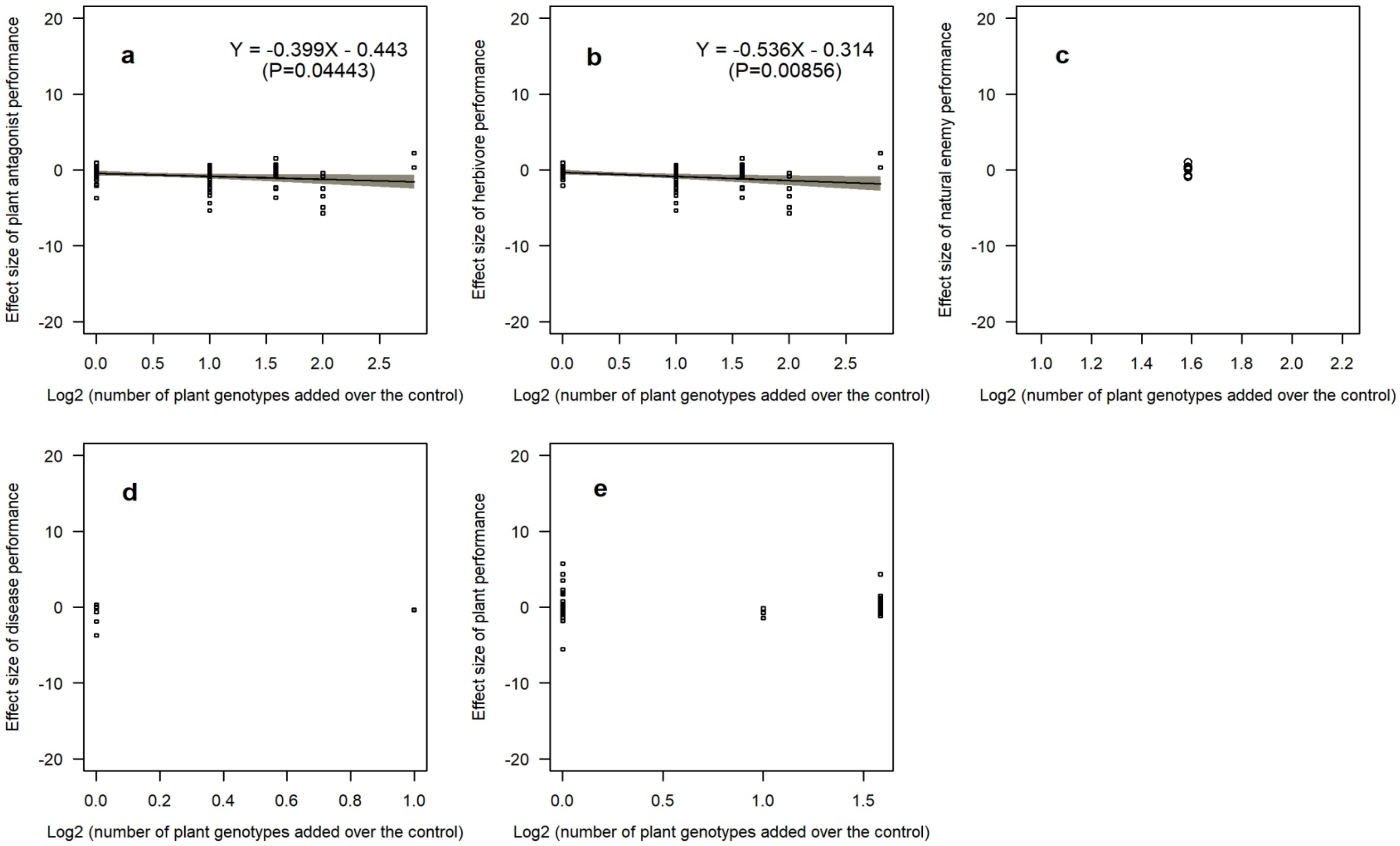

Supplementary Fig. 4 | Relationship between log-transformed number of added genotypes in the plant genetic diversity treatment over the control and the different effect sizes along with fitted meta-regression lines in forests. **a**, Scatter plot for plant antagonist performance (89 observations /14 studies). **b**, Scatter plot for invertebrate herbivore performance (79 observations / 14 studies). **c**, Scatter plot for natural enemy performance (7 observations /5 studies). **d**, Scatter plot for plant disease performance (10 observations /1 studies). **e**, Scatter plot for plant performance (61 observations / 4 studies). No relationship between added genotypes and natural enemy performance (7 observations / 5 studies) was found. No scatter plots were found for weed performance (0 observations / 0 studies) or plant-feeding nematode performance (0 observations / 0 studies). Plant antagonist performance include herbivore, weed, nematode and disease performances. Herbivore performance includes herbivore abundance, herbivore damage and herbivore diversity. Natural enemy performance includes predator abundance and diversity of predators, abundance and diversity of parasitoids and parasitism. Weed performance includes weed growth and weed diversity. Nematode performance is nematode abundance. Disease performance includes spread and damage of disease. Plant performance includes growth, reproduction and quality of plants. The dark shaded region indicates the 95% confidence interval for the predicted average SMD. The regression model intercepts, slopes and the P-values for the slopes are presented when the slope was significant.

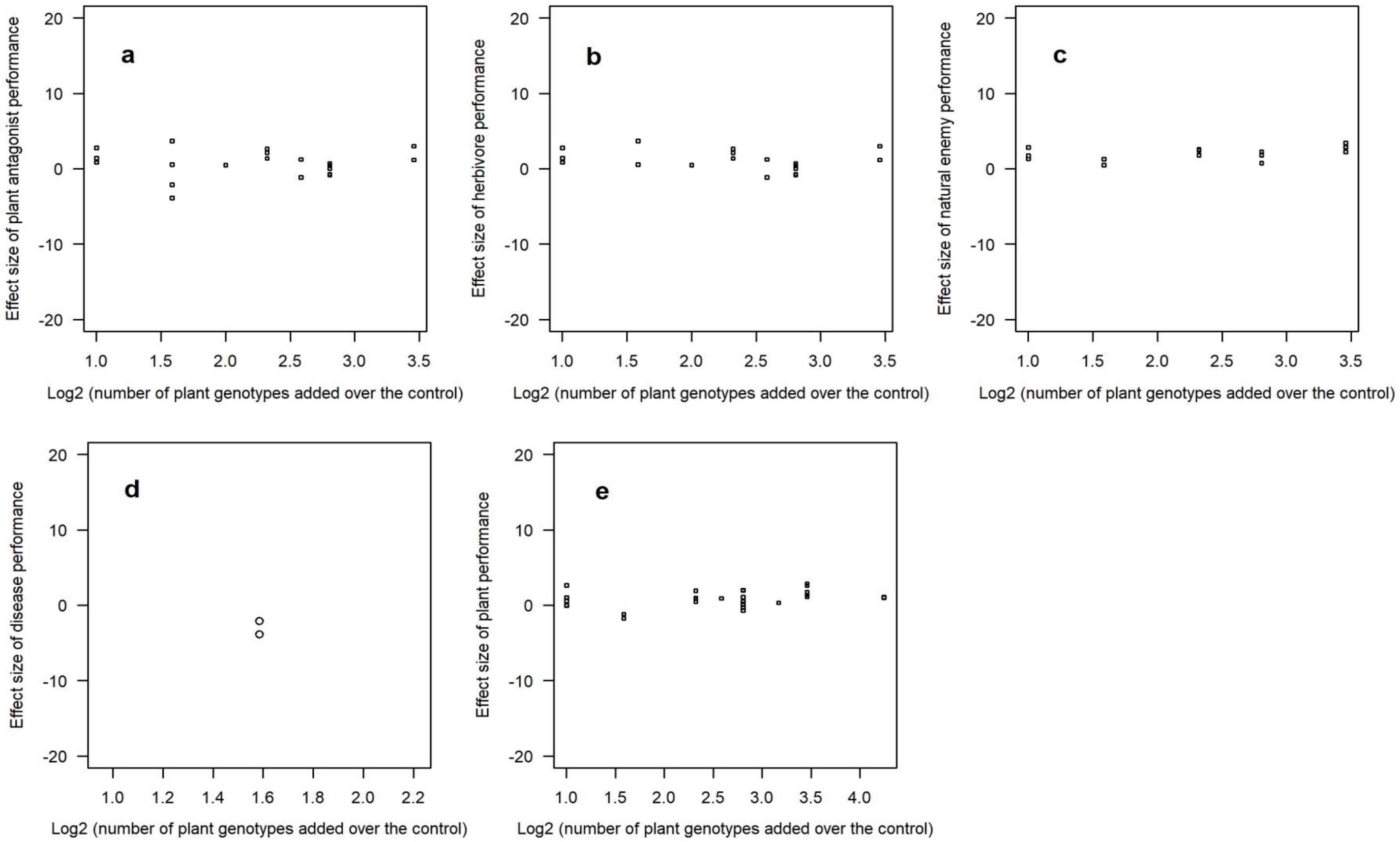

Supplementary Fig. 5 | Relationship between log-transformed number of added genotypes in the plant genetic diversity treatment over the control and the different effect sizes in old-field ecosystems. **a**, Scatter plot for plant antagonist performance (20 observations /10 studies). **b**, Scatter plot for invertebrate herbivore performance (18 observations / 9 studies). **c**, Scatter plot for natural enemy performance (14 observations / 4 studies). **d**, Scatter plot for plant disease performance (2 observations / 1 study). **e**, Scatter plot for plant performance (27 observations / 11 studies). No relationship between added genotypes and plant disease performance (2 observations /1 studies) was found. No scatter plots were found for weed performance (0 observations / 0 studies) or plant-feeding nematode performance (0 observations / 0 studies). Plant antagonist performance include herbivore, weed, nematode and disease performances. Herbivore performance includes herbivore abundance, herbivore damage and herbivore diversity. Natural enemy performance includes predator abundance and diversity of predators, abundance and diversity of parasitoids and parasitism. Weed performance includes weed growth and weed diversity. Nematode performance is nematode abundance. Disease performance includes spread and damage of disease. Plant performance includes growth, reproduction and quality of plants. The dark shaded region indicates respectively the 95% confidence interval for the predicted average SMD. The regression model intercepts, slopes and the P-values for the slopes are presented when the slope was significant.

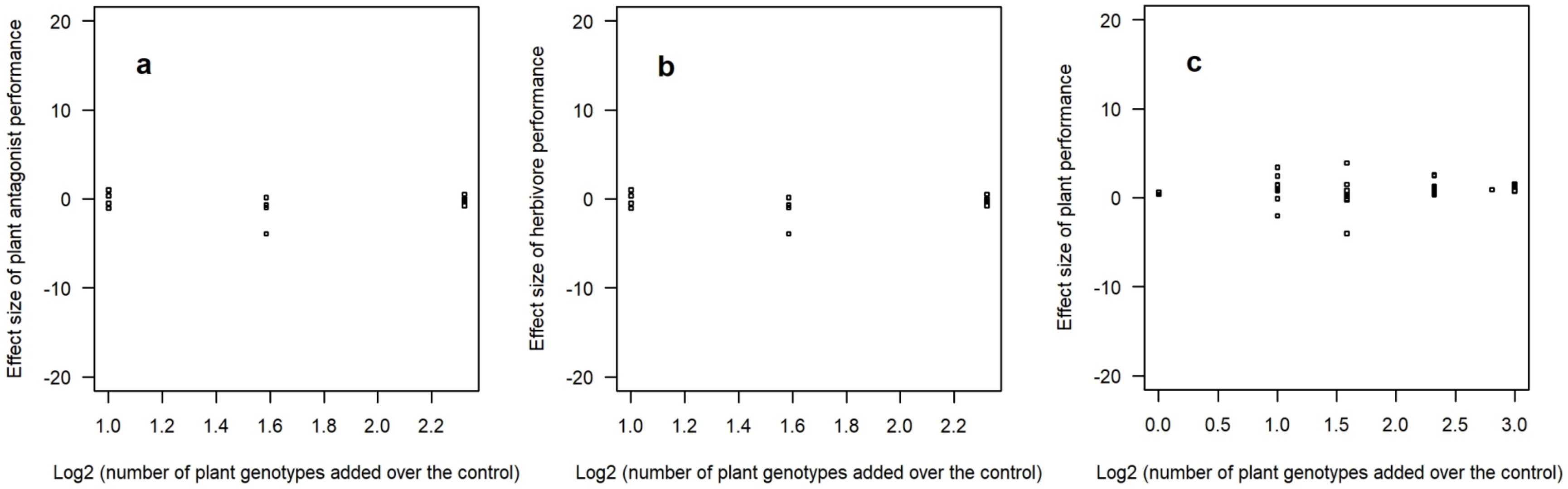

Supplementary Fig. 6 | Relationship between log-transformed number of added genotypes in the plant genetic diversity treatment over the control and the different effect sizes in marine ecosystems. **a**, Scatter plot for plant antagonist performance (13 observations /5 studies). **b**, Scatter plot for invertebrate herbivore performance (13 observations / 5 studies). **c**, Scatter plot for plant performance (37 observations / 9 studies). No scatter plots were found for natural enemy performance (0 observations / 0 studies), weed performance (0 observations / 0 studies), plant-feeding nematode performance (0 observations / 0 studies) or plant disease performance (0 observations / 0 studies). Plant antagonist performance include herbivore, weed, nematode and disease performances. Herbivore performance includes herbivore abundance, herbivore damage and herbivore diversity. Natural enemy performance includes predator abundance and diversity of predators, abundance and diversity of parasitoids and parasitism. Weed performance includes weed growth and weed diversity. Nematode performance is nematode abundance. Disease performance includes spread and damage of disease. Plant performance includes growth, reproduction and quality of plants. The dark shaded region indicates the 95% confidence interval for the predicted average SMD. The regression model intercepts, slopes and the P-values for the slopes are presented when the slope was significant.

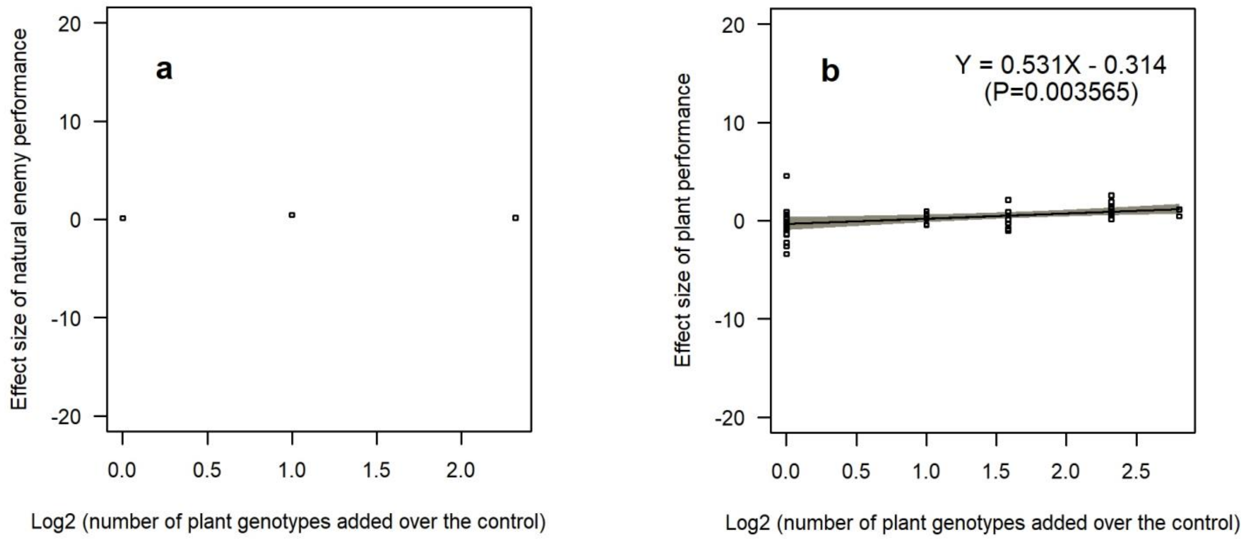

Supplementary Fig. 7 | Relationship between log-transformed number of added genotypes in the plant genetic diversity treatment over the control and the different effect sizes in wetlands. **A**, Scatter plot for natural enemy performance (3 observations / 1 studies). **B**, Scatter plot for plant performance (43 observations / 9 studies). No scatter plots were found for plant antagonist performance (0 observations /0 studies), invertebrate herbivore performance (0 observations / 0 studies), weed performance (0 observations / 0 studies), plant-feeding nematode performance (0 observations / 0 studies) or plant disease performance (0 observations / 0 studies). Plant antagonist performance include herbivore, weed, nematode and disease performances. Herbivore performance includes herbivore abundance, herbivore damage and herbivore diversity. Natural enemy performance includes predator abundance and diversity of predators, abundance and diversity of parasitoids and parasitism. Weed performance includes weed growth and weed diversity. Nematode performance is nematode abundance. Disease performance includes spread and damage of disease. Plant performance includes growth, reproduction and quality of plants. The dark shaded region indicates the 95% confidence interval for the predicted average SMD. The regression model intercepts, slopes and the P-values for the slopes are presented when the slope was significant.

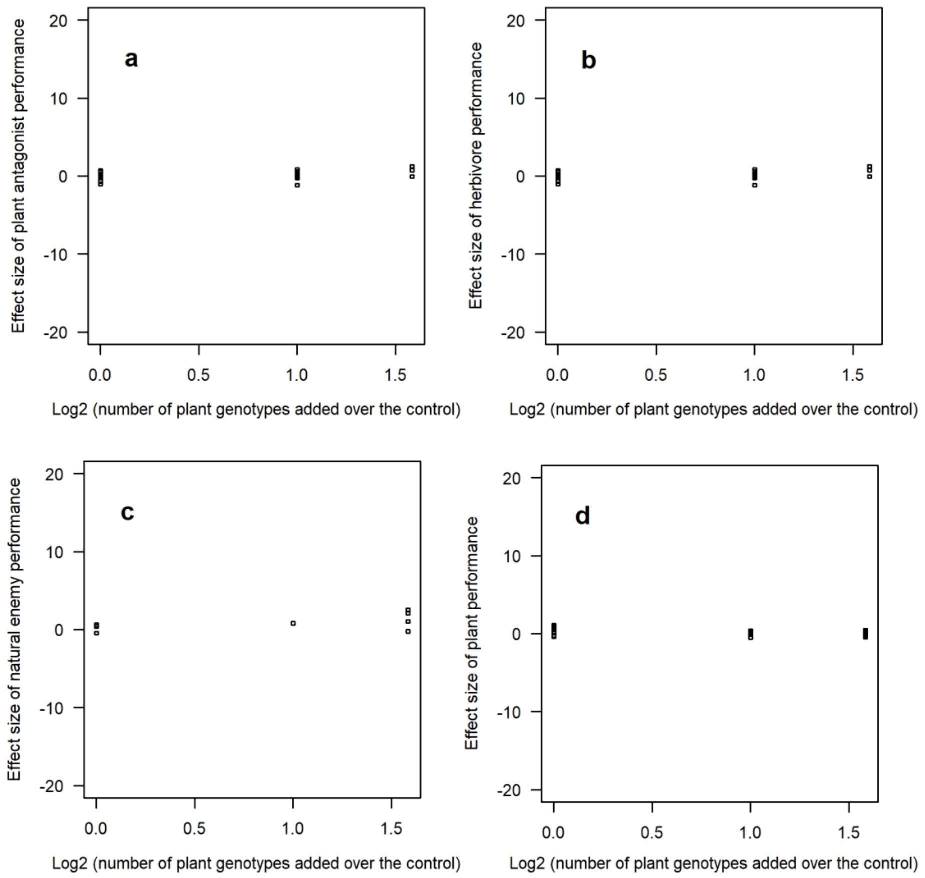

Supplementary Fig. 8 | Relationship between log-transformed number of added genotypes in the plant genetic diversity treatment over the control and the different effect sizes in shrublands. **A**, Scatter plot for plant antagonist performance (23 observations / 3 studies). **B**, Scatter plot for invertebrate herbivore performance (23 observations / 3 studies). **C**, Scatter plot for natural enemy performance (8 observations / 3 studies). **D**, Scatter plot for plant performance (22 observations / 3 studies). No scatter plots were found for weed performance (0 observations / 0 studies), plant-feeding nematode performance (0 observations / 0 studies) or plant disease performance (0 observations / 0 studies). Plant antagonist performance include herbivore, weed, nematode and disease performances. Herbivore performance includes herbivore abundance, herbivore damage and herbivore diversity. Natural enemy performance includes predator abundance and diversity of predators, abundance and diversity of parasitoids and parasitism. Weed performance includes weed growth and weed diversity. Nematode performance is nematode abundance. Disease performance includes spread and damage of disease. Plant performance includes growth, reproduction and quality of plants. The dark shaded region indicates the 95% confidence interval for the predicted average SMD. The regression model intercepts, slopes and the P-values for the slopes are presented when the slope was significant.

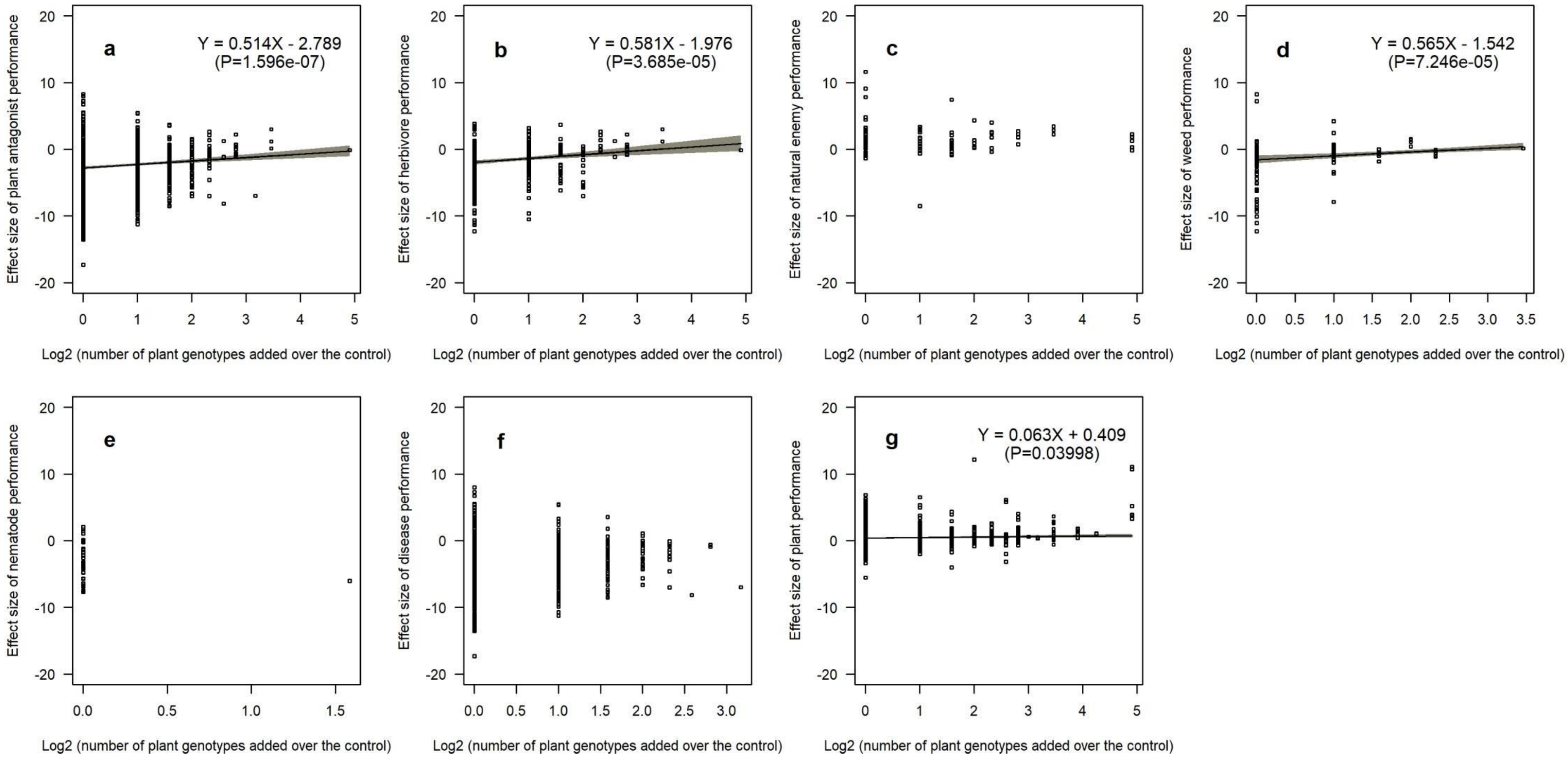

Supplementary Fig. 9 | Relationship between log-transformed number of added genotypes in the plant genetic diversity treatment over the control and the different effect sizes along with fitted meta-regression lines in plot experiments. **a**, Scatter plot for plant antagonist performance (1582 observations /216 studies). **b**, Scatter plot for invertebrate herbivore performance (406 observations / 78 studies). **c**, Scatter plot for natural enemy performance (100 observations / 32 studies). **d**, Scatter plot for weed performance (131 observations / 13 studies). **e**, Scatter plot for plant-feeding nematode performance (35 observations / 7 studies). **f**, Scatter plot for plant disease performance (1010 observations /132 studies). **g**, Scatter plot for plant performance (2667 observations /286 studies). Plant antagonist performance include herbivore, weed, nematode and disease performances. Herbivore performance includes herbivore abundance, herbivore damage and herbivore diversity. Natural enemy performance includes predator abundance and diversity of predators, abundance and diversity of parasitoids and parasitism. Weed performance includes weed growth and weed diversity. Nematode performance is nematode abundance. Disease performance includes spread and damage of disease. Plant performance includes growth, reproduction and quality of plants. The dark shaded region indicates the 95% confidence interval for the predicted average SMD. The regression model intercepts, slopes and the P-values for the slopes are presented when the slope was significant.

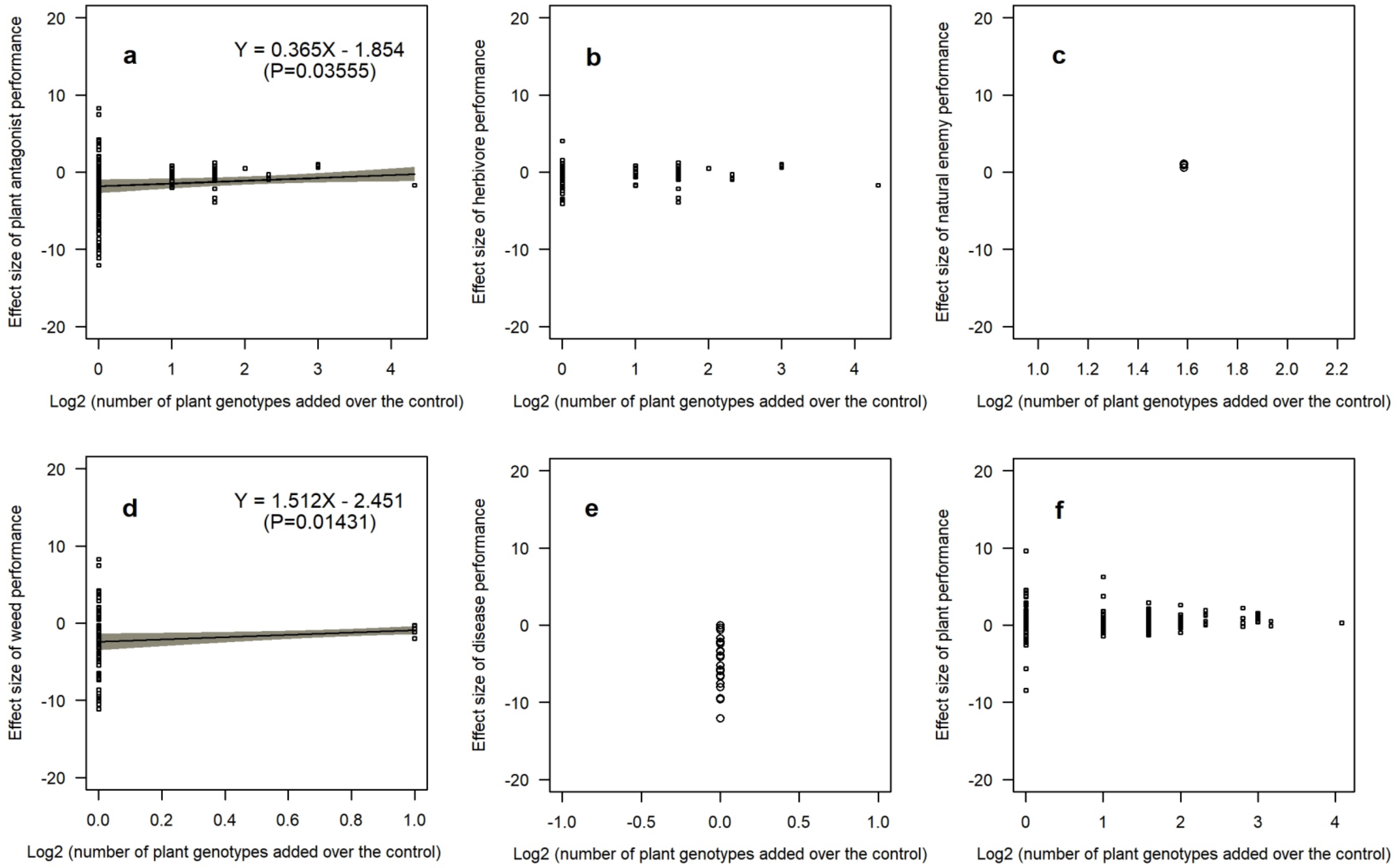

Supplementary Fig. 10 | Relationship between log-transformed number of added genotypes in the plant genetic diversity treatment over the control and the different effect sizes along with fitted meta-regression lines in pot experiments. **a**, Scatter plot for plant antagonist performance (154 observations / 24 studies). **b**, Scatter plot for invertebrate herbivore performance (62 observations / 16 studies). **c**, Scatter plot for natural enemy performance (4 observations / 1 study). **d**, Scatter plot for weed performance (69 observations / 3 studies). **e**, Scatter plot for weed performance (79 observations / 3 studies). **f**, Scatter plot for plant performance (195 observations / 34 studies). No relationships between added genotypes and natural enemy performance (4 observations / 1 studies) and between added genotypes and plant disease performance (23 observations / 5 studies) were found. No scatter plots were found for plant-feeding nematode performance (0 observations / 0 studies). Plant antagonist performance include herbivore, weed, nematode and disease performances. Herbivore performance includes herbivore abundance, herbivore damage and herbivore diversity. Natural enemy performance includes predator abundance and diversity of predators, abundance and diversity of parasitoids and parasitism. Weed performance includes weed growth and weed diversity. Nematode performance is nematode abundance. Disease performance includes spread and damage of disease. Plant performance includes growth, reproduction and quality of plants. The dark shaded region indicates the 95% confidence interval for the predicted average SMD. The regression model intercepts, slopes and the P-values for the slopes are presented when the slope was significant.

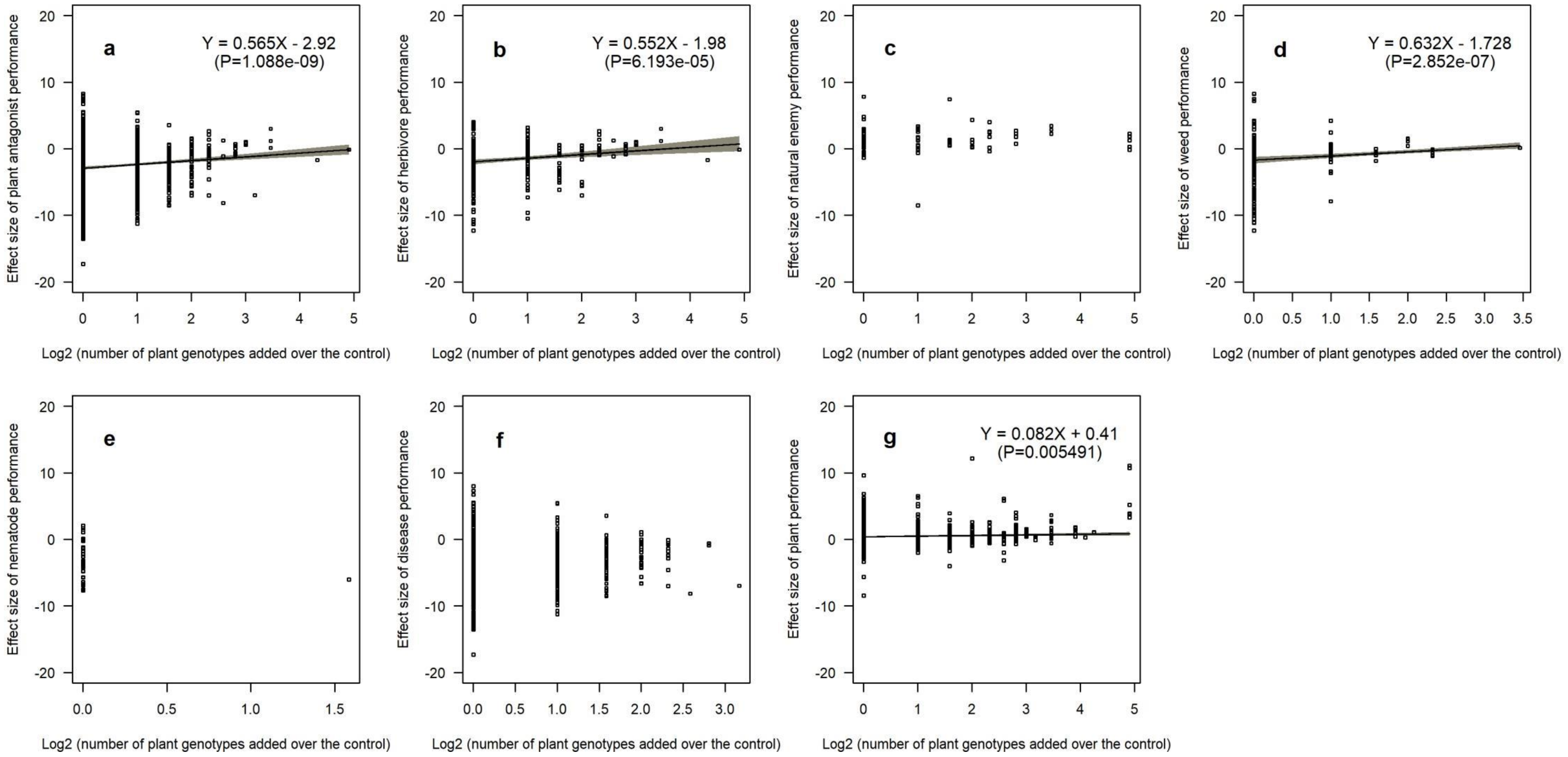

Supplementary Fig. 11 | Relationship between log-transformed number of added genotypes in the plant genetic diversity treatment over the control and the different effect sizes along with fitted meta-regression lines for herbaceous plants. **a**, Scatter plot for plant antagonist performance (1537 observations / 207 studies). **b**, Scatter plot for invertebrate herbivore performance (340 observations / 66 studies). **c**, Scatter plot for natural enemy performance (84 observations / 23 studies). **d**, Scatter plot for weed performance (200 observations / 14 studies). **e**, Scatter plot for plant-feeding nematode performance (35 observations / 7 studies). **f**, Scatter plot for plant disease performance (1010 observations / 131 studies). **g**, Scatter plot for plant performance (2741 observations / 306 studies). Plant antagonist performance include herbivore, weed, nematode and disease performances. Herbivore performance includes herbivore abundance, herbivore damage and herbivore diversity. Natural enemy performance includes predator abundance and diversity of predators, abundance and diversity of parasitoids and parasitism. Weed performance includes weed growth and weed diversity. Nematode performance is nematode abundance. Disease performance includes spread and damage of disease. Plant performance includes growth, reproduction and quality of plants. The dark shaded region indicates the 95% confidence interval for the predicted average SMD. The regression model intercepts, slopes and the P-values for the slopes are presented when the slope was significant.

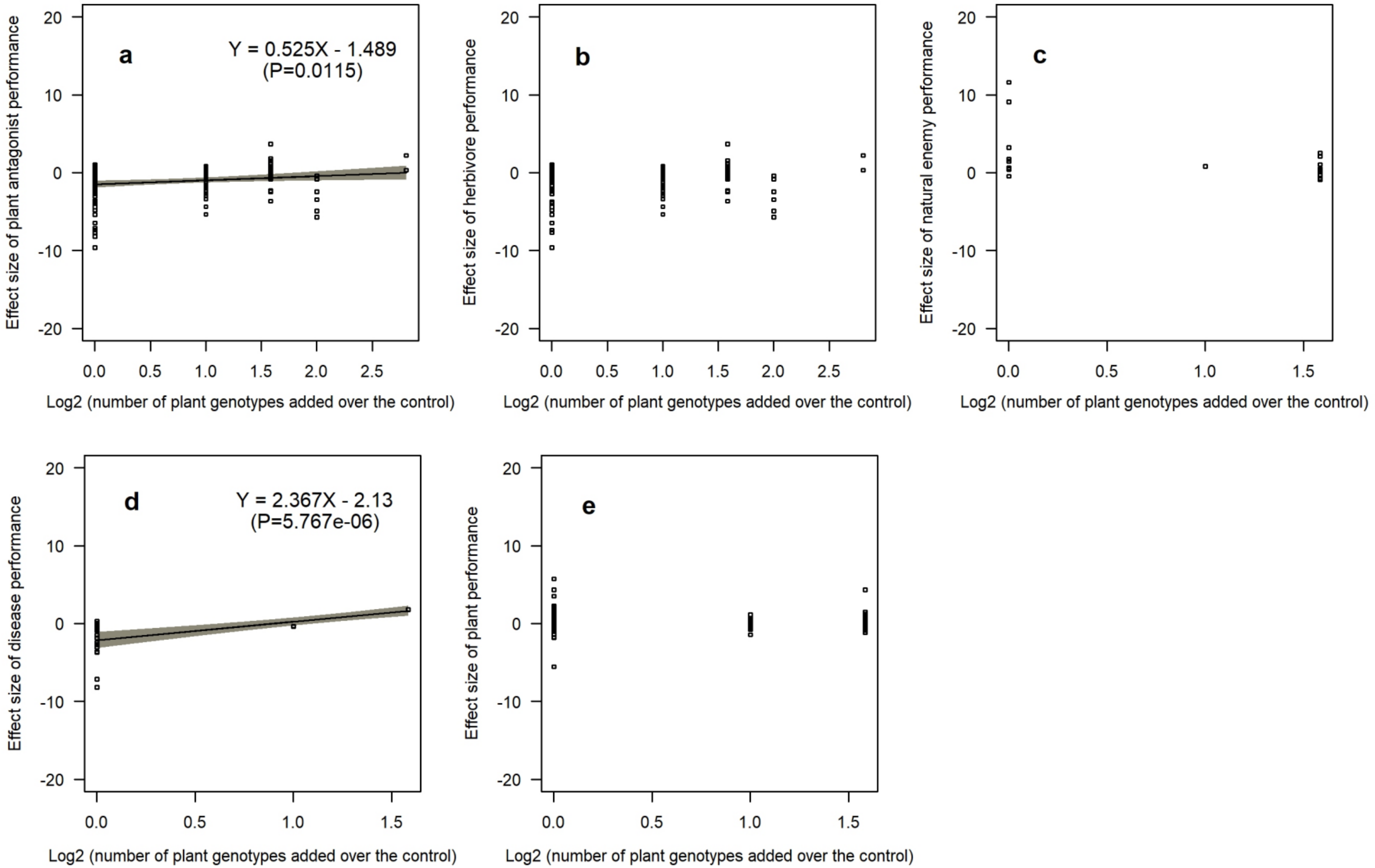

Supplementary Fig. 12 | Relationship between log-transformed number of added genotypes in the plant genetic diversity treatment over the control and the different effect sizes along with fitted meta-regression lines for woody plants. **a**, Scatter plot for plant antagonist performance (157 observations / 29 studies). **b**, Scatter plot for invertebrate herbivore performance (134 observations / 28 studies). **c**, Scatter plot for natural enemy performance (20 observations / 10 studies). **d**, Scatter plot for plant disease performance (23 observations / 5 studies). **e**, Scatter plot for plant performance (121 observations /14 studies). No scatter plots were found for weed performance (0 observations / 0 studies) or plant-feeding nematode performance (0 observations / 0 studies). Plant antagonist performance include herbivore, weed, nematode and disease performances. Herbivore performance includes herbivore abundance, herbivore damage and herbivore diversity. Natural enemy performance includes predator abundance and diversity of predators, abundance and diversity of parasitoids and parasitism. Weed performance includes weed growth and weed diversity. Nematode performance is nematode abundance. Disease performance includes spread and damage of disease. Plant performance includes growth, reproduction and quality of plants. The dark shaded region indicates the 95% confidence interval for the predicted average SMD. The regression model intercepts, slopes and the P-values for the slopes are presented when the slope was significant.

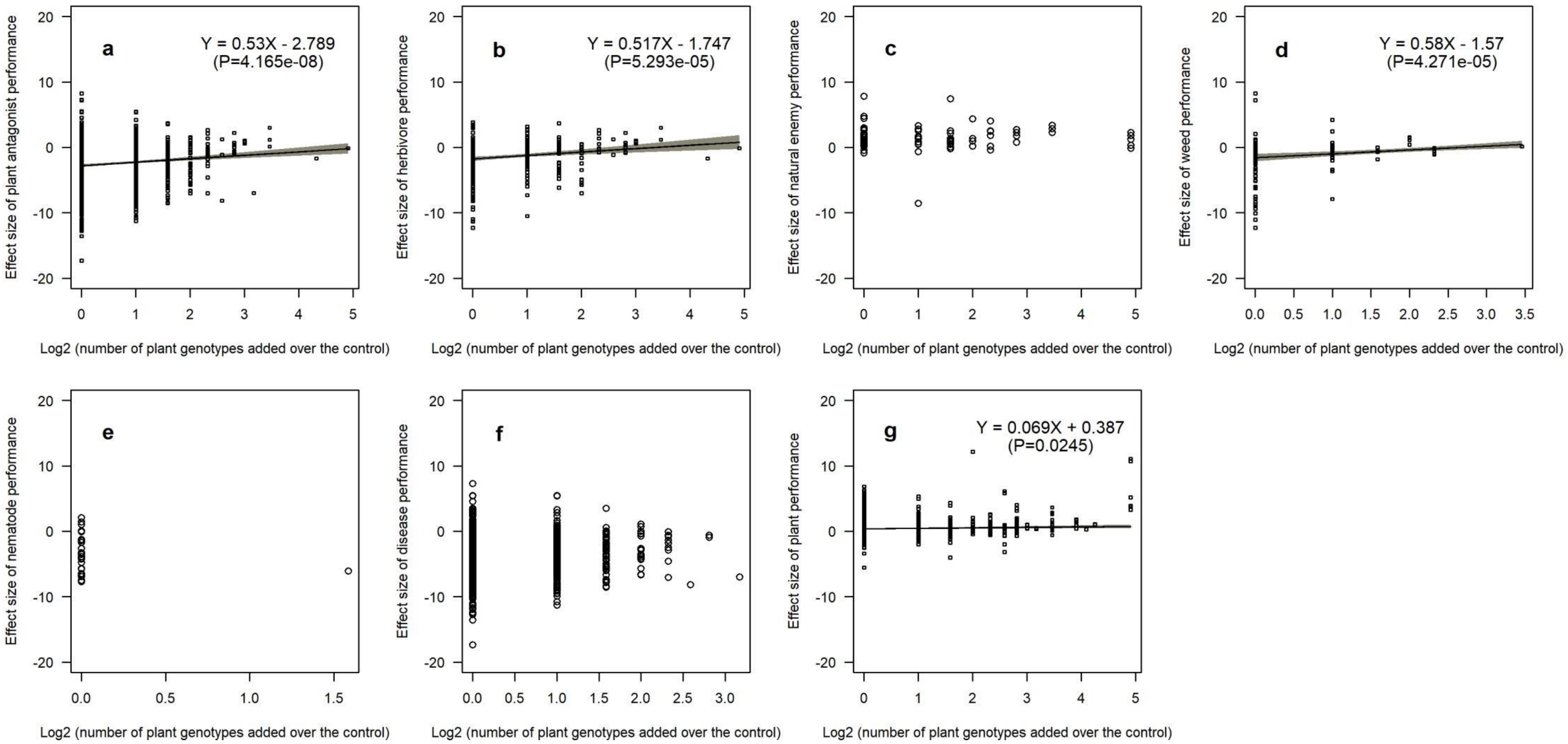

Supplementary Fig. 13 | Relationship between log-transformed number of added genotypes in the plant genetic diversity treatment over the control and the different effect sizes along with fitted meta-regression lines for temperate zones. **A**, Scatter plot for plant antagonist performance (1490 observations / 192 studies). **B**, Scatter plot for invertebrate herbivore performance (384 observations / 66 studies). **C**, Scatter plot for natural enemy performance (85 observations / 23 studies). **D**, Scatter plot for weed performance (138 observations / 11 studies). **E**, Scatter plot for plant-feeding nematode performance (29 observations / 6 studies). **F**, Scatter plot for plant disease performance (939 observations / 119 studies). **G**, Scatter plot for plant performance (2516 observations / 266 studies). Plant antagonist performance include herbivore, weed, nematode and disease performances. Herbivore performance includes herbivore abundance, herbivore damage and herbivore diversity. Natural enemy performance includes predator abundance and diversity of predators, abundance and diversity of parasitoids and parasitism. Weed performance includes weed growth and weed diversity. Nematode performance is nematode abundance. Disease performance includes spread and damage of disease. Plant performance includes growth, reproduction and quality of plants. The dark shaded region indicates the 95% confidence interval for the predicted average SMD. The regression model intercepts, slopes and the P-values for the slopes are presented when the slope was significant. If the data are from greenhouse or indoor conditions, they have been removed from the figures.

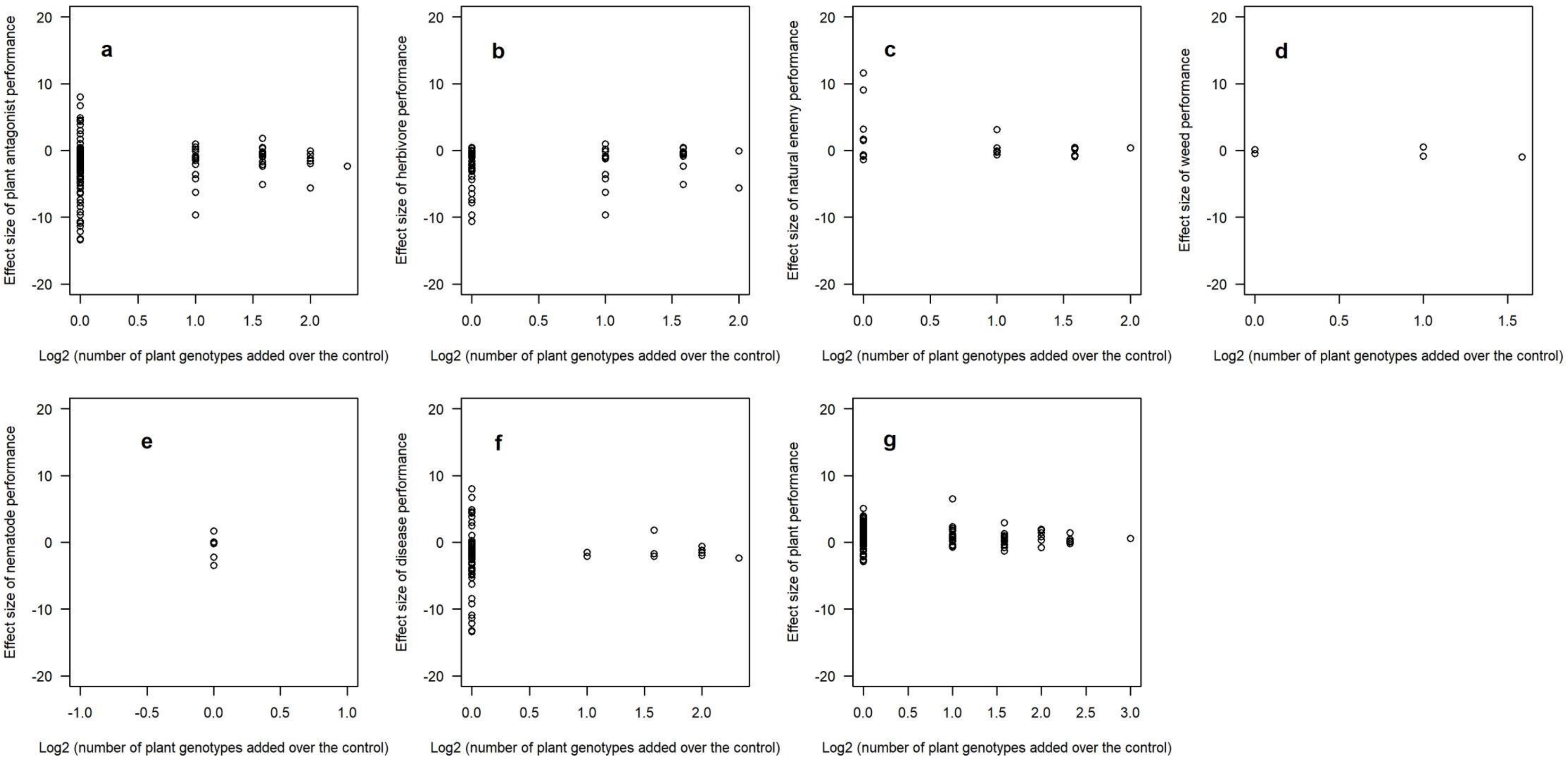

Supplementary Fig. 14 | Relationship between log-transformed number of added genotypes in the plant genetic diversity treatment over the control and the different effect sizes for tropical zones. **a**, Scatter plot for plant antagonist performance (140 observations / 32 studies). **b**, Scatter plot for invertebrate herbivore performance (58 observations / 20 studies). **c**, Scatter plot for natural enemy performance (19 observations / 11 studies). **d**, Scatter plot for weed performance (5 observations / 2 studies). **e**, Scatter plot for nematode performance (6 observations / 1 study). **f**, Scatter plot for plant disease performance (72 observations / 14 studies). **g**, Scatter plot for plant performance (189 observations / 31 studies). No relationship between added genotypes and plant-feeding nematode performance (6 observations / 1 studies) was found. Plant antagonist performance include herbivore, weed, nematode and disease performances. Herbivore performance includes herbivore abundance, herbivore damage and herbivore diversity. Natural enemy performance includes predator abundance and diversity of predators, abundance and diversity of parasitoids and parasitism. Weed performance includes weed growth and weed diversity. Nematode performance is nematode abundance. Disease performance includes spread and damage of disease. Plant performance includes growth, reproduction and quality of plants. The dark shaded region indicates the 95% confidence interval for the predicted average SMD. The regression model intercepts, slopes and the P-values for the slopes are presented when the slope was significant. If the data were from greenhouse or indoor conditions, they have been removed from the figures.

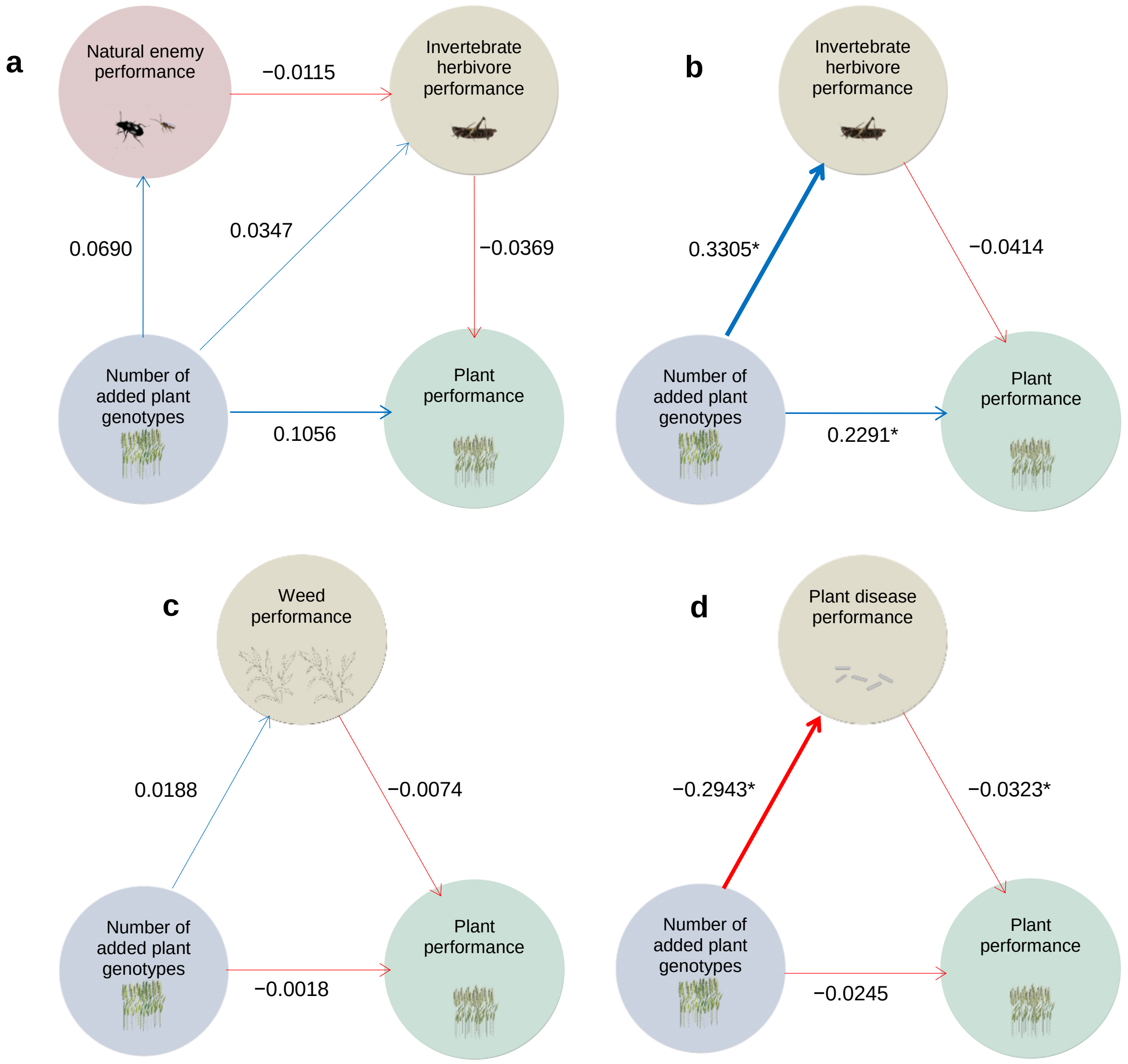

Supplementary Fig. 15 | Path analysis for the effects of number of added plant genotypes in all analyzed ecosystems. a, on tri-trophic interactions of invertebrate herbivore, natural enemy and plant performances. **b,** on bi-trophic interactions of invertebrate herbivore and plant performance. **c,** on bi-trophic interactions of weed and plant performance. **d,** on bi-trophic interactions of plant disease and plant performance. No data are found to test the effects of the number of added plant genotypes on bi-trophic interactions of plant-feeding nematode and plant performance. Herbivore performance, weed performance (growth and diversity of weeds), nematode performance (nematode abundance), and disease performance (disease spread and damage) are shown in beige circles. Plant genetic diversity is shown in dusty blue. Natural enemy performance (predator abundance, predator diversity, parasitoid abundance, parasitoid diversity and parasitism) is shown in pink. Plant performance (growth, quality and reproduction of plants) is shown in teal. The blue and red arrows denote positive and negative relationships respectively, numbers beside each arrow are the estimate coefficients for the fitted path-analytic models (Supplementary Tables 9, 10), and line width is proportional to the magnitude of the presented coefficient. The asterisks indicate significance at 5% level.

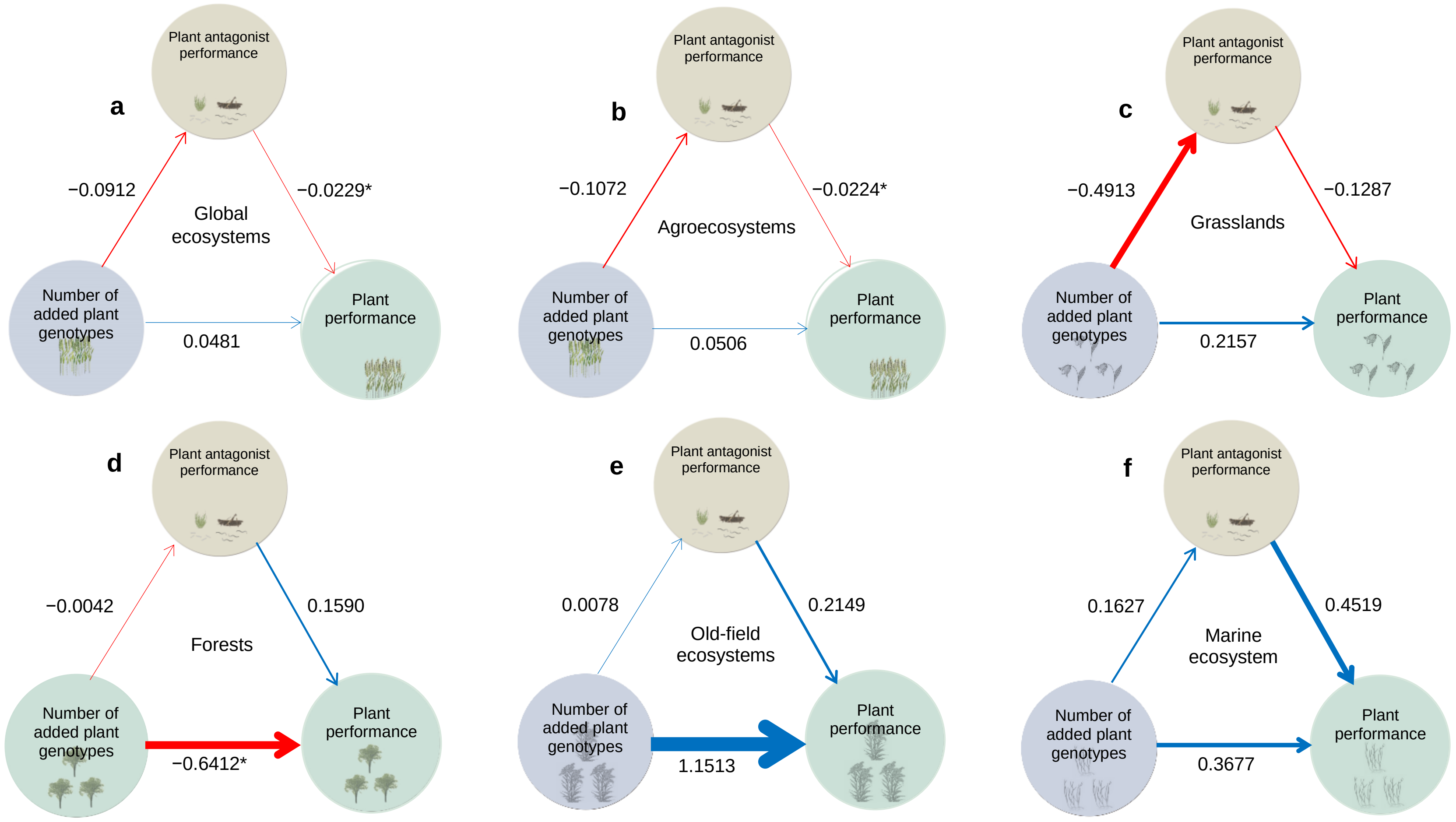

Supplementary Fig. 16 | Path analysis for the effects of number of added plant genotypes on the bi-trophic interactions of plant antagonist and plant performance. a, In global ecosystems. **b,** In agroecosystems. **c**, In grasslands. **d**, In forests. **e**, In old-field ecosystems. **f**, In marine ecosystems. No bi-trophic interactions were found in wetlands or shrublands. Plant antagonist performance including herbivore performance (abundance, damage and diversity of herbivores), weed performance (growth and diversity of weeds), plant-feeding nematode performance (nematode abundance) and plant disease performance (disease spread and damage) are shown in beige circles. Plant genetic diversity is shown in dusty blue. Plant performance (growth, quality and reproduction of plants) is shown in teal. The blue and red arrows denote positive and negative relationships respectively, numbers beside each arrow are the estimate coefficients for the fitted path-analytic models (Supplementary Tables 11, 12), and line width is proportional to the magnitude of the presented coefficient. The asterisks indicate significance at 5% level

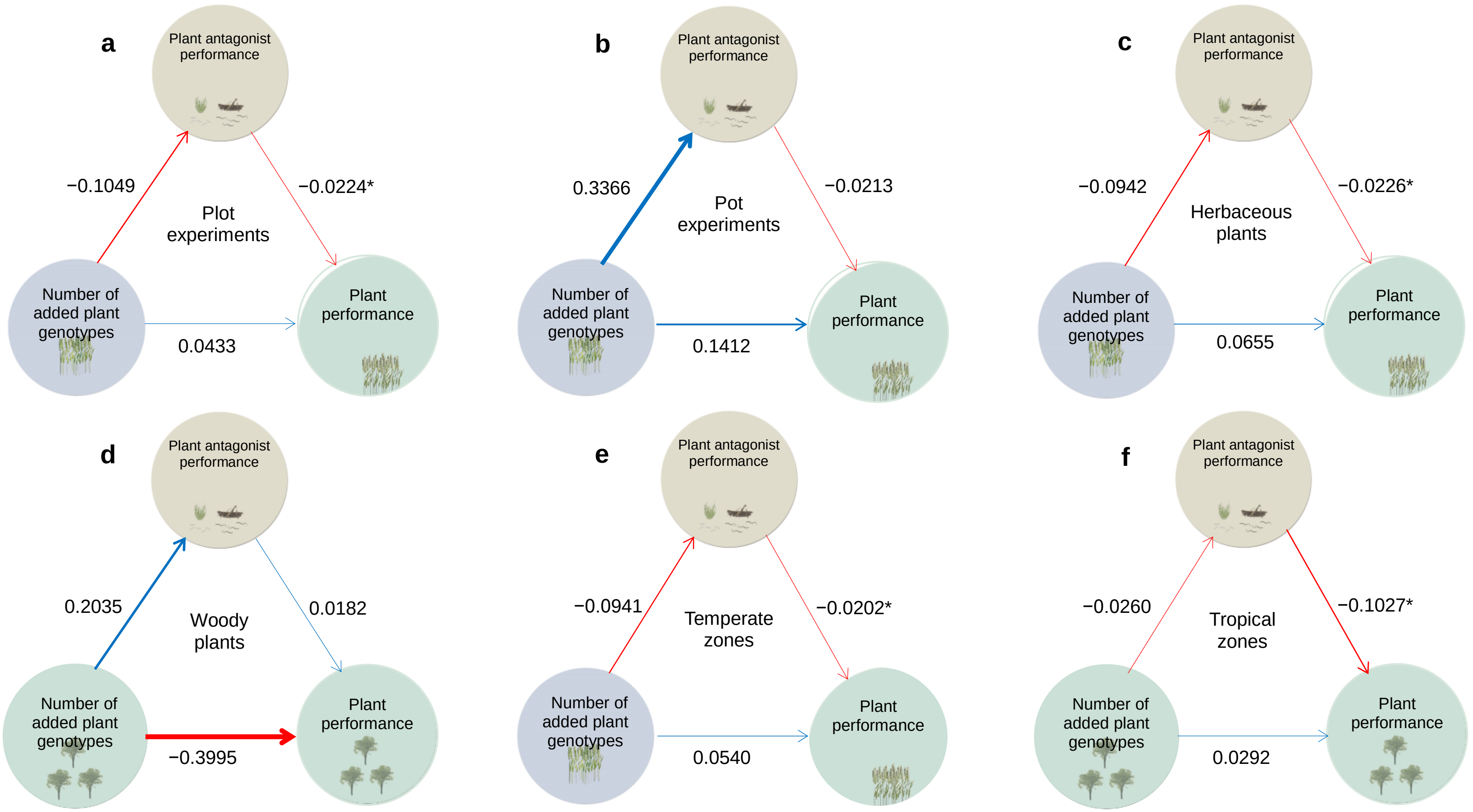

Supplementary Fig. 17 | Path analysis for the effects of number of added plant genotypes on the bi-trophic interactions of plant antagonist and plant performance. a, In plot experiments. **b,** In pot experiments. **c,** In herbaceous plants. **d,** In woody plants. **e,** In temperate zones. **f,** In tropical zones. Plant antagonist performance including herbivore performance (abundance, damage and diversity of herbivores), weed performance (growth and diversity of weeds), nematode performance (nematode abundance), and disease performance (disease spread and damage) are shown in beige circles. Plant genetic diversity is shown in dusty blue. Plant performance (growth, quality and reproduction of plants) is shown in teal. The blue and red arrows denote positive and negative relationships respectively, numbers beside each arrow are the estimate coefficients for the fitted path-analytic models (Supplementary Tables 13, 14), and line width is proportional to the magnitude of the presented coefficient. The asterisks indicate significance at 5% level.

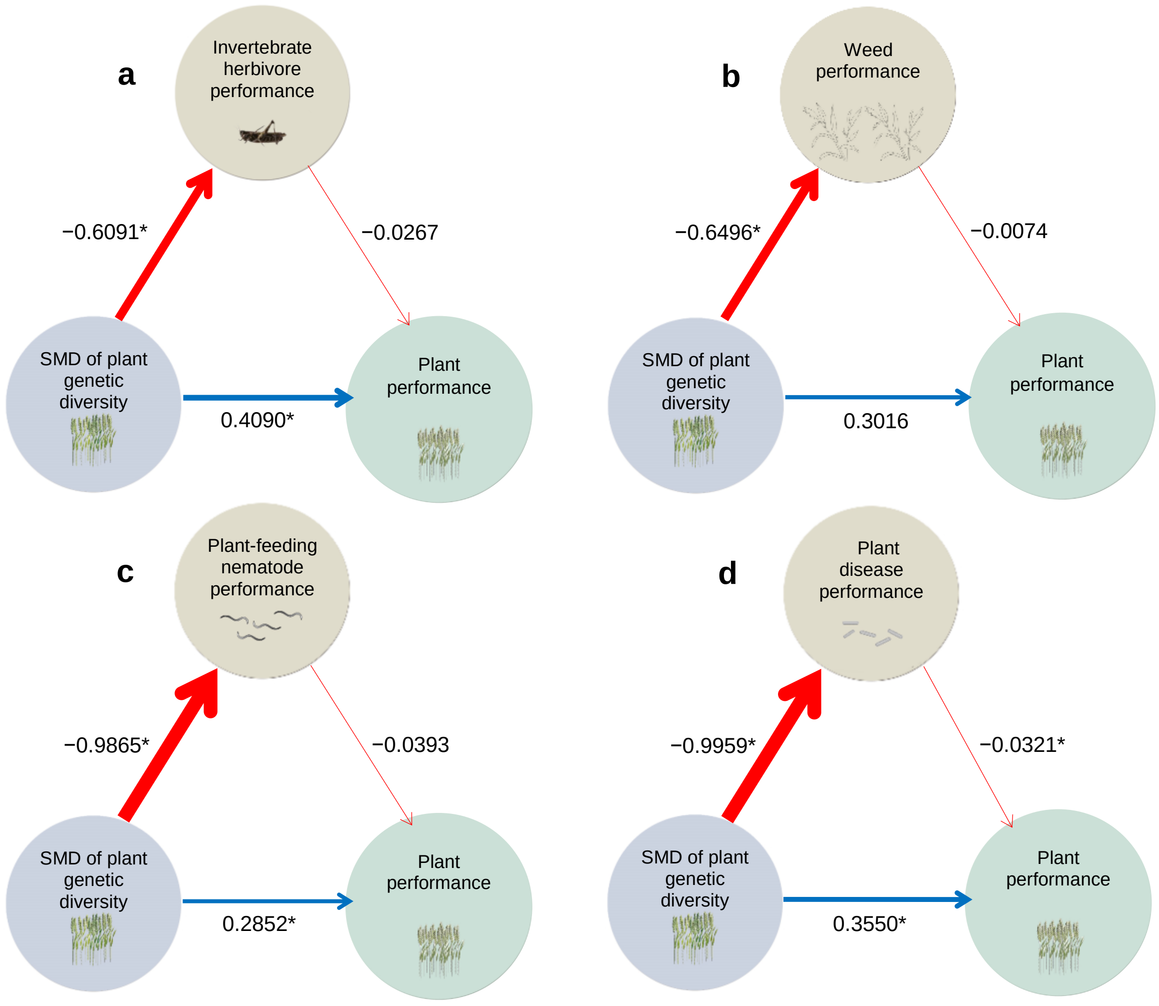

Supplementary Fig. 18 | Path analysis for the effects of SMD of plant genetic diversity across all analyzed ecosystems. SMD is the abbreviation of Standardized Mean Difference. **a,** on bi-trophic interactions of invertebrate herbivore and plant performance. **b,** on bi-trophic interactions of weed and plant performance. **c,** on bi-trophic interactions of plant-feeding nematode and plant performance. **d,** on bi-trophic interactions of plant disease and plant performance. Herbivore performance (abundance, damage and diversity of herbivores), weed performance (growth and diversity of weeds), nematode performance (nematode abundance), and disease performance (disease spread and damage) are shown in beige circles. Plant genetic diversity is shown in dusty blue. Plant performance (growth, quality and reproduction of plants) is shown in teal. The blue and red arrows denote positive and negative relationships respectively, numbers beside each arrow are the estimate coefficients for the fitted path-analytic models (Supplementary Tables 9, 10), and line width is proportional to the magnitude of the presented coefficient. The asterisks indicate significance at 5% level.

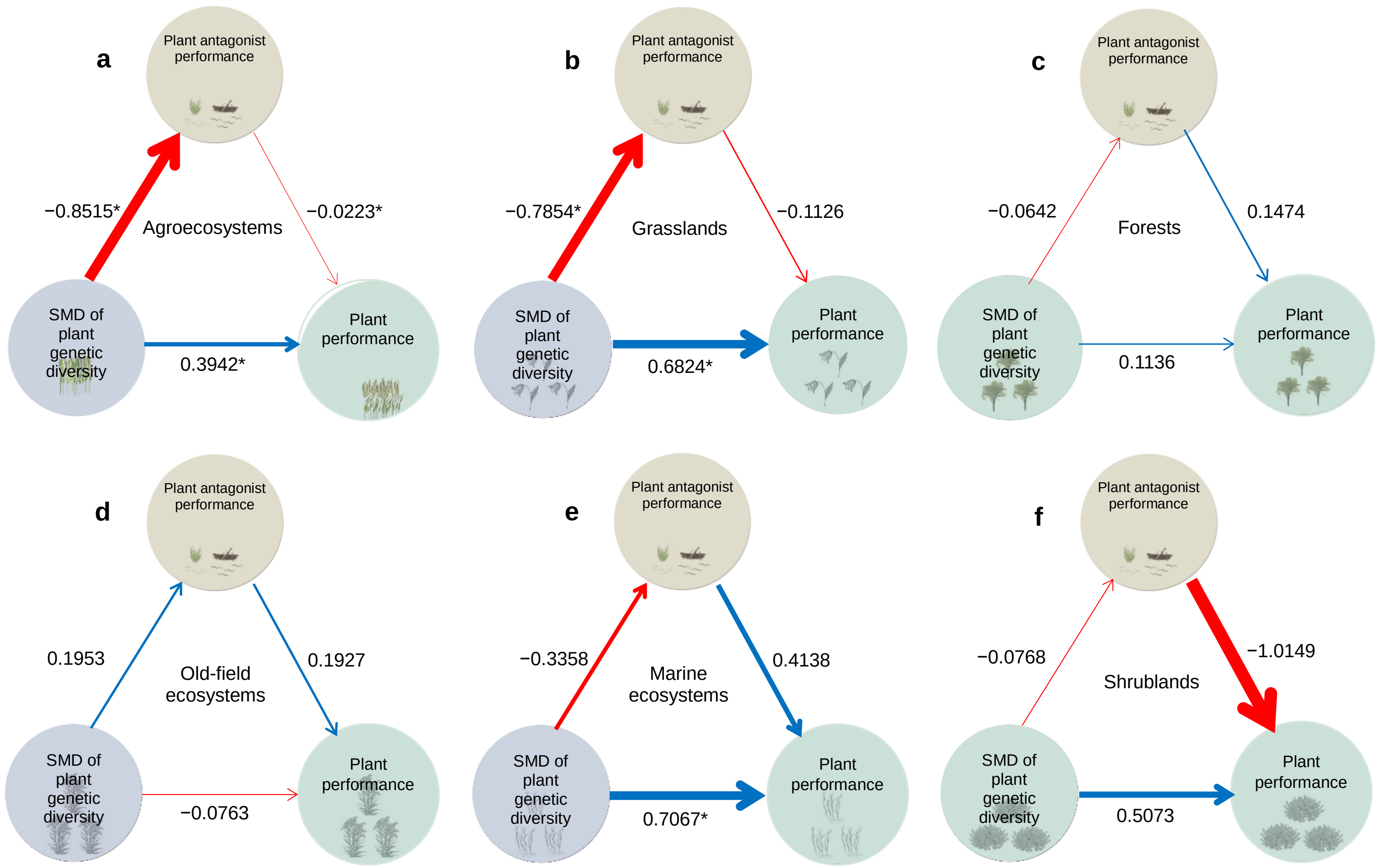

Supplementary Fig. 19 | Path analysis for the effects of SMD of plant genetic diversity on bi-trophic interactions of plant antagonist and plant performance on different ecosystems. SMD is the abbreviation of Standardized Mean Difference. **a,** agroecosystems. **b**, grasslands. **c**, forests. **d**, old-field ecosystems. **e**, marine ecosystems. **f**, shrublands. Bi-trophic interactions between plant antagonist and plant performance were not found in wetlands. Plant antagonist performance including herbivore performance (abundance, damage and diversity of herbivores), weed performance (growth and diversity of weeds), plant-feeding nematode performance (nematode abundance) and plant disease performance (disease spread and damage) are shown in beige circles. Plant genetic diversity is shown in dusty blue. Plant performance (growth, quality and reproduction of plants) is shown in teal. Blue and red arrows denote positive and negative relationships respectively, numbers beside each arrow are the estimated coefficients for the fitted path-analytic models (Supplementary Tables 11, 12), and line width is proportional to the magnitude of the presented coefficient. The asterisks indicate significance at 5% level.

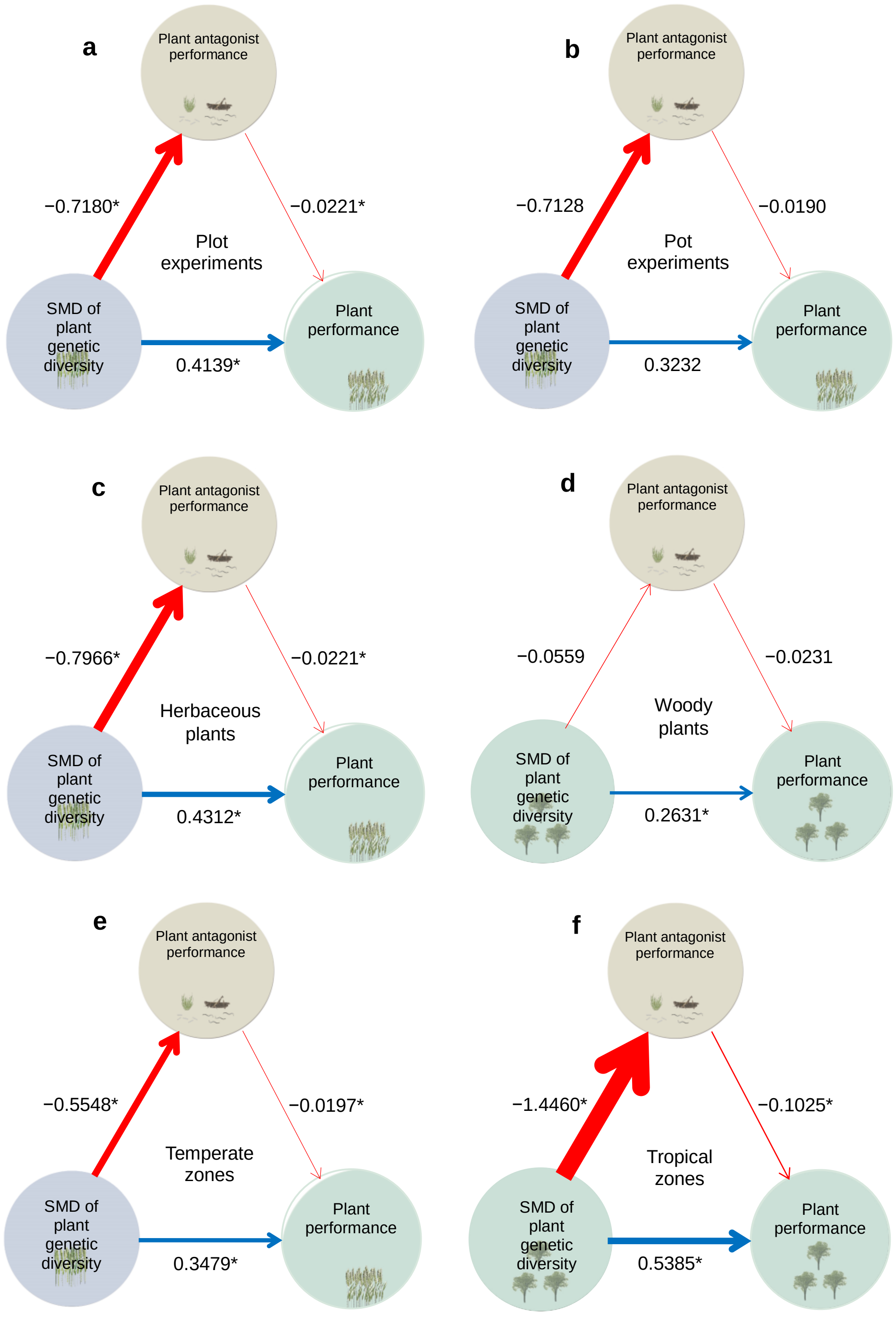

Supplementary Fig. 20 | Path analysis for the effects of SMD of plant genetic diversity on trophic interactions of plant antagonist and plant performance. SMD is the abbreviation of Standardized Mean Difference. **a,** plot experiments. **b,** pot experiments. **c,** herbaceous plants. **d,** woody plants. **e,** temperate zones. **f,** tropical zones. Plant antagonist performance including herbivore performance (abundance, damage and diversity of herbivores), weed performance (growth and diversity of weeds), nematode performance (nematode abundance), and disease performance (disease spread and damage) are shown in beige circles. Plant genetic diversity is shown in dusty blue. Plant performance (growth, quality and reproduction of plants) is shown in teal. The blue and red arrows denote positive and negative relationships respectively, numbers beside each arrow are the estimate coefficients for the fitted path-analytic models (Supplementary Tables 13, 14), and line width is proportional to the magnitude of the presented coefficient. The asterisks indicate significance at 5% level.

Supplementary Methods

Further description of study selection

For studies that included ≥ one location, we considered these experimental observations separately in each location and used the longitudes and latitudes of all locations in Fig.1, respectively. When the means of observed weed performance indicators (e.g., weed growth in one study), or the indicators for herbivores (e.g., herbivore damage in one study) and predators (e.g., predator abundance in one study) were not given in the study, we extracted these values directly from the figures (e.g. if a linear or a non-linear relationship between plant genetic diversity and one of these indicators was presented in a figure in the paper, we extracted the values from the fitting equations).

When the treatment group was paired with the control group, we excluded multiple comparisons within a single study, and we selected different comparison data (observations with pure or single plant genetic diversity were considered as control group while the others as the treatment group).

Further description of predictor variables

As predictor variables, we used six categorical variables and one continuous variable. **(1) Trophic group:** A categorical variable that denotes whether the organisms whose responses were studied were invertebrate herbivores (arthropod herbivores, amphipod herbivores and molluscan herbivores), natural enemies of invertebrate herbivores (invertebrate predators including carnivores of invertebrate herbivores, and parasitic wasps), weeds (harmful plants which have damage to ecosystems dominated by other plants or crops), plant-feeding nematodes (harmful nematodes which damage plants or crops), plant diseases (bacterial, fungal and virus pathogens which infest or infect plants or crops and cause damage to plants or crops), or primary producers (i.e., plants including grains, fruits, vegetables, shrubs, trees, grasses, eelgrass, seagrass, and epiphyte). To reformulate variables, we provide a comprehensive categorical variable of plant antagonists which included invertebrate herbivores, weeds, plant-feeding nematodes and plant disease. **(2) Response category:** We split the response variables into multiple categories nested within each trophic group. The *invertebrate herbivore group* included the abundance and diversity of herbivores, and herbivory damage. The *natural enemy group* included the abundance and diversity of invertebrate predators (predation was not found in this paper), and parasitoid abundance, diversity (species richness and Shannon's diversity) and parasitism. The *weed group* comprised weed growth and diversity (species richness and Shannon's diversity). The *plant-feeding nematode group* included only nematode abundance, and the *plant disease group* included disease spread and disease damage from bacterial, fungal and virus pathogens. *Disease spread* included the rates of disease development or progress rate per unit time, area under

a disease progress curve, plant virus disease frequency, disease incidence, the rate of increase of lesions, and spore output. Damage incurred by plant diseases was assessed as the percentage or number of disease severity and infection. *Plant performance* included plant growth, reproduction and quality. In the original studies, *invertebrates* (herbivores and their natural enemies) had been sampled with sweep nets, pitfall traps or pan traps around the focal plants, collected directly on the plant or plant parts (leaves, flower stalks, shoots, ramets, stems, tillers), and combined samples from direct counts of the visible invertebrates on a plant and from counting invertebrates that had dropped into trays below the plants after shaking or beating the plants. Damage incurred by herbivores and plant diseases was assessed as percentage or number of plants infested by herbivores and plant disease, or as loss of plant yield or biomass due to herbivores and plant disease. Plant growth and weed growth were assessed as growth rate and size characteristics (e.g., density, weight, height, biomass, dry matter, stem length, leaf area, leaf thickness and number of leaves). Here, we considered aboveground net primary productivity (ANPP) of plants as plant growth. *Plant reproduction* included the yield of grains, fruits, flowers, pods, reproductive plant parts and seeds per unit, and reproductive traits measured on individual plants (e.g., flower production, pod production and grain number per spike). *Plant quality* included the content of protein, gluten, detergent fiber, sucrose, starch and oil, the uptakes of nitrogen, phosphorus and potassium, milled rice milling rate (an index of product quality in rice), leaf chlorophyll concentration and chemical anti-herbivore defensive traits of plants (e.g. content of polyphenolics in stems). *Plant antagonist performance* was assessed by both plant antagonist intensity (including abundance of herbivores and nematodes, damage of herbivores and plant disease, plant disease spread and weed growth), and plant antagonist diversity. **(3) Ecosystem Type**: We categorized the ecosystems into agroecosystems, old-field ecosystems, marine ecosystems, grasslands, forests, shrublands and wetlands. Cited studies for each ecosystem type included in this paper should be more than 3 (Supplementary references). Agroecosystems could include crop ecosystems, ornamental plant plantation systems, orchards, etc. We abandoned the only one study for freshwater and dune ecosystem and only one study for dune ecosystem to reveal the effects of plant genetic diversity on trophic groups, respectively. According to the goals of the experiments in which they simulate such an ecosystem, we confirmed the type of ecosystem that a study addresses. **(4) Plant life form**: A categorical variable indicating whether the terrestrial or marine plants on or around which invertebrate herbivores and their natural enemies, weeds, nematodes and plant diseases were sampled, or for which growth, reproduction and quality were assessed are herbaceous or woody (Liao et al., 2008; Wan et al., 2020)[1, 2]. **(5) Climatic zone type:** A categorical variable indicating whether a certain study was carried out in the temperate or tropical zones (data from greenhouse and other indoor experiments have been removed from the models with climatic predictors). Temperate zones ranged from 23.5^o N to 66.5^o N and from 23.5^o S to 66.5^o S, and the tropical zones are from 0-23.5^o N and from 0-23.5^o S (Wan et al., 2020)[2].

(6) Type of experimental study: This variable includes plot experiments and pot experiments. Common garden experiments with a few or several replicated plots were considered as plot experiments. Field plot experiments in terrestrial ecosystems were involved in plot experiments. Also, experiments in aquatic ecosystems with a few or several replicated plots were also considered as plot experiment. Tray, box, tanker and container experiments were considered as pot experiments. Glasshouse experiments were integrated into plot or pot experiments. **(7) Number of added plant genotypes**: A continuous variable presenting the number of genotypes by which the number of plant genotypes was increased over the pure genotype (i.e. the number of genotypes added by manipulated plant genetical diversity in experimental designs, such as interplanting or undersowing, intercropping, mixed cropping, mixed planting). When we compared the genotypes of the control (pure or mono- genotype) with the ones of the treatment (higher genotypes, ≥2 genotypes), we confirmed that both the control and treatment were compared on a single plant species.

R code:

```
library(metafor)
library(Hmisc)
library(export)
library(V.PhyloMaker)
library(xlsx)
setwd("...")
########## loading data ########
Plant_antagonist_intensity<-read.csv("plant antagonist intensity.csv",header = TRUE)
Plant_antagonist_diversity<-read.csv("plant antagonist diversity.csv",header = TRUE)
TotalPlant_antagonist<-rbind(Plant_antagonist_intensity,Plant_antagonist_diversity)
Herbivore_abundance<-read.csv("herbivore abundance.csv",header = TRUE)
Herbivore_diversity<-read.csv("herbivore diversity.csv",header = TRUE)
Herbivore_damage<-read.csv("herbivore damage.csv",header = TRUE)
TotalHerbivore<-rbind(Herbivore_abundance,Herbivore_diversity,Herbivore_damage)
Predator_abundance<-read.csv("predator abundance.csv",header = TRUE)
Predator_diversity<-read.csv("predator diversity.csv",header = TRUE)
TotalPredator<-rbind(Predator_abundance,Predator_diversity)
Parasitoid_abundance<-read.csv("parasitoid abundance.csv",header = TRUE)
Parasitoid_diversity<-read.csv("parasitoid diversity.csv",header = TRUE)
Parasitism<-read.csv("parasitism.csv",header = TRUE)
TotalParasitoid<-rbind(Parasitoid_abundance,Parasitoid_diversity,Parasitism)
Weed_growth<-read.csv("weed growth.csv",header = TRUE)
Weed_diversity<-read.csv("weed diversity.csv",header = TRUE)
TotalWeed<-rbind(Weed_growth,Weed_diversity)
```

```
nematode_abundance<-read.csv("nematode abundance.csv",header = TRUE)
Disease_spread<-read.csv("disease spread.csv",header = TRUE)
Disease_damage<-read.csv("disease damage.csv",header = TRUE)
TotalDisease<-rbind(Disease_spread,Disease_damage)
Plant_growth<-read.csv("plant growth.csv",header = TRUE)
Plant_quality<-read.csv("plant quality.csv",header = TRUE)
Plant_reproduction<-read.csv("plant reproduction.csv",header = TRUE)
TotalPlant<-rbind(Plant_growth,Plant_quality,Plant_reproduction)
enemy<-rbind(TotalPredator,TotalParasitoid)

######Tree and Phylogenies made######
myphylo=function(Total.data2){
  species=read.xlsx('plant species information-0713-wan-revised.xls',1)
  species=subset(species,species %in% unique(Total.data2$Plant.species))
  mycor=phylo.maker(species)
  mytree <- compute.brlen(mycor$scenario.3)
  A <- vcv(mytree, corr=TRUE)
  Total.data2$Plant.species=factor(Total.data2$Plant.species)
  Total.data2$Plant.species.new=Total.data2$Plant.species
  levels(Total.data2$Plant.species.new)=sort(dimnames(A)[[1]])
  Total.data2$Plant.species.new.p=Total.data2$Plant.species.new
  return(list(Total.data2,A))
}

########## Figures    ##########

# figure 1b plot
fig1b_dat=list(TotalPlant_antagonist,TotalHerbivore,enemy,TotalWeed,nematode_abundance,
               TotalDisease,TotalPlant,Plant_antagonist_intensity,Plant_antagonist_diversity,
               Herbivore_abundance,Herbivore_damage,Herbivore_diversity,
               Predator_abundance,Predator_diversity,Parasitoid_abundance,
               Parasitoid_diversity,Parasitism,Weed_growth,Weed_diversity,
               nematode_abundance,Disease_spread,Disease_damage,Plant_growth,
               Plant_quality,Plant_reproduction)

res=matrix(NA,length(fig1b_dat),3)
for (i in 1:length(fig1b_dat)) {
  res1=escalc(measure="SMD",n1i=Tn, n2i=Cn, m1i=T, m2i=C,
              sd1i=Tsd, sd2i=Csd,
              data=fig1b_dat[[i]],vtype = "UB",append = TRUE)
```

```
    if (length(unique(res1$Plant.species))==1) {
        res2=rma.mv(yi, vi,data = res1)
    } else {
        phylodat=myphylo(res1)
        res2=rma.mv(yi, vi,
                    random = list(~ 1 | Plant.species.new,~ 1|Plant.species.new.p),
                    R=list(Plant.species.new.p=phylodat[[2]]),
                    data = phylodat[[1]])
    }
    res[i,]=c(res2$b,res2$ci.lb,res2$ci.ub)
}

par(lwd=4)
par(mar=c(5, 4, 4, 8) + 0.1)
errbar(1:nrow(res),res[,1], res[,2], res[,3],ylim=c(-5,5),pch=19,
       xaxt="n",yaxt="n",ann = FALSE,lwd=4,cex=2,
       col=c(1:6,'orange',rep(1,2),rep(2,3),rep(3,5),rep(4,2),rep(5,1),rep(6,2),rep('orange',3)),
       errbar.col=c(1:6,'orange',rep(1,2),rep(2,3),rep(3,5),rep(4,2),rep(5,1),rep(6,2),rep('orange',3)))
axis(4,cex.axis=1.5)
axis(1,at=1:nrow(res),labels=FALSE)
mtext("Effect size",side=4,line=3,cex=1.5,family="sans") #Arial font
abline(h=0,lty=2)
graph2jpg(file='Fig 1(b)',height=11,width=12,dpi=300)



##########    Figures 4-7    ##########
Sfig_dat=list(Plant_antagonist_intensity,Plant_antagonist_diversity,
              Herbivore_abundance,Herbivore_damage,Herbivore_diversity,
              Predator_abundance,Predator_diversity,Parasitoid_abundance,
              Parasitoid_diversity,Parasitism,Weed_growth,Weed_diversity,
              nematode_abundance,Disease_spread,Disease_damage,Plant_growth,
              Plant_quality,Plant_reproduction)

# Fig. 4
res=matrix(NA,length(Sfig_dat),3)
res_all=list()
subgroup=c('Agroecosystem','Grassland','Forest',
           'Old-field-ecosystem','Marine-ecosystem',
           'Wetland','Shrubland')
```

```
for (j in 1:length(subgroup)) {
  for (i in 1:length(Sfig_dat)) {
    if(nrow(subset(Sfig_dat[[i]],Ecosystem==subgroup[j]))<=1) {res[i,]=NA}
    else {
      res1=escalc(measure="SMD",n1i=Tn, n2i=Cn, m1i=T, m2i=C,
                  sd1i=Tsd, sd2i=Csd,data=Sfig_dat[[i]],
                  subset=(Ecosystem==subgroup[j]),
                  vtype = "UB",append = TRUE)
      if (length(unique(res1$Plant.species))==1) {
        res2=rma.mv(yi, vi,data = res1)
      } else {
        phylodat=myphylo(res1)
        res2=rma.mv(yi, vi,
                    random = list(~ 1 | Plant.species.new,~ 1|Plant.species.new.p),
                    R=list(Plant.species.new.p=phylodat[[2]]),
                    data = phylodat[[1]])
      }
      res[i,]=c(res2$b,res2$ci.lb,res2$ci.ub)
    }
  }
  res_all[[j]]=res
}

res_Agro=res_all[[1]]
res_Grass=res_all[[2]]
res_Forest=res_all[[3]]
res_old=res_all[[4]]
res_Marine=res_all[[5]]
res_Wet=res_all[[6]]
res_Shrub=res_all[[7]]

par(lwd=4)
par(mar=c(5, 4, 4, 8) + 0.1)
errbar(1:nrow(res_Agro),res_Agro[,1], res_Agro[,2], res_Agro[,3],
       ylim=c(-5,5),pch=19,xaxt="n",yaxt="n",ann = FALSE,lwd=4,cex=2,
       col=c(rep(1,2),rep(2,3),rep(3,5),rep(4,2),5,rep(6,2),rep('orange',3)),
       errbar.col=c(rep(1,2),rep(2,3),rep(3,5),rep(4,2),5,rep(6,2),rep('orange',3)))
axis(4,cex.axis=1.5)
axis(1,at=1:nrow(res_Agro),labels=FALSE)
mtext("Effect size",side=4,line=3,cex=1.5,family="sans") #Arial font
```

```
abline(h=0,lty=2)
graph2jpg(file='Supplementary Fig 4(a)_Agroecosystems',height=10,width=8,dpi=300)

par(lwd=4)
par(mar=c(5, 4, 4, 8) + 0.1)
errbar(1:nrow(res_Grass),res_Grass[,1], res_Grass[,2], res_Grass[,3],
       ylim=c(-3,3),pch=19,xaxt="n",yaxt="n",ann = FALSE,lwd=4,cex=2,
       col=c(rep(1,2),rep(2,3),rep(3,5),rep(4,2),5,rep(6,2),rep('orange',3)),
       errbar.col=c(rep(1,2),rep(2,3),rep(3,5),rep(4,2),5,rep(6,2),rep('orange',3)))
axis(4,cex.axis=1.5)
axis(1,at=1:nrow(res_Grass),labels=FALSE)
mtext("Effect size",side=4,line=3,cex=1.5,family="sans") #Arial font
abline(h=0,lty=2)
graph2jpg(file='Supplementary Fig 4(b)_Grasslands',height=10,width=8,dpi=300)

par(lwd=4)
par(mar=c(5, 4, 4, 8) + 0.1)
errbar(1:nrow(res_Forest),res_Forest[,1], res_Forest[,2], res_Forest[,3],
       ylim=c(-1.5,1.5),pch=19,xaxt="n",yaxt="n",ann = FALSE,lwd=4,cex=2,
       col=c(rep(1,2),rep(2,3),rep(3,5),rep(4,2),5,rep(6,2),rep('orange',3)),
       errbar.col=c(rep(1,2),rep(2,3),rep(3,5),rep(4,2),5,rep(6,2),rep('orange',3)))
axis(4,cex.axis=1.5)
axis(1,at=1:nrow(res_Forest),labels=FALSE)
mtext("Effect size",side=4,line=3,cex=1.5,family="sans") #Arial font
abline(h=0,lty=2)
graph2jpg(file='Supplementary Fig 4(c)_Forests',height=10,width=8,dpi=300)

par(lwd=4)
par(mar=c(5, 4, 4, 8) + 0.1)
errbar(1:nrow(res_old),res_old[,1], res_old[,2], res_old[,3],
       ylim=c(-4.5,4.5),pch=19,xaxt="n",yaxt="n",ann = FALSE,lwd=4,cex=2,
       col=c(rep(1,2),rep(2,3),rep(3,5),rep(4,2),5,rep(6,2),rep('orange',3)),
       errbar.col=c(rep(1,2),rep(2,3),rep(3,5),rep(4,2),5,rep(6,2),rep('orange',3)))
axis(4,cex.axis=1.5)
axis(1,at=1:nrow(res_old),labels=FALSE)
mtext("Effect size",side=4,line=3,cex=1.5,family="sans") #Arial font
abline(h=0,lty=2)
graph2jpg(file='Supplementary Fig 4_Old-field-ecosystems',height=10,width=8,dpi=300)

par(lwd=4)
```

```
par(mar=c(5, 4, 4, 8) + 0.1)
errbar(1:nrow(res_Marine),res_Marine[,1], res_Marine[,2], res_Marine[,3],
        ylim=c(-1.5,1.5),pch=19,xaxt="n",yaxt="n",ann = FALSE,lwd=4,cex=2,
        col=c(rep(1,2),rep(2,3),rep(3,5),rep(4,2),5,rep(6,2),rep('orange',3)),
        errbar.col=c(rep(1,2),rep(2,3),rep(3,5),rep(4,2),5,rep(6,2),rep('orange',3)))
axis(4,cex.axis=1.5)
axis(1,at=1:nrow(res_Marine),labels=FALSE)
mtext("Effect size",side=4,line=3,cex=1.5,family="sans") #Arial font
abline(h=0,lty=2)
graph2jpg(file='Supplementary Fig 4(e)_Marine-ecosystems',height=10,width=8,dpi=300)

par(lwd=4)
par(mar=c(5, 4, 4, 8) + 0.1)
errbar(1:nrow(res_Wet),res_Wet[,1], res_Wet[,2], res_Wet[,3],
        ylim=c(-1.5,1.5),pch=19,xaxt="n",yaxt="n",ann = FALSE,lwd=4,cex=2,
        col=c(rep(1,2),rep(2,3),rep(3,5),rep(4,2),5,rep(6,2),rep('orange',3)),
        errbar.col=c(rep(1,2),rep(2,3),rep(3,5),rep(4,2),5,rep(6,2),rep('orange',3)))
axis(4,cex.axis=1.5)
axis(1,at=1:nrow(res_Wet),labels=FALSE)
mtext("Effect size",side=4,line=3,cex=1.5,family="sans") #Arial font
abline(h=0,lty=2)
graph2jpg(file='Supplementary Fig 4(f)_Wetlands',height=10,width=8,dpi=300)

par(lwd=4)
par(mar=c(5, 4, 4, 8) + 0.1)
errbar(1:nrow(res_Shrub),res_Shrub[,1], res_Shrub[,2], res_Shrub[,3],
        ylim=c(-2,2),pch=19,xaxt="n",yaxt="n",ann = FALSE,lwd=4,cex=2,
        col=c(rep(1,2),rep(2,3),rep(3,5),rep(4,2),5,rep(6,2),rep('orange',3)),
        errbar.col=c(rep(1,2),rep(2,3),rep(3,5),rep(4,2),5,rep(6,2),rep('orange',3)))
axis(4,cex.axis=1.5)
axis(1,at=1:nrow(res_Shrub),labels=FALSE)
mtext("Effect size",side=4,line=3,cex=1.5,family="sans") #Arial font
abline(h=0,lty=2)
graph2jpg(file='Supplementary Fig 4(g)_Shrublands',height=10,width=8,dpi=300)

# Fig. 5
res=matrix(NA,length(Sfig_dat),3)
res_all=list()
subgroup=c('Plot-experiment','Pot-experiment')
for (j in 1:length(subgroup)) {
```

```
    for (i in 1:length(Sfig_dat)) {
        if(nrow(subset(Sfig_dat[[i]],Study.type==subgroup[j]))<=1) {res[i,]=NA}
        else {
            res1=escalc(measure="SMD",n1i=Tn, n2i=Cn, m1i=T, m2i=C,
                        sd1i=Tsd, sd2i=Csd,data=Sfig_dat[[i]],
                        subset=(Study.type==subgroup[j]),
                        vtype = "UB",append = TRUE)
            if (length(unique(res1$Plant.species))==1) {
                res2=rma.mv(yi, vi,data = res1)
            } else {
                phylodat=myphylo(res1)
                res2=rma.mv(yi, vi,
                            random = list(~ 1 | Plant.species.new,~ 1|Plant.species.new.p),
                            R=list(Plant.species.new.p=phylodat[[2]]),
                            data = phylodat[[1]])
            }
            res[i,]=c(res2$b,res2$ci.lb,res2$ci.ub)
        }
    }
    res_all[[j]]=res
}

res_Plot=res_all[[1]]
res_Pot=res_all[[2]]

par(lwd=4)
par(mar=c(5, 4, 4, 8) + 0.1)
errbar(1:nrow(res_Plot),res_Plot[,1], res_Plot[,2], res_Plot[,3],
       ylim=c(-5,5),pch=19,xaxt="n",yaxt="n",ann = FALSE,lwd=4,cex=2,
       col=c(rep(1,2),rep(2,3),rep(3,5),rep(4,2),5,rep(6,2),rep('orange',3)),
       errbar.col=c(rep(1,2),rep(2,3),rep(3,5),rep(4,2),5,rep(6,2),rep('orange',3)))
axis(4,cex.axis=1.5)
axis(1,at=1:nrow(res_Plot),labels=FALSE)
mtext("Effect size",side=4,line=3,cex=1.5,family="sans") #Arial font
abline(h=0,lty=2)
graph2jpg(file='Supplementary Fig 5(a)_Plot-experiments',height=10,width=8,dpi=300)

par(lwd=4)
par(mar=c(5, 4, 4, 8) + 0.1)
errbar(1:nrow(res_Pot),res_Pot[,1], res_Pot[,2], res_Pot[,3],
```

```
              ylim=c(-7,7),pch=19,xaxt="n",yaxt="n",ann = FALSE,lwd=4,cex=2,
              col=c(rep(1,2),rep(2,3),rep(3,5),rep(4,2),5,rep(6,2),rep('orange',3)),
              errbar.col=c(rep(1,2),rep(2,3),rep(3,5),rep(4,2),5,rep(6,2),rep('orange',3)))
axis(4,cex.axis=1.5)
axis(1,at=1:nrow(res_Pot),labels=FALSE)
mtext("Effect size",side=4,line=3,cex=1.5,family="sans") #Arial font
abline(h=0,lty=2)
graph2jpg(file='Supplementary Fig 5(b)_Pot-experiments',height=10,width=8,dpi=300)

# Fig. 6
res=matrix(NA,length(Sfig_dat),3)
res_all=list()
subgroup=c('Herbaceous-plant','Woody-plant')
for (j in 1:length(subgroup)) {
    for (i in 1:length(Sfig_dat)) {
        if(nrow(subset(Sfig_dat[[i]],Plant.type==subgroup[j]))<=1) {res[i,]=NA}
        else {
            res1=escalc(measure="SMD",n1i=Tn, n2i=Cn, m1i=T, m2i=C,
                            sd1i=Tsd, sd2i=Csd,data=Sfig_dat[[i]],
                            subset=(Plant.type==subgroup[j]),
                            vtype = "UB",append = TRUE)
            if (length(unique(res1$Plant.species))==1) {
                res2=rma.mv(yi, vi,data = res1)
            } else {
                phylodat=myphylo(res1)
                res2=rma.mv(yi, vi,
                                random = list(~ 1 | Plant.species.new,~ 1|Plant.species.new.p),
                                R=list(Plant.species.new.p=phylodat[[2]]),
                                data = phylodat[[1]])
            }
            res[i,]=c(res2$b,res2$ci.lb,res2$ci.ub)
        }
    }
    res_all[[j]]=res
}

res_Herba=res_all[[1]]
res_Woody=res_all[[2]]

par(lwd=4)
```

```
par(mar=c(5, 4, 4, 8) + 0.1)
errbar(1:nrow(res_Herba),res_Herba[,1], res_Herba[,2], res_Herba[,3],
       ylim=c(-5,5),pch=19,xaxt="n",yaxt="n",ann = FALSE,lwd=4,cex=2,
       col=c(rep(1,2),rep(2,3),rep(3,5),rep(4,2),5,rep(6,2),rep('orange',3)),
       errbar.col=c(rep(1,2),rep(2,3),rep(3,5),rep(4,2),5,rep(6,2),rep('orange',3)))
axis(4,cex.axis=1.5)
axis(1,at=1:nrow(res_Herba),labels=FALSE)
mtext("Effect size",side=4,line=3,cex=1.5,family="sans") #Arial font
abline(h=0,lty=2)
graph2jpg(file='Supplementary Fig 6(a)_Herbaceous-plants',height=10,width=8,dpi=300)

par(lwd=4)
par(mar=c(5, 4, 4, 8) + 0.1)
errbar(1:nrow(res_Woody),res_Woody[,1], res_Woody[,2], res_Woody[,3],
       ylim=c(-4,4),pch=19,xaxt="n",yaxt="n",ann = FALSE,lwd=4,cex=2,
       col=c(rep(1,2),rep(2,3),rep(3,5),rep(4,2),5,rep(6,2),rep('orange',3)),
       errbar.col=c(rep(1,2),rep(2,3),rep(3,5),rep(4,2),5,rep(6,2),rep('orange',3)))
axis(4,cex.axis=1.5)
axis(1,at=1:nrow(res_Woody),labels=FALSE)
mtext("Effect size",side=4,line=3,cex=1.5,family="sans") #Arial font
abline(h=0,lty=2)
graph2jpg(file='Supplementary Fig 6(b)_Woody-plants',height=10,width=8,dpi=300)

# Supplementary Fig. 7
res=matrix(NA,length(Sfig_dat),3)
res_all=list()
subgroup=c('Temperate','Tropical')
for (j in 1:length(subgroup)) {
  for (i in 1:length(Sfig_dat)) {
    if(nrow(subset(Sfig_dat[[i]],Biome==subgroup[j]))<=1) {res[i,]=NA}
    else {
      res1=escalc(measure="SMD",n1i=Tn, n2i=Cn, m1i=T, m2i=C,
                  sd1i=Tsd, sd2i=Csd,data=Sfig_dat[[i]],
                  subset=(Biome==subgroup[j]),
                  vtype = "UB",append = TRUE)
      res1=res1[res1$Outdoor..indoor.or.greenhouse.exp.=='Outdoor',]
      if (length(unique(res1$Plant.species))==1) {
        res2=rma.mv(yi, vi,data = res1)
      } else {
        phylodat=myphylo(res1)
```

```
            res2=rma.mv(yi, vi,
                        random = list(~ 1 | Plant.species.new,~ 1|Plant.species.new.p),
                        R=list(Plant.species.new.p=phylodat[[2]]),
                        data = phylodat[[1]])
          }
          res[i,]=c(res2$b,res2$ci.lb,res2$ci.ub)
        }
    }
    res_all[[j]]=res
}

res_Tem=res_all[[1]]
res_Tro=res_all[[2]]

par(lwd=4)
par(mar=c(5, 4, 4, 8) + 0.1)
errbar(1:nrow(res_Tem),res_Tem[,1], res_Tem[,2], res_Tem[,3],
       ylim=c(-8,8),pch=19,xaxt="n",yaxt="n",ann = FALSE,lwd=4,cex=2,
       col=c(rep(1,2),rep(2,3),rep(3,5),rep(4,2),5,rep(6,2),rep('orange',3)),
       errbar.col=c(rep(1,2),rep(2,3),rep(3,5),rep(4,2),5,rep(6,2),rep('orange',3)))
axis(4,cex.axis=1.5)
axis(1,at=1:nrow(res_Tem),labels=FALSE)
mtext("Effect size",side=4,line=3,cex=1.5,family="sans") #Arial font
abline(h=0,lty=2)
graph2jpg(file='Supplementary Fig 7(a)_Temperate zones',height=10,width=8,dpi=300)

par(lwd=4)
par(mar=c(5, 4, 4, 8) + 0.1)
errbar(1:nrow(res_Tro),res_Tro[,1], res_Tro[,2], res_Tro[,3],
       ylim=c(-4.5,4.5),pch=19,xaxt="n",yaxt="n",ann = FALSE,lwd=4,cex=2,
       col=c(rep(1,2),rep(2,3),rep(3,5),rep(4,2),5,rep(6,2),rep('orange',3)),
       errbar.col=c(rep(1,2),rep(2,3),rep(3,5),rep(4,2),5,rep(6,2),rep('orange',3)))
axis(4,cex.axis=1.5)
axis(1,at=1:nrow(res_Tro),labels=FALSE)
mtext("Effect size",side=4,line=3,cex=1.5,family="sans") #Arial font
abline(h=0,lty=2)
graph2jpg(file='Supplementary Fig 7(b)_Tropical zones',height=10,width=8,dpi=300)
```

Different established meta-regression models for model comparison and parameter evaluation

We performed meta-regression analysis using the R package metafor (version 2.4-0), in which we took Standardized Mean Difference (SMD) as the effect size measure (in the function “escalc()”, the argument “measure” was specified as “SMD”). Then, unbiased sample variance estimates were constructed to evaluate the variances of SMDs (in the function “escalc()”, the argument “vtype” was specified as “UB”). Supposing there are trophic groups (invertebrate herbivores, natural enemies of herbivores, weeds, plant-feeding nematodes, plant diseases and plants), newly integrated trophic groups (plant antagonists, herbivore natural enemies and plants) and different moderators (i.e. ecosystem types, types of experimental study, plant life forms, climatic zone types, and number of added plant genotypes), we employed a likelihood ratio test to compare the full model with the null model to investigate the significance of the interactive effects between trophic groups or integrated trophic groups and various moderators.

The null model corrsponded to the equation:

$$\beta_{ij} = \text{trophic groups } + r_i + w_j + \varepsilon_{ij} .$$

The full model corresponded to the equation:

$$\beta_{ij} = \text{trophic groups} + \text{moderators} + r_i + w_j + \varepsilon_{ij} ,$$

$$\beta_{ij} = \text{trophic groups} + \text{moderators} + \text{trophic groups} \times \text{moderators} + r_i + w_j + \varepsilon_{ij}$$

where β_{ij} is the jth effect size of study *i*, r_i represents the random effect of the plant species in ith study, and w_{ij} indicates the random effect of the phylogenetic relatedness of the plant species within jth study. In addition, ε_{ij} is the random error term with variance equal to the SMD’s sample variance estimate.

For examining the significant terms of the trophic groups, we compared the full model with the null model, and the full model was as follows:

$$\beta_{ij} = \text{trophic group} + \text{trophic group response categories} \times \text{moderators} + r_i + w_j + \varepsilon_{ij} ,$$

Note that we nested the trophic group response categories into trophic groups. Namely, invertebrate herbivore response categories (invertebrate herbivore abundance, herbivore damage, herbivore diversity) were nested in the invertebrate herbivore group; natural enemy group included natural enemy response categories (predator abundance, predator diversity, parasitoid abundance, parasitoid diversity and parasitism); weed response categories (weed growth and weed diversity) were nested within weed group; only plant-feeding nematode abundance response category were nested within plant-feeding nematode group; plant disease group encompassed plant disease response categories (plant disease spread and plant disease damage); plant response categories (plant growth, plant reproduction and plant quality) were nested within plant group. Additionally, response categories of plant antagonist intensity (abundance of herbivores and nematodes, damage of herbivores and plant disease, plant disease spread and weed growth), and plant antagonist diversity (weed diversity and herbivore diversity) were nested into plant antagonist group. So relevant response category effects would have an impact on the corresponding trophic group effect. In order to examine whether the full model had a better explanation than the null model, because there is overlap in evaluating the effect sizes of trophic groups and a series of

trophic group response categories, we took the predator abundance, parasitism, herbivore damage, and plant reproduction one by one in the full model, not estimating together. Furthermore, the likelihood-ratio-test (LRT) could be used to compare the full model which comprised the effects of the four trophic groups with the null model that remained nested in the full model. It is worth mentioning that the four removed response categories were assessed later via another alternatively-parametrized model without the four trophic group effects (see below).

We applied the LRT in comparing the null model with the full model for exploring the interactions between the trophic group response categories (invertebrate herbivore abundance, herbivore damage, herbivore diversity, predator abundance, predator diversity, parasitoid abundance, parasitoid diversity, parasitism, weed growth, weed diversity, plant-feeding nematode abundance, plant disease spread, plant disease damage, plant growth, plant reproduction, plant quality, plant antagonist intensity and plant antagonist diversity) and the different moderators. The null model was as follows:

$$\beta_{ij} = \text{trophic group response categories } + r_i + w_j + \varepsilon_{ij}.$$

The full model followed the equation:

$$\beta_{ij} = \text{trophic group response categories} + \text{trophic group response categories} \times \text{moderators} + r_i + w_j + \varepsilon_{ij},$$

$$\beta_{ij} = \text{trophic group response categories} + \text{moderators} + \\ \text{trophic group response categories} \times \text{moderators} + r_i + w_j + \varepsilon_{ij},$$

Subsequently, in order to estimate the effects and their corresponding confidence intervals of each trophic group, trophic group response category and their associations with the moderators, the following models were employed:

$$\beta_{ij} = \text{trophic groups } + r_i + w_j + \varepsilon_{ij},$$

$$\beta_{ij} = \text{trophic groups} + \text{moderators} + r_i + w_j + \varepsilon_{ij}$$

$$\beta_{ij} = \text{trophic groups} + \text{moderators} + \text{trophic groups} \times \text{moderators} + r_i + w_j + \varepsilon_{ij},$$

$$\beta_{ij} = \text{trophic group response categories } + r_i + w_j + \varepsilon_{ij},$$

$$\beta_{ij} = \text{trophic group response categories} + \text{moderators} + r_i + w_j + \varepsilon_{ij},$$

$$\beta_{ij} = \text{trophic group response categories} + \text{moderators} + \\ \text{trophic group response categories} \times \text{moderators} + r_i + w_j + \varepsilon_{ij}.$$

Where compared to the earlier models with interactions terms, the above models contained the main effect in terms of trophic groups and trophic group response categories.

R code:

```
library(metafor)
```

```
library(xlsx)
library(V.PhyloMaker)
library(taxize)

setwd("...")
########## loading data ########
plant_antagonist_intensity<-read.csv("plant antagonist intensity.csv",header = TRUE)
plant_antagonist_intensity$Trophic<-"plant antagonist"
plant_antagonist_intensity$Trophic.response<-"plant antagonist intensity"

plant_antagonist_diversity<-read.csv("plant antagonist diversity.csv",header = TRUE)
plant_antagonist_diversity$Trophic<-"plant antagonist"
plant_antagonist_diversity$Trophic.response<-"plant antagonist diversity"

Herbivore_abundance<-read.csv("herbivore abundance.csv",header = TRUE)
Herbivore_abundance$Trophic<-"Herbivore"
Herbivore_abundance$Trophic.response<-"Herbivore abundance"

Herbivore_diversity<-read.csv("herbivore diversity.csv",header = TRUE)
Herbivore_diversity$Trophic<-"Herbivore"
Herbivore_diversity$Trophic.response<-"Herbivore diversity"

Herbivore_damage<-read.csv("herbivore damage.csv",header = TRUE)
Herbivore_damage$Trophic<-"Herbivore"
Herbivore_damage$Trophic.response<-"Herbivore damage"

Predator_abundance<-read.csv("predator abundance.csv",header = TRUE)
Predator_abundance$Trophic<-"Enemy"
Predator_abundance$Trophic.response<-"Predator abundance"

Predator_diversity<-read.csv("predator diversity.csv",header = TRUE)
Predator_diversity$Trophic<-"Enemy"
Predator_diversity$Trophic.response<-"Predator diversity"

Parasitoid_abundance<-read.csv("parasitoid abundance.csv",header = TRUE)
Parasitoid_abundance$Trophic<-"Enemy"
Parasitoid_abundance$Trophic.response<-"Parasitoid abundance"

Parasitoid_diversity<-read.csv("parasitoid diversity.csv",header = TRUE)
Parasitoid_diversity$Trophic<-"Enemy"
```

```
Parasitoid_diversity$Trophic.response<-"Parasitoid diversity"

Parasitism<-read.csv("parasitism.csv",header = TRUE)
Parasitism$Trophic<-"Enemy"
Parasitism$Trophic.response<-"Parasitism"

Weed_growth<-read.csv("weed growth.csv",header = TRUE)
Weed_growth$Trophic<-"Weed"
Weed_growth$Trophic.response<-"Weed growth"

Weed_diversity<-read.csv("weed diversity.csv",header = TRUE)
Weed_diversity$Trophic<-"Weed"
Weed_diversity$Trophic.response<-"Weed diversity"

nematode_abundance<-read.csv("nematode abundance.csv",header = TRUE)
nematode_abundance$Trophic<-"Nematode"
nematode_abundance$Trophic.response<-"Nematode abundance"

Disease_spread<-read.csv("disease spread.csv",header = TRUE)
Disease_spread$Trophic<-"Disease"
Disease_spread$Trophic.response<-"Disease spread"

Disease_damage<-read.csv("disease damage.csv",header = TRUE)
Disease_damage$Trophic<-"Disease"
Disease_damage$Trophic.response<-"Disease damage"

Plant_growth<-read.csv("plant growth.csv",header = TRUE)
Plant_growth$Trophic<-"Plant"
Plant_growth$Trophic.response<-"Plant growth"

Plant_quality<-read.csv("plant quality.csv",header = TRUE)
Plant_quality$Trophic<-"Plant"
Plant_quality$Trophic.response<-"Plant quality"

Plant_reproduction<-read.csv("plant reproduction.csv",header = TRUE)
Plant_reproduction$Trophic<-"Plant"
Plant_reproduction$Trophic.response<-"Plant reproduction"

Total.data1<-rbind(Herbivore_abundance,Herbivore_diversity,Herbivore_damage,
                   Predator_abundance,Predator_diversity,
```

```
                    Parasitoid_abundance,Parasitoid_diversity,Parasitism,
                    Weed_growth,Weed_diversity,nematode_abundance,
                    Disease_spread,Disease_damage,
                    Plant_growth,Plant_reproduction,Plant_quality)

Total.data2<-rbind(plant_antagonist_intensity,plant_antagonist_diversity,
                    Predator_abundance,Predator_diversity,
                    Parasitoid_abundance,Parasitoid_diversity,Parasitism,
                    Plant_growth,Plant_reproduction,Plant_quality)

Total.data1$xi<-log2(Total.data1$No..genotypes.treat-Total.data1$No..genotypes.control)
Total.data2$xi<-log2(Total.data2$No..genotypes.treat-Total.data2$No..genotypes.control)

Total.data1=escalc(measure="SMD",n1i=Tn, n2i=Cn, m1i=T, m2i=C,
                    sd1i=Tsd, sd2i=Csd,
                    data=Total.data1,vtype = "UB",append = TRUE)
Total.data1=na.omit(Total.data1)
Total.data2=escalc(measure="SMD",n1i=Tn, n2i=Cn, m1i=T, m2i=C,
                     sd1i=Tsd, sd2i=Csd,
                     data=Total.data2,vtype = "UB",append = TRUE)
Total.data2=na.omit(Total.data2)

## fail-safe test
fsn_res<-fsn(yi,vi,data=Total.data1)

######Tree and Phylogenies made######
species=read.xlsx('plant species information-0713-wan-revised.xls',1)
mycor=phylo.maker(species)
mytree <- compute.brlen(mycor$scenario.3)
A <- vcv(mytree, corr=TRUE)
Total.data1$Plant.species=factor(Total.data1$Plant.species)
Total.data1$Plant.species.new=Total.data1$Plant.species
levels(Total.data1$Plant.species.new)=sort(dimnames(A)[[1]])
Total.data1$Plant.species.new.p=Total.data1$Plant.species.new

## supplementary table 1

# supplementary table 1(1)
modelis=list()
category=c('Ecosystem','Study.type','Plant.type','Biome','log2')
```

```
Total.data=Total.data1
# relatedness=sqrt(vcalc(vi, cluster=Plant.species, data=Total.data,nearpd=TRUE))
system.time(model1<-rma.mv(yi,vi,mods=~factor(Trophic)-1,
                           random = list(~ 1 | Plant.species.new,~ 1|Plant.species.new.p),
                           R=list(Plant.species.new.p=A),
                           data=Total.data,method="ML"))
S1_model1=matrix(NA,11,5)
for (i in 1:length(category)) {
  if(i<length(category)) {
    if(i==4) {
      Total.data4=Total.data[Total.data$Outdoor..indoor.or.greenhouse.exp.=='Outdoor',]
      #relatedness4=sqrt(vcalc(vi, cluster=Plant.species, data=Total.data4,nearpd=TRUE))
      modelB<-
rma.mv(yi,vi,mods=~factor(Trophic)+factor(Total.data4[,category[i]])+factor(Trophic):factor(Total.data4[,category[i]])
-1,
                     random = list(~ 1 | Plant.species.new,~ 1|Plant.species.new.p),
                     R=list(Plant.species.new.p=A),
                     data=Total.data4,
                     method="ML")
      modelA<-rma.mv(yi,vi,mods=~factor(Trophic)+factor(Total.data4[,category[i]])-1,
                     random = list(~ 1 | Plant.species.new,~ 1|Plant.species.new.p),
                     R=list(Plant.species.new.p=A),
                     data=Total.data4,
                     method="ML")
      model4<-rma.mv(yi,vi,mods=~factor(Trophic)-1,
                     random = list(~ 1 | Plant.species.new,~ 1|Plant.species.new.p),
                     R=list(Plant.species.new.p=A),
                     data=Total.data4,method="ML")
      A1=anova(modelA,model4)
      BA=anova(modelB,modelA)
    }
    else {
      modelB<-
rma.mv(yi,vi,mods=~factor(Trophic)+factor(Total.data[,category[i]])+factor(Trophic):factor(Total.data[,category[i]])-1,
                     random = list(~ 1 | Plant.species.new,~ 1|Plant.species.new.p),
                     R=list(Plant.species.new.p=A),
                     data=Total.data,method="ML")
      modelA<-rma.mv(yi,vi,mods=~factor(Trophic)+factor(Total.data[,category[i]])-1,
                     random = list(~ 1 | Plant.species.new,~ 1|Plant.species.new.p),
                     R=list(Plant.species.new.p=A),
```

```
                              data=Total.data,method="ML")
            A1=anova(modelA,model1)
            BA=anova(modelB,modelA)
          }
        }
    else{
        modelB<-rma.mv(yi,vi,mods=~factor(Trophic)+xi+factor(Trophic):xi-1,
                              random = list(~ 1 | Plant.species.new,~ 1|Plant.species.new.p),
                              R=list(Plant.species.new.p=A),
                              data=Total.data,method="ML")
        modelA<-rma.mv(yi,vi,mods=~factor(Trophic)+xi-1,
                              random = list(~ 1 | Plant.species.new,~ 1|Plant.species.new.p),
                              R=list(Plant.species.new.p=A),
                              data=Total.data,method="ML")
        A1=anova(modelA,model1)
        BA=anova(modelB,modelA)
    }
    modelis[[2*i-1]]=modelA
    modelis[[2*i]]=modelB
    S1_model1[1,]=c(A1[["fit.stats.r"]][["AIC"]],A1[["fit.stats.r"]][["ll"]],'-',A1[["parms.r"]],'-')
    S1_model1[2*i,]=c(A1[["fit.stats.f"]][["AIC"]],A1[["fit.stats.f"]][["ll"]],
                               A1[["LRT"]],A1[["parms.f"]],A1[["pval"]])
    S1_model1[2*i+1,]=c(BA[["fit.stats.f"]][["AIC"]],BA[["fit.stats.f"]][["ll"]],
                                 BA[["LRT"]],BA[["parms.f"]],BA[["pval"]])
}

model2<-rma.mv(yi,vi,mods=~factor(Trophic.response)-1,
                        random = list(~ 1 | Plant.species.new,~ 1|Plant.species.new.p),
                        R=list(Plant.species.new.p=A),
                        data=Total.data,method="ML")
S1_model2=matrix(NA,11,5)
aa=anova(model2,model1)
S1_model2[1,]=c(aa[["fit.stats.f"]][["AIC"]],aa[["fit.stats.f"]][["ll"]],
                         aa[["LRT"]],aa[["parms.f"]],aa[["pval"]])
for (i in 1:length(category)) {
    if(i<length(category)) {
        if(i==4) {
            Total.data4=Total.data[Total.data$Outdoor..indoor.or.greenhouse.exp.=='Outdoor',]
            # relatedness4=vcalc(vi, cluster=Plant.species, data=Total.data4,nearpd=TRUE)
            modelB<-
```

```
rma.mv(yi,vi,mods=~factor(Trophic.response)+factor(Total.data4[,category[i]])+factor(Trophic.response):factor(Total.d
ata4[,category[i]])-1,
                    random = list(~ 1 | Plant.species.new,~ 1|Plant.species.new.p),
                    R=list(Plant.species.new.p=A),
                    data=Total.data4,
                    method="ML")
      modelA<-rma.mv(yi,vi,mods=~factor(Trophic.response)+factor(Total.data4[,category[i]])-1,
                    random = list(~ 1 | Plant.species.new,~ 1|Plant.species.new.p),
                    R=list(Plant.species.new.p=A),
                    data=Total.data4,
                    method="ML")
      model4<-rma.mv(yi,vi,mods=~factor(Trophic.response)-1,
                    random = list(~ 1 | Plant.species.new,~ 1|Plant.species.new.p),
                    R=list(Plant.species.new.p=A),
                    data=Total.data4,method="ML")
      A1=anova(modelA,model4)
      BA=anova(modelB,modelA)
    }
    else {
      modelB<-
rma.mv(yi,vi,mods=~factor(Trophic.response)+factor(Total.data[,category[i]])+factor(Trophic.response):factor(Total.da
ta[,category[i]])-1,
                    random = list(~ 1 | Plant.species.new,~ 1|Plant.species.new.p),
                    R=list(Plant.species.new.p=A),
                    data=Total.data,method="ML")
      modelA<-rma.mv(yi,vi,mods=~factor(Trophic.response)+factor(Total.data[,category[i]])-1,
                    random = list(~ 1 | Plant.species.new,~ 1|Plant.species.new.p),
                    R=list(Plant.species.new.p=A),
                    data=Total.data,method="ML")
      A1=anova(modelA,model1)
      BA=anova(modelB,modelA)
    }
  }
  else{
    modelB<-rma.mv(yi,vi,mods=~factor(Trophic.response)+xi+factor(Trophic.response):xi-1,
                  random = list(~ 1 | Plant.species.new,~ 1|Plant.species.new.p),
                  R=list(Plant.species.new.p=A),
                  data=Total.data,method="ML")
    modelA<-rma.mv(yi,vi,mods=~factor(Trophic.response)+xi-1,
                  random = list(~ 1 | Plant.species.new,~ 1|Plant.species.new.p),
```

```
                              R=list(Plant.species.new.p=A),
                              data=Total.data,method="ML")
        A1=anova(modelA,model1)
        BA=anova(modelB,modelA)
    }
    modelis[[2*(i+5)-1]]=modelA
    modelis[[2*(i+5)]]=modelB
    S1_model2[2*i,]=c(A1[["fit.stats.f"]][["AIC"]],A1[["fit.stats.f"]][["ll"]],
                              A1[["LRT"]],A1[["parms.f"]],A1[["pval"]])
    S1_model2[2*i+1,]=c(BA[["fit.stats.f"]][["AIC"]],BA[["fit.stats.f"]][["ll"]],
                                BA[["LRT"]],BA[["parms.f"]],BA[["pval"]])
}
Ref=c('-',rep(c(1,"A"),5),1,rep(c(2,"A"),5))
Predictor_category=c('Trophic group + ecosystem type',
                              'Trophic group × ecosystem type',
                              'Trophic group + type of experimental study',
                              'Trophic group × type of experimental study',
                              'Trophic group + plant life form',
                              'Trophic group × plant life form',
                              'Trophic group + biome type',
                              'Trophic group × biome type',
                              'Trophic group + log2 (added plant genotypes over control)',
                              'Trophic group × log2 (added plant genotypes over control)')
Predictor=c('Trophic group',Predictor_category,
                    'Trophic group response category',Predictor_category)

n=c(model1[["k"]],unlist(lapply(modelis,function(x)x[["k"]]))[1:10],
        model2[["k"]],unlist(lapply(modelis,function(x)x[["k"]]))[11:20])
S1=cbind(Predictor,Ref,rbind(S1_model1,S1_model2),n)
colnames(S1)=c('Predictor','Ref','AIC','L-L','χ2','d.f.','P','n')
write.xlsx(S1,'Supplementary table 1(1).xls',row.names = F)
save.image(file = 'Supplementary Table 1(1).Rdata')

# supplementary table 1(2)
modelis=list()
category=c('Ecosystem','Study.type','Plant.type','Biome','log2')
Total.data=Total.data2
# relatedness=sqrt(vcalc(vi, cluster=Plant.species, data=Total.data,nearpd=TRUE))
system.time(model1<-rma.mv(yi,vi,mods=~factor(Trophic)-1,
                                    random = list(~ 1 | Plant.species.new,~ 1|Plant.species.new.p),
```

```
                                R=list(Plant.species.new.p=A),
                                data=Total.data,method="ML"))
S1_model1=matrix(NA,11,5)
for (i in 1:length(category)) {
  if(i<length(category)) {
    if(i==4) {
      Total.data4=Total.data[Total.data$Outdoor..indoor.or.greenhouse.exp.=='Outdoor',]
      #relatedness4=sqrt(vcalc(vi, cluster=Plant.species, data=Total.data4,nearpd=TRUE))
      modelB<-
rma.mv(yi,vi,mods=~factor(Trophic)+factor(Total.data4[,category[i]])+factor(Trophic):factor(Total.data4[,category[i]])
-1,
                          random = list(~ 1 | Plant.species.new,~ 1|Plant.species.new.p),
                          R=list(Plant.species.new.p=A),
                          data=Total.data4,
                          method="ML")
      modelA<-rma.mv(yi,vi,mods=~factor(Trophic)+factor(Total.data4[,category[i]])-1,
                          random = list(~ 1 | Plant.species.new,~ 1|Plant.species.new.p),
                          R=list(Plant.species.new.p=A),
                          data=Total.data4,
                          method="ML")
      model4<-rma.mv(yi,vi,mods=~factor(Trophic)-1,
                          random = list(~ 1 | Plant.species.new,~ 1|Plant.species.new.p),
                          R=list(Plant.species.new.p=A),
                          data=Total.data4,method="ML")
      A1=anova(modelA,model4)
      BA=anova(modelB,modelA)
    }
    else {
      modelB<-
rma.mv(yi,vi,mods=~factor(Trophic)+factor(Total.data[,category[i]])+factor(Trophic):factor(Total.data[,category[i]])-1,
                          random = list(~ 1 | Plant.species.new,~ 1|Plant.species.new.p),
                          R=list(Plant.species.new.p=A),
                          data=Total.data,method="ML")
      modelA<-rma.mv(yi,vi,mods=~factor(Trophic)+factor(Total.data[,category[i]])-1,
                          random = list(~ 1 | Plant.species.new,~ 1|Plant.species.new.p),
                          R=list(Plant.species.new.p=A),
                          data=Total.data,method="ML")
      A1=anova(modelA,model1)
      BA=anova(modelB,modelA)
    }
```

```
    }
    else{
        modelB<-rma.mv(yi,vi,mods=~factor(Trophic)+xi+factor(Trophic):xi-1,
                              random = list(~ 1 | Plant.species.new,~ 1|Plant.species.new.p),
                              R=list(Plant.species.new.p=A),
                              data=Total.data,method="ML")
        modelA<-rma.mv(yi,vi,mods=~factor(Trophic)+xi-1,
                              random = list(~ 1 | Plant.species.new,~ 1|Plant.species.new.p),
                              R=list(Plant.species.new.p=A),
                              data=Total.data,method="ML")
        A1=anova(modelA,model1)
        BA=anova(modelB,modelA)
    }
    modelis[[2*i-1]]=modelA
    modelis[[2*i]]=modelB
    S1_model1[1,]=c(A1[["fit.stats.r"]][["AIC"]],A1[["fit.stats.r"]][["ll"]],'-',A1[["parms.r"]],'-')
    S1_model1[2*i,]=c(A1[["fit.stats.f"]][["AIC"]],A1[["fit.stats.f"]][["ll"]],
                               A1[["LRT"]],A1[["parms.f"]],A1[["pval"]])
    S1_model1[2*i+1,]=c(BA[["fit.stats.f"]][["AIC"]],BA[["fit.stats.f"]][["ll"]],
                                 BA[["LRT"]],BA[["parms.f"]],BA[["pval"]])
}

model2<-rma.mv(yi,vi,mods=~factor(Trophic.response)-1,
                         random = list(~ 1 | Plant.species.new,~ 1|Plant.species.new.p),
                         R=list(Plant.species.new.p=A),
                         data=Total.data,method="ML")
S1_model2=matrix(NA,11,5)
aa=anova(model2,model1)
S1_model2[1,]=c(aa[["fit.stats.f"]][["AIC"]],aa[["fit.stats.f"]][["ll"]],
                         aa[["LRT"]],aa[["parms.f"]],aa[["pval"]])
for (i in 1:length(category)) {
    if(i<length(category)) {
        if(i==4) {
            Total.data4=Total.data[Total.data$Outdoor..indoor.or.greenhouse.exp.=='Outdoor',]
            # relatedness4=vcalc(vi, cluster=Plant.species, data=Total.data4,nearpd=TRUE)
            modelB<-
rma.mv(yi,vi,mods=~factor(Trophic.response)+factor(Total.data4[,category[i]])+factor(Trophic.response):factor(Total.d
ata4[,category[i]])-1,
                                   random = list(~ 1 | Plant.species.new,~ 1|Plant.species.new.p),
                                   R=list(Plant.species.new.p=A),
```

```
                              data=Total.data4,
                              method="ML")
      modelA<-rma.mv(yi,vi,mods=~factor(Trophic.response)+factor(Total.data4[,category[i]])-1,
                              random = list(~ 1 | Plant.species.new,~ 1|Plant.species.new.p),
                              R=list(Plant.species.new.p=A),
                              data=Total.data4,
                              method="ML")
      model4<-rma.mv(yi,vi,mods=~factor(Trophic.response)-1,
                              random = list(~ 1 | Plant.species.new,~ 1|Plant.species.new.p),
                              R=list(Plant.species.new.p=A),
                              data=Total.data4,method="ML")
      A1=anova(modelA,model4)
      BA=anova(modelB,modelA)
    }
    else {
      modelB<-
rma.mv(yi,vi,mods=~factor(Trophic.response)+factor(Total.data[,category[i]])+factor(Trophic.response):factor(Total.da
ta[,category[i]])-1,
                              random = list(~ 1 | Plant.species.new,~ 1|Plant.species.new.p),
                              R=list(Plant.species.new.p=A),
                              data=Total.data,method="ML")
      modelA<-rma.mv(yi,vi,mods=~factor(Trophic.response)+factor(Total.data[,category[i]])-1,
                              random = list(~ 1 | Plant.species.new,~ 1|Plant.species.new.p),
                              R=list(Plant.species.new.p=A),
                              data=Total.data,method="ML")
      A1=anova(modelA,model1)
      BA=anova(modelB,modelA)
    }
  }
  else{
    modelB<-rma.mv(yi,vi,mods=~factor(Trophic.response)+xi+factor(Trophic.response):xi-1,
                            random = list(~ 1 | Plant.species.new,~ 1|Plant.species.new.p),
                            R=list(Plant.species.new.p=A),
                            data=Total.data,method="ML")
    modelA<-rma.mv(yi,vi,mods=~factor(Trophic.response)+xi-1,
                            random = list(~ 1 | Plant.species.new,~ 1|Plant.species.new.p),
                            R=list(Plant.species.new.p=A),
                            data=Total.data,method="ML")
    A1=anova(modelA,model1)
    BA=anova(modelB,modelA)
```

```
    }
    modelis[[2*(i+5)-1]]=modelA
    modelis[[2*(i+5)]]=modelB
    S1_model2[2*i,]=c(A1[["fit.stats.f"]][["AIC"]],A1[["fit.stats.f"]][["ll"]],
                                  A1[["LRT"]],A1[["parms.f"]],A1[["pval"]])
    S1_model2[2*i+1,]=c(BA[["fit.stats.f"]][["AIC"]],BA[["fit.stats.f"]][["ll"]],
                                    BA[["LRT"]],BA[["parms.f"]],BA[["pval"]])
}
Ref=c('-',rep(c(1,"A"),5),1,rep(c(2,"A"),5))
Predictor_category=c('Trophic group + ecosystem type',
                                  'Trophic group × ecosystem type',
                                  'Trophic group + type of experimental study',
                                  'Trophic group × type of experimental study',
                                  'Trophic group + plant life form',
                                  'Trophic group × plant life form',
                                  'Trophic group + biome type',
                                  'Trophic group × biome type',
                                  'Trophic group + log2 (added plant genotypes over control)',
                                  'Trophic group × log2 (added plant genotypes over control)')
Predictor=c('Trophic group',Predictor_category,
                    'Trophic group response category',Predictor_category)

n=c(model1[["k"]],unlist(lapply(modelis,function(x)x[["k"]]))[1:10],
      model2[["k"]],unlist(lapply(modelis,function(x)x[["k"]]))[11:20])
S1=cbind(Predictor,Ref,rbind(S1_model1,S1_model2),n)
colnames(S1)=c('Predictor','Ref','AIC','L-L','χ2','d.f.','P','n')
write.xlsx(S1,'Supplementary table 1(2).xls',row.names = F)
save.image(file = 'Supplementary Table 1(2).Rdata')

## supplementary table 2

# supplementary table 2(1)
modelis=list()
category=c('Ecosystem','Study.type','Plant.type','Biome','log2')
Total.data=Total.data1
# relatedness=sqrt(vcalc(vi, cluster=Plant.species, data=Total.data,nearpd=TRUE))
system.time(model1<-rma.mv(yi,vi,mods=~factor(Trophic)-1+vi,
                                          random = list(~ 1 | Plant.species.new,~ 1|Plant.species.new.p),
                                          R=list(Plant.species.new.p=A),
                                          data=Total.data,method="ML"))
```

```
S2_model1=matrix(NA,11,2)
for (i in 1:length(category)) {
  if(i<length(category)) {
    if(i==4) {
      Total.data4=Total.data[Total.data$Outdoor..indoor.or.greenhouse.exp.=='Outdoor',]
      # relatedness4=sqrt(vcalc(vi, cluster=Plant.species, data=Total.data4,nearpd=TRUE))
      modelB<-
rma.mv(yi,vi,mods=~factor(Trophic)+factor(Total.data4[,category[i]])+factor(Trophic):factor(Total.data4[,category[i]])
-1+vi,
                     random = list(~ 1 | Plant.species.new,~ 1|Plant.species.new.p),
                     R=list(Plant.species.new.p=A),
                     data=Total.data4,
                     method="ML")
      modelA<-rma.mv(yi,vi,mods=~factor(Trophic)+factor(Total.data4[,category[i]])-1+vi,
                     random = list(~ 1 | Plant.species.new,~ 1|Plant.species.new.p),
                     R=list(Plant.species.new.p=A),
                     data=Total.data4,
                     method="ML")
    }
    else {
      modelB<-
rma.mv(yi,vi,mods=~factor(Trophic)+factor(Total.data[,category[i]])+factor(Trophic):factor(Total.data[,category[i]])-
1+vi,
                     random = list(~ 1 | Plant.species.new,~ 1|Plant.species.new.p),
                     R=list(Plant.species.new.p=A),
                     data=Total.data,method="ML")
      modelA<-rma.mv(yi,vi,mods=~factor(Trophic)+factor(Total.data[,category[i]])-1+vi,
                     random = list(~ 1 | Plant.species.new,~ 1|Plant.species.new.p),
                     R=list(Plant.species.new.p=A),
                     data=Total.data,method="ML")
    }
  }
  else{
    modelB<-rma.mv(yi,vi,mods=~factor(Trophic)+xi+factor(Trophic):xi-1+vi,
                   random = list(~ 1 | Plant.species.new,~ 1|Plant.species.new.p),
                   R=list(Plant.species.new.p=A),
                   data=Total.data,method="ML")
    modelA<-rma.mv(yi,vi,mods=~factor(Trophic)+xi-1+vi,
                   random = list(~ 1 | Plant.species.new,~ 1|Plant.species.new.p),
                   R=list(Plant.species.new.p=A),
```

```
                              data=Total.data,method="ML")
  }
  modelis[[2*i-1]]=modelA
  modelis[[2*i]]=modelB
  S2_model1[1,]=c(tail(model1[["zval"]],1),tail(model1[["pval"]],1))
  S2_model1[2*i,]=c(tail(modelA[["zval"]],1),tail(modelA[["pval"]],1))
  S2_model1[2*i+1,]=c(tail(modelB[["zval"]],1),tail(modelB[["pval"]],1))
}

model2<-rma.mv(yi,vi,mods=~factor(Trophic.response)-1+vi,
               random = list(~ 1 | Plant.species.new,~ 1|Plant.species.new.p),
               R=list(Plant.species.new.p=A),
               data=Total.data,method="ML")
S2_model2=matrix(NA,11,2)
S2_model2[1,]=c(tail(model2[["zval"]],1),tail(model2[["pval"]],1))
for (i in 1:length(category)) {
  if(i<length(category)) {
    if(i==4) {
      Total.data4=Total.data[Total.data$Outdoor..indoor.or.greenhouse.exp.=='Outdoor',]
      # relatedness4=vcalc(vi, cluster=Plant.species,data=Total.data4,nearpd=TRUE)
      modelB<-
rma.mv(yi,vi,mods=~factor(Trophic.response)+factor(Total.data4[,category[i]])+factor(Trophic.response):factor(Total.d
ata4[,category[i]])-1+vi,
                     random = list(~ 1 | Plant.species.new,~ 1|Plant.species.new.p),
                     R=list(Plant.species.new.p=A),
                     data=Total.data4,
                     method="ML")
      modelA<-rma.mv(yi,vi,mods=~factor(Trophic.response)+factor(Total.data4[,category[i]])-1+vi,
                     random = list(~ 1 | Plant.species.new,~ 1|Plant.species.new.p),
                     R=list(Plant.species.new.p=A),
                     data=Total.data4,
                     method="ML")
    }
    else {
      modelB<-
rma.mv(yi,vi,mods=~factor(Trophic.response)+factor(Total.data[,category[i]])+factor(Trophic.response):factor(Total.da
ta[,category[i]])-1+vi,
                     random = list(~ 1 | Plant.species.new,~ 1|Plant.species.new.p),
                     R=list(Plant.species.new.p=A),
                     data=Total.data,method="ML")
```

```
        modelA<-rma.mv(yi,vi,mods=~factor(Trophic.response)+factor(Total.data[,category[i]])-1+vi,
                       random = list(~ 1 | Plant.species.new,~ 1|Plant.species.new.p),
                       R=list(Plant.species.new.p=A),
                       data=Total.data,method="ML")
      }
    }
    else{
      modelB<-rma.mv(yi,vi,mods=~factor(Trophic.response)+xi+factor(Trophic.response):xi-1+vi,
                     random = list(~ 1 | Plant.species.new,~ 1|Plant.species.new.p),
                     R=list(Plant.species.new.p=A),
                     data=Total.data,method="ML")
      modelA<-rma.mv(yi,vi,mods=~factor(Trophic.response)+xi-1+vi,
                     random = list(~ 1 | Plant.species.new,~ 1|Plant.species.new.p),
                     R=list(Plant.species.new.p=A),
                     data=Total.data,method="ML")
    }
    modelis[[2*(i+5)-1]]=modelA
    modelis[[2*(i+5)]]=modelB
    S2_model2[2*i,]=c(tail(modelA[["zval"]],1),tail(modelA[["pval"]],1))
    S2_model2[2*i+1,]=c(tail(modelB[["zval"]],1),tail(modelB[["pval"]],1))
}
Ref=c('-',rep(c(1,"A"),5),1,rep(c(2,"A"),5))
Predictor_category=c('Trophic group + ecosystem type',
                     'Trophic group × ecosystem type',
                     'Trophic group + type of experimental study',
                     'Trophic group × type of experimental study',
                     'Trophic group + plant life form',
                     'Trophic group × plant life form',
                     'Trophic group + biome type',
                     'Trophic group × biome type',
                     'Trophic group + log2 (added plant genotypes over control)',
                     'Trophic group × log2 (added plant genotypes over control)')
Predictor=c('Trophic group',Predictor_category,
            'Trophic group response category',Predictor_category)

n=c(model1[["k"]],unlist(lapply(modelis,function(x)x[["k"]]))[1:10],
    model2[["k"]],unlist(lapply(modelis,function(x)x[["k"]]))[11:20])
S2=cbind(Predictor,Ref,rbind(S2_model1,S2_model2),n)
colnames(S2)=c('Predictor','Ref','Regression test value','P','n')
write.xlsx(S2,'Supplementary table 2(1).xls',row.names = F)
```

```
save.image(file = 'Supplementary Table 2(1).Rdata')

# supplementary table 2(2)
modelis=list()
category=c('Ecosystem','Study.type','Plant.type','Biome','log2')
Total.data=Total.data2
# relatedness=sqrt(vcalc(vi, cluster=Plant.species, data=Total.data,nearpd=TRUE))
system.time(model1<-rma.mv(yi,vi,mods=~factor(Trophic)-1+vi,
                           random = list(~ 1 | Plant.species.new,~ 1|Plant.species.new.p),
                           R=list(Plant.species.new.p=A),
                           data=Total.data,method="ML"))
S2_model1=matrix(NA,11,2)
for (i in 1:length(category)) {
  if(i<length(category)) {
    if(i==4) {
      Total.data4=Total.data[Total.data$Outdoor..indoor.or.greenhouse.exp.=='Outdoor',]
      # relatedness4=sqrt(vcalc(vi, cluster=Plant.species, data=Total.data4,nearpd=TRUE))
      modelB<-
rma.mv(yi,vi,mods=~factor(Trophic)+factor(Total.data4[,category[i]])+factor(Trophic):factor(Total.data4[,category[i]])
-1+vi,
                  random = list(~ 1 | Plant.species.new,~ 1|Plant.species.new.p),
                  R=list(Plant.species.new.p=A),
                  data=Total.data4,
                  method="ML")
      modelA<-rma.mv(yi,vi,mods=~factor(Trophic)+factor(Total.data4[,category[i]])-1+vi,
                  random = list(~ 1 | Plant.species.new,~ 1|Plant.species.new.p),
                  R=list(Plant.species.new.p=A),
                  data=Total.data4,
                  method="ML")
    }
    else {
      modelB<-
rma.mv(yi,vi,mods=~factor(Trophic)+factor(Total.data[,category[i]])+factor(Trophic):factor(Total.data[,category[i]])-
1+vi,
                  random = list(~ 1 | Plant.species.new,~ 1|Plant.species.new.p),
                  R=list(Plant.species.new.p=A),
                  data=Total.data,method="ML")
      modelA<-rma.mv(yi,vi,mods=~factor(Trophic)+factor(Total.data[,category[i]])-1+vi,
                  random = list(~ 1 | Plant.species.new,~ 1|Plant.species.new.p),
                  R=list(Plant.species.new.p=A),
```

```
                              data=Total.data,method="ML")
        }
    }
    else{
        modelB<-rma.mv(yi,vi,mods=~factor(Trophic)+xi+factor(Trophic):xi-1+vi,
                              random = list(~ 1 | Plant.species.new,~ 1|Plant.species.new.p),
                              R=list(Plant.species.new.p=A),
                              data=Total.data,method="ML")
        modelA<-rma.mv(yi,vi,mods=~factor(Trophic)+xi-1+vi,
                              random = list(~ 1 | Plant.species.new,~ 1|Plant.species.new.p),
                              R=list(Plant.species.new.p=A),
                              data=Total.data,method="ML")
    }
    modelis[[2*i-1]]=modelA
    modelis[[2*i]]=modelB
    S2_model1[1,]=c(tail(model1[["zval"]],1),tail(model1[["pval"]],1))
    S2_model1[2*i,]=c(tail(modelA[["zval"]],1),tail(modelA[["pval"]],1))
    S2_model1[2*i+1,]=c(tail(modelB[["zval"]],1),tail(modelB[["pval"]],1))
}

model2<-rma.mv(yi,vi,mods=~factor(Trophic.response)-1+vi,
                  random = list(~ 1 | Plant.species.new,~ 1|Plant.species.new.p),
                  R=list(Plant.species.new.p=A),
                  data=Total.data,method="ML")
S2_model2=matrix(NA,11,2)
S2_model2[1,]=c(tail(model2[["zval"]],1),tail(model2[["pval"]],1))
for (i in 1:length(category)) {
    if(i<length(category)) {
        if(i==4) {
            Total.data4=Total.data[Total.data$Outdoor..indoor.or.greenhouse.exp.=='Outdoor',]
            # relatedness4=vcalc(vi, cluster=Plant.species,data=Total.data4,nearpd=TRUE)
            modelB<-
rma.mv(yi,vi,mods=~factor(Trophic.response)+factor(Total.data4[,category[i]])+factor(Trophic.response):factor(Total.d
ata4[,category[i]])-1+vi,
                              random = list(~ 1 | Plant.species.new,~ 1|Plant.species.new.p),
                              R=list(Plant.species.new.p=A),
                              data=Total.data4,
                              method="ML")
            modelA<-rma.mv(yi,vi,mods=~factor(Trophic.response)+factor(Total.data4[,category[i]])-1+vi,
                              random = list(~ 1 | Plant.species.new,~ 1|Plant.species.new.p),
```

```
                                R=list(Plant.species.new.p=A),
                                data=Total.data4,
                                method="ML")
        }
        else {
            modelB<-
rma.mv(yi,vi,mods=~factor(Trophic.response)+factor(Total.data[,category[i]])+factor(Trophic.response):factor(Total.da
ta[,category[i]])-1+vi,
                                random = list(~ 1 | Plant.species.new,~ 1|Plant.species.new.p),
                                R=list(Plant.species.new.p=A),
                                data=Total.data,method="ML")
            modelA<-rma.mv(yi,vi,mods=~factor(Trophic.response)+factor(Total.data[,category[i]])-1+vi,
                                random = list(~ 1 | Plant.species.new,~ 1|Plant.species.new.p),
                                R=list(Plant.species.new.p=A),
                                data=Total.data,method="ML")
        }
    }
    else{
        modelB<-rma.mv(yi,vi,mods=~factor(Trophic.response)+xi+factor(Trophic.response):xi-1+vi,
                              random = list(~ 1 | Plant.species.new,~ 1|Plant.species.new.p),
                              R=list(Plant.species.new.p=A),
                              data=Total.data,method="ML")
        modelA<-rma.mv(yi,vi,mods=~factor(Trophic.response)+xi-1+vi,
                              random = list(~ 1 | Plant.species.new,~ 1|Plant.species.new.p),
                              R=list(Plant.species.new.p=A),
                              data=Total.data,method="ML")
    }
    modelis[[2*(i+5)-1]]=modelA
    modelis[[2*(i+5)]]=modelB
    S2_model2[2*i,]=c(tail(modelA[["zval"]],1),tail(modelA[["pval"]],1))
    S2_model2[2*i+1,]=c(tail(modelB[["zval"]],1),tail(modelB[["pval"]],1))
}
Ref=c('-',rep(c(1,"A"),5),1,rep(c(2,"A"),5))
Predictor_category=c('Trophic group + ecosystem type',
                                'Trophic group × ecosystem type',
                                'Trophic group + type of experimental study',
                                'Trophic group × type of experimental study',
                                'Trophic group + plant life form',
                                'Trophic group × plant life form',
                                'Trophic group + biome type',
```

```
                                'Trophic group × biome type',
                                'Trophic group + log2 (added plant genotypes over control)',
                                'Trophic group × log2 (added plant genotypes over control)')
Predictor=c('Trophic group',Predictor_category,
            'Trophic group response category',Predictor_category)

n=c(model1[["k"]],unlist(lapply(modelis,function(x)x[["k"]]))[1:10],
    model2[["k"]],unlist(lapply(modelis,function(x)x[["k"]]))[11:20])
S2=cbind(Predictor,Ref,rbind(S2_model1,S2_model2),n)
colnames(S2)=c('Predictor','Ref','Regression test value','P','n')
write.xlsx(S2,'Supplementary table 2(2).xls',row.names = F)
save.image(file = 'Supplementary Table 2(2).Rdata')
```

Relationship between log-transformed number of added genotypes in the plant genetic diversity treatment over the control and the effect sizes along with the fitted meta-regression line

To assess whether the number of added genotypes (relative genetic diversity) had additional explanatory power, we set up generalized least-squares models with the effect sizes with the fitted meta-regression line as the response variable, and the log-transformed number of added genotypes in the plant genetic diversity treatment over the control as an explanatory variable. Results from these models are displayed in Supplementary Figs. 1–14. The "gls()" function in the "nlme" library in R (Pinheiro & Bates, 2000; Pinheiro et al., 2021)[3,4] was employed, which fits a linear model using generalized least squares. The errors are allowed to be correlated or have unequal variances, implemented using an exponential variance function by setting "weights=varExp()" in the call to gls. We then employed the "Effect()" function in the "effects" library (Fox & Weisberg, 2019)[5] to calculate predicted values from the model object and a 95% confidence interval of the predictor considering unequal variances among observations with increasing values of the fitted values.

R code:

```
#Supplementary Fig 1-14 plot analysis
library(xlsx)
library(metafor)
library(export)
setwd("...")
########## loading data ########
plant_antagonist_intensity<-read.csv("plant antagonist intensity.csv",header = TRUE)
plant_antagonist_intensity$Trophic<-"plant antagonist"
plant_antagonist_intensity$Trophic.response<-"plant antagonist intensity"

plant_antagonist_diversity<-read.csv("plant antagonist diversity.csv",header = TRUE)
plant_antagonist_diversity$Trophic<-"plant antagonist"
```

```
plant_antagonist_diversity$Trophic.response<-"plant antagonist diversity"

Herbivore_abundance<-read.csv("herbivore abundance.csv",header = TRUE)
Herbivore_abundance$Trophic<-"Herbivore"
Herbivore_abundance$Trophic.response<-"Herbivore abundance"

Herbivore_diversity<-read.csv("herbivore diversity.csv",header = TRUE)
Herbivore_diversity$Trophic<-"Herbivore"
Herbivore_diversity$Trophic.response<-"Herbivore diversity"

Herbivore_damage<-read.csv("herbivore damage.csv",header = TRUE)
Herbivore_damage$Trophic<-"Herbivore"
Herbivore_damage$Trophic.response<-"Herbivore damage"

Predator_abundance<-read.csv("predator abundance.csv",header = TRUE)
Predator_abundance$Trophic<-"Enemy"
Predator_abundance$Trophic.response<-"Predator abundance"

Predator_diversity<-read.csv("predator diversity.csv",header = TRUE)
Predator_diversity$Trophic<-"Enemy"
Predator_diversity$Trophic.response<-"Predator diversity"

Parasitoid_abundance<-read.csv("parasitoid abundance.csv",header = TRUE)
Parasitoid_abundance$Trophic<-"Enemy"
Parasitoid_abundance$Trophic.response<-"Parasitoid abundance"

Parasitoid_diversity<-read.csv("parasitoid diversity.csv",header = TRUE)
Parasitoid_diversity$Trophic<-"Enemy"
Parasitoid_diversity$Trophic.response<-"Parasitoid diversity"

Parasitism<-read.csv("parasitism.csv",header = TRUE)
Parasitism$Trophic<-"Enemy"
Parasitism$Trophic.response<-"Parasitism"

Weed_growth<-read.csv("weed growth.csv",header = TRUE)
Weed_growth$Trophic<-"Weed"
Weed_growth$Trophic.response<-"Weed growth"

Weed_diversity<-read.csv("weed diversity.csv",header = TRUE)
Weed_diversity$Trophic<-"Weed"
```

```
Weed_diversity$Trophic.response<-"Weed diversity"

nematode_abundance<-read.csv("nematode abundance.csv",header = TRUE)
nematode_abundance$Trophic<-"Nematode"
nematode_abundance$Trophic.response<-"Nematode abundance"

Disease_spread<-read.csv("disease spread.csv",header = TRUE)
Disease_spread$Trophic<-"Disease"
Disease_spread$Trophic.response<-"Disease spread"

Disease_damage<-read.csv("disease damage.csv",header = TRUE)
Disease_damage$Trophic<-"Disease"
Disease_damage$Trophic.response<-"Disease damage"

Plant_growth<-read.csv("plant growth.csv",header = TRUE)
Plant_growth$Trophic<-"Plant"
Plant_growth$Trophic.response<-"Plant growth"

Plant_quality<-read.csv("plant quality.csv",header = TRUE)
Plant_quality$Trophic<-"Plant"
Plant_quality$Trophic.response<-"Plant quality"

Plant_reproduction<-read.csv("plant reproduction.csv",header = TRUE)
Plant_reproduction$Trophic<-"Plant"
Plant_reproduction$Trophic.response<-"Plant reproduction"

Total.data1<-rbind(Herbivore_abundance,Herbivore_diversity,Herbivore_damage,
                   Predator_abundance,Predator_diversity,
                   Parasitoid_abundance,Parasitoid_diversity,Parasitism,
                   Weed_growth,Weed_diversity,nematode_abundance,
                   Disease_spread,Disease_damage,
                   Plant_growth,Plant_reproduction,Plant_quality)

Total.data2<-rbind(plant_antagonist_intensity,plant_antagonist_diversity,
                   Predator_abundance,Predator_diversity,
                   Parasitoid_abundance,Parasitoid_diversity,Parasitism,
                   Plant_growth,Plant_reproduction,Plant_quality)

Total.data1$xi<-log2(Total.data1$No..genotypes.treat-Total.data1$No..genotypes.control)
Total.data2$xi<-log2(Total.data2$No..genotypes.treat-Total.data2$No..genotypes.control)
```

```
Total.data1=escalc(measure="SMD",n1i=Tn, n2i=Cn, m1i=T, m2i=C,
                   sd1i=Tsd, sd2i=Csd,
                   data=Total.data1,vtype = "UB",append = TRUE)
Total.data1=na.omit(Total.data1)
Total.data2=escalc(measure="SMD",n1i=Tn, n2i=Cn, m1i=T, m2i=C,
                   sd1i=Tsd, sd2i=Csd,
                   data=Total.data2,vtype = "UB",append = TRUE)
Total.data2=na.omit(Total.data2)
datlis=list(Total.data2[Total.data2$Trophic=='plant antagonist',],
            Total.data1[Total.data1$Trophic=='Herbivore',],
            Total.data1[Total.data1$Trophic=='Enemy',],
            Total.data1[Total.data1$Trophic=='Weed',],
            Total.data1[Total.data1$Trophic=='Nematode',],
            Total.data1[Total.data1$Trophic=='Disease',],
            Total.data1[Total.data1$Trophic=='Plant',])
require(nlme)
require(effects)

scatter_plot=function(datlis){
  columname=c('plant antagonist','herbivore','natural enemy','weed','nematode','disease','plant')
  number=c('a','b','c','d','e','f','g')
  par(mfrow=c(2,4),mar=c(4,4,2,2))

  for (i in 1:length(datlis)) {
    x <- datlis[[i]][,'xi']
    y <- datlis[[i]][,'yi']
    mydata=data.frame(x=x,y=y)

    if(length(unique(x))<=1){
      if(length(unique(x))==0) {
        plot(0,0,type = "n",xlab="Log2 (number of plant genotypes added over the control)",
             ylim=c(-20,20),las=1,ylab=paste0('Effect size of ',columname[i]," performance"),family="sans")
        text(-0.5,15,family="sans",number[i],cex = 1.5,font = 2)
      } else {
        plot(x,y,type = "p",xlab="Log2 (number of plant genotypes added over the control)",
             ylim=c(-20,20),las=1,ylab=paste0('Effect size of ',columname[i]," performance"),family="sans")
        text(max(x)-0.5,15,family="sans",number[i],cex = 1.5,font = 2)
      }
    } else {
```

```
            # for gls and Effect, mydata needs to be in global environment (renamed as "mydata2"):
            newx<-log2(seq(min(2^x),max(2^x),length=500))
            assign("mydata2", mydata, envir=.GlobalEnv)
            fit<-gls(y~x,weights=varExp(),data=mydata2)
            e1=Effect("x",fit,xlevels=list(x=newx))
            remove("mydata2", envir=.GlobalEnv) # remove from global environment

            P=signif(summary(fit)$tTable[2,4],4)
            if (P>0.05) {
              plot(x,y,type = "p",xlab="Log2 (number of plant genotypes added over the control)",
                   ylim=c(-20,20),las=1,ylab=paste0('Effect size of ',columname[i]," performance"),family="sans")
              text(min(x)+diff(range(x))*0.1,15,family="sans",number[i],cex = 1.5,font = 2)
            } else {
              pred.df=as.data.frame(e1)
              pred.c=pred.df[,c(2,4,5)]
              plot(x,y,type = "n",xlab="Log2 (number of plant genotypes added over the control)",
                   ylim=c(-20,20),las=1,ylab=paste0('Effect size of ',columname[i]," performance"),family="sans")
              #polygon(c(rev(newx), newx), c(rev(pred.p[ ,3]), pred.p[ ,2]),col = 'grey', border = NA)
              polygon(c(rev(newx), newx), c(rev(pred.c[ ,3]), pred.c[ ,2]),col = 'cornsilk4', border = NA)
              points(x, y,pch=22,cex=0.5)
              lines(x,fitted(fit),lwd=1)
              beta=round(summary(fit)$coef[2],3)
              intercept=round(summary(fit)$coef[1],3)
              intercept=ifelse(intercept<0,paste0('- ',abs(intercept)),paste0('+ ',intercept))
              text(min(x)+diff(range(x))*0.7,16,family="sans",paste0('Y = ',beta,'X ',intercept),cex = 1.2)
              text(min(x)+diff(range(x))*0.7,13,family="sans",paste0('(P=',P,')'),cex = 1.2)
              text(min(x)+diff(range(x))*0.1,15,family="sans",number[i],cex = 1.5,font = 2)
            }
         }
    }
}

########## Supplementary Fig. 1 ###########
scatter_plot(datlis)
graph2jpg(file='Supplementary Fig. 1',height=6,width=12,dpi=300)

########## Supplementary Fig. 2-8 #########
ecosystem=c('Agroecosystem','Grassland','Forest','Old-field-ecosystem',
                   'Marine-ecosystem','Wetland','Shrubland')
```

```
for (i in 1:length(ecosystem)) {
   dat_eco=lapply(1:length(datlis),function(x) {
                  datlis[[x]][datlis[[x]]$Ecosystem==ecosystem[i],]})
   scatter_plot(dat_eco)
   graph2jpg(file=paste0('Scatter_',ecosystem[i]),height=6,width=12,dpi=300)
}

########## Supplementary Fig. 9-10 #########
studytype=c('Plot-experiment','Pot-experiment')
for (i in 1:length(studytype)) {
   dat_study=lapply(1:length(datlis),function(x) {
      datlis[[x]][datlis[[x]]$Study.type==studytype[i],]})
   scatter_plot(dat_study)
   graph2jpg(file=paste0('Scatter_',studytype[i]),height=6,width=12,dpi=300)
}

########## Supplementary Fig. 11-12 #########
planttype=c('Herbaceous-plant','Woody-plant')
for (i in 1:length(planttype)) {
   dat_plant=lapply(1:length(datlis),function(x) {
      datlis[[x]][datlis[[x]]$Plant.type==planttype[i],]})
   scatter_plot(dat_plant)
   graph2jpg(file=paste0('Scatter_',planttype[i]),height=6,width=12,dpi=300)
}

########## Supplementary Fig. 13-14 #########
biome=c('Temperate','Tropical')
for (i in 1:length(biome)) {
   dat_biome=lapply(1:length(datlis),function(x) {
      subset(datlis[[x]],Biome==biome[i]&Outdoor..indoor.or.greenhouse.exp.=='Outdoor',)})
   scatter_plot(dat_biome)
   graph2jpg(file=paste0('Scatter_',biome[i]),height=6,width=12,dpi=300)
}

############### Supplementary Table 14 ##############
res=matrix(NA,length(datlis),4)
for (i in 1:length(datlis)) {
   if(i<7){
      res1=rma(yi, vi, data = datlis[[i]])
      res2=trimfill(res1,control=list(stepadj=0.5,maxiter=200))
```

```
        res[i,]=c(res2$k0,res2$b,res2$zval,res2$pval)
    }
else {
    res1=rma(yi, vi, data = datlis[[i]])
    res2=trimfill(res1,control=list(stepadj=0.1,maxiter=200))
    res[i,]=c(res2$k0,res2$b,res2$zval,res2$pval)
}
}
res<-as.data.frame(res,row.names=c('Pest','Herbivore','Enemy','Weed',
                                   'Nematode','Disease','Plant'))
colnames(res)<-c("Missing studies","Effect size","T-value","P-value")
write.xlsx(res,"Supplementary Table 14.xls")

#Supplemetary Table 16
require(nlme)
require(effects)

scatter_plot=function(EDF,datlis){
    columname=c('plant antagonist','herbivore','natural enemy','weed','nematode','disease','plant')
    number=c('a','b','c','d','e','f','g')
    res=matrix(NA,length(number),10)
    for (i in 1:length(datlis)) {
        x <- datlis[[i]][,'xi']
        y <- datlis[[i]][,'yi']
        mydata=data.frame(Code=datlis[[i]]$Code,x=x,y=y)

        if(length(unique(x))<=1){
            res[i,]=c(paste0(EDF,number[i]),nrow(mydata),length(unique(mydata$Code)),rep(NA,7))
        } else {

            # for gls and Effect, mydata needs to be in global environment (renamed as "mydata2"):
            newx<-log2(seq(min(2^x),max(2^x),length=500))
            assign("mydata2", mydata, envir=.GlobalEnv)
            fit<-gls(y~x,weights=varExp(),data=mydata2)
            e1=Effect("x",fit,xlevels=list(x=newx))
            remove("mydata2", envir=.GlobalEnv) # remove from global environment

            df=nrow(mydata)-2
            beta=round(summary(fit)$coef[2],3)
            intercept=round(summary(fit)$coef[1],3)
```

```
        intercept=ifelse(intercept<0,paste0('- ',abs(intercept)),paste0('+ ',intercept))
        Re=paste0('Y = ',beta,'X ',intercept)
        CI=signif(confint(fit, 'x', level=0.95),4)
        Parameter=signif(summary(fit)$tTable[2,2:4],4)
        res[i,]=c(paste0(EDF,number[i]),nrow(mydata),length(unique(mydata$Code)),
                  df,Re,CI,Parameter)
      }
  }
  colnames(res)=c('Figure Source','Number of observations','Number of Studies',
                  'd.f.','Regression equation','95%CL','95%CU',
                  'Std. Error','t-value','P-value')
  return(res)
}

########## Supplementary Fig. 1 ###########
EDF1=scatter_plot('Supplementary Fig. 1',datlis)

########## Supplementary Fig. 2-8 #########
ecosystem=c('Agroecosystem','Grassland','Forest','Old-field-ecosystem',
            'Marine-ecosystem','Wetland','Shrubland')
dat_eco=lapply(1:length(datlis),function(x) {
  datlis[[x]][datlis[[x]]$Ecosystem==ecosystem[1],]})
EDF2=scatter_plot('Supplementary Fig. 2',dat_eco)

dat_eco=lapply(1:length(datlis),function(x) {
  datlis[[x]][datlis[[x]]$Ecosystem==ecosystem[2],]})
EDF3=scatter_plot('Supplementary Fig. 3',dat_eco)

dat_eco=lapply(1:length(datlis),function(x) {
  datlis[[x]][datlis[[x]]$Ecosystem==ecosystem[3],]})
EDF4=scatter_plot('Supplementary Fig. 4',dat_eco)

dat_eco=lapply(1:length(datlis),function(x) {
  datlis[[x]][datlis[[x]]$Ecosystem==ecosystem[4],]})
EDF5=scatter_plot('Supplementary Fig. 5',dat_eco)

dat_eco=lapply(1:length(datlis),function(x) {
  datlis[[x]][datlis[[x]]$Ecosystem==ecosystem[5],]})
EDF6=scatter_plot('Supplementary Fig. 6',dat_eco)
```

```
dat_eco=lapply(1:length(datlis),function(x) {
  datlis[[x]][datlis[[x]]$Ecosystem==ecosystem[6],]})
EDF7=scatter_plot('Supplementary Fig. 7',dat_eco)

dat_eco=lapply(1:length(datlis),function(x) {
  datlis[[x]][datlis[[x]]$Ecosystem==ecosystem[7],]})
EDF8=scatter_plot('Supplementary Fig. 8',dat_eco)

########## Supplementary Fig. 9-10 #########
studytype=c('Plot-experiment','Pot-experiment')
dat_study=lapply(1:length(datlis),function(x) {
  datlis[[x]][datlis[[x]]$Study.type==studytype[1],]})
EDF9=scatter_plot('Supplementary Fig. 9',dat_study)

dat_study=lapply(1:length(datlis),function(x) {
  datlis[[x]][datlis[[x]]$Study.type==studytype[2],]})
EDF10=scatter_plot('Supplementary Fig. 10',dat_study)

########## Supplementary Fig. 11-12 #########
planttype=c('Herbaceous-plant','Woody-plant')
dat_plant=lapply(1:length(datlis),function(x) {
  datlis[[x]][datlis[[x]]$Plant.type==planttype[1],]})
EDF11=scatter_plot('Supplementary Fig. 11',dat_plant)

dat_plant=lapply(1:length(datlis),function(x) {
  datlis[[x]][datlis[[x]]$Plant.type==planttype[2],]})
EDF12=scatter_plot('Supplementary Fig. 12',dat_plant)

########## Supplementary Fig. 13-14 #########
biome=c('Temperate','Tropical')
dat_biome=lapply(1:length(datlis),function(x) {
  subset(datlis[[x]],Biome==biome[1]&Outdoor..indoor.or.greenhouse.exp.=='Outdoor',)})
EDF13=scatter_plot('Supplementary Fig. 13',dat_biome)

dat_biome=lapply(1:length(datlis),function(x) {
  subset(datlis[[x]],Biome==biome[2]&Outdoor..indoor.or.greenhouse.exp.=='Outdoor',)})
EDF14=scatter_plot('Supplementary Fig. 14',dat_biome)

S16=rbind(EDF1,EDF2,EDF3,EDF4,EDF5,EDF6,EDF7,EDF8,
          EDF9,EDF10,EDF11,EDF12,EDF13,EDF14)
```

```
write.xlsx(S16,"Supplementary Table 16.xls",row.names = F)
```

Meta-analytic structural equation modelling via piecewiseSEM

We set up piecewise structural equation models using the R package "piecewiseSEM". Because the "metafor" package cannot be integrated with "piecewiseSEM", we replaced the functions from metafor by calls to the "lme()" function in R package "nlme", which can reasonably evaluate the relevant results calculated by the "metafor" package by setting the arguments "control" and "weights" to "lmeControl(sigma=1)" and "varFixed(~ vi)", where "vi" denotes the estimated sample variance, which was calculated from the data using the "metafor" package. Such an approach is also advocated by the authors of the metafor package (https://www.metafor-project.org/doku.php/tips:rma_vs_lm_lme_lmer). Briefly, the main difference between mixed models fitted using rma() vs. lme() is that in lme models, the error variances are assumed to be known only up to a proportionality constant, while in rma() these variances are assumed to be exactly known. Overall, we decided to set up our piecewiseSEM models using combinations of linear mixed-effects models (lme), setting the control parameters and weights arguments as described above. Specifically, the following models were constructed as input to piecewiseSEM:

a) Plant genetic diversity / number of added plant genotypes– natural enemy performance – invertebrate herbivore performance in tri-trophic interactions (Fig. 3; Supplementary Fig. 15a)

a1. $$\begin{aligned}&\text{natural enemy performance SMD in tri-trophic interactions}\\&=\text{intercept}+\beta_{\text{enemy1}}\times\log_2(\text{the number of increased plant genetic diversity})+r_i+w_j+\varepsilon_{ij},\end{aligned}$$

a2. $$\begin{aligned}&\text{invertebrate herbivore performance SMD in tri-trophic interactions}\\&=\text{intercept}+\beta_{\text{enemy2}}\times\text{natural enemy performance SMD in tri-trophic interactions}+\\&\quad\beta_{\text{enemy3}}\times\log_2(\text{the number of increased plant genetic diversity})+r_i+w_j+\varepsilon_{ij}.\end{aligned}$$

b) Plant genetic diversity / number of added plant genotypes– invertebrate herbivore performance– plant performance in tri-trophic interactions (Fig. 3; Supplementary Fig. 15a)

b1. $$\begin{aligned}&\text{invertebrate herbivore performance SMD in tri-trophic interactions}\\&=\text{intercept}+\beta_{\text{herbivore1}}\times\log_2(\text{the number of increased plant genetic diversity})+r_i+w_j+\varepsilon_{ij},\end{aligned}$$

b2. $$\begin{aligned}&\text{plant performance SMD in tri-trophic interactions}\\&=\text{intercept}+\beta_{\text{herbivore2}}\times\text{invertebrate herbivore performance SMD in tri-trophic interactions}+\\&\quad\beta_{\text{herbivore3}}\times\log_2(\text{the number of increased plant genetic diversity})+r_i+w_j+\varepsilon_{ij}.\end{aligned}$$

c) Plant genetic diversity / number of added plant genotypes – natural enemy performance – invertebrate herbivore performance– plant performance in tri-trophic interactions (Fig. 3; Supplementary Fig. 15a)

c1. $$\text{natural enemy performance SMD in tri-trophic interactions} = \text{intercept} + \beta_{\text{enemy1}} \times \log_2(\text{the number of increased plant genetic diversity}) + r_i + w_j + \varepsilon_{ij},$$

c2. $$\text{invertebrate herbivore performance SMD in tri-trophic interactions} = \text{intercept} + \beta_{\text{enemy2}} \times \text{natural enemy performance SMD in tri-trophic interactions} + \beta_{\text{enemy3}} \times \log_2(\text{the number of increased plant genetic diversity}) + r_i + w_j + \varepsilon_{ij}.$$

c3. $$\text{plant performance SMD in tri-trophic interactions} = \text{intercept} + \beta_{\text{herbivore2}} \times \text{invertebrate herbivore performance SMD in tri-trophic interactions} + \beta_{\text{herbivore3}} \times \log_2(\text{the number of increased plant genetic diversity}) + r_i + w_j + \varepsilon_{ij}.$$

d) Plant genetic diversity / number of added plant genotypes– invertebrate herbivore performance– plant performance in bi-trophic interactions (Supplementary Figs. 15b and 18a)

d1. $$\text{invertebrate herbivore performance SMD in bi-trophic interactions} = \text{intercept} + \tilde{\beta}_{\text{herbivore1}} \times \log_2(\text{the number of increased plant genetic diversity}) + r_i + w_j + \varepsilon_{ij},$$

d2. $$\text{plant performance SMD in bi-trophic interactions} = \text{intercept} + \tilde{\beta}_{\text{herbivore2}} \times \text{invertebrate herbivore performance SMD in bi-trophic interactions} + \tilde{\beta}_{\text{herbivore3}} \times \log_2(\text{the number of increased plant genetic diversity}) + r_i + w_j + \varepsilon_{ij}.$$

e) Plant genetic diversity / number of added plant genotypes– weed performance– plant performance in bi-trophic interactions (Supplementary Figs. 15c and 18b)

e1. $$\text{weed performance SMD in bi-trophic interactions} = \text{intercept} + \beta_{\text{weed1}} \times \log_2(\text{the number of increased plant genetic diversity}) + r_i + w_j + \varepsilon_{ij},$$

e2. $$\text{plant performance SMD in bi-trophic interactions} = \text{intercept} + \beta_{\text{weed2}} \times \text{weed performance SMD in bi-trophic interactions} + \beta_{\text{weed3}} \times \log_2(\text{the number of increased plant genetic diversity}) + r_i + w_j + \varepsilon_{ij}.$$

f) Plant genetic diversity / number of added plant genotypes– plant-feeding nematode performance– plant performance in bi-trophic interactions (Supplementary Fig. 18c)

f1. $$\text{plant-feeding nematode performance SMD in bi-trophic interactions} = \text{intercept} + \beta_{\text{nematode1}} \times \log_2(\text{the number of increased plant genetic diversity}) + r_i + w_j + \varepsilon_{ij},$$

$$\text{plant performance SMD in bi-trophic interactions}$$

f2. $= \text{intercept} + \beta_{\text{nematode2}} \times \text{plant-feeding nematode performance SMD in bi-trophic interactions} + \beta_{\text{nematode3}} \times \log_2(\text{the number of increased plant genetic diversity}) + r_i + w_j + \varepsilon_{ij}.$

g) Plant genetic diversity / number of added plant genotypes– plant disease performance– plant performance in bi-trophic interactions (Supplementary Figs. 15d and 18d)

g1. $\text{plant disease performance SMD in bi-trophic interactions} = \text{intercept} + \beta_{\text{disease1}} \times \log_2(\text{the number of increased plant genetic diversity}) + r_i + w_j + \varepsilon_{ij},$

g2. $\text{plant performance SMD in bi-trophic interactions} = \text{intercept} + \beta_{\text{disease2}} \times \text{plant disease performance SMD in bi-trophic interactions} + \beta_{\text{disease3}} \times \log_2(\text{the number of increased plant genetic diversity}) + r_i + w_j + \varepsilon_{ij}.$

h) Plant genetic diversity / number of added plant genotypes– plant antagonist performance– plant performance in global ecosystems, agroecosystems, grasslands, forests, old-field ecosystems, marine ecosystems, wetlands and shrublands respectively in bi-trophic interactions (Supplementary Figs. 16a-f; Supplementary Figs. 19a-f)

h1. $\text{plant antagonists performance SMD in different ecosystems in bi-trophic interactions} = \text{intercept} + \beta_{\text{pest1}} \times \log_2(\text{the number of increased plant genetic diversity}) + r_i + w_j + \varepsilon_{ij},$

h2.

$$\text{plant performance SMD in different ecosystems in bi-trophic interactions} = \text{intercept} + \beta_{\text{pest2}} \times \text{plant antagonists performance SMD in different ecosystems in bi-trophic interactions} + \beta_{\text{pest3}} \times \log_2(\text{the number of increased plant genetic diversity}) + r_i + w_j + \varepsilon_{ij}.$$

i) Plant genetic diversity / number of added plant genotypes– plant antagonist performance– plant performance in plot experiments, pot experiments, herbaceous plants, woody plants, temperate climatic zones, and tropical climatic zones respectively in bi-trophic interactions (Supplementary Figs. 17a-f; Supplementary Figs. 20a-f)

i1. $\text{plant antagonists performance SMD in different subgroups in bi-trophic interactions} = \text{intercept} + \tilde{\beta}_{\text{pest1}} \times \log_2(\text{the number of increased plant genetic diversity}) + r_i + w_j + \varepsilon_{ij},$

i2.

$$\text{plant performance SMD in different subgroups in bi-trophic interactions} = \text{intercept} + \tilde{\beta}_{\text{pest2}} \times \text{plant antagonists performance SMD in different subgroups in bi-trophic interactions} + \tilde{\beta}_{\text{pest3}} \times \log_2(\text{the number of increased plant genetic diversity}) + r_i + w_j + \varepsilon_{ij}.$$

R code:

```
library(metafor)
library(piecewiseSEM)
library(nlme)
library(MuMIn)
library(xlsx)
library(V.PhyloMaker)
setwd("...")

########## fig 3, supplementary fig 19 and supplementary table 9 ##########
fig3=read.csv("enemy vs. herbivore vs. plant.csv",header = TRUE)
log_num_additional_species<-log2(fig3$No..genotypes.treat-fig3$No..genotypes.control)
SMD_enemy<-escalc(measure="SMD",n2i=enemy.Control.n,n1i=enemy.Treatment.n,
                  m2i=enemy.Control.value.,m1i=enemy.Treatment..value.,
                  sd2i=enemy.Control.sd,sd1i=enemy.Treatment.sd,
                  data=fig3,vtype = "UB",append = F)
SMD_herbivore<-escalc(measure="SMD",n2i=herbivore.Control.n, n1i=herbivore.Treat.n,
                      m2i=herbivore.Control.value., m1i=herbivore.Treatment..value.,
                      sd2i=herbivore.Control.sd, sd1i=herbivore.Treat.sd,
                      data=fig3,vtype = "UB",append = F)
SMD_plant<-escalc(measure="SMD",n2i=plant.Control.n, n1i=plant.Treat.n,
                  m2i=plant.Control.value., m1i=plant.Treatment..value.,
                  sd2i=plant.Control.sd, sd1i=plant.Treat.sd,
                  data=fig3,vtype = "UB",append = F)
fig3$log_num_additional_species<-log_num_additional_species
fig3$yi_SMD_enemy<-SMD_enemy$yi;fig3$vi_SMD_enemy<-SMD_enemy$vi
fig3$yi_SMD_herbivore<-SMD_herbivore$yi;fig3$vi_SMD_herbivore<-SMD_herbivore$vi
fig3$yi_SMD_plant<-SMD_plant$yi;fig3$vi_SMD_plant<-SMD_plant$vi

######Tree and Phylogenies made######
myphylo=function(Total.data2){
  species=read.xlsx('plant species information-0713-wan-revised.xls',1)
```

```
  species=subset(species,species %in% unique(Total.data2$Plant.species))
  mycor=phylo.maker(species)
  mytree <- compute.brlen(mycor$scenario.3)
  A <- vcv(mytree, corr=TRUE)
  Total.data2$Plant.species=factor(Total.data2$Plant.species)
  Total.data2$Plant.species.new=Total.data2$Plant.species
  levels(Total.data2$Plant.species.new)=sort(dimnames(A)[[1]])
  Total.data2$Plant.species.new.p=Total.data2$Plant.species.new
  return(list(Total.data2,A))
}

# figure 3
phylodat=myphylo(fig3)
res_enemy=rma.mv(yi_SMD_enemy, vi_SMD_enemy,
                 random = list(~ 1 | Plant.species.new,~ 1|Plant.species.new.p),
                 R=list(Plant.species.new.p=phylodat[[2]]),
                 data = phylodat[[1]])
res_herbivore=rma.mv(yi_SMD_herbivore, vi_SMD_herbivore,
                     random = list(~ 1 | Plant.species.new,~ 1|Plant.species.new.p),
                     R=list(Plant.species.new.p=phylodat[[2]]),
                     data = phylodat[[1]])
res_plant=rma.mv(yi_SMD_plant, vi_SMD_plant,
                 random = list(~ 1 | Plant.species.new,~ 1|Plant.species.new.p),
                 R=list(Plant.species.new.p=phylodat[[2]]),
                 data = phylodat[[1]])
model.list <- list(
  lme(yi_SMD_herbivore ~ yi_SMD_enemy,
      random = list(~ 1 | Plant.species,~ 1|Code),
      data = fig3,method = "REML",
      weights = varFixed(~ vi_SMD_herbivore), control = lmeControl(sigma = 1)),

  lme(yi_SMD_plant ~ yi_SMD_herbivore,
      random = list(~ 1 | Plant.species,~ 1|Code),
      data = fig3,method = "REML",
      weights = varFixed(~ vi_SMD_plant), control = lmeControl(sigma = 1))
)

psem_list <- as.psem(model.list)
fit <- summary(psem_list, .progressBar = FALSE)
fig3_res1=as.matrix(subset(fit$coefficients,select = -c(Crit.Value,DF,Std.Estimate))[,1:5])
```

```
fig3_res=matrix(c('Enemy performance','Herbivore performance','Plant performance',
                  rep('Plant genetic diversity',3),
                  res_enemy$b,res_herbivore$b,res_plant$b,
                  res_enemy$se,res_herbivore$se,res_plant$se,
                  res_enemy$pval,res_herbivore$pval,res_plant$pval),nrow = 3)
fig3_res=rbind(fig3_res[1,],fig3_res1[1,],fig3_res[2,],fig3_res1[2,],fig3_res[3,])
Trophic=rep('Plant genetic diversity on enemy vs. herbivore vs. plant',
            nrow(fig3_res))
fig3_res=cbind(Trophic,fig3_res)
write.xlsx(fig3_res,'fig3_results.xls',row.names = F)

# Supplementary Fig. 15a
model.list <- list(
  lme(yi_SMD_enemy ~ log_num_additional_species,
      random =list(~ 1 | Plant.species,~ 1|Code),
      data = fig3,
      weights = varFixed(~ vi_SMD_enemy), control = lmeControl(sigma = 1)),

  lme(yi_SMD_herbivore ~ yi_SMD_enemy + log_num_additional_species,
      random = list(~ 1 | Plant.species,~ 1|Code),
      data = fig3,
      weights = varFixed(~ vi_SMD_herbivore), control = lmeControl(sigma = 1)),

  lme(yi_SMD_plant ~ yi_SMD_herbivore + log_num_additional_species,
      random = list(~ 1 | Plant.species,~ 1|Code),
      data = fig3,
      weights = varFixed(~ vi_SMD_plant), control = lmeControl(sigma = 1))
)

psem_list <- as.psem(model.list)
fit <- summary(psem_list, .progressBar = FALSE)
Sfig15a_res=subset(fit$coefficients,select = -Crit.Value)
Trophic=rep('Number of added genotypes on enemy vs. herbivore vs. plant',
            nrow(Sfig15a_res))
Sfig15a_res=cbind(Trophic,Sfig15a_res)
write.xlsx(Sfig15a_res,'Supplementary Fig.15a_results.xls',row.names = F)

# Supplementary Fig. 18
fig18a=read.csv("herbivore vs. plant.csv",header = TRUE)
fig18b=read.csv("weed vs. plant.csv",header = TRUE)
```

```
fig18c=read.csv("nematode vs. plant.csv",header = TRUE)
fig18d=read.csv("disease vs. plant.csv",header = TRUE)
fig18lisdat=list(fig22a,fig22b,fig22c,fig22d)
fig18lisres=list()
category=c('herbivore','weed',
           'nematode','disease')
for (i in 1:length(fig18lisdat)) {
  SMD_notplant<-escalc(measure="SMD",n2i=fig18lisdat[[i]][,10], n1i=fig18lisdat[[i]][,13],
                       m2i=fig18lisdat[[i]][,8], m1i=fig18lisdat[[i]][,11],
                       sd2i=fig18lisdat[[i]][,9], sd1i=fig18lisdat[[i]][,12],
                       vtype = "UB",append = F)
  SMD_plant<-escalc(measure="SMD",n2i=plant.Control.n, n1i=plant.Treatment.n,
                    m2i=plant.Control..value., m1i=plant.Treatment..value.,
                    sd2i=plant.Control.sd, sd1i=plant.Treatment.sd,
                    data=fig18lisdat[[i]],vtype = "UB",append = F)
  fig18lisdat[[i]]$yi_SMD_notplant<-SMD_notplant$yi
  fig18lisdat[[i]]$vi_SMD_notplant<-SMD_notplant$vi
  fig18lisdat[[i]]$yi_SMD_plant<-SMD_plant$yi
  fig18lisdat[[i]]$vi_SMD_plant<-SMD_plant$vi
  fig18lisdat[[i]]$id=1:nrow(fig18lisdat[[i]])
  if (length(unique(fig18lisdat[[i]]$Plant.species))==1) {
    res_notplant=rma.mv(yi_SMD_notplant,vi_SMD_notplant,
                        data = fig18lisdat[[i]])
    res_plant=rma.mv(yi_SMD_plant,vi_SMD_plant,
                     data = fig18lisdat[[i]])
  } else {
    phylodat=myphylo(fig18lisdat[[i]])
    res_notplant=rma.mv(yi_SMD_notplant,vi_SMD_notplant,
                        random = list(~ 1 | Plant.species.new,~ 1|Plant.species.new.p),
                        R=list(Plant.species.new.p=phylodat[[2]]),
                        data = phylodat[[1]])
    res_plant=rma.mv(yi_SMD_plant,vi_SMD_plant,
                     random = list(~ 1 | Plant.species.new,~ 1|Plant.species.new.p),
                     R=list(Plant.species.new.p=phylodat[[2]]),
                     data = phylodat[[1]])
  }
  fig18lisdat[[i]]=na.omit(fig18lisdat[[i]])
  model.list <- list(
    lme(yi_SMD_plant ~ yi_SMD_notplant,
        random = list(~ 1 | Code, ~ 1 | Plant.species),
```

```
            data = fig18lisdat[[i]],
            weights = varFixed(~ vi_SMD_plant), control = lmeControl(sigma = 1))
  )
  psem_list <- as.psem(model.list)
  fit <- summary(psem_list, .progressBar = FALSE)
  fig18lisres1=as.matrix(subset(fit$coefficients,
                                select = -c(Crit.Value,DF,Std.Estimate))[,1:5])
  fig18lisres[[i]]=matrix(c(category[i],'Plant performance',
                            rep('Plant genetic diversity',2),
                            res_notplant$b,res_plant$b,
                            res_notplant$se,res_plant$se,
                            res_notplant$pval,res_plant$pval),nrow = 2)
  fig18lisres[[i]]=rbind(fig18lisres[[i]][1,],fig18lisres1,fig18lisres[[i]][2,])
  Trophic=rep(paste0('Plant genetic diversity on ',paste0(category[i],' vs. plant')),
              nrow(fig18lisres[[i]]))
  fig18lisres[[i]]=cbind(Trophic,fig18lisres[[i]])
}
write.xlsx(fig18lisres[[1]],'Supplementary Fig.18a_results.xls',row.names = F)
write.xlsx(fig18lisres[[2]],'Supplementary Fig.18b_results.xls',row.names = F)
write.xlsx(fig18lisres[[3]],'Supplementary Fig.18c_results.xls',row.names = F)
write.xlsx(fig18lisres[[4]],'Supplementary Fig.18d_results.xls',row.names = F)

# Supplementary Fig. 15b-d
Sfig15lisres=list()
for (i in 1:length(fig18lisdat)) {
  log_num_additional<-log2(fig18lisdat[[i]]$No..genotypes.treat-fig18lisdat[[i]]$No..genotypes.control)
  if(length(unique(log_num_additional))==1) {print(paste(category[i],'failed',sep=' '))
    Sfig15lisres[[i]]=cbind(paste0(category[i],' vs. plant'),NA)}
  else {
  fig18lisdat[[i]]$log_num_additional<-log_num_additional
  model.list <- list(
    lme(yi_SMD_notplant ~ log_num_additional,
        random = list(~ 1 | Code, ~ 1 | Plant.species),
        data = fig22lisdat[[i]],
        weights = varFixed(~ vi_SMD_notplant), control = lmeControl(sigma = 1)),

    lme(yi_SMD_plant ~ yi_SMD_notplant + log_num_additional,
        random = list(~ 1 | Code, ~ 1 | Plant.species),
        data = fig22lisdat[[i]],
        weights = varFixed(~ vi_SMD_plant), control = lmeControl(sigma = 1))
```

```
    )
    psem_list <- as.psem(model.list)
    fit <- summary(psem_list, .progressBar = FALSE)
    Sfig15lisres[[i]]=subset(fit$coefficients,select = -Crit.Value)
    Trophic=rep(paste0('Number of added genotypes on ',paste0(category[i],' vs. plant')),
                nrow(Sfig15lisres[[i]]))
    Sfig15lisres[[i]]=cbind(Trophic,Sfig15lisres[[i]])
    }
}
write.xlsx(Sfig15lisres[[1]],'Supplementary Fig.15b_results.xls',row.names = F)
write.xlsx(Sfig15lisres[[2]],'Supplementary Fig.15c_results.xls',row.names = F)
write.xlsx(Sfig15lisres[[4]],'Supplementary Fig.15e_results.xls',row.names = F)


# Supplementary Table 9
Observations=rep(nrow(fig3),nrow(fig3_res))
Studies=rep(length(unique(fig3[,1])),nrow(fig3_res))
fig3_S10=cbind(fig3_res[,c('Trophic','Predictor','Response')],Observations,
               Studies,fig3_res[,c('Estimate','Std.Error','P.Value')])
Sfig19a_S9=as.matrix(cbind(Sfig19a_res[,c('Trophic','Predictor','Response')],Observations,
                           Studies,Sfig19a_res[,c('Estimate','Std.Error','P.Value')]))
fig3_S9_list=list()
for (i in 1:length(fig22lisres)) {
    if(nrow(fig22lisres[[i]])<=1) {print(paste(category[i],'failed',sep=' '))
        fig3_S9_list[[i]]=NA}
    else {
        Observations=rep(nrow(fig22lisdat[[i]]),nrow(fig22lisres[[i]]))
        Studies=rep(length(unique(fig22lisdat[[i]][,1])),nrow(fig22lisres[[i]]))
        fig3_S9_list[[i]]=cbind(fig22lisres[[i]][,c('Trophic','Predictor','Response')],
                                Observations,Studies,
                                fig22lisres[[i]][,c('Estimate','Std.Error','P.Value')])
    }
}
Sfig19_S9_list=list()
for (i in 1:length(Sfig19lisres)) {
    if(nrow(Sfig19lisres[[i]])<=1) {print(paste(category[i],'failed',sep=' '))
        Sfig19_S9_list[[i]]=NA}
    else {
        Observations=rep(nrow(fig22lisdat[[i]]),nrow(fig22lisres[[i]]))
        Studies=rep(length(unique(fig22lisdat[[i]][,1])),nrow(fig22lisres[[i]]))
```

```
        Sfig19_S9_list[[i]]=as.matrix(cbind(Sfig19lisres[[i]][,c('Trophic','Predictor','Response')],
                                            Observations,Studies,
                                            Sfig19lisres[[i]][,c('Estimate','Std.Error','P.Value')]))
    }
}
S9_be=sapply(1:length(Sfig19_S9_list),
             function(x) {rbind(fig3_S9_list[[x]],Sfig19_S9_list[[x]])})
S9=rbind(fig3_S9,Sfig19a_S9,do.call('rbind',S9_be))
write.xlsx(S9,"Supplementary Table 9.xls",row.names = F)

########## Fig. 2, Supplementary Fig. 16, 19 and supplementary table 11 ##########
fig2=read.csv("plant antagonist vs. plant.csv",header = TRUE)
fig19a=subset(fig2,Ecosystem=='Agroecosystem')
fig19b=subset(fig2,Ecosystem=='Grassland')
fig19c=subset(fig2,Ecosystem=='Forest')
fig19d=subset(fig2,Ecosystem=='Old-field-ecosystem')
fig19e=subset(fig2,Ecosystem=='Marine-ecosystem')
fig19f=subset(fig2,Ecosystem=='Wetland')
fig19g=subset(fig2,Ecosystem=='Shrubland')
fig2lisdat=list(fig2,fig19a,fig19b,fig19c,fig19d,fig19e,fig19f,fig19g)

# fig 2 and Supplementary Fig. 19
fig2lisres=list()
category=c('global ecosystems','Agroecosystem','Grassland','Forest',
           'Old-field-ecosystem','Marine-ecosystem','Wetland','Shrubland')
for (i in 1:length(fig2lisdat)) {
    if(nrow(fig2lisdat[[i]])==0) {print(paste(category[i],'failed',sep=' '))
        fig2lisres[[i]]=cbind(category[i],NA)}
    else {
        SMD_pest<-escalc(measure="SMD",n2i=pest.Control.n, n1i=pest.Treatment.n,
                         m2i=pest.Control..value., m1i=pest.Treatment..value.,
                         sd2i=pest.Control.sd, sd1i=pest.Treatment.sd,
                         data=fig2lisdat[[i]],vtype = "UB",append = F)
        SMD_plant<-escalc(measure="SMD",n2i=plant.Control.n, n1i=plant.Treatment.n,
                          m2i=plant.Control..value., m1i=plant.Treatment..value.,
                          sd2i=plant.Control.sd, sd1i=plant.Treatment.sd,
                          data=fig2lisdat[[i]],vtype = "UB",append = F)
        fig2lisdat[[i]]$yi_SMD_pest<-SMD_pest$yi
        fig2lisdat[[i]]$vi_SMD_pest<-SMD_pest$vi
        fig2lisdat[[i]]$yi_SMD_plant<-SMD_plant$yi
```

```
fig2lisdat[[i]]$vi_SMD_plant<-SMD_plant$vi
fig2lisdat[[i]]$id=1:nrow(fig2lisdat[[i]])
if (length(unique(fig2lisdat[[i]]$Plant.species))==1) {
   res_pest=rma.mv(yi_SMD_pest,vi_SMD_pest,
                                    data = fig2lisdat[[i]])
   res_plant=rma.mv(yi_SMD_plant,vi_SMD_plant,
                               data = fig2lisdat[[i]])
} else {
   phylodat=myphylo(fig2lisdat[[i]])
   res_pest=rma.mv(yi_SMD_pest,vi_SMD_pest,
                                    random = list(~ 1 | Plant.species.new,~ 1|Plant.species.new.p),
                                    R=list(Plant.species.new.p=phylodat[[2]]),
                                    data = phylodat[[1]])
   res_plant=rma.mv(yi_SMD_plant,vi_SMD_plant,
                               random = list(~ 1 | Plant.species.new,~ 1|Plant.species.new.p),
                               R=list(Plant.species.new.p=phylodat[[2]]),
                               data = phylodat[[1]])
}
fig2lisdat[[i]]=na.omit(fig2lisdat[[i]])
if(i==3|i==4) {
   model.list <- list(
      lme(yi_SMD_plant ~ yi_SMD_pest,
            random = list(~ 1 | Code, ~ 1 | Plant.species),
            data = fig2lisdat[[i]],
            weights = varFixed(~ vi_SMD_plant), control = lmeControl(opt='optim',sigma = 1))
   )
}
else {
   model.list <- list(
      lme(yi_SMD_plant ~ yi_SMD_pest,
            random = list(~ 1 | Code, ~ 1 | Plant.species),
            data = fig2lisdat[[i]],
            weights = varFixed(~ vi_SMD_plant), control = lmeControl(sigma = 1))
   )
}
psem_list <- as.psem(model.list)
fit <- summary(psem_list, .progressBar = FALSE)
fig2lisres1=as.matrix(subset(fit$coefficients,
                                                select = -c(Crit.Value,DF,Std.Estimate))[,1:5])
fig2lisres[[i]]=matrix(c('Pest performance','Plant performance',
```

```
                                                rep('Plant genetic diversity',2),
                                                res_pest$b,res_plant$b,
                                                res_pest$se,res_plant$se,
                                                res_pest$pval,res_plant$pval),nrow = 2)
        fig2lisres[[i]]=rbind(fig2lisres[[i]][1,],fig2lisres1,fig2lisres[[i]][2,])
        Trophic=rep(paste0(category[i],'| Plant genetic diversity on ','pest vs. plant'),
                    nrow(fig2lisres[[i]]))
        fig2lisres[[i]]=cbind(Trophic,fig2lisres[[i]])
    }
}
write.xlsx(fig2lisres[[1]],'Fig.2_results.xls',row.names = F)
write.xlsx(fig2lisres[[2]],'Supplementary Fig.19a_results.xls',row.names = F)
write.xlsx(fig2lisres[[3]],'Supplementary Fig.19b_results.xls',row.names = F)
write.xlsx(fig2lisres[[4]],'Supplementary Fig.19c_results.xls',row.names = F)
write.xlsx(fig2lisres[[5]],'Supplementary Fig.19d_results.xls',row.names = F)
write.xlsx(fig2lisres[[6]],'Supplementary Fig.19e_results.xls',row.names = F)
write.xlsx(fig2lisres[[8]],'Supplementary Fig.19f_results.xls',row.names = F)

# Supplementary Fig. 16
Sfig16lisres=list()
for (i in 1:length(fig2lisdat)) {
    log_num_additional<-log2(fig2lisdat[[i]]$No..genotypes.treat-fig2lisdat[[i]]$No..genotypes.control)
    if(length(unique(log_num_additional))<=1) {print(paste(category[i],'failed',sep=' '))
        Sfig16lisres[[i]]=cbind(category[i],NA)}
    else {
        fig2lisdat[[i]]$log_num_additional<-log_num_additional
        if(i==1) {
            model.list <- list(
                lme(yi_SMD_pest ~ log_num_additional,
                        random = list(~ 1 | Code, ~ 1 | Plant.species),
                        data = fig2lisdat[[i]],
                        weights = varFixed(~ vi_SMD_pest), control = lmeControl(sigma = 1)),

                lme(yi_SMD_plant ~ yi_SMD_pest + log_num_additional,
                        random = list(~ 1 | Code, ~ 1 | Plant.species),
                        data = fig2lisdat[[i]],
                        weights = varFixed(~ vi_SMD_plant), control = lmeControl(opt='optim',sigma = 1))
            )
        }
        else {
```

```
        model.list <- list(
          lme(yi_SMD_pest ~ log_num_additional,
              random = list(~ 1 | Code, ~ 1 | Plant.species),
              data = fig2lisdat[[i]],
              weights = varFixed(~ vi_SMD_pest), control = lmeControl(sigma = 1)),

          lme(yi_SMD_plant ~ yi_SMD_pest + log_num_additional,
              random = list(~ 1 | Code, ~ 1 | Plant.species),
              data = fig2lisdat[[i]],
              weights = varFixed(~ vi_SMD_plant), control = lmeControl(sigma = 1))
        )
        }
      psem_list <- as.psem(model.list)
      fit <- summary(psem_list, .progressBar = FALSE)
      Sfig16lisres[[i]]=subset(fit$coefficients,select = -Crit.Value)
      Trophic=rep(paste0(category[i],'| Number of added genotypes on ',
                          'pest vs. plant'),nrow(Sfig16lisres[[i]]))
      Sfig16lisres[[i]]=cbind(Trophic,Sfig16lisres[[i]])
    }
}
write.csv(Sfig16lisres[[1]],'Supplementary Fig.16a_results.csv',row.names = F)
write.csv(Sfig16lisres[[2]],'Supplementary Fig.16b_results.csv',row.names = F)
write.csv(Sfig16lisres[[3]],'Supplementary Fig.16c_results.csv',row.names = F)
write.csv(Sfig16lisres[[4]],'Supplementary Fig.16d_results.csv',row.names = F)
write.csv(Sfig16lisres[[5]],'Supplementary Fig.16e_results.csv',row.names = F)
write.csv(Sfig16lisres[[6]],'Supplementary Fig.16f_results.csv',row.names = F)

# Supplementary Table 11
fig2_S11=list()
for (i in 1:length(fig2lisres)) {
  if(nrow(fig2lisres[[i]])<=1) {print(paste(category[i],'failed',sep=' '))
    fig2_S11[[i]]=NA}
  else {
    Observations=rep(nrow(fig2lisdat[[i]]),nrow(fig2lisres[[i]]))
    Studies=rep(length(unique(fig2lisdat[[i]][,1])),nrow(fig2lisres[[i]]))
    fig2_S11[[i]]=cbind(fig2lisres[[i]][,c('Trophic','Predictor','Response')],
                        Observations,Studies,
                        fig2lisres[[i]][,c('Estimate','Std.Error','P.Value')])
  }
}
```

```
Sfig20_S11=list()
for (i in 1:length(Sfig20lisres)) {
  if(nrow(Sfig20lisres[[i]])<=1) {print(paste(category[i],'failed',sep=' '))
    Sfig20_S11[[i]]=NA}
  else {
    Observations=rep(nrow(fig2lisdat[[i]]),nrow(Sfig20lisres[[i]]))
    Studies=rep(length(unique(fig2lisdat[[i]][,1])),nrow(Sfig20lisres[[i]]))
    Sfig20_S11[[i]]=as.matrix(cbind(Sfig20lisres[[i]][,c('Trophic','Predictor','Response')],
                                    Observations,Studies,
                                    Sfig20lisres[[i]][,c('Estimate','Std.Error','P.Value')]))
  }
}
S11_ah=sapply(1:length(fig2_S11),
              function(x) {rbind(fig2_S11[[x]],Sfig20_S11[[x]])})
S11=do.call('rbind',S11_ah[-7])
write.xlsx(S11,"Supplementary Table 11.xls",row.names = F)

########## Supplementary Fig. 17, 20 and supplementary table 13 ##########
fig20a=subset(fig2,Study.type=='Plot-experiment')
fig20b=subset(fig2,Study.type=='Pot-experiment')
fig20c=subset(fig2,Plant.type=='Herbaceous-plant')
fig20d=subset(fig2,Plant.type=='Woody-plant')
fig20e=subset(fig2,Biome=='Temperate'&Outdoor..indoor.or.greenhouse.exp.=='Outdoor')
fig20f=subset(fig2,Biome=='Tropical'&Outdoor..indoor.or.greenhouse.exp.=='Outdoor')
fig20lisdat=list(fig20a,fig20b,fig20c,fig20d,fig20e,fig20f)
category=c('Plot-experiment','Pot-experiment',
           'Herbaceous-plant','Woody-plant','Temperate','Tropical')

# Supplementary Fig. 20
fig20lisres=list()
for (i in 1:length(fig20lisdat)) {
  if(nrow(fig20lisdat[[i]])==0) {print(paste(category[i],'failed',sep=' '))
    fig20lisres[[i]]=cbind(category[i],NA)}
  else {
    SMD_pest<-escalc(measure="SMD",n2i=pest.Control.n, n1i=pest.Treatment.n,
                     m2i=pest.Control..value., m1i=pest.Treatment..value.,
                     sd2i=pest.Control.sd, sd1i=pest.Treatment.sd,
                     data=fig20lisdat[[i]],vtype = "UB",append = F)
    SMD_plant<-escalc(measure="SMD",n2i=plant.Control.n, n1i=plant.Treatment.n,
```

```
                         m2i=plant.Control..value., m1i=plant.Treatment..value.,
                         sd2i=plant.Control.sd, sd1i=plant.Treatment.sd,
                         data=fig20lisdat[[i]],vtype = "UB",append = F)
fig20lisdat[[i]]$yi_SMD_pest<-SMD_pest$yi
fig20lisdat[[i]]$vi_SMD_pest<-SMD_pest$vi
fig20lisdat[[i]]$yi_SMD_plant<-SMD_plant$yi
fig20lisdat[[i]]$vi_SMD_plant<-SMD_plant$vi
fig20lisdat[[i]]$id=1:nrow(fig20lisdat[[i]])
fig20lisdat[[i]]=na.omit(fig20lisdat[[i]])
if (length(unique(fig20lisdat[[i]]$Plant.species))==1) {
  res_pest=rma.mv(yi_SMD_pest,vi_SMD_pest,
                         data = fig20lisdat[[i]])
  res_plant=rma.mv(yi_SMD_plant,vi_SMD_plant,
                          data = fig20lisdat[[i]])
} else {
  phylodat=myphylo(fig20lisdat[[i]])
  res_pest=rma.mv(yi_SMD_pest,vi_SMD_pest,
                         random = list(~ 1 | Plant.species.new,~ 1|Plant.species.new.p),
                         R=list(Plant.species.new.p=phylodat[[2]]),
                         data = phylodat[[1]])
  res_plant=rma.mv(yi_SMD_plant,vi_SMD_plant,
                          random = list(~ 1 | Plant.species.new,~ 1|Plant.species.new.p),
                          R=list(Plant.species.new.p=phylodat[[2]]),
                          data = phylodat[[1]])
}
model.list <- list(
    lme(yi_SMD_plant ~ yi_SMD_pest,
        random = list(~ 1 | Code, ~ 1 | Plant.species),
        data = fig20lisdat[[i]],
        weights = varFixed(~ vi_SMD_plant), control = lmeControl(sigma = 1))
)
psem_list <- as.psem(model.list)
fit <- summary(psem_list, .progressBar = FALSE)
fig20lisres1=as.matrix(subset(fit$coefficients,
                               select = -c(Crit.Value,DF,Std.Estimate))[,1:5])
fig20lisres[[i]]=matrix(c('Pest performance','Plant performance',
                          rep('Plant genetic diversity',2),
                          res_pest$b,res_plant$b,
                          res_pest$se,res_plant$se,
                          res_pest$pval,res_plant$pval),nrow = 2)
```

```
        fig20lisres[[i]]=rbind(fig20lisres[[i]][1,],fig20lisres1,fig20lisres[[i]][2,])
        Trophic=rep(paste0(category[i],'| Plant genetic diversity on ','plant antagonist vs. plant'),
                        nrow(fig20lisres[[i]]))
        fig20lisres[[i]]=cbind(Trophic,fig20lisres[[i]])
    }
}
write.csv(fig20lisres[[1]],'Supplementary Fig.20a_results.csv',row.names = F)
write.csv(fig20lisres[[2]],'Supplementary Fig.20b_results.csv',row.names = F)
write.csv(fig20lisres[[3]],'Supplementary Fig.20c_results.csv',row.names = F)
write.csv(fig20lisres[[4]],'Supplementary Fig.20d_results.csv',row.names = F)
write.csv(fig20lisres[[5]],'Supplementary Fig.20e_results.csv',row.names = F)
write.csv(fig20lisres[[6]],'Supplementary Fig.20f_results.csv',row.names = F)

# Supplementary Fig 4
Sfig17lisres=list()
for (i in 1:length(fig20lisdat)) {
    log_num_additional<-log2(fig20lisdat[[i]]$No..genotypes.treat-fig20lisdat[[i]]$No..genotypes.control)
    if(length(unique(log_num_additional))<=1) {print(paste(category[i],'failed',sep=' '))
        Sfig17lisres[[i]]=cbind(category[i],NA)}
    else {
        fig20lisdat[[i]]$log_num_additional<-log_num_additional
        model.list <- list(
            lme(yi_SMD_pest ~ log_num_additional,
                    random = list(~ 1 | Code, ~ 1 | Plant.species),
                    data = fig20lisdat[[i]],
                    weights = varFixed(~ vi_SMD_pest), control = lmeControl(sigma = 1)),

            lme(yi_SMD_plant ~ yi_SMD_pest + log_num_additional,
                    random = list(~ 1 | Code, ~ 1 | Plant.species),
                    data = fig20lisdat[[i]],
                    weights = varFixed(~ vi_SMD_plant), control = lmeControl(sigma = 1))
        )
        psem_list <- as.psem(model.list)
        fit <- summary(psem_list, .progressBar = FALSE)
        Sfig17lisres[[i]]=subset(fit$coefficients,select = -Crit.Value)
        Trophic=rep(paste0(category[i],'| Number of added genotypes on ','plant antagonist vs. plant'),
                        nrow(fig20lisres[[i]]))
        Sfig17lisres[[i]]=cbind(Trophic,Sfig17lisres[[i]])
    }
}
```

```
write.csv(Sfig17lisres[[1]],'Supplementary Fig. 17a_results.csv',row.names = F)
write.csv(Sfig17lisres[[2]],'Supplementary Fig. 17b_results.csv',row.names = F)
write.csv(Sfig17lisres[[3]],'Supplementary Fig. 17c_results.csv',row.names = F)
write.csv(Sfig17lisres[[4]],'Supplementary Fig. 17d_results.csv',row.names = F)
write.csv(Sfig17lisres[[5]],'Supplementary Fig. 17e_results.csv',row.names = F)
write.csv(Sfig17lisres[[6]],'Supplementary Fig. 17f_results.csv',row.names = F)
write.csv(Sfig17lisres[[7]],'Supplementary Fig. 17g_results.csv',row.names = F)

# Supplementary Table 13
S24_S13=list()
for (i in 1:length(fig24lisres)) {
  if(nrow(fig24lisres[[i]])<=1) {print(paste(category[i],'failed',sep=' '))
    S24_S13[[i]]=NA}
  else {
    Observations=rep(nrow(fig24lisdat[[i]]),nrow(fig24lisres[[i]]))
    Studies=rep(length(unique(fig24lisdat[[i]][,1])),nrow(fig24lisres[[i]]))
    S24_S13[[i]]=cbind(fig24lisres[[i]][,c('Trophic','Predictor','Response')],
                       Observations,Studies,
                       fig24lisres[[i]][,c('Estimate','Std.Error','P.Value')])
  }
}
Sfig21_S13=list()
for (i in 1:length(Sfig21lisres)) {
  if(nrow(Sfig21lisres[[i]])<=1) {print(paste(category[i],'failed',sep=' '))
    Sfig21_S13[[i]]=NA}
  else {
    Observations=rep(nrow(fig24lisdat[[i]]),nrow(Sfig21lisres[[i]]))
    Studies=rep(length(unique(fig24lisdat[[i]][,1])),nrow(Sfig21lisres[[i]]))
    Sfig21_S13[[i]]=as.matrix(cbind(Sfig21lisres[[i]][,c('Trophic','Predictor','Response')],
                                    Observations,Studies,
                                    Sfig21lisres[[i]][,c('Estimate','Std.Error','P.Value')]))
  }
}
S13_ag=lapply(1:length(S24_S13), function(x) {rbind(S24_S13[[x]],Sfig21_S13[[x]])})
S13=do.call('rbind',S13_ag)
write.xlsx(S13,"Supplementary Table 13.xls",row.names = F)
```

Meta-regression in a number of data subsets

We conducted various meta-regression models for a series of data subsets. Specifically, each trophic group (plant

antagonists, invertebrate herbivores, natural enemies of invertebrate herbivores, weeds, plant-feeding nematodes, plant diseases and plants) was investigated respectively for examining the impact of the increasing of plant genotypes on the homozygous genotype (i.e. the number of genotypes in the plant genetic diversity was changed by manipulated in experimental designs), where we took log_2-transformed number of plant genotypes added as a moderator term. In addition, we also treated the type of ecosystem, experiment study, plant life form or climatic zone as a moderator term separately to evaluate the effect sizes in different ecosystems, experimental studies, plant life forms and climatic zones (excluding greenhouse or indoor observations) of each trophic group. In all these subset analyses, we performed LRTs by employing the intercept-only models as reference models.

Supplementary information on path analysis

Student´s t-tests were applied to assess statistical significance, where a positive SMD revealed that the plant genetic diversity could increase the mean value of the response variable in the trophic group and a negative SMD and t-test statistic indicated that the plant genetic diversity could reduce the mean value of the response variable in the trophic group (Supplementary Table 14).

In this paper, tri-trophic interactions included the interactions among invertebrate herbivore, natural enemy and plant performances, and bi-trophic interactions encompassed the interactions between invertebrate herbivore and plant performances, weed and plant performances, plant-feeding nematode and plant performances, as well as plant disease and plant performances. We fitted various models to explore the direct and indirect effects of plant genetic diversity on tri-trophic and bi-trophic interactions in all ecosystems (Supplementary Tables 8, 9). Do the same thing on the bi-trophic interactions of plant antagonist and plant performances in different ecosystems (i.e., global ecosystem, agroecosystem, grassland, forest, old-field ecosystem, marine ecosystem, wetlands and shrubland) (Supplementary Tables 10, 11 and Supplementary Figs. 19a–f), and on the trophic interactions of plant antagonist and plant performances in different experiments (i.e., plot and pot experiments), two plant life forms (herbaceous and woody plants) and two climatic zones (temperate and tropical zones (excluding greenhouse or indoor observations) (Supplementary Tables 12, 13 and Supplementary Figs. 20a–f).

Because the gradient effects of the number of increased genotypes of treatment over that of control cannot be replaced by the plant genetic diversity, we further explored the effects of number of added plant genotypes over the control (i.e., plants with homozygous genotype). In other words, we constructed various models to explore the direct and indirect effects of number of added plant genotypes on tri-trophic and bi-trophic interactions in all ecosystems (Supplementary Tables 8, 9). Do the same thing on the bi-trophic interactions of plant antagonist and plant performances in different ecosystems (Supplementary Tables 10 and 11; Supplementary Figs. 16a-f), and on the trophic interactions of plant antagonist and plant performances in different experiments, two plant life forms and two climatic zones (excluding greenhouse or indoor observations) (Supplementary Tables 12, 13 and Supplementary Figs. 17a–f).

The goodness-of-fit for path analysis models were examined by the d-separation test based on the Fisher' C statistic. The fitted model has a significant lack-of –fit if the p-value of it is < 0.05; this is a bit counter-intuitive: large P values indicate good support of a path analysis model by the data. All the 13 studies (including 91 observations) were comprised in the model which was derived from the experimental studies and each pairwise comparison (natural enemy performance vs. invertebrate herbivore performance vs. plant performance) was tested at the same site for the path analysis of tri-

trophic interactions in invertebrate herbivore, natural enemy and plant performances (Supplementary Tables 8, 9). Similarly, in the path analysis for each bi-trophic interaction of any two trophic groups, all studies involved in this model were also tested at the same site (Supplementary Tables 10–13). Importantly, the study identity and plant species treated as a random effect in the model could exclude most of effect of confounding variables and pseudo-replicated observations.

Effects of different ecosystems, experimental studies, plant life forms and climatic zones (excluding greenhouse or indoor observations) on trophic group responses

1) Responses of invertebrate herbivores, natural enemies of herbivores, weeds, plant-feeding nematodes, plant diseases and plants

Different ecosystems had significant difference in response to plant genetic diversity among the six trophic groups (i.e., invertebrate herbivores, herbivore natural enemies, weeds, plant-feeding nematodes, plant diseases and plants) (X^2 =25.6048, df = 14, P=0.0003; Supplementary Table 1). In agroecosystems, plant genetic diversity had positively significant effects on plants (t_{2538} = 8.2750, P< 0.0001), and negatively significant effects on herbivores (t_{310} = -5.4189, P< 0.0001), weeds (t_{178} =-3.3504, P=0.0008) and plant diseases (t_{1013} = -5.2113, P< 0.0001). In grasslands, plant genetic diversity had positively significant effects on natural enemies (t_{20}=4.5133, P< 0.0001) and plants (t_{134} =4.6700, P< 0.0001), negatively significant effects on herbivores (t_{25}= -2.2319, P=0.0256) and plant disease (t_8= -3.0311, P=0.0024). In forests, the responses of herbivores, enemies, plant diseases and plants to plant genetic diversity were not significant (herbivore: t_{79}= -0.6788, P=0.4973; enemy: t_7=0.6015, P=0.5475; disease: t_{10}= -1.0790, P=0.2806; plant: t_{61}=0.6028, P =0.5467). In old-field ecosystems, the responses of diseases to plant genetic diversity were significantly negative (t_2= -3.1762, P=0.0015), the responses of herbivores were not significant (herbivore: t_{18}=1.2242, P=0.2209), and the responses of enemies and plants were significantly positive (enemy: t_{14}=3.4054, P=0.0007; plants: t_{27}=5.5731, P<0.0011). In marine ecosystems, plant genetic diversity had significant effects only on plants (t_{37} =3.7529, P=0.0002), and had marginally significant effects on herbivores (t_{13}= -1.7652, P=0.0775). Due to lack of observations, the effects of plant genetic diversity on trophic groups were not significant or cannot be obtained in shrublands or wetlands (Supplementary Table 4). Overall, the trophic group response categories had a significant influence on the effects of different ecosystems (X^2 =86.1858, df=24, P<0.0001), whose results for different subsets were observed in Supplementary Table 4 and Fig. 4.

The types of experiment study were not significantly associated with the plant genetic diversity among the six trophic groups (X^2 =0.0846, df =9, P =0.7711; Supplementary Table 1). For plot experiment, plant genetic diversity had positive effects on enemies (t_{100}=3.9787, P < 0.0001) and plants (t_{2667} =8.5367, P < 0.0001), and negatively significant effects on herbivores (t_{406} = -3.9446, P < 0.0001) and disease (t_{1010}= -4.3422, P < 0.0001). For pot experiment, the responses to plant genetic diversity were positive on enemies (t_4 =1.8947, P =0.0581) and plants (t_{195} =3.6388, P=0.0003), while negative on other trophic groups (herbivore: t_{62} = -0.8224, P=0.4109; weed: t_{69}= -6.4918, P < 0.0001; disease: t_{23}= -1.2167, P =0.2237). In summary, the effects of the types of experimental study were significantly depend on the trophic group response categories (X^2 = 63.0993, df = 19, P < 0.0001; Supplementary Table 1), and the results of these different subsets were presented in Supplementary Table 5 and Fig. 5.

The plant life forms were significantly linked with the plant genetic diversity among the six trophic groups (X^2 =1.5972, df =9, P=0.2063; Supplementary Table 1). For herbaceous plants, the responses of the six trophic groups to plant genetic diversity were significant (herbivore: t_{336} = -4.1377, P<0.0001; enemy: t_{84}=3.7356, P=0.0002; plant disease:

t_{1010}= -6.8910, P<0.0001; plant: t_{2741}=8.9148, P<0.0001). There were no responses from weeds or plant-feeding nematodes in woody-dominated ecosystems , but plant genetic diversity had marginally significant effects on two groups (herbivore: t_{132}= -1.7636, P=0.0778; enemy: t_{20}=1.8212, P=0.0686) and had no significant effects on other two groups (plant disease: t_{23}= -0.6864, P=0.4925; plant: t_{121}=1.4127, P=0.1577). Generally, the trophic group response categories had a significant association with the effects of plant life form (X^2 =64.2222, df =19, P<0.0001; Supplementary Table 1), and we can get the results of subsets in Supplementary Table 6 and Fig. 6.

The differences in response to plant genetic diversity among the six trophic groups marginally but not significantly counted on the climatic zone types (excluding greenhouse or indoor observations) (X^2 =3.2202, df =9, P=0.0727; Supplementary Table 1). In temperate climatic zones, the responses of the six trophic groups to plant genetic diversity were not always significant (herbivore: t_{384}= -3.2895, P=0.0010; enemy: t_{85}=5.7467, P<0.0001; weed: t_{138}= -0.1462, P=0.8838; plant-feeding nematode: t_{29}= -1.2976, P=0.1944; plant disease: t_{939}= -5.4873, P< 0.0001; plant: t_{2516}=7.8416, P< 0.0001). In tropical climatic zones, the responses of four trophic groups to plant genetic diversity were significant (herbivore: t_{58}= -3.4824, P=0.0005; plant-feeding nematode: t_6= -2.0777, P=0.0377; disease: t_{71} = -2.2319, P=0.0256; plant: t_{189} =4.8670, P<0.0001). Overall, the effects of climatic zone type counted significantly on the trophic group response categories (X^2 =6.0541, df=19, P=0.0139; Supplementary Table 1), and the results for these different subsets were listed in Supplementary Table 7 and Fig. 7.

2) Responses of plant antagonists, natural enemies of herbivores and plants

When separated by different types of ecosystems, experimental studies, plant life forms and climatic zones (excluding greenhouse or indoor observations), the overall observations were insufficient to conduct path analysis, so we integrated the trophic groups of invertebrate herbivores, weeds, plant-feeding nematodes and plant disease into one subset for obtaining another three trophic groups (i.e. plant antagonists, natural enemies of herbivores and plants). The above adjacent paragraph had introduced the responses of natural enemies and plants toward to plant genetic diversity. For concision, we only interpreted the response of plant antagonists comprised plant antagonist intensity and plant antagonist diversity to the plant genetic diversity.

There existed significant difference in types of ecosystems for the three trophic groups (i.e. plant antagonists, natural enemies and plants) responded to the plant genetic diversity (ecosystem: X^2 =48.5123, df=11, P < 0.0001), while not significant difference in experimental studies, plant life forms or climatic zones (excluding greenhouse or indoor observations) for the three trophic groups (experimental study: X^2 =3.0741, df=6, P=0.0795; plant life form: X^2=0.4639, df=6, P=0.4958; climatic zone: X^2=1.4170, df=6, P=0.2339; Supplementary Table 1).

In agroecosystems, plant genetic diversity had significantly negative effects on plant antagonists, plant antagonist intensity but not plant antagonist diversity (plant antagonists: t_{1536}= -6.6770, P< 0.0001; plant antagonist intensity: t_{1517}= -7.1618, P< 0.0001; plant antagonist diversity: t_{19}= -1.5274, P=0.1267). In grasslands, plant genetic diversity had marginally but not significantly negative effects on plant antagonists (i.e., plant antagonist intensity) without observations of plant antagonist diversity (plant antagonists or plant antagonist intensity: t_{55}= -1.8706, P=0.0614). In forest, plant genetic diversity did not have significant effects on plant antagonists, plant antagonist intensity or plant antagonist diversity (plant antagonists: t_{89}= -0.4616, P=0.6444; plant antagonist intensity: t_{85}= -0.2368, P=0.8128; plant antagonist diversity: t_4= -0.2199, P=0.8260). In old-field ecosystems, the responses of plant antagonist performance and plant

antagonist diversity to plant genetic diversity were significant (plant antagonists: t_{20}=2.3269, P=0.0200; plant antagonist diversity: t_4=3.3837, P= 0.0007), but the responses of plant antagonist intensity were not significant (plant antagonist intensity: t_{16}=1.0458, P=0.2957). In marine ecosystems and shrublands, the response of plant antagonists (i.e., plant antagonist intensity) without observations of plant antagonist diversity was not significantly associated with the plant genetic diversity (plant antagonists or plant antagonist intensity in marine ecosystems: t_{13}= -1.7652, P=0.0775; plant antagonists or plant antagonist intensity in shrublands: t_{23}=0.1779, P=0.3620) (Supplementary Table 4).

For plot experiments, plant genetic diversity had not significant effects on plant antagonists (t_{1582}= -0.7231, P =0.4696), plant antagonist intensity (t_{1557}= -0.6906, P=0.4898) or plant antagonist diversity (t_{25}= -0.0352, P=0.9719), and similar effects were found for pot experiments (plant antagonists: t_{154}= -1.2485, P= 0.2119; plant antagonist intensity: t_{152}=-1.2699, P=0.2041; plant antagonist diversity: t_2=0.4823, P= 0.6296) (Supplementary Table 5).

For herbaceous plants, the associations of plant antagonists, plant antagonist intensity or plant antagonist diversity with plant genetic diversity were not significant (plant antagonists: t_{1581} = -1.3647, P =0.1723; plant antagonist intensity: t_{1558} = -1.5086, P =0.1314; plant antagonist diversity: t_{23} =0.0729, P=0.9419), and these similar results were also found for woody plants (plant antagonists: t_{155}= -1.6345, P =0.1021; plant antagonist intensity: t_{151}= -1.6511, P=0.0987; plant antagonist diversity: t_4= -0.2199, P= 0.8260) (Supplementary Table 6).

In temperate zones (excluding greenhouse or indoor observations), plant genetic diversity had significant effects on plant antagonists, but not significant for plant antagonist intensity or plant antagonist diversity (plant antagonists: t_{1490}= -2.0265, P=0.0427; plant antagonist intensity: t_{1468}= -1.7605, P=0.0783; plant antagonist diversity: t_{22}= 0.0818, P=0.9348). In addition, significant effects were on plant antagonists and plant antagonist intensity but not on plant antagonist diversity in trophic zones (plant antagonists: t_{140}= -4.4638, P< 0.0001; plant antagonist intensity: t_{135}= -4.7490, P<0.0001; plant antagonist diversity: t_5= 0.0654, P=0.94785) (Supplementary Table 7).

In conclusion, significant effects were detected between the trophic group and the different subsets including ecosystem type but not including experimental study type, plant life or climate zone (excluding greenhouse or indoor observations) (ecosystem: X^2 =48.5123, df =11, P < 0.0001; experimental study: X^2 =3.0741, df =6, P=0.0795; plant life form: X^2 =0.4639, df =6, P=0.4958; climatic zone: X^2 =1.4170, df=6, P=0.2339; Supplementary Table 1). The effects of ecosystem types, types of experimental study and plant life forms significantly depended on the trophic group response categories, respectively (ecosystem: X^2 =121.2040, df =18, P < 0.0001; experimental study: X^2 =80.7442, df =13, P < 0.0001; plant life form: X^2 =77.0929, df =13, P < 0.0001; climatic zone: X^2 = 2.6810, df =13, P = 0.1016; Supplementary Table 1). All the results can be revealed in Supplementary Tables 4–7 and Figs. 4–7.

Publication bias evaluation

The regression model for publication bias of the overall observations was based on the raw effect size showed that there was publication bias for the 4702 observations of the 413 cited articles in this paper (value of regression test = -30.5120, P < 0.0001). Therefore, we did sensitivity analysis in different trophic groups (plant antagonist performance, herbivore performance, natural enemy performance, weed performance, nematode performance, disease performance and plant performance) and the results can be seen in Supplementary Table 14. As a result, after removing the extreme studies,

the results of different trophic groups had not changed. In other words, our results were stable even if there had been publication bias. However, since it is important for researchers to account for the heterogeneity in different studies, Pappalardo et al. (2020)[6] argued that applying the residuals from the regression models of various parametric settings may be a more appropriate approach to perform publication bias evaluation. As a result, publication bias was assessed for all models (Supplementary Table 2). Additionally, we did the fail-safe test and the number was 280436 for the whole dataset from 413 cited articles, which was much larger than the as-hoc threshold 23520 (23520=5*n* + 10, where *n* is the number of observations in the analyses (*n* = 4702 in our meta-analysis)). That means, at least 280436 studies with negative effects need to be incorporated in our dataset for showing an insignificant overall effect size, proving that the result in this paper is strongly stable for publication bias.

R code:

```
library(metafor)
library(xlsx)
library(V.PhyloMaker)
setwd("...")
########## loading data ########
plant_antagonist_intensity<-read.csv("plant antagonist intensity.csv",header = TRUE)
plant_antagonist_diversity<-read.csv("plant antagonist diversity.csv",header = TRUE)
Totalplant_antagonist<-rbind(plant_antagonist_intensity,plant_antagonist_diversity)
Herbivore_abundance<-read.csv("herbivore abundance.csv",header = TRUE)
Herbivore_diversity<-read.csv("herbivore diversity.csv",header = TRUE)
Herbivore_damage<-read.csv("herbivore damage.csv",header = TRUE)
TotalHerbivore<-rbind(Herbivore_abundance,Herbivore_diversity,Herbivore_damage)
Predator_abundance<-read.csv("predator abundance.csv",header = TRUE)
Predator_diversity<-read.csv("predator diversity.csv",header = TRUE)
TotalPredator<-rbind(Predator_abundance,Predator_diversity)
Parasitoid_abundance<-read.csv("parasitoid abundance.csv",header = TRUE)
Parasitoid_diversity<-read.csv("parasitoid diversity.csv",header = TRUE)
Parasitism<-read.csv("parasitism.csv",header = TRUE)
TotalParasitoid<-rbind(Parasitoid_abundance,Parasitoid_diversity,Parasitism)
Weed_growth<-read.csv("weed growth.csv",header = TRUE)
Weed_diversity<-read.csv("weed diversity.csv",header = TRUE)
TotalWeed<-rbind(Weed_growth,Weed_diversity)
nematode_abundance<-read.csv("nematode abundance.csv",header = TRUE)
Disease_spread<-read.csv("disease spread.csv",header = TRUE)
Disease_damage<-read.csv("disease damage.csv",header = TRUE)
TotalDisease<-rbind(Disease_spread,Disease_damage)
Plant_growth<-read.csv("plant growth.csv",header = TRUE)
Plant_quality<-read.csv("plant quality.csv",header = TRUE)
```

```
Plant_reproduction<-read.csv("plant reproduction.csv",header = TRUE)
TotalPlant<-rbind(Plant_growth,Plant_quality,Plant_reproduction)
enemy<-rbind(TotalPredator,TotalParasitoid)

lisdat=list(Totalplant_antagonist,TotalHerbivore,enemy,TotalWeed,nematode_abundance,
            TotalDisease,TotalPlant,plant_antagonist_intensity,plant_antagonist_diversity,
            Herbivore_abundance,Herbivore_damage,Herbivore_diversity,
            Predator_abundance,Predator_diversity,Parasitoid_abundance,
            Parasitoid_diversity,Parasitism,Weed_growth,Weed_diversity,
            nematode_abundance,Disease_spread,Disease_damage,Plant_growth,
            Plant_quality,Plant_reproduction)
Total=rbind(Totalplant_antagonist,TotalHerbivore,enemy,TotalWeed,nematode_abundance,
            TotalDisease,TotalPlant)
Rnames=c("plant antagonist performance","herbivore performance",
         "enemy performance of herbivores","weed performance",
         "nematode performance","disease performance","plant performance",
         "plant antagonist intensity","plant antagonist diversity","Herbivore abundance",
         "Herbivore damage","Herbivore diversity","Predator abundance",
         "Predator diversity","Parasitoid abundance","Parasitoid diversity",
         'Parasitism','Weed growth','Weed diversity','nematode abundance',
         'disease spread','disease damage','Plant growth',
         'Plant quality','Plant reproduction')

######Tree and Phylogenies made######
myphylo=function(Total.data2){
  species=read.xlsx('plant species information-0713-wan-revised.xls',1)
  species=subset(species,species %in% unique(Total.data2$Plant.species))
  mycor=phylo.maker(species)
  mytree <- compute.brlen(mycor$scenario.3)
  A <- vcv(mytree, corr=TRUE)
  Total.data2$Plant.species=factor(Total.data2$Plant.species)
  Total.data2$Plant.species.new=Total.data2$Plant.species
  levels(Total.data2$Plant.species.new)=sort(dimnames(A)[[1]])
  Total.data2$Plant.species.new.p=Total.data2$Plant.species.new
  return(list(Total.data2,A))
}


######### Supplementary Table 3 ##########
res=matrix(NA,length(lisdat),5)
```

```
for (i in 1:length(lisdat)) {
  res1=escalc(measure="SMD",n1i=Tn, n2i=Cn, m1i=T, m2i=C,
              sd1i=Tsd, sd2i=Csd,
              data=lisdat[[i]],vtype = "UB",append = TRUE)
  phylodat=myphylo(res1)
  res2=rma.mv(yi, vi,
              random = list(~ 1 | Plant.species.new,~ 1|Plant.species.new.p),
              R=list(Plant.species.new.p=phylodat[[2]]),
              data = phylodat[[1]])
  res[i,]=c(nrow(res1),length(unique(res1$Code)),
            res2$b,res2$zval,res2$pval)
}
res<-as.data.frame(res,row.names<-Rnames)
colnames(res)<-c("#Obs","#Studies","Effect size","T-value","P-value")
write.xlsx(res,"Supplementary Table 3.xls")

########## Supplementary Table 4 ###########
res=matrix(NA,length(lisdat),5)
res_all=list()
subgroup=c('Agroecosystem','Grassland','Forest',
           'Old-field-ecosystem','Marine-ecosystem',
           'Wetland','Shrubland')
for (j in 1:length(subgroup)) {
  for (i in 1:length(lisdat)) {
    if(nrow(subset(lisdat[[i]],Ecosystem==subgroup[j]))<=1) {res[i,]=NA}
    else {
      res1=escalc(measure="SMD",n1i=Tn, n2i=Cn, m1i=T, m2i=C,
                  sd1i=Tsd, sd2i=Csd,data=lisdat[[i]],
                  subset=(Ecosystem==subgroup[j]),
                  vtype = "UB",append = TRUE)

      if (length(unique(res1$Plant.species))==1) {
        res2=rma.mv(yi, vi,data = res1)
      } else {
        phylodat=myphylo(res1)
        res2=rma.mv(yi, vi,
                    random = list(~ 1 | Plant.species.new,~ 1|Plant.species.new.p),
                    R=list(Plant.species.new.p=phylodat[[2]]),
                    data = phylodat[[1]])
      }
```

```
      res[i,]=c(nrow(res1),length(unique(res1$Code)),
                res2$b,res2$zval,res2$pval)
    }
  }
  res_all[[j]]=res
}
res=do.call('rbind',res_all)
Ecosystem_names=paste(rep(subgroup,each=length(Rnames)),
                      rep(Rnames,length(subgroup)),sep = '_')
res<-as.data.frame(res,row.names<-Ecosystem_names)
colnames(res)<-c("#Obs","#Studies","Effect size","T-value","P-value")
write.xlsx(res,"Supplementary Table 4.xls")

########## Supplementary Table 5 ###########
res=matrix(NA,length(lisdat),5)
res_all=list()
subgroup=c('Plot-experiment','Pot-experiment')
for (j in 1:length(subgroup)) {
  for (i in 1:length(lisdat)) {
    if(nrow(subset(lisdat[[i]],Study.type==subgroup[j]))<=1) {res[i,]=NA}
    else {
      res1=escalc(measure="SMD",n1i=Tn, n2i=Cn, m1i=T, m2i=C,
                  sd1i=Tsd, sd2i=Csd,data=lisdat[[i]],
                  subset=(Study.type==subgroup[j]),
                  vtype = "UB",append = TRUE)
      if (length(unique(res1$Plant.species))==1) {
        res2=rma.mv(yi, vi,data = res1)
      } else {
        phylodat=myphylo(res1)
        res2=rma.mv(yi, vi,
                    random = list(~ 1 | Plant.species.new,~ 1|Plant.species.new.p),
                    R=list(Plant.species.new.p=phylodat[[2]]),
                    data = phylodat[[1]])
      }
      res[i,]=c(nrow(res1),length(unique(res1$Code)),
                res2$b,res2$zval,res2$pval)
    }
  }
  res_all[[j]]=res
}
```

```
res=do.call('rbind',res_all)
study.type_names=paste(rep(subgroup,each=length(Rnames)),
                       rep(Rnames,length(subgroup)),sep = '_')
res<-as.data.frame(res,row.names<-study.type_names)
colnames(res)<-c("#Obs","#Studies","Effect size","T-value","P-value")
write.xlsx(res,"Supplementary Table 5.xls")

########## Supplementary Table 6 ###########
res=matrix(NA,length(lisdat),5)
res_all=list()
subgroup=c('Herbaceous-plant','Woody-plant')
for (j in 1:length(subgroup)) {
  for (i in 1:length(lisdat)) {
    if(nrow(subset(lisdat[[i]],Plant.type==subgroup[j]))<=1) {res[i,]=NA}
    else {
      res1=escalc(measure="SMD",n1i=Tn, n2i=Cn, m1i=T, m2i=C,
                  sd1i=Tsd, sd2i=Csd,data=lisdat[[i]],
                  subset=(Plant.type==subgroup[j]),
                  vtype = "UB",append = TRUE)
      if (length(unique(res1$Plant.species))==1) {
        res2=rma.mv(yi, vi,data = res1)
      } else {
        phylodat=myphylo(res1)
        res2=rma.mv(yi, vi,
                    random = list(~ 1 | Plant.species.new,~ 1|Plant.species.new.p),
                    R=list(Plant.species.new.p=phylodat[[2]]),
                    data = phylodat[[1]])
      }
      res[i,]=c(nrow(res1),length(unique(res1$Code)),
                res2$b,res2$zval,res2$pval)
    }
  }
  res_all[[j]]=res
}
res=do.call('rbind',res_all)
Plant.type_names=paste(rep(subgroup,each=length(Rnames)),
                       rep(Rnames,length(subgroup)),sep = '_')
res<-as.data.frame(res,row.names<-Plant.type_names)
colnames(res)<-c("#Obs","#Studies","Effect size","T-value","P-value")
write.xlsx(res,"Supplementary Table 6.xls")
```

```
########## Supplementary Table 7 ###########
res=matrix(NA,length(lisdat),5)
res_all=list()
subgroup=c('Temperate','Tropical')
for (j in 1:length(subgroup)) {
  for (i in 1:length(lisdat)) {
    if(nrow(subset(lisdat[[i]],Biome==subgroup[j]))<=1) {res[i,]=NA}
    else {
      res1=escalc(measure="SMD",n1i=Tn, n2i=Cn, m1i=T, m2i=C,
                  sd1i=Tsd, sd2i=Csd,data=lisdat[[i]],
                  subset=(Biome==subgroup[j]),
                  vtype = "UB",append = TRUE)
      res1=res1[res1$Outdoor..indoor.or.greenhouse.exp.=='Outdoor',]
      if (length(unique(res1$Plant.species))==1) {
        res2=rma.mv(yi, vi,data = res1)
      } else {
        phylodat=myphylo(res1)
        res2=rma.mv(yi, vi,
                    random = list(~ 1 | Plant.species.new,~ 1|Plant.species.new.p),
                    R=list(Plant.species.new.p=phylodat[[2]]),
                    data = phylodat[[1]])
      }
      res[i,]=c(nrow(res1),length(unique(res1$Code)),
                res2$b,res2$zval,res2$pval)
    }
  }
  res_all[[j]]=res
}
res=do.call('rbind',res_all)
Biome_names=paste(rep(subgroup,each=length(Rnames)),
                  rep(Rnames,length(subgroup)),sep = '_')
res<-as.data.frame(res,row.names<-Biome_names)
colnames(res)<-c("#Obs","#Studies","Effect size","T-value","P-value")
write.xlsx(res,"Supplementary Table 7.xls")

########## Supplementary Table 8###########
enemy_herbivore_plant=read.csv("enemy vs. herbivore vs. plant.csv",header = TRUE)
herbivore_plant=read.csv("herbivore vs. plant.csv",header = TRUE)
weed_plant=read.csv("weed vs. plant.csv",header = TRUE)
```

```
nematode_plant=read.csv("nematode vs. plant.csv",header = TRUE)
disease_plant=read.csv("disease vs. plant.csv",header = TRUE)
S8lisdat=list(enemy_herbivore_plant[,c(1,9:14,27:28)],enemy_herbivore_plant[,c(1,15:20,27:28)],
              enemy_herbivore_plant[,c(1,21:28)],herbivore_plant[,c(1,8:13,20:21)],
              herbivore_plant[,c(1,14:21)],weed_plant[,c(1,8:13,20:21)],
              weed_plant[,c(1,14:21)],nematode_plant[,c(1,8:13,20:21)],
              nematode_plant[,c(1,14:21)],disease_plant[,c(1,8:13,20:21)],
              disease_plant[,c(1,14:21)])
res=matrix(NA,length(S8lisdat),5)
for (i in 1:length(S8lisdat)) {
  res1=escalc(measure="SMD",n1i=S8lisdat[[i]][,7], n2i=S8lisdat[[i]][,4],
              m1i=S8lisdat[[i]][,5], m2i=S8lisdat[[i]][,2],
              sd1i=S8lisdat[[i]][,6], sd2i=S8lisdat[[i]][,3],
              data=S8lisdat[[i]],vtype = "UB",append = TRUE)
  if (length(unique(res1$Plant.species))==1) {
    res2=rma.mv(yi, vi,data = res1)
  } else {
    phylodat=myphylo(res1)
    res2=rma.mv(yi, vi,
                random = list(~ 1 | Plant.species.new,~ 1|Plant.species.new.p),
                R=list(Plant.species.new.p=phylodat[[2]]),
                data = phylodat[[1]])
  }
  res[i,]=c(nrow(res1),length(unique(res1[,1])),
            res2$b,res2$zval,res2$pval)
}
S8_names=c(paste(rep('enemy.herbivore.plant',each=3),
                 c('enemy','Invertebrate herbivore','Plant'),sep = '_'),
           paste(rep('herbivore.plant',each=2),
                 c('Invertebrate','Plant'),sep = '_'),
           paste(rep('weed.plant',each=2),
                 c('Weed','Plant'),sep = '_'),
           paste(rep('nematode.plant',each=2),
                 c('nematode','Plant'),sep = '_'),
           paste(rep('disease.plant',each=2),
                 c('disease','Plant'),sep = '_'))
res<-as.data.frame(res,row.names<-S8_names)
colnames(res)<-c("#Obs","#Studies","Effect size","T-value","P-value")
write.xlsx(res,"Supplementary Table 8.xls")
```

```
# Supplementary Table 9, 11, 13 refer to "path analysis(Genetics)" file.

########## Supplementary Table 10 and 12 ###########
plant_antagonist_plant=read.csv("plant antagonist vs. plant.csv",header = TRUE)
plant_antagonist_plant_list=list(plant_antagonist_plant[,c(1:13,20:21)],
                                 plant_antagonist_plant[,c(1:7,14:21)])
plant_antagonist_name=c('plant antagonist','plant')

# Supplementary Table 10
S10_whole=matrix(NA,length(plant_antagonist_plant_list),5)
for (i in 1:length(plant_antagonist_plant_list)) {
  res1=escalc(measure="SMD",n1i=plant_antagonist_plant_list[[i]][,13],
              n2i=plant_antagonist_plant_list[[i]][,10],
              m1i=plant_antagonist_plant_list[[i]][,11],
              m2i=plant_antagonist_plant_list[[i]][,8],
              sd1i=plant_antagonist_plant_list[[i]][,12],
              sd2i=plant_antagonist_plant_list[[i]][,9],
              data=plant_antagonist_plant_list[[i]],vtype = "UB",append = TRUE)
  if (length(unique(res1$Plant.species))==1) {
    res2=rma.mv(yi, vi,data = res1)
  } else {
    phylodat=myphylo(res1)
    res2=rma.mv(yi, vi,
                random = list(~ 1 | Plant.species.new,~ 1|Plant.species.new.p),
                R=list(Plant.species.new.p=phylodat[[2]]),
                data = phylodat[[1]])
  }
  S10_whole[i,]=c(nrow(res1),length(unique(res1$Code)),
            res2$b,res2$zval,res2$pval)
}
S10_whole<-as.data.frame(S11_whole,
                         row.names<-matrix(paste(rep('Global ecosystem',
                                                     length(plant_antagonist_name)),
                                                 plant_antagonist_name,sep = '_')))
colnames(S10_whole)<-c("#Obs","#Studies","Effect size","T-value","P-value")

S10_Eco=c('Agroecosystem','Grassland','Forest',
          'Old-field-ecosystem','Marine-ecosystem','Wetland','Shrubland')
res=matrix(NA,length(plant_antagonist_plant_list),5)
res_all=list()
```

```
for (j in 1:length(S10_Eco)) {
  for (i in 1:length(plant_antagonist_plant_list)) {
    if(nrow(subset(plant_antagonist_plant_list[[i]],Ecosystem==S10_Eco[j]))==0) {res[i,]=NA}
    else {
      res1=escalc(measure="SMD",n1i=plant_antagonist_plant_list[[i]][,13],
                  n2i=plant_antagonist_plant_list[[i]][,10],
                  m1i=plant_antagonist_plant_list[[i]][,11],
                  m2i=plant_antagonist_plant_list[[i]][,8],
                  sd1i=plant_antagonist_plant_list[[i]][,12],
                  sd2i=plant_antagonist_plant_list[[i]][,9],
                  data=plant_antagonist_plant_list[[i]],subset=(Ecosystem==S10_Eco[j]),
                  vtype = "UB",append = TRUE)
      if (length(unique(res1$Plant.species))==1) {
        res2=rma.mv(yi, vi,data = res1)
      } else {
        phylodat=myphylo(res1)
        res2=rma.mv(yi, vi,
                    random = list(~ 1 | Plant.species.new,~ 1|Plant.species.new.p),
                    R=list(Plant.species.new.p=phylodat[[2]]),
                    data = phylodat[[1]])
      }
      res[i,]=c(nrow(res1),length(unique(res1$Code)),
                res2$b,res2$zval,res2$pval)
    }
  }
  res_all[[j]]=res
}
res=do.call('rbind',res_all)
Ecosystem_names=paste(rep(S10_Eco,each=length(plant_antagonist_name)),
                      rep(plant_antagonist_name,length(S10_Eco)),sep = '_')
res<-as.data.frame(res,row.names<-Ecosystem_names)
colnames(res)<-c("#Obs","#Studies","Effect size","T-value","P-value")
S10_res=rbind(S10_whole,res)
write.xlsx(S10_res,"Supplementary Table 10.xls")

## Supplementary Table 12
res=matrix(NA,length(plant_antagonist_plant_list),5)
res_all=list()
subgroup=c('Plot-experiment','Pot-experiment')
for (j in 1:length(subgroup)) {
```

```
    for (i in 1:length(plant_antagonist_plant_list)) {
        if(nrow(subset(plant_antagonist_plant_list[[i]],Study.type==subgroup[j]))==0) {res[i,]=NA}
        else {
            res1=escalc(measure="SMD",n1i=plant_antagonist_plant_list[[i]][,13],
                        n2i=plant_antagonist_plant_list[[i]][,10],
                        m1i=plant_antagonist_plant_list[[i]][,11],
                        m2i=plant_antagonist_plant_list[[i]][,8],
                        sd1i=plant_antagonist_plant_list[[i]][,12],
                        sd2i=plant_antagonist_plant_list[[i]][,9],
                        data=plant_antagonist_plant_list[[i]],
                        subset=(Study.type==subgroup[j]),
                        vtype = "UB",append = TRUE)
            if (length(unique(res1$Plant.species))==1) {
                res2=rma.mv(yi, vi,data = res1)
            } else {
                phylodat=myphylo(res1)
                res2=rma.mv(yi, vi,
                            random = list(~ 1 | Plant.species.new,~ 1|Plant.species.new.p),
                            R=list(Plant.species.new.p=phylodat[[2]]),
                            data = phylodat[[1]])
            }
            res[i,]=c(nrow(res1),length(unique(res1$Code)),
                      res2$b,res2$zval,res2$pval)
        }
    }
    res_all[[j]]=res
}
res=do.call('rbind',res_all)
study.type_names=paste(rep(subgroup,each=length(plant_antagonist_name)),
                       rep(plant_antagonist_name,length(subgroup)),sep = '_')
res_study.type<-as.data.frame(res,row.names<-study.type_names)

res=matrix(NA,length(plant_antagonist_plant_list),5)
res_all=list()
subgroup=c('Herbaceous-plant','Woody-plant')
for (j in 1:length(subgroup)) {
    for (i in 1:length(plant_antagonist_plant_list)) {
        if(nrow(subset(plant_antagonist_plant_list[[i]],Plant.type==subgroup[j]))==0) {res[i,]=NA}
        else {
            res1=escalc(measure="SMD",n1i=plant_antagonist_plant_list[[i]][,13],
```

```
                              n2i=plant_antagonist_plant_list[[i]][,10],
                              m1i=plant_antagonist_plant_list[[i]][,11],
                              m2i=plant_antagonist_plant_list[[i]][,8],
                              sd1i=plant_antagonist_plant_list[[i]][,12],
                              sd2i=plant_antagonist_plant_list[[i]][,9],
                              data=plant_antagonist_plant_list[[i]],
                              subset=(Plant.type==subgroup[j]),
                              vtype = "UB",append = TRUE)
      if (length(unique(res1$Plant.species))==1) {
        res2=rma.mv(yi, vi,data = res1)
      } else {
        phylodat=myphylo(res1)
        res2=rma.mv(yi, vi,
                      random = list(~ 1 | Plant.species.new,~ 1|Plant.species.new.p),
                      R=list(Plant.species.new.p=phylodat[[2]]),
                      data = phylodat[[1]])
      }
      res[i,]=c(nrow(res1),length(unique(res1$Code)),
                  res2$b,res2$zval,res2$pval)
    }
  }
  res_all[[j]]=res
}
res=do.call('rbind',res_all)
Plant.type_names=paste(rep(subgroup,each=length(plant_antagonist_name)),
                        rep(plant_antagonist_name,length(subgroup)),sep = '_')
res_plant.type<-as.data.frame(res,row.names<-Plant.type_names)

res=matrix(NA,length(plant_antagonist_plant_list),5)
res_all=list()
subgroup=c('Temperate','Tropical')
for (j in 1:length(subgroup)) {
  for (i in 1:length(plant_antagonist_plant_list)) {
    if(nrow(subset(plant_antagonist_plant_list[[i]],Biome==subgroup[j]))==0) {res[i,]=NA}
    else {
      res1=escalc(measure="SMD",n1i=plant_antagonist_plant_list[[i]][,13],
                              n2i=plant_antagonist_plant_list[[i]][,10],
                              m1i=plant_antagonist_plant_list[[i]][,11],
                              m2i=plant_antagonist_plant_list[[i]][,8],
                              sd1i=plant_antagonist_plant_list[[i]][,12],
```

```
                              sd2i=plant_antagonist_plant_list[[i]][,9],
                              data=plant_antagonist_plant_list[[i]],
                              subset=(Biome==subgroup[j]),
                              vtype = "UB",append = TRUE)
            res1=res1[res1$Outdoor..indoor.or.greenhouse.exp.=='Outdoor',]
            if (length(unique(res1$Plant.species))==1) {
               res2=rma.mv(yi, vi,data = res1)
            } else {
               phylodat=myphylo(res1)
               res2=rma.mv(yi, vi,
                              random = list(~ 1 | Plant.species.new,~ 1|Plant.species.new.p),
                              R=list(Plant.species.new.p=phylodat[[2]]),
                              data = phylodat[[1]])
            }
            res[i,]=c(nrow(res1),length(unique(res1$Code)),
                          res2$b,res2$zval,res2$pval)
         }
   }
   res_all[[j]]=res
}
res=do.call('rbind',res_all)
Climatic_zone_names=paste(rep(subgroup,each=length(plant_antagonist_name)),
                                    rep(plant_antagonist_name,length(subgroup)),sep = '_')
res_Climatic_zone<-as.data.frame(res,row.names<-Climatic_zone_names)

res=rbind(res_study.type,res_plant.type,res_Climatic_zone)
colnames(res)<-c("#Obs","#Studies","Effect size","T-value","P-value")
write.xlsx(res,"Supplementary Table 12.xls")
```


1. Liao, C. et al. Altered ecosystem carbon and nitrogen cycles by plant invasion: A meta-analysis. *New Phytol.* **177**, 706–714 (2008).
2. Wan, N.F. et al. Global synthesis of effects of plant species diversity on trophic groups and interactions. *Nat. Plants*, **6**, 503–510 (2020).
3. Pinheiro, J. C., Bates, D.M. Mixed-effects models in S and S-PLUS. Springer (2000).
4. Pinheiro, J., Bates, D., DebRoy, S., Sarkar, D., R Core Team. nlme: Linear and Nonlinear Mixed Effects Models. R package version 3.1-153 (2021). <URL: https://CRAN.R-project.org/package=nlme>.
5. Fox, J., Weisberg, S. An R Companion to Applied Regression, 3rd Edition. Thousand Oaks, CA (2019). <https://socialsciences.mcmaster.ca/jfox/Books/Companion/index.html>
6. Pappalardo, P., Ogle, K., Hamman, E.A., Bence, J.R., Hungate, B.A., Osenberg, C.W. Comparing traditional and Bayesian approaches to ecological meta-analysis. *Methods Ecol. Evol*. **11**, 1286–1295 (2020).

Supplementary References

The list of 413 studies from which data were extracted for this meta-analysis

1. Abdala-Roberts L, González-Moreno A, Mooney KA, Moreira X, González-Hernández A, Parra-Tabla, V, 2016a. Effects of tree species diversity and genotypic diversity on leafminers and parasitoids in a tropical forest plantation. Agricultural and Forest Entomology, 18: 43-51.
2. Abdala-Roberts L, Mooney KA, 2014. Ecological and evolutionary consequences of plant genotype diversity in a tri-trophic system. Ecology, 95: 2879-2893.
3. Abdala-Roberts L, Mooney KA, Quijano-Medina T, Campos-Navarrete MJ, González-Moreno A, Parra-Tabla V, 2015a. Comparison of tree genotypic diversity and species diversity effects on different guilds of insect herbivores. Oikos, 124: 1527-1535.
4. Abdala-Roberts L, Pratt R, Pratt JD, Mooney KA, 2017. Traits underlying community consequences of plant intra-specific diversity. PLoS ONE, 12: e0183493.
5. Abdala-Roberts L, Terán JCBM, Jorge C., Moreira X, Durán-Yáñez A, Tut-Pech F, 2015b. Effects of pepper (*Capsicum chinense*) genotypic diversity on insect herbivores. Agricultural and Forest Entomology, 17: 433-438.
6. Abdala-Roberts L,Hernández-Cumplido J, Chel-Guerrero L, Betancur-Ancona D, Benrey B, Moreira X, 2016b. Effects of plant intraspecific diversity across three trophic levels: underlying mechanisms and plant traits. American Journal of Botany, 103: 1-9.
7. Abdelaziz BAH, 1983. Effects of cultivar mixtures, fertilizer, and plant density on grain sorghum (*Sorghum bicolor*)/*Striga hermonthica* relations. Weed Science, 31: 552-556.
8. Adam N, Kallenbach M, Meldau S, Veit D, van Dam NM, Baldwin IT, Schuman MC, 2018. Functional variation in a key defense gene structures herbivore communities and alters plant performance. PLoS ONE, 13: e0197221.
9. Ahmed HU, Finckh MR, Alfonso RF, Mundt CC, 1997. Epidemiological effect of gene deployment strategies on bacterial blight of rice. Phytopathology, 87: 66-70.
10. Akanda S, Mundt CC, 1996. Effects of two-component wheat cultivar mixtures on stripe rust severity. Phytopathology, 86: 347-353.
11. Akanda SI, Mundt CC, 1997. Effect of two component cultivar mixtures and yellow rust on yield and yield components of wheat. Plant Pathology, 46: 566-580.
12. Allan RE, Line RF, Peterson CJ, Rubenthaler GL, Rohde CR, 1983. Crew, a multiline wheat cultivar. Crop Science, 23: 1015-1016.
13. Allard RW, 1961. Relationship between genetic diversity and consistency of performance in different environments. Crop Science, 1: 127-133.
14. Allard RW, Adams J, 1969. Population studies in predominantly self-pollinating species. XIII. intergenotypic competition and population structure in barley and wheat. The American Naturalist, 103: 621-645.
15. Altieri M, Schmidt L, 1987. Mixing broccoli cultivars reduces cabbage aphid numbers. California Agriculture, 41: 24-26.
16. Anand SC, Koenning SR, Sharma SB, 1995. Performance of blends of soybean cyst nematode resistant and susceptible cultivars. Crop Science, 35: 524-528.
17. Andrivon D, Lucas JM, Ellissèche D, 2010. Development of natural late blight epidemics in pure and mixed plots of potato cultivars with different levels of partial resistance. Plant Pathology, 52: 586-594.
18. Ashizawa T, Sasahara M, Ohba A, Hori T, Ishikawa K, Sasaki Y, Kuroda T, Harasawa R, Zenbayashi-Sawata K, Koizumi S, 2007. Lesion-based analysis of leaf blast suppression in mixture of rice cultivar and a resistant near-isogenic line. Journal of General Plant Pathology, 73: 15-21.
19. Aslam M, Fischbeck G, 1993. Development of stripe and leaf rusts in wheat cultivar mixtures. Journal of Agronomy and Crop Science, 171: 49-54.
20. Åssveen M, Gunnarstorp T, 1996. Effects of barley cultivar mixture on certain agronomically important characteristics. Norsk Landbruksforsking, 10: 149-158.
21. Atwater DZ, Callaway RM, 2015. Testing the mechanisms of diversity-dependent overyielding in a grass species. Ecology, 96: 3332-3342.
22. Ayanru DKG, Browning JA, 1977. Effect of heterogeneous oat populations on the epiphytotic development of victoria blight. New Phytologist, 79: 613-623.
23. Baker RJ, 1977. Yield of pure and mixed stands of two spring wheat cultivars sown at five rates of seeding. Canadian Journal of Plant Science, 1005-1007.
24. Baniszewski J, Burton A, Kemanian AR, Roth G, 2021. Wheat intraspecific diversity suppressed diseases with subdued yield, economic return and arthropod predation services. Agriculture, Ecosystems and Environment, 315: 107438.
25. Barrera JAS, Frederiksen RA, 1994. Evaluation of sorghum hybrid mixtures for controlling sorghum leaf blight. Plant Disease, 78: 499-503.
26. Barrett JA, Wolfe MS, 1980. Pathogen response to host resistance and its implication in breeding programmes. EPPO Bulletin, 10: 341-347.
27. Barton KE, Valkama E, Vehviläinen H, Ruohomäki K, Knight TF, Koricheva J, 2015. Additive and non-additive effects of birch genotypic diversity on arthropod herbivory in a long-term field experiment. Oikos, 124: 697-706.
28. Beg A, Emery DA, Wynne JC, 1975. Estimation and utilization of inter-cultivar competition in peanuts. Crop Science, 15: 633-637.
29. Blijenburg JG, Sneep J, 1975. Natural selection in a mixture of eight barley varieties, grown in six successive years. 1. competition between the varieties. Euphytica, 24: 305-315.
30. Brim CA, Schutz WM, 1968. Inter-genotypic competition in soybeans II. predicted and observed performance of multiline mixtures. Crop Science, 8: 735-739.
31. Brophy LS, Mundt CC, 1991. Influence of plant spatial patterns on disease dynamics, plant competition and grain yield in genetically diverse wheat populations. Agriculture Ecosystems & Environment, 35: 1-12.

32. Burton GW, 1948. The performance of various mixtures of hybrid and parent inbred pearl millet, *Pennisetum Glaucum* (L.) R. BR. Journal of the American Society of Agronomy, 2: 908-914.
33. Bush L, Slosser JE, Worrall WD, Horner NV, 1991. Potential of wheat cultivar mixtures for green bug (Homoptera: Aphididae) management. Journal of Economic Entomology, 84: 1619-1624.
34. Cadet P, Berry SD, Leslie GW, Spaull VW, 2007. Management of nematodes and a stalk borer by increasing within-field sugarcane cultivar diversity. Plant Pathology, 56: 526-535.
35. Cai T, Peng D, Wang R, Jia X, Qiao D, Liu T, Jia Z, Wang Z, Ren X, 2019. Can intercropping or mixed cropping of two genotypes enhance wheat lodging resistance. Field Crops Research, 239: 10–18.
36. Calonnec A, Goyeau H, de Vallavieille-Pope C, 1996. Effects of induced resistance on infection efficiency and sporulation of Puccinia striiformis on seedlings in varietal mixtures and on field epidemics in pure stands. European Journal of Plant Pathology, 102: 733-741.
37. Campos-Navarrete MJ, Abdala-Roberts L, Munguía-Rosas MA, Parra-Tabla V, 2015a. Are tree species diversity and genotypic diversity effects on insect herbivores mediated by ants? PLOS ONE, 10: e0132671.
38. Campos-Navarrete MJ, Munguía-Rosas MA, Abdala-Roberts L, Quinto J, Parra-Tabla V, 2015b. Effects of tree genotypic diversity and species diversity on the arthropod community associated with big-leaf mahogany. Biotropica, 47: 579-587.
39. Cantelo WW, Sanford LL, 1984. Insect population response to mixed and uniform plantings of resistant and susceptible plant material. Environmental Entomology, 13: 1443-1445.
40. Cao H, Klein AM, Zhu C, Staab M, Durka W, Fischer M, Fornoff F, 2018. Intra- and interspecific tree diversity promotes multitrophic plant-hemiptera-ant interactions in a forest diversity experiment. Basic and Applied Ecology, 29: 89-97.
41. Castagneyrol B, Lagache L, Giffard B, Kremer A, Jactel H, 2012. Genetic diversity increases insect herbivory on oak saplings. PLoS ONE 7: e44247.
42. Caviness CE, Sneller C, 1992. Performance of blends of determinate and indeterminate soybeans. Arkansas farm research, 41: 3-3.
43. Chakraborty S, 1991. Anthracnose development in pure and mixed stands of the pasture legume *Stylosanthes scabra*. Phytopathology, 81: 788-793.
44. Chapman SR, Allard RW, Adams J, 1969. Effect of planting rate and genotypic frequency on yield and seed size in mixtures of two wheat varieties. Crop Science, 9: 575-576.
45. Chateil C, Goldringer I, Taralloa L, Kerbiriou C, Viol IL, Ponge JF, Salmon S, Gachet S, Porcher E, 2013. Crop genetic diversity benefits farmland biodiversity in cultivated fields. Agriculture, Ecosystems & Environment, 171: 25-32.
46. Chaulagain B, Chhetri GBK, Shrestha SM, Sharma S, Sharma-Poudyal D, Lamichhane JR, 2017. Effect of two-component cultivar mixtures on development of wheat yellow rust disease in the field and greenhouse in the Nepal Himalayas. Journal of General Plant Pathology, 83: 131-139.
47. Chen F, Mu L, Wen Z Z, Zhang ZF, Wei ZS, 2018. Study of the effects of mixed sowing of different alfalfa varieties in the Dongting Lake area. Pratacultural Science, 35: 1734-1742 (in Chinese).
48. Chen GL, Song DY, Qi LM, Liu JN, Li YL, Li QF, 2009. Effects of different maize varieties on resistance and yield characters. Shangdong Agricultural Sciences, 6: 46-47 (in Chinese).
49. Chen QC, Zhu YY, Li ZQ, Tang YS, Kang Z, 2009. Effect of wheat cultivar mixtures on wheat yield and stripe rust. Chinese Journ al of Eco-Agriculture, 17: 29-33 (in Chinese).
50. Chen QC, Zhu YY, Li ZQ, Tang YS, Kang ZS, 2008. Effect of wheat cultivar mixtures on severity of stripe rust. Journal of Northwest A&F University (Nat. Sci. Ed.), 36: 119-123 (in Chinese).
51. Cheng LG, Li AD, Hu FY, Wang DL, 2008. Preliminary study on mixed sowing cultivation technique of different rice varieties. Hunan Agricultural Sciences, 3: 29-30 (in Chinese).
52. Chin KM, Wolfe MS, 1984a. Selection on *Erysiphe graminis* in pure and mixed stands of barley. Plant Pathology, 33: 535-546.
53. Chin KM, Wolfe MS, 1984b. The spread of *Erysiphe graminis* f. sp. *hordei* in mixtures of barley varieties. Plant Pathology, 33: 89-100.
54. Chowdhury AR, Hodgson DR, 1982. Growth and yield in pure and mixed crops of potato cultivars. The Journal of Agricultural Science, 98: 505-516.
55. Clay RE, Allard RW, 1969. A comparison of the performance of homogeneous and heterogeneous barley populations. Crop Science, 9: 407-412.
56. Collins AR, Beckage B,Molofsky J, 2018. Small-scale genotypic richness stabilizes plot biomass and increases phenotypic variance in the invasive grass *Phalaris arundinacea*. Journal of Plant Ecology, 11: 47-55.
57. Cook-Patton SC, Mcart SH, Parachnowitsch AL, Thaler JS, Agrawal AA, 2011. A direct comparison of the consequences of plant genotypic and species diversity on communities and ecosystem function. Ecology, 92: 915-923.
58. Cowger C, Mundt CC, 2002. Effects of wheat cultivar mixtures on epidemic progression of *Septoria tritici* blotch and pathogenicity of *Mycosphaerella graminicola*. Phytopathology, 92: 617-623.
59. Cowger C, Weisz R, 2008. Winter wheat blends (mixtures) produce a yield advantage in North Carolina. Agronomy Journal, 100: 169-177.
60. Cox AE, Inns DF, 1974. Trials with mixed varieties of spring barley in south-west England 1970-72. Experimental Husbandry, 26: 90-95.
61. Cox CM, Garrett KA, Bowden RL, Fritz AK, Dendy SP, Heer WF, 2004. Cultivar mixtures for the simultaneous management of multiple diseases: tan spot and leaf rust of wheat. Phytopathology, 94: 961-969.
62. Crawford KM, Crutsinger GM, Sanders NJ, 2007. Host-plant genotypic diversity mediates the distribution of an ecosystem engineer. Ecology, 88: 2114-2120.
63. Crawford KM, Rudgers JA, 2013. Genetic diversity within a dominant plant outweighs plant species diversity in structuring an arthropod community. Ecology, 94: 1025-1035.
64. Crawford KM, Whitney KD, 2010. Population genetic diversity influences colonization success. Molecular Ecology, 19: 1253-

1263.
65. Creissen HE, Jorgensen TH, Brown JKM, 2013. Stabilization of yield in plant genotype mixtures through compensation rather than complementation. Annals of Botany, 112: 1439-1447.
66. Creissen HE, Jorgensen TH, Brown JKM, 2016. Increased yield stability of field-grown winter barley (*Hordeum vulgare* l.) varietal mixtures through ecological processes. Crop Protection, 85: 1-8.
67. Crutsinger GM, Collins MD, Fordyce JA, Gompert Z, Nice CC, Sanders NJ, 2006. Plant genotypic diversity predicts community structure and governs an ecosystem process. Science, 313: 966-967.
68. Crutsinger GM, Collins MD, Fordyce JA, Sanders NJ, 2008a. Temporal dynamics in non-additive responses of arthropods to host-plant genotypic diversity. Oikos, 117: 255-264.
69. Crutsinger GM, Reynolds WN, Classen AT, Sanders NJ, 2008b. Disparate effects of plant genotypic diversity on foliage and litter arthropod communities. Oecologia, 158: 65-75.
70. Crutsinger GM, Souza L, Sanders NJ, 2008c. Intraspecific diversity and dominant genotypes resist plant invasions. Ecology Letters, 11: 16-23.
71. Dahlin I, Kiær LP, Bergkvist G, Weih M, Ninkovic V, 2020. Plasticity of barley in response to plant neighbors in cultivar mixtures. Plant and Soil, 447: 537-551.
72. Dahlin I, Rubene D, Glinwood R, Ninkovic V, 2018. Pest suppression in cultivar mixtures is influenced by neighbor-specific plant-plant communication. Ecological Applications, 28: 2187-2196.
73. Dai J, Wiersma JJ, Holen DL, 2012. Performance of hard red spring wheat cultivar mixtures. Agronomy Journal, 104: 17-21.
74. Darras S, Mckenzie RH, Olson MA, Willenborg CJ, 2015. Influence of genotypic mixtures on field pea yield and competitive ability. Canadian Journal of Plant Science, 95: 315-324.
75. Davidson JL, Jones DB, Christian KR, 1990. Winter feed production and grain yield in mixtures of spring and winter wheats. Australian Journal of Agricultural Research, 41: 1-18.
76. Didelot F, Brun L, Parisi L, 2007. Effects of cultivar mixtures on scab control in apple orchards. Plant Pathology, 56, 1014-1022.
77. Dileone JA, Mundt CC, 1994. Effect of wheat cultivar mixtures on populations of *Puccinia striiformis* races. Plant Pathology, 43: 917-930.
78. Doherty EM, Meagher RL, Dale AG, 2019. Turfgrass cultivar diversity provides associational resistance in the absence of pest resistant cultivars. Environmental Entomology, 48: 623-632.
79. Drummond EBM, Vellend M, 2012. Genotypic diversity effects on the performance of *Taraxacum officinale* populations increase with time and environmental favorability. PLoS ONE 7(2): e30314.
80. Dubin HJ, Wolfe MS, 1994. Comparative behavior of three wheat cultivars and their mixture in India, Nepal and Pakistan. Field Crops Research, 39: 71-83.
81. Dubs F, Vergnes A, Mirlicourtois E, Viol IL, Kerbiriou C, Goulnik J, Belghali S, Bentze L, Barot S, Porcher E, 2018. Positive effects of wheat variety mixtures on aboveground arthropods are weak and variable. Basic and Applied Ecology, 33: 66-78.
82. Dunn JH, Ervin EH, Fresenburg BS, 2002. Turf performance of mixtures and blends of tall fescue, Kentucky bluegrass, and perennial ryegrass. Hortscience, 37: 214-217.
83. Eagles CF, 1983. Relationship between competitive ability and yielding ability in mixtures and monocultures of populations of *Dactylis glomerata* L. Grass and Forage Science, 38: 21-24.
84. Early HL, Qualset CO, 1971. Complementary competition in cultivated barley (*Hordeum vulgare* L.). Euphytica, 20: 400-409.
85. Eberhart SA, Penny LH, Sprague GF, 1964. Intra-plot competition among maize single crosses. Crop Science, 4: 467-471.
86. Ehlers A, Worm B, Reusch T, 2008. Importance of genetic diversity in eelgrass *Zostera marina* for its resilience to global warming. Marine Ecology Progress Series, 355, 1-7.
87. Ellis PR, Pink DAC, Ramsey AD, 1994. Inheritance of resistance to lettuce root aphid in the lettuce cultivars 'Avoncrisp' and 'Lakeland'. Annals of Applied Biology, 124: 141-151.
88. Emery DA, Benson JA, Wynne JC, 1970. Four empirical experiments with Virginia peanut seed mixtures. Oleagineux, 25: 275-378.
89. Erskine W, 1977. Adaptation and competition in mixtures of cowpea (Vigna unguiculata, (L.) Walp.). Euphytica, 26: 193-202.
90. Essah SYC, Stoskopf NC, 2002. Mixture performance of phenotypically contrasting barley cultivars. Canadian Journal of Plant Science, 82: 1-6.
91. Fang H, Zhou JH, Wang YY, Zhou HP, He XH, Sun Y, Yang LH, Zhu YY, 2007. Optimizing cultivation patterns for rice blast control. Scientia Agricultura Sinica, 40: 916-924 (in Chinese).
92. Fang Y, Xu B, Liu L, Gu Y, Liu Q, Turner NC, Li FM, 2014. Does a mixture of old and modern winter wheat cultivars increase yield and water use efficiency in water-limited environments? Field Crops Research, 156: 12-21.
93. Fe HR, Edwin H, Lianqing Z, Francisco E, Duque JLJE, Mundt CC, Cruz CMV, Garrett KA, 2017. Cropping system diversification for food production in Mindanao rubber plantations: a rice cultivar mixture and rice intercropped with mungbean. PeerJ, 5: e2975.
94. Feaster CV, Turcotte EL, 1973. Yield stability in doubled haploids of American pima cotton. Crop Science, 13: 232-233.
95. Fehr WR, de Cianzio SR, 1980. Relationship of component frequency to compensatory response in soybean blends. Crop Science, 20: 392-393.
96. Fehr WR, Rodriguez SR, 1974. Effect of row spacing and genotypic frequency on the yield of soybean blends. Crop Science, 14: 521-525.
97. Fernandez-Conradi P, Jactel H, Hampe A, Leiva MJ, Castagneyrol B, 2017. The effect of tree genetic diversity on insect herbivory varies with insect abundance. Ecosphere, 8: e01637.
98. Field E, Schönrogge K, Barsoum N, Hector A, Gibbs M, 2019. Individual tree traits shape insect and disease damage on oak in a climate-matching tree diversity experiment. Ecology and Evolution, 9: 8524-8540.
99. Finckh MR, 1992. Stripe rust, yield, and plant competition in wheat cultivar mixtures. Phytopathology, 82: 905-913.
100. Finckh MR, Gacek EC, Czembor HJ, Wolfe MS, 1999. Host frequency and density effects on powdery mildew and yield in mixtures of barley cultivars. Plant Pathology, 48: 807-816.

101. Finckh MR, Mundt CC, 1992. Plant competition and disease in genetically diverse wheat populations. Oecologia, 91: 82-92.
102. Finckh MR, Mundt CC, 1993. Effects of stripe rust on the evolution of genetically diverse wheat populations. Theoretical and Applied Genetics, 85: 809-821.
103. Finckh MR, Mundt CC, 1996. Temporal dynamics of plant competition in genetically diverse wheat populations in the presence and absence of stripe rust. Journal of Applied Ecology, 33: 1041-1052.
104. Fletchera A, Ogden G, Sharma D, 2019. Mixing it up-wheat cultivar mixtures can increase yield and buffer the risk of flowering too early or too late. European Journal of Agronomy, 103: 90-97.
105. Frankel OH, 1939. Analytical yield investigations on new Zealand wheat: IV. blending varieties of wheat. Journal of Agricultural Science, 29: 249-261.
106. Frey KJ, Maldonado U, 1967. Relative productivity of homogeneous and heterogeneous oat cultivars in optimum and suboptimum environments. Crop Science, 7: 532-535.
107. Fridley JD, Grime JP, 2010. Community and ecosystem effects of intraspecific genetic diversity in grassland microcosms of varying species diversity. Ecology, 91: 2272-2283.
108. Fu ZL, Chu ZT, 1994. Effect of wheat hybrid cultivation on yield increase. Tillage and Cultivation, 5: 12-15 (in Chinese).
109. Gallandt ER, Dofing SM, Reisenauer PE, Donaldson E, 2001. Diallel analysis of cultivar mixtures in winter wheat. Crop Science, 41: 792-796.
110. Gan DY, Luo LC, Weng QY, He YX, 1995. Suppression of rice blast disease by mixed planting of high-quality rice with resistant varieties. Journal of Fujian Academy of Agricultural Sciences, 10: 48-51 (in Chinese).
111. Gan DY, Weng QY, Luo LC, Huang JP, 1994. Studies on Controlling Rice Blast by Mix-culture of Resistant with Susceptible Hybrid Rice Varieties (ReportI). Journal of Fujian Academy of Agricultural Sciences, 9: 28-30 (in Chinese).
112. Gardner AL, Hunt IV, 1963. Inter-varietal competition in perennial ryegrass swards. Journal of British Grassland Society, 18: 285-291.
113. Genung MA, Lessard J-P, Brown CB, Bunn WA, Cregger MA, Reynolds WN, Felker-Quinn E, Stevenson ML, Hartley AS, Crutsinger GM, Schweitzer JA, Bailey JK, 2010. Non-additive effects of genotypic diversity increase floral abundance and abundance of floral visitors. PLoS ONE 5: e8711.
114. Gieffers W, Hesselbach J, 1988a. Disease incidence and yield of different cereal cultivars in pure stands and mixtures. I. Spring barley (*Hordeum vulgare* L.). Journal of Plant Diseases and Protection, 95: 46-62.
115. Gieffers W, Hesselbach J, 1988b. Disease incidence and yield of different cereal cultivars in pure stands and mixtures. III. Winter wheat (*Triticum aestivum* L.). Journal of Plant Diseases and Protection, 95: 182-192.
116. Gigot C, Saint-Jean S, Huber L, Maumené C, Leconte M, Kerhornou B, de Vallavieille-Pope C, 2013. Protective effects of a wheat cultivar mixture against splash-dispersed *Septoria tritici* blotch epidemics. Plant Pathology, 62: 1011-1019.
117. Gill KS, Kumar V, Nanda GS, 1977. Multilines in wheat 4. performance of the multilines and components of tv 18'. Indian Journal of Genetics & Plant Breeding, 37: 388-394.
118. Gizlice Z, Carter TE, Burton JW, 1989. The impact of maturity and genotype on blend performance in group v and group vii soybean cultivars. Agronomy Journal, 81: 559-562.
119. Gold CS, 1994. The effects of cropping systems on cassava whiteflies in colombia: implications for control of African cassava mosaic virus disease. African Crop Science Journal, 2: 423-436.
120. Gold CS, Altieri MA, Bellotti AC, 1989a. Effects of cassava varietal mixtures on the whiteflies *Aleurotrachelus socialis* and *Triateurodes variabilis* in Colombia. Entomologia Experimentalis et Applicata, 53: 195-202.
121. Gold CS, Altieri MA, Bellotti AC, 1989b. Effects of intercrop competition and differential herbivore numbers on cassava growth and yields. Agriculture Ecosystems & Environment, 26: 131-146.
122. Gold CS, Altieri MA, Bellotti AC, 1989c. The effects of intercropping and mixed varieties of predators and parasitoids of cassava whiteflies (Hemiptera: Aleyrodidae) in Colombia. Bulletin of Entomological Research, 79: 115-121.
123. Gold CS, Altleri MA, Bellotti AC, 1990. Effects of intercropping and varietal mixtures on the cassava hornworm, *Erinnyis ello* L. (Lepidoptera: Sphingidae), and the stemborer, *Chilomima clarkei* (Amsel) (Lepidoptera: Pyralidae), in Colombia. International Journal of Pest Management, 36: 362-367.
124. Grettenberger IM, Tooker JF, 2016. Inter-varietal interactions among plants in genotypically diverse mixtures tend to decrease herbivore performance. Oecologia, 182: 189-202.
125. Grettenberger IM, Tooker JF, 2017. Variety mixtures of wheat influence aphid populations and attract an aphid predator. Arthropod-Plant Interactions, 11: 133-146.
126. Grettenberger IM, Tooker JF, 2020. Cultivar mixtures of soybeans have inconsistent effects on herbivore and natural-enemy populations. Agriculture, Ecosystems and Environment, 292:106835.
127. Grossman JJ, Cavender-Bares J, 2019. Consequences of biodiversity shift across phylogenetic scales for aspen and willow growth, survival, and herbivory. Journal of Vegetation Science, 30: 301-311.
128. Grümmer G, Roy SK, 1966. Intervarietal mixtures of rice and incidence of brown-spot disease (*Helminthosporium oryzae* Breda de Haan). Nature, 209: 1265-1267.
129. Gubbels GH, Kenaschuk EO, 1987. Performance of pure and mixed stands of flax cultivars. Canadian Journal of Plant Science, 67: 797-802.
130. Guo X, Petermann JS, Schittko C, Wurst S, 2016. Root-knot nematodes (*Meloidogyne hapla*) can modify the positive plant intraspecific diversity-productivity effect on red clover in clover-grass communities. Applied Soil Ecology, 102: 26-35.
131. Gustafsson Å, 1953. The cooperation of genotypes in barley. Hereditas, 39: 1-18.
132. Haghshenas A, Emam Y, Ghadiri H, Kazemeini SA, Kamgarhaghighi AA, 2013. Effect of mixed cropping of an early- and a middle-ripening wheat cultivar on mitigation of competition during post-anthesis moisture stress. Journal of Agricultural Science & Technology, 15: 491-503.
133. Hahn CZ, Niklaus PA, Bruelheide H, Michalski SG, Shi M, Yang X, Zeng X, Fischer M, Durka W, 2017. Opposing intraspecific vs. interspecific diversity effects on herbivory and growth in subtropical experimental tree assemblages. Journal of

Plant Ecology, 10: 242-251.
134. Hamback PA, Bjorkman M, Hopkins RJ, 2010. Patch size effects are more important than genetic diversity for plant-herbivore interactions in brassica crops. Ecological Entomology, 35: 299-306.
135. Hamblin J, 1975. Effect of environment, seed size and competitive ability on yield and survival of *Phaseolus vulgaris* (L.) genotypes in mixtures. Euphytica, 24: 435-445.
136. Han G, Lang J, Sun Y, Wang YY, Zhu YY, Lu BR, 2016. Intercropping of rice varieties increases the efficiency of blast control through reduced disease occurrence and variability. Journal of Integrative Agriculture, 15: 795-802.
137. Han HH, Zhou YJ, Chen X, Yu LQ, 2007. Inhibitory effects of mixed-planting of rice varieties with different weed-tolerant potentials on *Echinochloa crus-galli*. Chinese Journal of Rice Sciences, 21: 319-322 (in Chinese).
138. Hanson AA, Garber RJ, Myers WM, 1952. Yields of individual and combined apomictic strains of Kentucky bluegrass (*Poa pratensis* L.). Agronomy journal, 44: 125-128.
139. Hariri D, Fouchard M, Prud'homme H, 2001. Incidence of soil-borne wheat mosaic virus in mixtures of susceptible and resistant wheat cultivars. European Journal of Plant Pathology, 107: 625-631.
140. Harrabi MM, Larlbl A, Bouslama M, 1986. Stability and yield performance of some barley (*Hordeurn vulgare* L.) cultivars and mixtures. RACHIS, 5: 11-14.
141. Hartmann RW, Allard RW, 1964. Effect of nutrient and moisture levels on competitive ability in barley (*Hordeum vulgare* L.). Crop Science, 4: 424-426.
142. Hartwig EE, Epps JM, Buehring N, 1982. Response of resistant and susceptible soybean cultivars to continuous cropping in area infested with cyst nematode. Plant Disease, 66: 18-20.
143. Hartwig EE, Young LD, Buehring N, 1987. Effects of monocropping resistant and suspectible soybean cultivars on cyst nematode infested soil. Crop Science, 27: 576-579.
144. Harveson RM, Rush CM, 2002. The influence of irrigation frequency and cultivar blends on the severity of multiple root diseases in sugar beets. Plant Disease, 86: 901-908.
145. Hauri KC, Glassmire AE, Wetzel WC, 2021. Chemical diversity rather than cultivar diversity predicts natural enemy control of herbivore pests. Ecological Applications, 31: e02289.
146. Helland SJ, Holland JB, 2001. Blend response and stability and cultivar blending ability in oat. Crop Science, 41: 1689-1696.
147. Hoekstra GJ, Kannenberg LW, Christie BR, 1985a. Grain yield comparison of pure stands and equal proportion mixtures for seven hybrids of maize. Canadian Journal of Plant Science, 65: 471-479.
148. Hoekstra GJ, Kannenberg LW, Christie BR, 1985b. Grain yield comparison of pure stands and mixtures of different proportions for two hybrids of maize. Canadian Journal of Plant Science, 65: 481-485.
149. Horner A, Browett SS, Antwis RE, 2019. Mixed-cropping between field pea varieties alters root bacterial and fungal communities. Scientific Reports, 9: 16953.
150. Hovick SM, Whitney KD, 2019. Propagule pressure and genetic diversity enhance colonization by a ruderal species: a multi-generation field experiment. Ecological Monographs, 89: e01368.
151. Hu X, Xu YL, Li SX, Gao SJ, 2012. Effect of cultivar mixture on growth and developoment of soybean inoculated with soybean cyst nematode. Soybean Science, 31: 449-452 (in Chinese).
152. Huang C, Sun Z, Wang H, Luo Y, Ma Z, 2011. Spatiotemporal effects of cultivar mixtures on wheat stripe rust epidemics. European Journal of Plant Pathology, 131: 483-496.
153. Huang R, Kranz J, Welz HG, 1995. Increase of complex pathotypes of *Erysiphe graminis* f. sp. *hordei* in two-component mixtures of spring barley cultivars. Journal of Phytopathology, 143: 281-286.
154. Hughes AR, 2014. Genotypic diversity and trait variance interact to affect marsh plant performance. Journal of Ecology, 102: 651-658.
155. Hughes AR, Best RJ, Stachowicz JJ, 2010. Genotypic diversity and grazer identity interactively influence seagrass and grazer biomass.Marine Ecology Progress Series, 403: 43-51.
156. Hughes AR, Hanley TC, Schenck FR, Hays CG, 2016. Genetic diversity of seagrass seeds influences seedling morphology and biomass. Ecology, 97: 3538-3546.
157. Hughes AR, Stachowicz JJ, 2004. Genetic diversity enhances the resistance of a seagrass ecosystem to disturbance. Proceedings of the National Academy of Sciences of the United States of America, 101: 8998-9002.
158. Hughes AR, Stachowicz JJ, 2011. Seagrass genotypic diversity increases disturbance response via complementarity and dominance. Journal of Ecology, 99: 445–453.
159. Ibenthal WD, von Meier zu Beerentrup H, Nabizadeh F, 1985. Levels of yield and disease in cultivar mixtures of spring barley. Journal of Plant Diseases and Protection, 92: 37-46.
160. Ichinose K, Reddy GVP, Shrestha G, Sharma A, Okada Y, Yoshida M, Sakai T, 2019. Interplanting different varieties of a sweet potato crop to reduce damage by oligophagous insect pests. Annals of the Entomological Society of America, 112: 365-371.
161. Innes NL, Jones GB, 1977. Performance of seed mixtures and multilines of upland cotton in Uganda. Journal of Agricultural Science, 88: 47-54.
162. Ito PJ, Hamilton RA, 1980. Quality and yield of 'Keauhou' macadamia nuts from mixed and pure block plantings. HortScience, 15: 307-307.
163. Jackson LF, Wennig RW, 1997. Use of wheat cultivar blends to improve grain yield and quality and reduce disease and lodging. Field Crops Research, 52: 261-269.
164. Jacques S, Bacon R, Parsch L, 1997. Comparison of single cropping, relay cropping and double cropping of soyabeans with wheat using cultivar blends. Experimental Agriculture, 33: 477-486.
165. Jedel PE, Helm JH, Burnett PA, 1998. Yield, quality and stress tolerance of barley mixtures in central alberta. Canadian Journal of Plant Science, 78: 429-436.
166. Jeger MJ, Jones DG, Griffiths E, 1983. Disease spread of non-specialised fungal pathogens from inoculated point sources in intraspecific mixed stands of cereal cultivars. Annals of Applied Biology, 102: 237-244.

167. Jeger MJ, Jones DG, Griffiths E, 2008. Disease progress of non-specialised fungal pathogens in intraspecific mixed stands of cereal cultivars. II. field experiments. Annals of Applied Biology, 98: 199-210.
168. Jennings PR, Aquino RC, 1968. Studies on competition in rice. III. the mechanism of competition among phenotypes. Evolution, 22: 529-542.
169. Jensen NF, 1952. Intra-varietal diversification in oat breeding. Agronomy journal, 44: 30-34.
170. Jiang X, Wang L, Qiao Y, Li X, Xue Y, Zhang R, Xia H, 2021. Effects of mixed culture on yield，agronomic characters and grain zinc content of eight winter wheat cultivars mainly popularized in the Huanghe-Huaihe-Haihe Region. Shandong Agricultural Sciences, 53: 34-40 (in Chinese).
171. Jiang YT, Xu T, Duan XY, Zhou YL, 2015. Effect of variety mixture planting on powdery mildew controlling as well as yield and protein contents in common wheat. Acta Agronomica Sinica, 41: 276-285 (in Chinese).
172. Johnson MTJ, Lajeunesse MJ, Agrawal AA, 2006. Additive and interactive effects of plant genotypic diversity on arthropod communities and plant fitness. Ecology Letters, 9: 24-34.
173. Jokinen K, 1991a. Assessment of competition and yield advantage in addition series of barley variety mixtures. Journal of Agricultural Science in Finland, 63: 307-320.
174. Jokinen K, 1991b. Yield and competition in barley variety mixtures. Journal of Agricultural Science in Finland, 63: 287-305.
175. Jones TS, Allan E, Härri SA, Krauss J, Müller CB, van Veen FJF, 2011. Effects of genetic diversity of grass on insect species diversity at higher trophic levels are not due to cascading diversity effects. Oikos, 120: 1031-1036.
176. Juskiw PE, Helm JH, Burnett PA, 2001. Three-component barley mixtures: ratio effects in replacement series. Canadian Journal of Plant Science, 81: 651-656.
177. Juskiw PE, Helm JH, Salmon DF, 2000a. Competitive ability in mixtures of small grain cereals. Crop Science, 40: 159-164.
178. Juskiw PE, Helm JH, Salmon DF, 2000b. Forage yield and quality for monocrops and mixtures of small grain cereals. Crop Science, 40: 138-147.
179. Kabululu MS, Ojiewo C, Oluoch M, Maass BL, 2014. Cowpea cultivar mixtures for stable and optimal leaf and seed yields in a maize intercropping system. International Journal of Vegetable Science, 20: 270-284.
180. Kannenberg LW, Hunter RB, 1972. Yielding ability and competitive influence in hybrid mixtures of maize. Crop Science, 12: 274-277.
181. Kapur R, Bhat SR, Tripathi BK, 1988. Performance of varietal mixtures in sugarcane. Experimental Agriculture, 24: 163-168.
182. Karjalainen R, 1986. Spring wheat mixtures in northern crop production: ability of mixtures to buffer disease development and yield loss caused by *Septoria nodorum*. Journal of Agricultural Science in Finland, 58: 33-42.
183. Karjalainen R, Peltonen-Sainio P, 1993. Effect of oat cultivar mixtures on disease progress and yield reduction caused by barley yellow dwarf virus. Journal of Plant Diseases and Protection, 100: 58-68.
184. Kathju S, Garg BK, Vyas SP, Lahiri AN, 2003. Sustainable production of moth bean through genotype management under arid environments. Journal of Arid Environments, 53: 137-143.
185. Kaut AHEE, Mason HE, Navabi A, O'Donovan JT, Spaner D, 2009. Performance and stability of performance of spring wheat variety mixtures in organic and conventional management systems in western Canada. Journal of Agricultural Science, 147: 141-153.
186. Khalifa MA, Qualset CO, 1974. Intergenotypic competition between tall and dwarf wheats. I. in mechanical mixtures. Crop Science, 14: 795-799.
187. Kiær LP, Skovgaard IM, Østergård H, 2012. Effects of inter-varietal diversity, biotic stresses and environmental productivity on grain yield of spring barley variety mixtures. Euphytica, 185: 123-138.
188. Kiyosawa S, Shiyomi M, 1972. A theoretical evaluation of the effect of mixing resistant variety with susceptible variety for controlling plant diseases. Annals of the Phytopathological Society of Japan, 38: 41-51.
189. Knauft DA, Gorbet DW, 1991. Agronomic performance and genetic shifts of genotype mixtures in peanut. Euphytica, 52: 85-90.
190. Knott EA, Mundt CC, 1990. Mixing ability analysis of wheat cultivar mixtures under diseased and nondiseased conditions. Theoretical and Applied Genetics, 80: 313-320.
191. Knowles RP, 1979. Comparison of cultivar hybrids and blends with pure cultivars in crested wheatgrass. Canadian Journal of Plant Science, 59: 1019-1023.
192. Koizumi S, Kato H, 1987. Effect of mixed plantings of susceptible and resistant rice varieties on leaf blast development. Annals of the Phytopathological Society of Japan, 53: 28-38.
193. Kollberg I, Weih M, Glynn C, 2021. The effect of willow diversity on insect herbivory and predation. Agricultural and Forest Entomology, DOI: 10.1111/afe.12466
194. Kølster P, Munk L, Stølen O, 1989. Disease severity and grain yield in barley multilines with resistance to powdery mildew. Crop Science, 29: 1459-1463.
195. Kotowska AM, Jr JFC, Keddie BA, 2010. Plant genetic diversity yields increased plant productivity and herbivore performance. Journal of Ecology, 98: 237-245.
196. Kousik CS, Sanders DC, Ritchie DF, 1996. Mixed genotypes combined with copper sprays to manage bacterial spot of bell peppers. Phytopathology, 86: 502-508.
197. Kraska P, Andruszczak S, Staniak M, Kwiecińska-Poppe E, Różyło K, Rusecki H, 2018. Evaluation of chemical composition of lentil seeds in sole crop and row intercropped with naked oats in an organic farm. Applied Ecology and Environmental Research, 16: 1855-1867.
198. Lai GS, 1986. Mixing different sugarcane varieties at the same ripening stage can increase yield. Crops, 2: 32-33 (in Chinese).
199. Lai R, You M, Zhu C, Gu G, Lin Z, Liao L, Lin L, Zhong X, 2017. *Myzus persicae*, and aphid-transmitted viral disease control via variety intercropping in flue-cured tobacco. Crop Protection, 100: 157-162.
200. Lang RW, Holmes JC, Taylor BR, Waterson HA, 1975. The performance of barley variety mixtures. Experimental Husbandry,

28: 53-59.
201. Lannou C, Hubert P, Gimeno C, 2005. Competition and interactions among stripe rust pathotypes in wheat-cultivar mixtures. Plant Pathology, 54: 699-712.
202. Lannou C, Vallavieille-Pope CD, Biass C, Goyeau H, 1994. The efficacy of mixtures of susceptible and resistant hosts to two wheat rusts of different lesion size: controlled condition experiments and computerized simulations. Journal of Phytopathology, 140: 227-237.
203. Lazzaro M, Costanzo A, Bàrberi P, 2018. Single *vs* multiple agroecosystem services provided by common wheat cultivar mixtures: weed suppression, grain yield and quality. Field Crops Research 221: 277-297.
204. Lee JA, 1960. A study of plant competition in relation to development. Evolution, 14: 18-28.
205. Leon J, Diepenbrock W, 1987. Yielding ability of pure stands and equal proportion blends of rapeseed (*Brassica napus* L.) with double-low quality. Journal of Agronomy & Crop Science, 159: 82-89.
206. Lepiarczyk A, Abza T, Puzynska K, 2010. Productivity of winter wheat Turnia and Rysa cultivars sown in pure and mixed stand depending on tillage systems. Annales Universitatis Mariae Curie- Skłodowska Lubin- Polonia, 65: 42-50.
207. Letourneau DK, 1995. Associational susceptibility: effects of cropping pattern and fertilizer on Malawian bean fly levels. Ecological Applications, 5: 823-829.
208. Li CH, Su XH, Sun DL, 2002. Ecophysiological characterization of different maize (*Zea mays* L.) genotypes under mono-or inter-cropping conditions. Acta Ecologica Sinica, 22: 2096-2013 (in Chinese).
209. Li H, Liu Q, Li Y, Song W, Liu C, 2020. Intercropping and mixed cropping of wheat varieties: effects on wheat aphid occurrence. Chinese Agricultural Science Bulletin, 36: 135-142 (in Chinese)..
210. Li M, Li R, Zhang J, Liu S, Qiu S, 2019. A combination of rice cultivar mixed-cropping and duck co-culture suppressed weeds and pests in paddy fields. Basic and Applied Ecology, 40: 67-77.
211. Li M, Li R, Zhang J, Wu T, Liu S, Hei Z, Qiu S, 2020. Effects of the integration of mixed-cropping and rice-duck co-culture on rice yield and soil nutrients in southern china. Journal of the Science of Food and Agriculture, 100: 277-286.
212. Li M, Zhang J, Liu S, Ashraf U, Zhao B, Qiu S, 2019. Mixed-cropping systems of different rice cultivars have grain yield and quality advantages over mono-cropping systems. Journal of the Science of Food and Agriculture, 99: 3326-3334.
213. Li MJ, Zhou N, Zhang JE, Xiang HM, Liang KM, 2017. Effect of rice varieties mixed-cropping with duck raising on nutrient dynamics in paddy soils. Chinese Journal of Eco-Agriculture, 25: 211-220 (in Chinese).
214. Li SF, Rao WF, 2001. Effects of mixed planting on main agronomic characters and yield of Rapeseed. Tillage and Cultivation, 2: 18-19 (in Chinese).
215. Li Z, Wan G, Wang L, Parajulee MN, Zhao Z, Chen F, 2018. Effects of seed mixture sowing with resistant and susceptible rice on population dynamics of target planthoppers and non-target stemborers and leaffolders. Pest management science, 74: 1664-1676.
216. Liu EM, Zhu YY, Liu XM, Zhang SY, Liu AM, Ye HZ, 2002. Studies on control of rice blast by alternatively planting diverse rice varieties in a hilly region. Crop Research, 16: 7-10 (in Chinese).
217. Liu GJ, Chen SG, Wang JY, Shen JH, Sogawa K, Xie XM, Qiao QC, Pu ZG, Shi DG, Liu XG, 2013. Preliminary study on suppression of the whitebacked planthopper, *Sogatella furcifera* by cultivating the mixture of the resistant and susceptible rice varieties. Chinese Journal of Rice Sciences, 17: 103-107 (in Chinese).
218. Liu YB, Mccreight JD, 2006. Responses of *Nasonovia ribisnigri* (Homoptera: Aphididae) to susceptible and resistant lettuce. Journal of Economic Entomology, 99: 972-978.
219. Ma JH, Zhao ZH, Zhang R, 2017. Sowing a mixture of alfalfa modulates the population density of alfalfa pests. density of alfalfa pests. Pratacultural Science, 34: 2521-2527 (in Chinese).
220. Mahmood T, Marshall D, McDaniel ME, 1991. Effect of winter wheat cultivar mixtures on leaf rust severity and grain yield. Phytopathology, 81: 470-474.
221. Mansion-Vaquié A, Wezel A, Ferrer A, 2019. Wheat genotypic diversity and intercropping to control cereal aphids. Agriculture, Ecosystems & Environment, 285: 106604.
222. Manthey R, Fehrmann H, 1993. Effect of cultivar mixtures in wheat on fungal diseases, yield and profitability. Crop Protection, 12: 63-68.
223. Mao JH, He M, He ZQ, 1991.Effect of mixed plantings of resistant and susceptible varieties of rice on disease development. Acta Phytopathologica Sinica, 21: 155-160 (in Chinese).
224. Marshall DR, Allard RW, 1974. Performance and stability of mixtures of grain sorghum I. relationship between level of genetic diversity and performance. Theoretical and Applied Genetics, 44: 145-152.
225. Mateu MG, Yarwood SA, Yarwood AH, 2021. Positive interactions occur between *Phragmites australis* lineages across short term experimental nutrient regimes. Aquatic Botany, 172: 103382.
226. M'Barek SB, Karisto P, Abdedayem W, Laribi M, Fakhfakh M, Kouki H, Mikaberidze A, Yahyaoui A, 2020. Improved control of septoria tritici blotch in durum wheat using cultivar mixtures. Plant Pathology, 69: 1655-1665.
227. Mcart SH, Cook-Patton SC, Thaler JS, 2012. Relationships between arthropod richness, evenness, and diversity are altered by complementarity among plant genotypes. Oecologia, 168: 1013-1021.
228. McArt SH, Thaler JS. 2013. Plant genotypic diversity reduces the rate of consumer resource utilization. Proceedings of the Royal Society B: Biological Sciences, 280: 20130639.
229. Midmore DJ, Alcazar J, 1991. Mixed planting of potato cultivars: growth, yield and leafminer damage in the cool tropics. Experimental Agriculture, 27: 305-318.
230. Mille B, Jouan B, 1997. Influence of varietal associations on the development of leaf and glume blotch and brown leaf rust in winter bread wheat. Agronomie, 17: 247-251.
231. Montazeaud G, Violle C, Roumet P, Rocher A, Ecarnot M, Compan F, Maillet G, Fort F, Fréville H, 2020. Multifaceted functional diversity for multifaceted crop yield: Towards ecological assembly rules for varietal mixtures. Journal of Applied Ecology, 57: 2285-2295.

232. Moreira X, Abdala-Roberts L, Parra-Tabla V, Mooney, KA, 2014. Positive effects of plant genotypic and species diversity on anti-herbivore defenses in a tropical tree species. PLoS ONE, 9: e105438.
233. Moreira X, Mooney KA. 2013, Influence of plant genetic diversity on interactions between higher trophic levels. Biology Letters, 9: 20130133.
234. Moreira X, Nell CS, Meza-Lopez MM, Rasmann S, Mooney KA, 2018. Specificity of plant-plant communication for *Baccharis salicifolia* sexes but not genotypes. Ecology, 99: 2731-2739.
235. Mundt CC, 1985. Development of crown rust epidemics in genetically diverse oat populations: effect of genotype unit area. Phytopathology, 75: 607-610.
236. Mundt CC, 2002. Performance of wheat cultivars and cultivar mixtures in the presence of *Cephalosporium stripe*. Crop Protection, 21: 93-99.
237. Mundt CC, Brophy LS, Kolar SC, 2003. Effect of genotype unit number and spatial arrangement on severity of yellow rust in wheat cultivar mixtures. Plant Pathology, 45: 215-222.
238. Mundt CC, Brophy LS, Schmitt MS, 1995a. Choosing crop cultivars and cultivar mixtures under low versus high disease pressure: a case study with wheat. Crop Protection, 14: 509-515.
239. Mundt CC, Brophy LS, Schmitt MS, 1995b. Disease severity and yield of pure-line wheat cultivars and mixtures in the presence of eyespot, yellow rust, and their combination. Plant Pathology, 44: 173-182.
240. Mundt CC, Garrett KA, 2000. Host diversity can reduce potato late blight severity for focal and general patterns of primary inoculum. Phytopathology, 90: 1307-1312.
241. Mundt CC, Hayes PM, Schon CC, 1994. Influence of barley variety mixtures on severity of scald and net blotch and on yield. Plant Pathology, 43: 356-361.
242. Mundt CC, Leonard KJ, 1986. Effect of host genotype unit area on development of focal epidemics of bean rust and common maize rust in mixtures of resistant and susceptible plants. Phytopathology, 76: 895-900.
243. Mundt CC, Leonard KJ, Thal WM, Fulton JH, 1986. Computerized simulation of crown rust epidemics in mixtures of immune and susceptible oat plants with different genotype unit areas and spatial distributions of initial disease. Phytopathology, 76: 590-598.
244. Münzbergová Z, Skálová H, Hadincová V, 2009. Genetic diversity affects productivity in early but not late stages of stand development. Basic and Applied Ecology, 10: 411-419.
245. Nault BA, Follett PA, Gould F, Kennedy GG, 1995. Assessing compensation for insect damage in mixed plantings of resistant and susceptible potatoes. American Potato Journal, 72: 157-176.
246. Navabi A, Singh RP, Tewari JP, Briggs KG, 2004. Inheritance of high levels of adult-plant resistance to stripe rust in five spring wheat genotypes. Crop Science, 44: 1156-1162.
247. Newton AC, Ellis RP, Hackett CA, Guy DC, 1997. The effect of component number on *Rhynchosporium secalis* infection and yield in mixtures of winter barley cultivars. Plant Pathology, 46: 930-938.
248. Newton AC, Guy DC, 2009. The effects of uneven, patchy cultivar mixtures on disease control and yield in winter barley. Field Crops Research, 110: 225-228.
249. Newton AC, Guy DC, Nadziak J, Gacek ES, 2002. The effect of inoculum pressure, germplasm selection and environment on spring barley cultivar mixtures efficacy. Euphytica, 125: 325-335.
250. Newton AC, Hackett CA, Swanston JS, 2008. Analysing the contribution of component cultivars and cultivar combinations to malting quality, yield and disease in complex mixtures. Journal of the Science of Food and Agriculture, 88: 2142-2152.
251. Newton AC, Swanston JS, Guy DC, Ellis RP, 1998. The effect of cultivar mixtures on malting quality in winter barley. Journal of the Institute of Brewing, 104: 41-45.
252. Newton AC, Thomas WTB, 1992. The effect of specific and non-specific resistance in mixtures of barley or genotypes on infection by mildew (*Erysiphe graminis* f. sp. *hordei*) and on yield. Euphytica, 59: 73-81.
253. Ngugi HK, King SB, Holt J, Julian AM, 2001. Simultaneous temporal progress of sorghum anthracnose and leaf blight in crop mixtures with disparate patterns. Phytopathology, 91: 720-729.
254. Ning LI, Jia SF, Wang XN, Duan XY, Zhou YL, Wang ZH, Lu G, 2012. The effect of wheat mixtures on the powdery mildew disease and some yield components. Journal of Integrative Agriculture, 11: 611-620.
255. Ninkovic V, Abassi SA, Ahmed E, Glinwood R, Pettersson J, 2011. Effect of within-species plant genotype mixing on habitat preference of a polyphagous insect predator. Oecologia, 166: 391-400.
256. Ninkovic V, Olsson U, Pettersson J, 2002. Mixing barley cultivars affects aphid host plant acceptance in field experiments. Entomologia Experimentalis et Applicata, 102: 177-182.
257. Noto AE, Hughes AR, 2020. Genotypic diversity weakens competition within, but not between, plant species. Journal of Ecology, 108: 2212-2220.
258. Ntahimpera N, Dillard HR, Cobb AC, Seem RC, 1996. Anthracnose development in mixtures of resistant and susceptible dry bean cultivars. Phytopathology, 86: 668-673.
259. Oikawa S, Katayanagi A, Abe A, Matsunami M, Shimono H, 2021. Lodging tolerance of rice is improved without decreasing productivity by mixing lines with different maturity dates. Crop & Pasture Science, 72, 38-43.
260. Olango N, Tusiime G, Mulumba JW, Nankya R, Fadda C, Jarvis DI, Paparu P, 2017. Response of Ugandan common bean varieties to *Pseudocercospora griseola* and Angular leaf spot disease development in varietal mixtures, International Journal of Pest Management, 63: 119-127.
261. Onzo A, Hanna R, Toko M, 2014. Mix-planting pubescent and glabrous cassava affects abundance of *Typhlodromalus aripo* and its prey mite *Mononychellus tanajoa*. Journal of Applied Entomology, 138: 297-306.
262. Pakeman RJ, Brooker RW, Karley AJ, Newton AC, Mitchell C, Hewison RL, Pollenus J, Guy DC, Schöb C, 2020. Increased crop diversity reduces the functional space available for weeds. Weed Research, 60: 121-131.
263. Palvakul M, Finkner VC, Davis DL, 1973. Blendability of phenotypically similar and dissimilar winter barley cultivars. Agronomy Journal, 65: 74-77.

264. Pan P, Qin Y, 2014. Genotypic diversity of soybean in mixed cropping can affect the populations of insect pests and their natural enemies. International Journal of Pest Management, 60: 287-292.
265. Pan P, Qin Y, Zhao Q, Geng Y, Jia Z, Tang Y, 2012. Effects of mixed cropping of different rice varieties on occurrence of insect pests, natural enemies and rice yield. Chinese Journal of Biological Control, 28: 212-219 (in Chinese).
266. Panse A, Davis JHC, Fischbeck G, 1989. Compensation-induced yield gains in mixtures of common bean (*Phaseolus vulgaris* L.). Journal of Agronomy and Crop Science, 162: 347-353.
267. Panse A, Davis JHC, Fischbeck G, 1997. Yield formation in mixtures of rust resistant and susceptible plants of common bean (*Phaseolus vulgaris* L.). Journal of Agronomy and Crop Science, 178: 111-116.
268. Parachnowitsch AL, Cook-Patton SC, Mcart SH, 2014. Neighbours matter: natural selection on plant size depends on the identity and diversity of the surrounding community. Evolutionary Ecology, 28: 1139-1153.
269. Parisi L, Gros C, Combe F, Parveaud CE, Gomez C, Brun L, 2013. Impact of a cultivar mixture on scab, powdery mildew and rosy aphid in an organic apple orchard. Crop Protection, 43: 207-212.
270. Parker JD, Salminen JP, Agrawal AA, 2010. Herbivory enhances positive effects of plant genotypic diversity. Ecology Letters, 13: 553-563.
271. Patterson F, Schafer J, Caldwell R, Compton L, 1963. Comparative standing ability and yield of variety blends of oats. Crop Science, 3: 558-560.
272. Paynter BH, Hills AL, 2008. Mixing feed barley cultivars did not decrease leaf disease or increase grain yield. Australasian Plant Pathology, 37: 626-636.
273. Peacock L, Herrick S, 2000. Responses of the willow beetle *Phratora vulgatissima* to genetically and spatially diverse *Salix* spp. plantations. Journal of Applied Ecology, 37: 821-831.
274. Peltonen-Sainio P, Karjalainen R, 1991. Agronomic evaluation of growing oat cultivar mixtures under various stress conditions in Finland. Acta Agriculturae Scandinavica, 41: 47-53.
275. Pfahler PL, 1965. Environmental variability and genetic diversity within populations of oats (cultivated species of *Avena*) and rye (*Secale cereale* L.). Crop Science, 5: 271-275.
276. Pfahler PL, Linskens HF, 1979. Yield stability and population diversity in oats (*Avena* sp.). Theoretical and Applied Genetics, 54: 1-5.
277. Phillips SL, Shaw MW, Wolfe MS, 2005. The effect of potato variety mixtures on epidemics of late blight in relation to plot size and level of resistance. Annals of Applied Biology, 147: 245-252.
278. Ping X, Xing R, Liu T, 2020. Complementary effect of anti-adversity and yield in different maize (*Zea mays* l.) genotypes intercropping system. Journal of Gansu Agricultural University, 55: 62-67 (in Chinese).
279. Pollnac FW, Smith RG, Warren ND, 2014. Cultivar diversity as a means of ecologically intensifying dry matter production in a perennial forage stand. Ecosphere, 5: 1-12.
280. Power AG, 1988. Leafhopper response to genetically diverse maize stands. Entomologia Experimentalis et Applicata, 49: 213-219.
281. Power AG, 1991. Virus spread and vector dynamics in genetically diverse plant populations. Ecology, 72: 232-241.
282. Prabhu AS, 1990. Cultivar mixtures in panicle blast control in upland rice. Pesquisa Agropecuaria Brasileira, 25: 1183-1192.
283. Pradhanang PM, Sthapit BR, 1995. Effect of cultivar mixtures on yellow rust incidence and grain yield of barley in the hills of Nepal. Crop Protection, 14: 331-334.
284. Prasad R, Rao BRR, 1981. Allelopathy in spring wheat mixtures. Cellular and Molecular Life Sciences, 37: 1078-1078.
285. Prasad R, Sharma SN, 1980. Systematic mixed stands of spring wheat cultivars. Journal of Agricultural Science, 94: 529-532.
286. Pridham JC, Entz MH, Martin RC, Hucl PJ, 2007. Weed, disease and grain yield effects of cultivar mixtures in organically managed spring wheat. Canadian Journal of Plant Science, 87: 855-859.
287. Prieto I, Violle C, Barre P, Durand JL, Ghesquiere M, Litrico I, 2015. Complementary effects of species and genetic diversity on productivity and stability of sown grasslands. Nature Plants, 1: 1-5.
288. Probst AH, 1957. Performance of variety blends in soybeans. Agronomy journal, 49: 148-150.
289. Puy J, Carmona CP, Dvoáková H, Latzel V, de Bello F, 2021. Diversity of parental environments increases phenotypic variation in arabidopsis populations more than genetic diversity but similarly affects productivity. Annals of Botany, 127: 425-436.
290. Qin X, Li Y, Shi C, Song D, Wen X, Liao Y, Siddique KHM, 2019. The number of cultivars in varietal winter-wheat mixtures influence aboveground biomass and grain yield in North China. Plant and Soil, 439:131-143.
291. Qualset CO, Granger RM, 1970. Frequency dependent stability of performance in oats. Crop Science, 10: 386-389.
292. Quénéhervé P, Barrière V, Salmon F, Houdin F, Achard R, Gertrude JC, Marie-Luce S, Chabrier C, Duyck PF, Tixier P, 2011. Effect of banana crop mixtures on the plant-feeding nematode community. Applied Soil Ecology, 49: 40-45.
293. Raboin LM, Ramanantsoanirina A, Dusserre J, Razasolofonanahary F, Sester M, 2012. Two-component cultivar mixtures reduce rice blast epidemics in an upland agrosystem. Plant Pathology, 61: 1103-1111.
294. Ram B, Redhu AS, Singh S, 1989. Development of rusts and powdery mildew in mixtures of wheat varieties. Cereal Research Communications, 17: 195-201.
295. Rao BRR, Prasad R, 1982. Productivity and nutrient uptake by two spring wheat cultivars in pure and mixed stands. Journal of Agronomy & Crop Science, 151: 235-244.
296. Rao BRR, Prasad R, 1984. Intergenotypic competition in mixed stands of spring wheat genotypes. Euphytica, 33: 241-247.
297. Rao BRR, Prasad R, 1985. Competition for water and nutrients in mixed stands of spring wheat. Irrigation Science, 6: 171-178.
298. Rao BRR, Prasad R, 1987. Influence of competition, irrigation levels and nitrogen fertilization on protein content and protein yield of three spring wheat (*Triticum aestivum* L.) cultivars. Plant Foods for Human Nutrition, 37: 127-131.
299. Rasmusson DC, 1968. Yield and stability of yield of barley populations. African Archaeological Review, 26: 600-602.
300. Raynaud T, Pivato B, Siol M, Spor A, Blouin M, 2021. Soil microbes drive the effect of plant species and genotypic diversity interaction on productivity. Plant and Soil, https://doi.org/10.1007/s11104-021-05071-z
301. Reinprecht Y, Schram L, Smith TH, Pauls KP, 2020. Enhancing in-crop diversity in common bean by planting cultivar mixtures

and its effect on productivity. Frontiers in Sustainable Food Systems, 4: 126.
302. Reusch TBH, Ehlers A, Hammerli A, Worm B, 2015. Ecosystem recovery after climatic extremes enhanced by genotypic diversity. Proceedings of the National Academy of Sciences of the United States of America, 102: 2826-2831.
303. Reynolds LK, Chan KM, Huynh E, Williams SL, Stachowicz JJ, 2018. Plant genotype identity and diversity interact with mesograzer species diversity to influence detrital consumption in eelgrass meadows. Oikos, 127: 327-336.
304. Richmond TR, Lewis CF, 1951. Evaluation of varietal mixtures of cotton. Agronomy Journal, 43: 66-70.
305. Rosado R, Carneiro JES, Vieira RF, Paula-Junior TJ, Queiroz TFN, de Carvalh CFM, 2011. Mixture of cultivar of contrasting root architecture increases common bean seed yield. Annual Report of the Bean, 54: 152-153.
306. Ross WM, 1965. Yield of grain sorghum (*Sorghum vulgare* Pers.) hybrids alone and in blends. Crop Science, 5: 593-594.
307. Roy NN, 1976. Inter-genotypic plant competition in wheat under single seed descent breeding. Euphytica, 25: 219-223.
308. Roy SK, 1960. Interaction between rice varieties. Journal of Genetics, 57: 137-152.
309. Sage GCM, 1971. Inter-varietal competion and its possible consequences for the production of f1 hybrid wheat. Journal of Agricultural Science, 77: 491-498.
310. Sakai KI, Suzuki Y, 1955. Studies on competition in plants. II. Competition between diploid and autotetraploid plants of barley. Journal of Genetics, 53: 11-20.
311. Sakai KI, Utiyamada H, 1957. Studies on competition in plants VIII. chromosome number, hybridity and competitive ability *Inoryza sativa* L. Journal of Genetics, 55: 235-240.
312. Salo T, Gustafsson C, 2016. The effect of genetic diversity on ecosystem functioning in vegetated coastal ecosystems. Ecosystems, 19: 1429-1444.
313. Sammons DJ, Baenziger PS, 1985. Performance of four winter wheat cultivars in blended populations. Field Crops Research, 10: 135-142.
314. Sarandon SJ, Sarandon R, 1995. Mixture of cultivars: pilot field trial of an ecological alternative to improve production or quality of wheat (*Triticum aestivum*). Journal of Applied Ecology, 32: 288-294.
315. Schalk JM, Jones A, Dukes PD, Burnham KP, 1992. Responses of soil insects to mixed and contiguous plantings of resistant and susceptible sweetpotato cultivars. HortScience, 27: 1089-1091.
316. Scheepens JF, Rauschkolb R, Ziegler R, Schroth V, Bossdorf O, 2018. Genotypic diversity and environmental variability affect the invasibility of experimental plant populations. Oikos, 127: 570-578.
317. Schilling TT, Mozingo RW, Wynne JC, Isleib TG, 1983. A comparison of peanut multilines and component lines across environments. Crop Science, 23: 101-105.
318. Schmid B, 1994. Effects of genetic diversity in experimental stands of *Solidago altissima*--evidence for the potential role of pathogens as selective agents in plant populations. Journal of Ecology, 82: 165-175.
319. Schöb C, Kerle S, Karley AJ, Morcillo L, Pakeman RJ, Newton AC, Brooker RW, 2015. Intraspecific genetic diversity and composition modify species-level diversity-productivity relationships. New Phytologist, 205: 720-730.
320. Schouls J, Langelaan JG, 1994. Lodging and yield of dry peas (*Pisum sativum* L.) as influenced by various mixing ratios of a conventional and a semi-leafless cultivar. Journal of Agronomy & Crop Science, 172: 207-214.
321. Schutz WM, Brim CA, 1971. Inter-genotypic competition in soybeans. III. an evaluation of stability in multiline mixtures. Crop Science, 11: 684-689.
322. Schweitzer LE, Nyquist WE, Santini JB, Kimes TM, 1986. Soybean cultivar mixtures in a narrow-row, noncultivatable production system. Crop Science, 26: 1043-1046.
323. Shaalan MI, Heyne EG, Lofgren JR, 1966. Mixtures of hard red winter wheat cultivars. Agronomy Journal, 58: 89-91.
324. Sharma AR, 1994. Effect of seed rate and row spacing on the performance of early and late maturing rice cultivars in mixed crop systems under intermediate deepwater conditions (15–50 cm). Journal of Agricultural Science, 122: 201-205.
325. Sharma RC, Dubin HJ, 1996. Effect of wheat cultivar mixtures on spot blotch (*Bipolaris sorokiniana*) and grain yield. Field Crops Research, 48: 95-101.
326. Sharma SN, Prasad R, 1978. Systematic mixed versus pure stands of wheat genotypes. Journal of Agricultural Science, 90: 441-444.
327. Shearman RC, Bishop DM, Steinegger DH, Bruneau AH, 1980. Kentucky bluegrass cultivar response to the bluegrass billbug. HortScience, 18: 441-442.
328. Shen FY, Zhang YF, Xiao ZJ, Li LK, Wei YJ, Yin Y, Chen FJ, 2020. Effect of planting mixed crops of resistant and susceptible soybeans on crop damage by *Spodoptera litura* and the population dynamics of key soybean pests. Chinese Journal of Applied Entomology, 57: 124-133 (in Chinese).
329. Shen J, Liu Z, Cai Y, Zhang W, 2015. Effect of mixture cropping of multiple rice varieties on brown planthopper (*Nilaparvata lugens*) and spiders. Chinese Journal of Biological Control, 31: 327-332 (in Chinese).
330. Shi C, Chen T, Feng F, Wang C，Lv X, Zhang L, Liao Y, Qin X, 2017. Production of mixed planting winter wheat and soil water efficiency in Guanzhong Irrigation Zone. Agricultural Research in the Arid Areas, 35: 29-37 (in Chinese).
331. Shoffner AV, Tooker JF, 2013. The potential of genotypically diverse cultivar mixtures to moderate aphid populations in wheat (*Triticum aestivum* L.). Arthropod-Plant Interactions, 7: 33-43.
332. Shorter R, Frey KJ, 1979. Relative yields of mixtures and monocultures of oat genotypes. Crop Science, 19: 548-553.
333. Sitch L, Whittington WJ, 1983. The effect of variety mixtures on the development of swede powdery mildew. Plant Pathology, 32: 41-46.
334. Smith AL, Atwater DZ, Kim W, Haak DC, Barney JN, 2021. Invasive plant rhizome production and competitiveness vary based on neighbor identity. Journal of Plant Ecology, 14: 638-647.
335. Srinivasan KV, 1968. Effect of a varietal mixture on sugarcane root rot susceptibility and on the rhizosphere microflora. Proceedings of the Indian Academy of Sciences-Section B, 68: 150-162.
336. Ssekandi W, Mulumba JW, Colangelo P, Nankya R, Fadda C,Karungi J, Otim M, De Santis P, Jarvis DI, 2016. The use of common bean (*Phaseolus vulgaris*) traditional varieties and their mixtures with commercial varieties to manage bean fly

(*Ophiomyia* spp.) infestations in Uganda. Journal of Pest Science, 89: 45-57.
337. Sserubombwe WS, Thresh JM, Otim-Nape GW, Osiru DOS, 2001. Progress of cassava mosaic virus disease and whitefly vector populations in single and mixed stands of four cassava varieties grown under epidemic conditions in Uganda. Annals of Applied Biology, 138: 161-170.
338. Stoelen O, Hermansen JE, Loehde J, 1980. Varietal mixtures of barley and their ability to reduce powdery mildew and yellow rust diseases. Kongelige veterinaer og landbohoejskole, 41: 109-116.
339. Stützel H, Aufhammer W, 1989. Effects of winter barley cultivar mixtures on lodging. Journal of Agricultural Science, 112: 47-55.
340. Sumarno, Fehr WR, 1980. Intergenotypic competition between determinate and indeterminate soybean cultivars in blends and alternate rows. Crop Science, 20: 251-254.
341. Sun XS, Chen YH, Zhuo N, Cui Y, Zhang MX, 2021. Effects of salinity and concomitant species on growth of *Phragmites australis* populations at different levels of genetic diversity. Science of The Total Environment, 780: 146516.
342. Suneson CA, 1960. Genetic diversity-a protection against plant diseases and insects. Agronomy Journal, 52: 319-321.
343. Sutton JD, Weaver DB, 1989. Intergenotypic competition between late-planted determinate and indeterminate soybean. Crop Science, 29: 1506-1510.
344. Swanston JS, Newton AC, Brosnan JM, Fotheringham A, Glasgow E, 2005. Determining the spirit yield of wheat varieties and variety mixtures. Journal of Cereal Science, 42: 127-134.
345. Tack AJM, Roslin T, 2011. The relative importance of host-plant genetic diversity in structuring the associated herbivore community. Ecology, 92: 1594-1604.
346. Tang J, Xie J, Chen X, Yu L, 2009. Can rice genetic diversity reduce *Echinochloa crus-galli* infestation? Weed Research, 49: 47-54.
347. Tansey JA, Dosdall LM, Keddie A, 2011. Incorporation of novel *Ceutorhynchus obstrictus*-resistant canola genotypes into mixed cropping strategies: evidence for associational resistance. Journal of Insect Behavior, 24: 116–131.
348. Tapsoba H, Wilson JP, 1999. Increasing complexity of resistance in host populations through intermating to manage rust of pearl millet. Phytopathology, 89: 450-455.
349. Tarhuni AM, McMeilly T, 1989. The effect of component frequency and plant density on yields of cultivar mixtures in spring faba beans. Journal of Agronomy & Crop Science, 163: 256-264.
350. Tarhuni AM, McMeilly T, 1990. Yield comparison of within row mixtures, between row mixtures, and composites of spring faba bean cultivars. Journal of Agronomy & Crop Science, 165: 39-46.
351. Teetes GL, Anderson RM, Peterson GC, 1994. Exploitation of sorghum nonpreference resistance to sorghum midge (Diptera: Cecidomyiidae) using mixed plantings of resistant and susceptible sorghum hybrids. Journal of Economic Entomology, 87: 826-831.
352. Teng F，Chen H，Xiang J，Cai XQ, Xu YC, Zeng YH, Zhu DF, 2014. Resistance changes and efectiveness experiment of increasing yield of rice with mixed planting under mechanical transplanting. Transactions of the Chinese Society of Agricultural Engineering, 30: 17-24 (in Chinese).
353. Tilahun A, 1995. Yield gain and risk minimization in maize (*Zea mays*) through cultivar mixtures in semi-arid zones of the rift valley in Ethiopia. Experimental Agriculture, 31: 161-168.
354. Tomas F, Abbott JM, Steinberg C, Balk M, Williams SL, Stachowicz WJ, 2011. Plant genotype and nitrogen loading influence seagrass productivity, biochemistry, and plant-herbivore interactions. Ecology, 92: 1807-1817.
355. Tomimatsu H, Nakano K, Yamamoto N, Suyama Y, 2014. Effects of genotypic diversity of *Phragmites australis* on primary productivity and water quality in an experimental wetland. Oecologia, 175: 163-172.
356. Tratwal A, Bocianowski J, 2018. Cultivar mixtures as part of integrated protection of spring barley. Journal of Plant Diseases and Protection, 125: 41-50.
357. Tratwal A, Roik K, Bocianowski J, 2014. The effect of growing mixtures of spring barley cultivars on pest occurrence and yields. Polish Journal of Entomology, 83: 295-311.
358. Travis SE, Proffitt CE, 2016. Genotypic interactions limit growth and stimulate flowering in a salt marsh foundation plant species. Perspectives in Plant Ecology, Evolution and Systematics, 18: 33-44.
359. Trimble MW, Fehr WR, 1983. Mixtures of soybean cultivars to minimize yield loss caused by iron-deficiency chlorosis. Crop Science, 23: 691-694.
360. Underwood N, 2009. Effect of genetic variance in plant quality on the population dynamics of a herbivorous insect. Journal of Animal Ecology, 78: 839-847.
361. Utsumi S, Ando Y, Craig TP, Ohgushi T, 2011. Plant genotypic diversity increases population size of a herbivorous insect. Proceedings of the Royal Society B: Biological Sciences, 278: 3108–3115.
362. Valentine J, 1982. Variation in monoculture and in mixture for grain yield and other characters in spring barley. Annals of Applied Biology, 101: 127-141.
363. Vallavieille-Pope CD, 2004. Management of disease resistance diversity of cultivars of a species in single fields: controlling epidemics. Comptes Rendus Biologies, 327: 611-620.
364. Vellend M, Drummond EBM, Tomimatsu H, 2010. Effects of genotype identity and diversity on the invasiveness and invasibility of plant populations. Oecologia, 162: 371-381.
365. Vidal T, Boixel AL, Durand B, de Vallavieille-Pope C, Huber L, Saint-Jean S, 2017. Reduction of fungal disease spread in cultivar mixtures: impact of canopy architecture on rain-splash dispersal and on crop microclimate. Agricultural and Forest Meteorology, 246: 154-161.
366. Vidal T, Saint-Jean S, Lusley P, Leconte M, Krima SB, Boixel AL, Consortium W, de Vallavieille-Pope C, 2020. Cultivar mixture effects on disease and yield remain despite diversity in wheat height and earliness. Plant Pathology, 69: 1148-1160.
367. Villaréal LMMA, Lannou C, 2000. Selection for increased spore efficacy by host genetic background in a wheat powdery mildew population. Phytopathology, 90: 1300-1306.

368. Vlachostergios D, Lithourgidis A, Korkovelos A, Baxevanos D, Mavromatis A, 2011a. Mixing ability of conventionally bred common vetch (vicia sativa l.) cultivars for grain yield under low-input cultivation. Australian Journal of Crop Science, 5: 1588-1594.
369. Vlachostergios DN, Lithourgidis AS, Dordas CA, Baxevanos D, 2011b. Advantages of mixing common vetch cultivars developed from conventional breeding programs when grown under low-input farming system. Crop Science, 51: 1274-1281.
370. Wallace MK, Orf JH, Stienstra WC, 1995. Field population dynamics of soybean cyst nematode on resistant and susceptible soybeans and their blends. Crop Science, 35: 703-707.
371. Walsh EJ, Noonan MG, 1998. Agronomic and quality performance of variety mixtures in spring wheat (*Triticum aestivum* L.) under Irish conditions. Cereal Research Communications, 26: 427-432.
372. Wang XL, Zhang SQ, 2013. Competitiveness of intercropped maize cultivars in the Loess Plateau，China. Chinese Journal of Eco-Agriculture, 21: 1403-1410 (in Chinese).
373. Wang XY, Shen DW, Jiao J, Xu NN, Chen XY, 2012. Genotypic diversity enhances invasive ability of *Spartina alterniflora*. Molecular Ecology, 21: 2542-2551.
374. Wang Y, Zhang Y, Ji W, Yu P, Wang B, Li J, Han M, Xu X,Wang Z, 2016. Cultivar mixture cropping increased water use efficiency in winter wheat under limited irrigation conditions. PLOS ONE, 11: e0158439.
375. Wang Y, Zhao Z, Li J, Zhang M, Zhou S, Wang Z, Zhang Y, 2017. Does maize hybrid intercropping increase yield due to border effects? Field Crops Research, 214: 283-290.
376. Weih M, Nordh NE, Manzoni S, Hoeber S, 2021. Functional traits of individual varieties as determinants of growth and nitrogen use patterns in mixed stands of willow (*Salix* spp.). Forest Ecology and Management, 479: 118605.
377. Weiss MJ, Riveland NR, Reitz LL, Olson TC, 1990. Influence of resistant and susceptible cultivar blends of hard red spring wheat on wheat stem sawfly (Hymenoptera: Cephidae) damage and wheat quality parameters. Journal of Economic Entomology, 83: 255-259.
378. Wetzel WC, Aflitto NC, Thaler JS, 2018. Plant genotypic diversity interacts with predation risk to influence an insect herbivore across its ontogeny. Ecology, 99: 2338-2347.
379. White EM, 1982. The effects of mixing barley cultivars on incidence of powdery mildew (*Erysighe graminis*) and on yield in Northern Ireland. Annals of Applied Biology, 101: 539-545.
380. Wilcox JR, 1985. Dry matter partitioning as influenced by competition between soybean isolines. Agronomy journal, 77: 738-742.
381. Williams SL, 2001. Reduced genetic diversity in eelgrass transplantations affects both population growth and individual fitness. Ecological Applications, 11: 1472-1488.
382. Wolfe MS, 1984. Trying to understand and control powdery mildew. Plant Pathology, 33: 451-466.
383. Wolfe MS, Barrett JA, 1979. Disease in crops: controlling the evolution of plant pathogens. Journal of the Royal Society of Arts, 127: 321-333.
384. Wolfe MS, Barrett JA, 1980. Can we lead the pathogen astray? Plant Disease, 64: 148-155.
385. Wolfe MS, Jeger MJ, 1981. Integrated use of fungicides and host resistance for stable disease control. Philosophical Transactions of the Royal Society B: Biological Sciences, 295: 175-184.
386. Wu H, Lin M, Rensing C, Qin X, Lin W, 2020. Plant-mediated rhizospheric interactions in intraspecific intercropping alleviate the replanting disease of *radix pseudostellariae*. Plant and Soil, 454: 411-430.
387. Xu T, Xu Z, Xu Y, Duan X, 2014. Effects of wheat cultivar mixtures on powdery mildew, wheat yield and grain protein content. Plant Protection, 40: 87093 (in Chinese).
388. Xu X, Zhang F, Wang L, 2018. Effects of different cultivation conditions on the traits, resistance to bacterial leaf streak, and economic benefits of two rice varieties. Plant Protection, 44: 92-98 (in Chinese).
389. Xu Y, Cheng HF, Kong CH, Meiners SJ, 2021. Intra-specific kin recognition contributes to inter-specific allelopathy: A case study of allelopathic rice interference with paddy weeds. Plant, Cell & Environment, https://doi.org/10.1111/pce.14083.
390. Xu Y, Diao Z, Li C, Pan F, Zhan L, Tian Z, Zhang S, Hu X, 2012. Soybean cultivar mixtures for managing soybean cyst nematode. Soil and Crop, 1: 70-78 (in Chinese).
391. Yan J, Zhang Y, Crawford KM, Chen X, Wu J, 2021. Plant genotypic diversity effects on soil nematodes vary with trophic level. New Phytologist, 229: 575-584.
392. Yang B, Ge F, Ouyang F, Parajulee M, 2012. Intra-species mixture alters pest and disease severity in cotton. Environmental Entomology, 41: 1029-1036.
393. Yang GB, Wang S, Tan FZ, Han CB, Liu XY, He CA, Ji CX, Wang H, 2009. Effects of mixed planting of two maize varieties on maize yield and component factors. Journal of Maize Sciences, 17: 104-106 (in Chinese).
394. Yang LN, Pan ZC, Zhu W, Wu EJ, He DC, Yuan X, Qin YY, Wang Y, Chen RS, Thrall PH, Burdon JJ, Shang LP, Sui QJ, Zhan J, 2019. Enhanced agricultural sustainability through within-species diversification. Nature Sustainability, 2: 46-52.
395. Yang LR, Yang JQ, 2017. Comparison of yield between hybrid and single maize varieties in Xishan area. Agriculture and Technology, 37: 38-38 (in Chinese).
396. Yang X, Wang XP, Qu YB, Wu M, Wang JL., Wang ZL, Wang SQ, Zhao NX, Gao YB, 2019. Comparing the effects of companion species diversity and the dominant species (*Stipa grandis*) genotypic diversity on the biomass explained by plant functional trait. Ecological Engineering, 136: 17-22.
397. Yang X, Xu Y, Jiang M, Wang Y, Lu H, Xue Y, Wang J, Zhao N, Gao Y, 2021. Interpreting the effects of plant species diversity and genotypic diversity within a dominant species on above-and belowground overyielding. Science of The Total Environment, 786: 147505.
398. Yoshihara Y, Isogai T, 2019. Does genetic diversity of grass improve yield, digestibility, and resistance to weeds, pests and disease infection? Archives of Agronomy and Soil Science, 65: 1623-1629.
399. Yu YM, 1990. Effect of kenaf mixed with different mature varieties on yield. China's Fiber Crops, 3: 38-39 (in Chinese).
400. Zeng X, Durka W, Fischer M, 2017. Species-specific effects of genetic diversity and species diversity of experimental

communities on early tree performance. Journal of Plant Ecology, 10: 252-258.
401. Zhang HW, Jiang SC, Kong P, Du B, ZHU JQ, 2021. Effects of mixed cropping of rice varieties on an occurrence of Cnaphalocrocis medinalis and rice growth under different rice cropping patterns. Chinese Journal of Ecology, 10.13292/j.1000-4890.202109.033 (in Chinese).
402. Zhao J, Liu M, Yang X, Fu Z, Zi S, 2018. Yield-increasing Effect by Mixed Sowing of Different Prismatic Barley Varieties. Chinese Agricultural Science Bulletin, 34: 21-24 (in Chinese).
403. Zhao J, Liu M, Yang X, Fu Z, Zi SY, 2020. Increase yield of barley cultivars with different rims and different plant height. Jiangsu Agricultural Science, 48: 95-98 (in Chinese).
404. Zhao JF, Chen J, 2012. Comparison experiment of rice biodiversity planting in the same field. Yunnan Agriculture, 2: 30-31 (in Chinese).
405. Zhao L, Sun ZY, Huang C, Wang S, Pan JJ, Wang HG, Cao SQ, Ma ZH, 2010. Study on the control effect and wheat yield of cultivar mixtures to wheat stripe rust. Journal of China Agricultural University, 15: 49-54 (in Chinese).
406. Zhou H, Chen J, Liu Y, Cheng D, Chen L, Sun J, 2009. Using genetic diversity of wheat varieties for ecological regulation on *Sitobion avenae*. Acta Phytophylacica Sinica, 36: 151-156 (in Chinese).
407. Zhou KQ, Wang GD, Li YH, Liu XB, Herbert SJ, Hashemi M, 2014. Assessing variety mixture of continuous spring wheat (*Triticum aestivum* L.) on grain yield and flour quality in Northeast China. International Journal of Plant Production, 8: 91-106.
408. Zhou N, Zhang J, 2012. Effects of mixed planting of different rice varieties on seedling growth. Guangdong Agricultural Sciences, 12: 12-14 (in Chinese).
409. Zhu YY, Chen HR, Fan JH, Wang YY, Li Y, Chen JB, Fan JX, Yang SS, Hu LP, Leung H, Mew TW, Teng PS, Wang ZH, Mundt CC, 2000. Genetic diversity and disease control in rice. Nature, 406: 718-722.
410. Zhu YY, Fang H, Wang YY, Fan JX, Yang SS, Mew TW, Mundt CC, 2005. Panicle blast and canopy moisture in rice cultivar mixtures. Phytopathology, 95: 433-438.
411. Zhu YY, Sun Y，Wang YY, Li Y, He YQ, He XH, Christopher CM, Tom WM, Hei L, 2004. Genetic analysis of rice varietal diversity for rice blast control. Acta Genetica Sinica, 31: 707-716 (in Chinese).
412. Zogg GP, Travis SE, 2021. Genotypic diversity reduces the negative effects of increased competition, herbivory, and tidal inundation on the productivity of *Spartina alterniflora*. Estuaries and Coasts, https://doi.org/10.1007/s12237-021-00962-x.
413. Zuo S, Yongqing MA, Shinobu I, 2008. Ecological adaptation of weed biodiversity to the allelopathic rank of the stubble of different wheat genotypes in a maize field. Weed Biology & Management, 8: 161-171.

The list of 27 review and meta-analysis studies of plant genetic diversity that were referred to in this paper

1. Babu BS, Pandravada SR, Rao RDVJP, Anitha K, Chakrabarty SK, Varaprasad KS, 2011. Global sources of pepper genetic resources against arthropods, nematodes and pathogens. Crop Protection, 30: 389-400.
2. Bailey JK, Schweitzer JA, Ubeda F, Koricheva J, Whitham TG, 2009. From genes to ecosystems: a synthesis of the effects of plant genetic factors across levels of organization. Philosophical Transactions of the Royal Society B Biological Sciences, 364: 1607-1616.
3. Bergman JM, Tingey WM, 1979. Aspects of interaction between plant genotypes and biological control. Bulletin of the Entomological Society of America, 25: 275-279.
4. Bolnick DI, Amarasekare P, Araújo MS, Bürger R, Levine JM, Novak M, Rudolf VHW, Schreiber SJ, Urban MC, Vasseur D, 2011. Why intraspecific trait variation matters in community ecology. Trends in Ecology & Evolution, 26: 183-192.
5. Bonman JM, Khush GS, Nelson RJ, 1992. Breeding rice for resistance to pests. Annual Review of Phytopathology, 30: 507-528.
6. Booy G, Hendriks RJJ, Smulders MJM, Groenendael JM, Vosman B, 2000. Genetic diversity and the survival of populations. Plant Biology, 2: 379-395.
7. Bottrell DG, Barbosa P, Gould F, 1998. Manipulating natural enemies by plant variety selection and modification: a realistic strategy? Annual Review of Entomology, 43: 347-367.
8. Castagneyrol B, Jactel H, 2012. Unraveling plant–animal diversity relationships: a meta-regression analysis. Ecology, 93: 2115-2124.
9. Hazard C, Johnson D, 2018. Does genotypic and species diversity of mycorrhizal plants and fungi affect ecosystem function? New Phytologist, 220: 1122-1128.
10. He HM, Liu LN, Munir S, Bashir NH, Li CY, 2019. Crop diversity and pest management in sustainable agriculture. Journal of Integrative Agriculture, 18: 1945-1952.
11. Kambach S, Kühn I, Castagneyrol B, Bruelheide H, 2016. The impact of tree diversity on different aspects of insect herbivory along a global temperature gradient-a meta-analysis. PLoS ONE, 11: e0165815.
12. Kettenring KM, Mercer KL, Adams CR, Hines J, 2014. Application of genetic diversity-ecosystem function research to ecological restoration. Journal of Applied Ecology, 51: 339-348.
13. Kiær, LP, Skovgaard IM, Østergard H, 2009. Grain yield increase in cereal variety mixtures: a meta-analysis of field trials. Field Crops Research, 114: 361-373.
14. Koricheva J, Hayes D, 2018. The relative importance of plant intraspecific diversity in structuring arthropod communities: a meta-analysis. Functional Ecology, 32: 1704-1717.
15. Lebeda A, Křístková E, Kitner M, Mieslerová B, Jemelková M, Pink DAC, 2014. Wild *Lactuca* species, their genetic diversity, resistance to diseases and pests, and exploitation in lettuce breeding. European Journal of Plant Pathology, 138: 597-640.
16. Moreira X, Abdalaroberts L, Rasmann S, Castagneyrol B, Mooney KA, 2016. Plant diversity effects on insect herbivores and their natural enemies: current thinking, recent findings, and future directions. Current Opinion in Insect Science, 14: 1-7.
17. Mundt CC, 2002. Use of multiline cultivars and cultivar mixtures for disease management. Annual Review of Phytopathology, 40: 381-410.
18. Reiss ER, Drinkwater LE, 2018. Cultivar mixtures: a meta-analysis of the effect of intraspecific diversity on crop yield. Ecological

Applications, 28: 62-77.
19. Salo T, Gustafsson C, 2016. The effect of genetic diversity on ecosystem functioning in vegetated coastal ecosystems. Ecosystems, 19: 1429-1444.
20. Simmonds NW, 1962. Variability in crop plants, its use and conservation. Biological Reviews, 37: 442-465.
21. Smithson JB, Lenné JM, 1996. Varietal mixtures: a viable strategy for sustainable productivity in subsistence agriculture. Annals of Applied Biology, 128: 127-158.
22. Tooker JF, Frank SD, 2012. Genotypically diverse cultivar mixtures for insect pest management and increased crop yields. Journal of Applied Ecology, 49: 974-985.
23. Vellend M, Geber MA, 2005. Connections between species diversity and genetic diversity. Ecology Letters, 8: 767-781.
24. Whitham TG, Bailey JK, Schweitzer JA, Shuster SM, Bangert RK, LeRoy CJ, Lonsdorf EV, Allan GJ, DiFazio SP, Potts BM, Fischer DG, Gehring CA, Lindroth RL, Marks JC, Hart SC, Wimp GM, Wooley SC, 2006. A framework for community and ecosystem genetics: from genes to ecosystems. Nature Reviews Genetics, 7: 510-523.
25. Whitlock R, 2014. Relationships between adaptive and neutral genetic diversity and ecological structure and functioning: a meta-analysis. Journal of Ecology, 102: 857-872.
26. Wolfe MS, 1985. The current status and prospects of multiline cultivars and variety mixtures for disease resistance. Annual Review of Phytopathology, 23: 251-273.
27. Wolfe MS, Mcdermott JM, 1994. Population genetics of plant pathogen interactions: the example of the *Erysiphe graminis-Hordeum vulgare* pathosystem. Annual Review of Phytopathology, 32: 89-113.

PRISMA 2020 flow diagram for new systematic reviews which included searches of databases and registers only

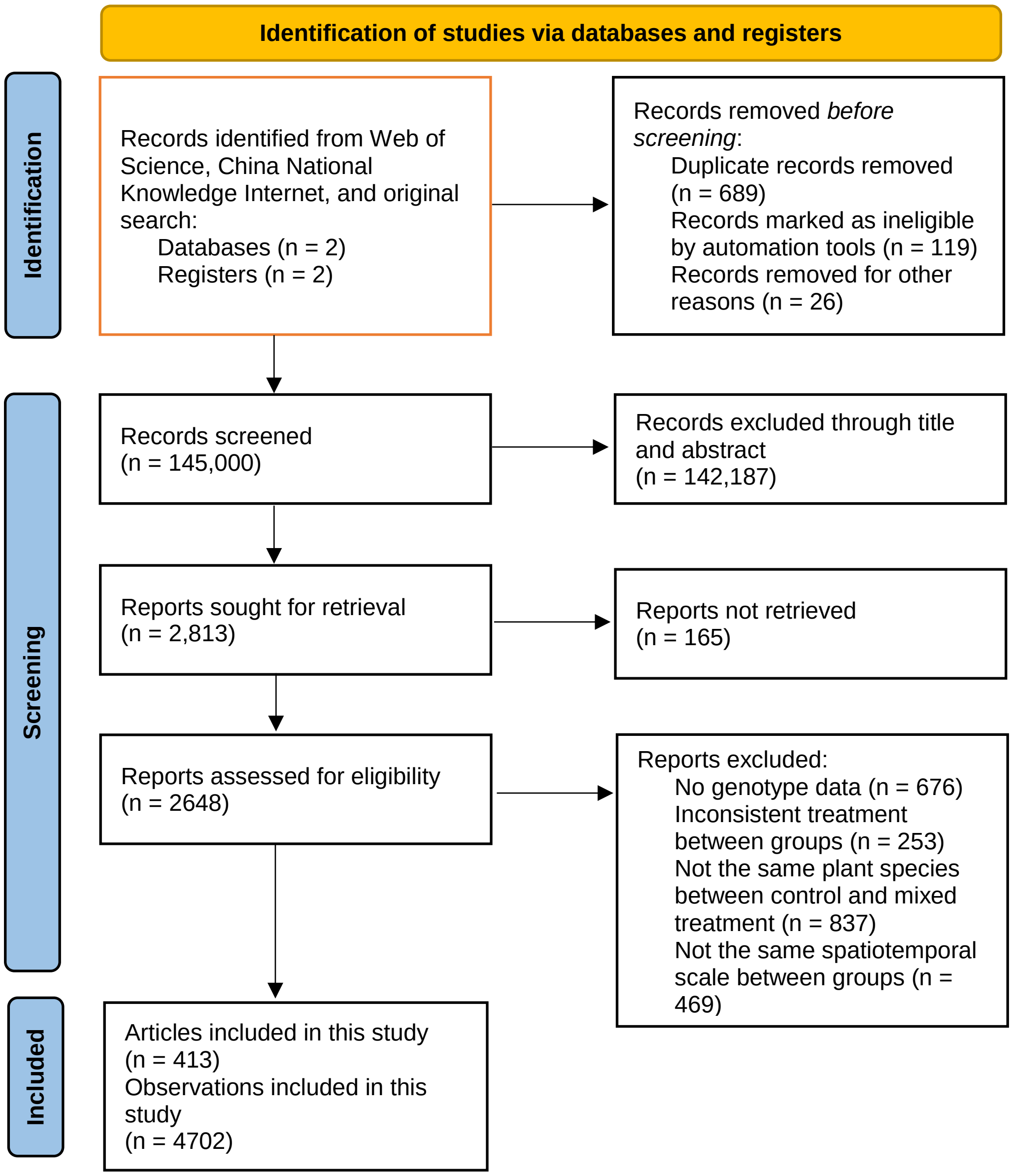

*Consider, if feasible to do so, reporting the number of records identified from each database or register searched (rather than the total number across all databases/registers).

**If automation tools were used, indicate how many records were excluded by a human and how many were excluded by automation tools.

From: Page MJ, McKenzie JE, Bossuyt PM, Boutron I, Hoffmann TC, Mulrow CD, et al. The PRISMA 2020 statement: an updated guideline for reporting systematic reviews. BMJ 2021;372:n71. doi: 10.1136/bmj.n71

For more information, visit: http://www.prisma-statement.org/

PRISMA 2020 Checklist

Section and Topic	Item #	Checklist item	Location where item is reported
**TITLE**			
Title	1	Identify the report as a systematic review.	Page1
**ABSTRACT**			
Abstract	2	See the PRISMA 2020 for Abstracts checklist.	Page2
**INTRODUCTION**			
Rationale	3	Describe the rationale for the review in the context of existing knowledge.	Pages2-5
Objectives	4	Provide an explicit statement of the objective(s) or question(s) the review addresses.	Pages4-5
**METHODS**			
Eligibility criteria	5	Specify the inclusion and exclusion criteria for the review and how studies were grouped for the syntheses.	Pages14-15
Information sources	6	Specify all databases, registers, websites, organisations, reference lists and other sources searched or consulted to identify studies. Specify the date when each source was last searched or consulted.	Pages14-15
Search strategy	7	Present the full search strategies for all databases, registers and websites, including any filters and limits used.	Pages14-15
Selection process	8	Specify the methods used to decide whether a study met the inclusion criteria of the review, including how many reviewers screened each record and each report retrieved, whether they worked independently, and if applicable, details of automation tools used in the process.	Pages14-15
Data collection process	9	Specify the methods used to collect data from reports, including how many reviewers collected data from each report, whether they worked independently, any processes for obtaining or confirming data from study investigators, and if applicable, details of automation tools used in the process.	Pages14-15
Data items	10a	List and define all outcomes for which data were sought. Specify whether all results that were compatible with each outcome domain in each study were sought (e.g. for all measures, time points, analyses), and if not, the methods used to decide which results to collect.	Pages15-17
	10b	List and define all other variables for which data were sought (e.g. participant and intervention characteristics, funding sources). Describe any assumptions made about any missing or unclear information.	Pages15-17
Study risk of bias assessment	11	Specify the methods used to assess risk of bias in the included studies, including details of the tool(s) used, how many reviewers assessed each study and whether they worked independently, and if applicable, details of automation tools used in the process.	Pages17-23
Effect measures	12	Specify for each outcome the effect measure(s) (e.g. risk ratio, mean difference) used in the synthesis or presentation of results.	Pages17-18
Synthesis methods	13a	Describe the processes used to decide which studies were eligible for each synthesis (e.g. tabulating the study intervention characteristics and comparing against the planned groups for each synthesis (item #5)).	Pages18-23
	13b	Describe any methods required to prepare the data for presentation or synthesis, such as handling of missing summary statistics, or data conversions.	Pages17-23
	13c	Describe any methods used to tabulate or visually display results of individual studies and syntheses.	Pages17-23
	13d	Describe any methods used to synthesize results and provide a rationale for the choice(s). If meta-analysis was performed, describe the model(s), method(s) to identify the presence and extent of statistical heterogeneity, and software package(s) used.	Pages14-23
	13e	Describe any methods used to explore possible causes of heterogeneity among study results (e.g. subgroup analysis, meta-regression).	Pages17-23
	13f	Describe any sensitivity analyses conducted to assess robustness of the synthesized results.	Pages17-23
Reporting bias assessment	14	Describe any methods used to assess risk of bias due to missing results in a synthesis (arising from reporting biases).	Pages17-22
Certainty assessment	15	Describe any methods used to assess certainty (or confidence) in the body of evidence for an outcome.	Pages17-22

PRISMA 2020 Checklist

Section and Topic	Item #	Checklist item	Location where item is reported
**RESULTS**			
Study selection	16a	Describe the results of the search and selection process, from the number of records identified in the search to the number of studies included in the review, ideally using a flow diagram.	Page4-5
	16b	Cite studies that might appear to meet the inclusion criteria, but which were excluded, and explain why they were excluded.	Page4-5
Study characteristics	17	Cite each included study and present its characteristics.	Supplementary Data 1
Risk of bias in studies	18	Present assessments of risk of bias for each included study.	Pages6-11
Results of individual studies	19	For all outcomes, present, for each study: (a) summary statistics for each group (where appropriate) and (b) an effect estimate and its precision (e.g. confidence/credible interval), ideally using structured tables or plots.	Supplementary information
Results of syntheses	20a	For each synthesis, briefly summarise the characteristics and risk of bias among contributing studies.	Supplementary information
	20b	Present results of all statistical syntheses conducted. If meta-analysis was done, present for each the summary estimate and its precision (e.g. confidence/credible interval) and measures of statistical heterogeneity. If comparing groups, describe the direction of the effect.	Supplementary information
	20c	Present results of all investigations of possible causes of heterogeneity among study results.	Supplementary information
	20d	Present results of all sensitivity analyses conducted to assess the robustness of the synthesized results.	Supplementary information
Reporting biases	21	Present assessments of risk of bias due to missing results (arising from reporting biases) for each synthesis assessed.	Supplementary information
Certainty of evidence	22	Present assessments of certainty (or confidence) in the body of evidence for each outcome assessed.	Supplementary information
**DISCUSSION**			
Discussion	23a	Provide a general interpretation of the results in the context of other evidence.	Pages6-13
	23b	Discuss any limitations of the evidence included in the review.	Pages7-13
	23c	Discuss any limitations of the review processes used.	Pages9-13
	23d	Discuss implications of the results for practice, policy, and future research.	Pages11-14
**OTHER INFORMATION**			
Registration and protocol	24a	Provide registration information for the review, including register name and registration number, or state that the review was not registered.	Supplementary information
	24b	Indicate where the review protocol can be accessed, or state that a protocol was not prepared.	Supplementary information
	24c	Describe and explain any amendments to information provided at registration or in the protocol.	Supplementary information
Support	25	Describe sources of financial or non-financial support for the review, and the role of the funders or sponsors in the review.	Page27
Competing interests	26	Declare any competing interests of review authors.	Page28

PRISMA 2020 Checklist

Section and Topic	Item #	Checklist item	Location where item is reported
Availability of data, code and other materials	27	Report which of the following are publicly available and where they can be found: template data collection forms; data extracted from included studies; data used for all analyses; analytic code; any other materials used in the review.	Page23

From: Page MJ, McKenzie JE, Bossuyt PM, Boutron I, Hoffmann TC, Mulrow CD, et al. The PRISMA 2020 statement: an updated guideline for reporting systematic reviews. BMJ 2021;372:n71. doi: 10.1136/bmj.n71

For more information, visit: http://www.prisma-statement.org/
