# Supplementary material for: Plant genetic diversity affects multiple trophic levels and trophic interactions
Source: Nat Commun. 2022 Nov 27;13:7312. doi: 10.1038/s41467-022-35087-7 (PMC9701765; doi:10.1038/s41467-022-35087-7)
Supplement: Supplementary file 3 — Description of Additional Supplementary Files [file 41467_2022_35087_MOESM3_ESM.pdf]

Description of Additional Supplementary Files

File Name: Supplementary Data 1
Description: Overview of the 413 studies covering 4702 observations included in the meta-analysis of the effects of plant genetic diversity on invertebrate herbivores, predators, parasitoids, weeds, plant-feeding nematodes, plant disease and plants. For each paper, the table indicates whether it provides information on abundance (A), diversity (D), and impact (I, indicating herbivore damage, and parasitism, weed growth, disease spread, disease damage, respectively) of the herbivores, predators and parasitoids, as well as information on plant growth (G), reproductive output (R) and quality (Q). The trophic interaction column indicates the study type of interactions.
