# Supplementary material for: Plant genetic diversity affects multiple trophic levels and trophic interactions
Source: Nat Commun. 2022 Nov 27;13:7312. doi: 10.1038/s41467-022-35087-7 (PMC9701765; doi:10.1038/s41467-022-35087-7)
Supplement: Supplementary file 5 — Reporting Summary [file 41467_2022_35087_MOESM5_ESM.pdf]

nature portfolio

Corresponding author(s): Nian-Feng Wan

Last updated by author(s): Nov 15, 2022

Reporting Summary

Nature Portfolio wishes to improve the reproducibility of the work that we publish. This form provides structure for consistency and transparency in reporting. For further information on Nature Portfolio policies, see our Editorial Policies and the Editorial Policy Checklist.

Statistics

For all statistical analyses, confirm that the following items are present in the figure legend, table legend, main text, or Methods section.

n/a	Confirmed	
☐	☒	The exact sample size (n) for each experimental group/condition, given as a discrete number and unit of measurement
☐	☒	A statement on whether measurements were taken from distinct samples or whether the same sample was measured repeatedly
☐	☒	The statistical test(s) used AND whether they are one- or two-sided<br>*Only common tests should be described solely by name; describe more complex techniques in the Methods section.*
☐	☒	A description of all covariates tested
☐	☒	A description of any assumptions or corrections, such as tests of normality and adjustment for multiple comparisons
☐	☒	A full description of the statistical parameters including central tendency (e.g. means) or other basic estimates (e.g. regression coefficient) AND variation (e.g. standard deviation) or associated estimates of uncertainty (e.g. confidence intervals)
☐	☒	For null hypothesis testing, the test statistic (e.g. F, t, r) with confidence intervals, effect sizes, degrees of freedom and P value noted<br>*Give P values as exact values whenever suitable.*
☒	☐	For Bayesian analysis, information on the choice of priors and Markov chain Monte Carlo settings
☐	☒	For hierarchical and complex designs, identification of the appropriate level for tests and full reporting of outcomes
☐	☒	Estimates of effect sizes (e.g. Cohen's d, Pearson's r), indicating how they were calculated

Our web collection on statistics for biologists contains articles on many of the points above.

Software and code

Policy information about availability of computer code

Data collection	We conducted a literature search on the Web of Science and China National Knowledge Internet (www.cnki.net) (last accessed in September 2021) using the Boolean search string: [“plant genetic diversity” OR “plant genotypic diversity” OR “crop genetic diversity” OR “crop genotypic diversity” OR “intraspecific diversity” OR “inter-genotypic” OR “intervarietal” OR “resistant *susceptible cultivar*” OR “pure * mixed cultivar” OR “cultivar mixture” OR “varietal mixture”] AND [“predat*” OR “herbivor*” OR “parasitoid” OR “wasp*” OR “natural enem*” OR “pest management” OR “pest control” OR “biological control” OR “plant disease” OR “plant virus” OR “nematode” OR “weed” OR “yield” OR “productivity” OR “biomass”]. Means, standard errors (or standard deviations) and sample sizes of the selected variables could be extracted from tables, figures, the main text or supporting information. The code used to analyse data is deposited to Zenodo: https://zenodo.org/record/7307292#.Y2tzsMhzmLl.
Data analysis	R version 4.1.0 was used for all statistical analyses. The R package ‘metafor’ 3.4-0 was used for performing meta-regression and analysis of publication bias. Piecewise structural equation models were performed using the R package ‘piecewiseSEM’ in conjunction with the R package ‘nlme’. The significance level 0.05 was used for all tests.

For manuscripts utilizing custom algorithms or software that are central to the research but not yet described in published literature, software must be made available to editors and reviewers. We strongly encourage code deposition in a community repository (e.g. GitHub). See the Nature Portfolio guidelines for submitting code & software for further information.

Data

Policy information about availability of data

All manuscripts must include a data availability statement. This statement should provide the following information, where applicable:

- Accession codes, unique identifiers, or web links for publicly available datasets
- A description of any restrictions on data availability
- For clinical datasets or third party data, please ensure that the statement adheres to our policy

All data needed to evaluate the conclusions in the paper are present in the paper and the Supplementary Information. The raw and processed data used in this study is available in the Supplementary Files (Supplementary Data 1) and is deposited to Zenodo: https://zenodo.org/record/7302775#.Y2tz8MhzmLk. The code used to analyse data is deposited to Zenodo: https://zenodo.org/record/7307292#.Y2tzsMhzmLl.

Field-specific reporting

Please select the one below that is the best fit for your research. If you are not sure, read the appropriate sections before making your selection.

☐ Life sciences ☐ Behavioural & social sciences ☒ Ecological, evolutionary & environmental sciences

For a reference copy of the document with all sections, see nature.com/documents/nr-reporting-summary-flat.pdf

Ecological, evolutionary & environmental sciences study design

All studies must disclose on these points even when the disclosure is negative.

Study description	We conducted a literature search on the Web of Science and China National Knowledge Internet (www.cnki.net) (last accessed in September 2021) using the Boolean search string: ["plant genetic diversity" OR "plant genotypic diversity" OR "crop genetic diversity" OR "crop genotypic diversity" OR "intraspecific diversity" OR "inter-genotypic" OR "intervarietal" OR "resistant *susceptible cultivar*" OR "pure * mixed cultivar" OR "cultivar mixture" OR "varietal mixture"] AND ["predat*" OR "herbivor*" OR "parasitoid" OR "wasp*" OR "natural enem*" OR "pest management" OR "pest control" OR "biological control" OR "plant disease" OR "plant virus" OR "nematode" OR "weed" OR "yield" OR "productivity" OR "biomass"]. Overall, about 145000 papers were screened for relevance and 413 were finally selected. Means, standard errors (or standard deviations), and sample sizes of the selected variables could be extracted from tables, figures, the main text or supporting information. Data extraction from figures was conducted with Get Data Graph Digitizer 2.25. When we obtained the data, we analyzed the effect size of response to plant genetic diversity for trophic groups, the effect of plant genetic diversity on bi-trophic/tri-trophic associations and finally analyzed the effect of plant genetic diversity across trophic levels.
Research sample	We followed the PRISMA protocols for study selection and inclusion in the systematic meta-analysis. We collected data from Web of Science and China National Knowledge Internet (www.cnki.net), finally we collected 4702 observations from 413 studies to study the effects of plant genetic diversity on multiple trophic groups (i.e., plants, plant antagonists, invertebrate herbivores, weeds, plant-feeding nematodes, plant diseases and natural enemies of herbivores). In addition, 1484 interactive effect sizes derived from 139 studies were used to test the effects of plant genetic diversity on the bi-trophic interactions between plant antagonist and plant performances, and 91 interactive effect sizes derived from 13 studies were used to test the effects of plant genetic diversity on the tri-trophic interactions among natural enemies, herbivores and plant performances.
Sampling strategy	With the aim of constructing a comprehensive database, we tried to collect as many as experiments that fulfill our criteria (described below). About 145000 papers were reviewed for relevance and 413 were finally selected based on the following criteria: (1) the study included at least one comparison between plant stands with one genotype (monoculture control treatment) or ≥2 genotypes (mixed treatment); (2) the use of pesticides and other practices (fertilizer, irrigation, etc) should be the same for the control and mixed treatments; (3) both the control and the mixed treatment had one and the same plant species; (4) the measurements of treatment and control groups were performed at the same spatiotemporal scale; (5) when a study covered multiple levels of plant genotypes, measurements of monoculture stands and different numbers of plant genotypes were recognized as independent observations; and (6) when a study included different levels of plant species addition, measurements for each plant species addition level were considered to be independent observations. Data extraction from figures was conducted with Get Data Graph Digitizer 2.25. We first used the data for which the authors in a cited paper had listed the mean values of multiple sampling dates or multiple sampling years. If the authors did not present these mean values, we adopted the data of the latest sampling date.
Data collection	First, we selected the papers through a search on the Web of Science and China National Knowledge Internet (www.cnki.net) (last accessed in September 2021), and then extracted the data from the papers. Second, we established a datasheet for trophic groups (invertebrate herbivores, weeds, plant-feeding nematodes, plant diseases, plant antagonist, predators, parasitoids and plants). In this datasheet, we included weed diversity, weed growth, herbivore abundance, herbivore diversity, herbivore damage, disease spread, disease damage, nematode abundance, predator abundance, predator diversity, parasitoid abundance, parasitoid diversity, parasitism, plant growth, plant reproduction, plant quality, plant antagonist intensity and plant antagonist diversity. Nian-Feng Wan and Liwan Fu recorded the data which were recorded in Excel.
Timing and spatial scale	The deadline for collecting data is September 2021 (searches were performed in Web of Science and China National Knowledge Internet between July and September 2021). Searches included globally published studies but were limited to those published in English and Chinese.
Data exclusions	When we collected the data, the data were excluded in this meta-analysis if they did not conform to the following criteria: (1) the

	study included at least one comparison between plant stands with one genotype (monoculture control treatment) or ≥2 genotypes (mixed treatment); (2) the use of pesticides and other practices (fertilizer, irrigation, etc) should be the same for the control and mixed treatments; (3) both the control and the mixed treatment had one and the same plant species; (4) the measurements of treatment and control groups were performed at the same spatiotemporal scale; (5) when a study covered multiple levels of plant genotypes, measurements of monoculture stands and different numbers of plant genotypes were recognized as independent observations; and (6) when a study included different levels of plant species addition, measurements for each plant species addition level were considered to be independent observations.
Reproducibility	The readers can extract the data from the 413 papers which were selected in this paper to reproduce the results. R code is provided to re-run all analyses
Randomization	We used a mixed-effects meta-regression to account for between-study, within-study and sampling variances.
Blinding	Not applicable. There were no human or animal research participant involved in this study.

Did the study involve field work? ☐ Yes ☒ No

Reporting for specific materials, systems and methods

We require information from authors about some types of materials, experimental systems and methods used in many studies. Here, indicate whether each material, system or method listed is relevant to your study. If you are not sure if a list item applies to your research, read the appropriate section before selecting a response.

Materials & experimental systems

n/a	Involved in the study
☒	☐ Antibodies
☒	☐ Eukaryotic cell lines
☒	☐ Palaeontology and archaeology
☒	☐ Animals and other organisms
☒	☐ Human research participants
☒	☐ Clinical data
☒	☐ Dual use research of concern

Methods

n/a	Involved in the study
☒	☐ ChIP-seq
☒	☐ Flow cytometry
☒	☐ MRI-based neuroimaging
